# Base-promoted direct amidation of esters: beyond the current scope and practical applications†

**DOI:** 10.1039/d2ra03524c

**Published:** 2022-07-15

**Authors:** Ivaylo Slavchev, Jas. S. Ward, Kari Rissanen, Georgi M. Dobrikov, Svilen Simeonov

**Affiliations:** Institute of Organic Chemistry with Centre of Phytochemistry, Bulgarian Academy of Sciences Acad. G. Bonchev Str., Bl. 9 Sofia 1113 Bulgaria svilen.simeonov@orgchm.bas.bg; University of Jyvaskyla, Department of Chemistry Survontie 9 B 40014 Jyväskylä Finland; Research Institute for Medicines (iMed.ULisboa), Faculty of Pharmacy, Universidade de Lisboa Av. Prof. Gama Pinto 1649-003 Lisbon Portugal

## Abstract

The base-promoted direct amidation of unactivated esters is among the most useful reactions for amide bond formation in contemporary organic chemistry. The intensive research in this area has led to the development of a number of new methods to achive this transformation. However, to date, the existing literature is more methodological and in many instances lacks practical directions. Therefore, the full potential of this transformation is yet to be revealed by broadening the substrate scope. In a search for new practical applications of the amidation reaction, herein we present a comprehensive study of a number of base-promoted direct amidations that encompass a wide range of amines and esters. Furthermore, we applied our findings in the synthesis of phosphoramidates and several industrially relevant products.

## Abstract 2

A new method for direct amidation of unactivated esters with unprecedented substrate scope is developed.

## Graphical abstract

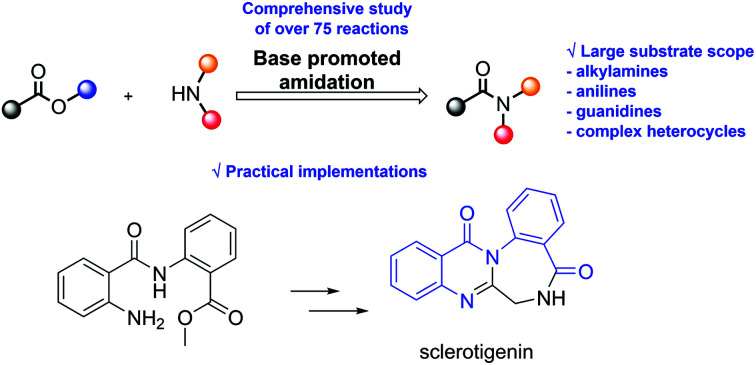

## Introduction

Due to its importance for organic synthesis and its ubiquity in nature, the amide bond is among the most fundamental functionalities in organic molecules. Therefore, new strategies are always needed to meet the contemporary synthetic challenges. Classical methods, using coupling agents,^1^ metal catalysts^2^ or harsh reaction conditions,^3^ have become less and less favored due to several issues, such as atom economy,^4^ energy efficiency, and poor green chemistry metrics.

**Scheme 1:**
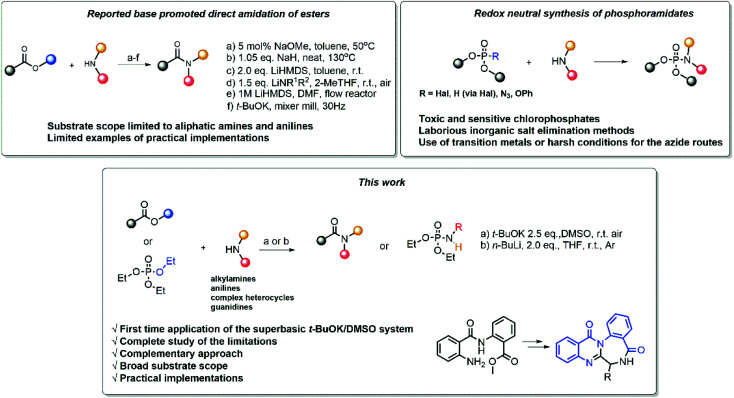
Synthesis of amides and phosphoramidates *via* direct amidation.

Due to their availability, the direct amidation of esters^5^ is among the most attractive methods to achieve the synthesis of amides. Different methods have been developed (Scheme 1), utilizing different bases, solvents and reaction conditions (Scheme 1a–d),^6–9^ including settings for flow chemistry (Scheme 1e),^10^ and mixer mill (Scheme 1f).^11^

However, despite their straightforwardness at first glance, these transformations are often challenging. Among the already developed methods, some limitations can be pointed out. In many instances the existing literature suffers from non-systematic approach, fractionalized data, lack of diversity in the used substrates and a complete overview of the reaction's scope and limitations. Virtually there are no reports of unsuccessful experiments, which can create a misconception that the reaction is universal towards all esters and amines. To date, the practical application, *i.e.* the implementation of the new reaction setting for the synthesis of valuable synthetic products, has received less research focus, and thus needs additional diligence.

Phosphoramidates are another important class of compounds, which finds applications spanning agriculture,^12^ organic synthesis,^13^ pharmaceutical and medicinal chemistry.^14,15^ To date, the synthesis of phosphoramidates mainly rely on oxidative cross-coupling or reduction routes. The preparation of these compounds by redox neutral methods remains rather unexplored and suffers from various drawbacks, such as the use of toxic and sensitive chlorophosphonates,^17^ laborious inorganic salt elimination methods^18–20^ or the use of harsh conditions^21^ and transition metal catalysis^22,23^ (Scheme 1).

Driven by these considerations, herein we report a detailed study of direct amidation of unactivated esters with amines, promoted by strong bases. Our work represents a systematic study of over 75 reactions that encompass large substrate scope, ranging from anilines and heterocyclic amines to aliphatic amines and guanidines, and stretching to the synthesis of phosphoramidates. We performed a thorough examination of the scope and the limitations of the reaction settings and applied them in the synthesis of several important derivatives.

## Results and discussion

### DMSO/*t*-BuOK promoted amidation of esters

The amidation of esters, promoted by potassium *tert*-butoxide has been reported in several instances.^11,24,25^ However, the reactions outcome was found to largely depend on the nature of both ester and amine reaction partners. For instance, Kim *et al.*^24^ reported a difference of approx. 30% higher yield for ethyl benzoate as compared to its methyl analogue. Similar differences could be found with respect to the amine counterparts.

**Scheme 2:**
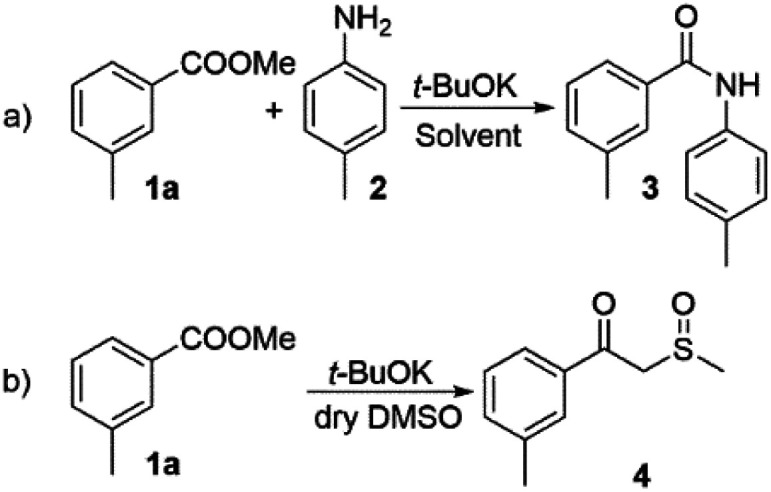
(a) *t*-BuOK promoted direct amidation; (b) possible side reaction under DMSO/*t*-BuOK conditions.

We started our investigation by exploring the reaction between methyl 3-methyl benzoate (1a) and 4-methyl aniline (2) (Scheme 2a). Under similar conditions to those, reported by Kim *et al.* we observed moderate yield of compound 3 (62%, Table 1, entry 1), which is in accordance with the literature data. The use of DMF as a solvent did not provide any significant advantage (Table 1, entry 2). In an attempt to solve this issue we decided to increase the basicity by using DMSO/*t*-BuOK system, which is reported to behave as a superbase.^26^ To the best of our knowledge DMSO/*t*-BuOK has never been reported as a reaction medium for the direct amidation of esters. A rational explanation of this observation is the possible competition between the amine nucleophile and the formed *in situ* sulphur ylide nucleophilic species^27^ (Scheme 2b). To our delight we observed significant improvement of the reaction yield up to 94% (Table 1, entry 3), without the formation of the competitive side product 4 (Scheme 2b) and only hydrolysis of 1a was observed in trace amount as a side reaction. The attempt to decrease the required equivalents of *t*-BuOK resulted in a significant decrease of the yield (Table 1, entries 4 and 5).

**Table 1:** Optimization of the synthesis of amide 3^a^

	Solvent	Concentration (amine)	Eq. (ester)	Eq. (*t*-BuOK)	Yield
1	THF	1 mmol ml^−1^	1.3	2.0	62%
2	DMF	1 mmol ml^−1^	1.3	2.0	70%
3	DMSO	1 mmol ml^−1^	1.3	2.0	94%
4	DMSO	1 mmol ml^−1^	1.0	0.3	Traces
5	DMSO	1 mmol ml^−1^	1.0	1.5	84%

a Reaction time 5 min.

**Scheme 3:**
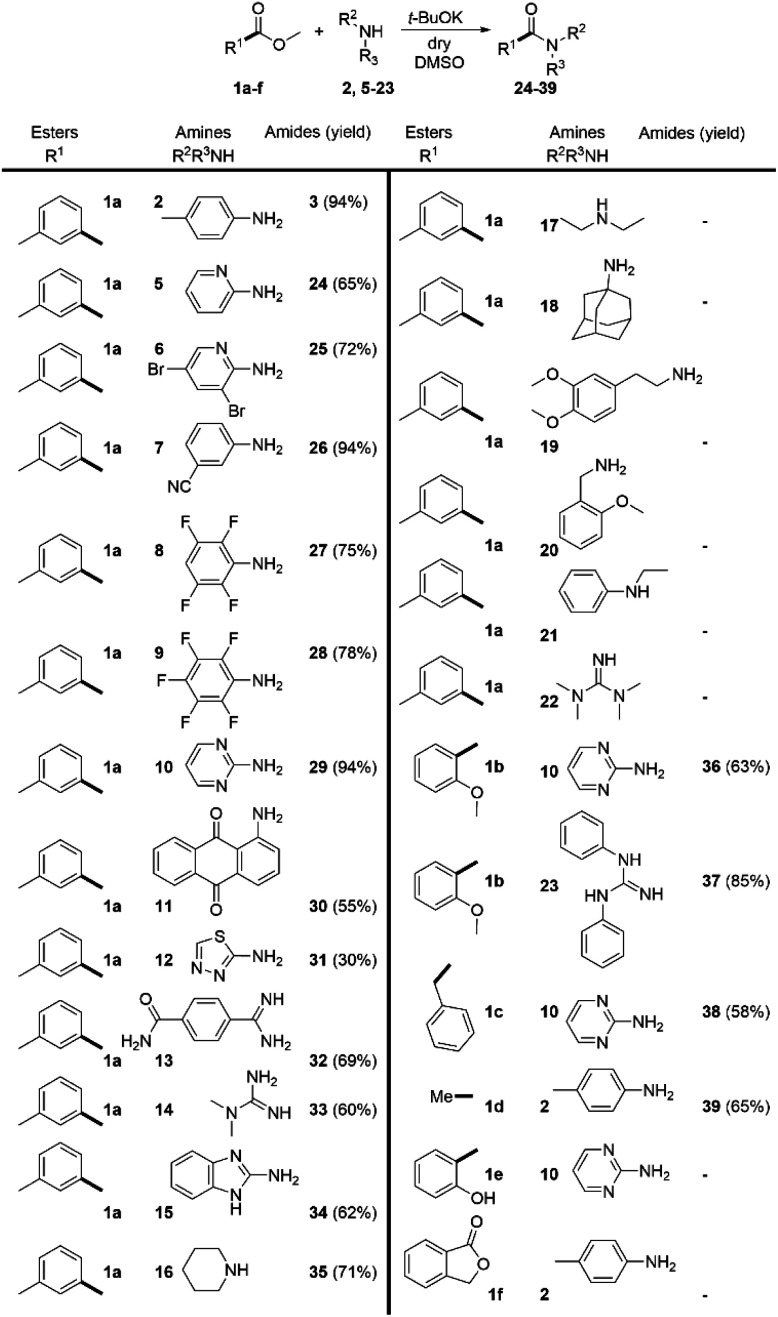
Substrate scope of the DMSO/*t*-BuOK system. Reaction conditions: 1.0 eq. of amine and 2.0 eq. of ester were dissolved in dry DMSO, and then t-BuOK (2.5 eq.) was added slowly portionwise. Reaction time 5 min.

Having these reaction conditions in hand, we explored the amine scope by using methyl 3-methylbenzoate (1a) as a model compound (Scheme 3). The reaction proceeded smoothly with various substituted anilines (2, 5–7). The presence of aliphatic, halogen or cyano-substituents at the aromatic ring was tolerated and the desired amides were isolated in moderate to excellent yields. The scope of the reaction was broadened by the acylation of two very weakly nucleophilic fluorinated anilines (8 and 9) in high yields. Notably, the challenging acylation of 2-aminopyrimidine (10) and the highly deactivated aminoanthraquinone (11) was achieved in 94% and 55% yield respectively. The heterocyclic amine 12 provided the desired product 31 in 30% yield. The reaction scope was extended with imidamide 13 and guanidine 14 and the heterocyclic aromatic amine 15 which were acylated in good yields. Nevertheless, the applicability of the method to aliphatic amines proved very limited. The only aliphatic amine that has been successfully acylated under these conditions was piperidine (16). Surprisingly, the use of tetramethylguanidine 22 was also unsuccessful.

We extended the scope of the method towards the acylation of methyl 2-methoxybenzoate (1b) with 2-aminopyrimidine (10) and *N*,*N*′-diphenylguanidine (23). The desired products 36 and 37 were formed in 63% and 85% yield respectively. CH-acidic esters methyl 2-phenylacetate (1c) and ethyl acetate (1d) were also successfully subjected to direct amidation with 2-aminopyrimidine (10) and 4-methylaniline (2) in moderate yields. However, the reaction of 2-aminopyrimidine with methyl 2-hydroxybenzoate (1e) and the attempt to amidate lactone 1f with aniline 2 were unsuccessful.

### *n*-BuLi promoted amidation of esters

In order to overcome the limitations of the DMSO/*t*-BuOK system, we decided to study a complimentary approach, which would allow us to comparably broaden the scope. Based on the data, reported in the literature^28^ and common synthetic rationale, our choice fell on *n*-BuLi. The ester 1a was again used as a model substrate.

**Scheme 4:**
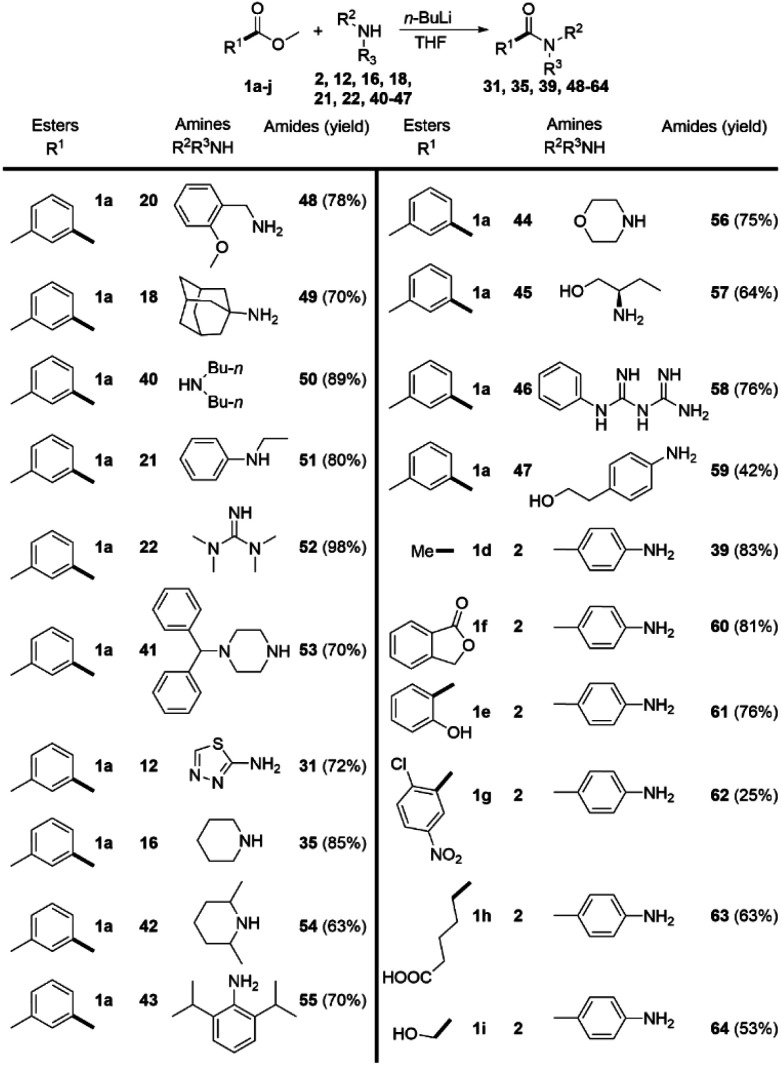
*n*-BuLi promoted direct amidation of esters. Reaction conditions: 1.0 eq. of the corresponding amine and 2.0 eq. ester; THF as solvent, and *n*-BuLi (2.0 eq.) as a base (at r.t. in inert atmosphere). Reaction time 5 min.

We methodically tested the classes of amine substrate (Scheme 4), which did not react or provided poor yields under the previous reaction conditions. In contrast with the DMSO/*t*-BuOK system benzyl amine (20), primary (18) and secondary (40) alkylamines, were successfully acylated, as well as alkyl-phenylamine (21), tetramethylguanidine (22), and *N*-monosubstituted piperazine (41). The products 31 and 35, which were previously obtained in poor yields, were isolated in significantly improved yields, 72% and 85% (Scheme 4) *versus* 30% and 71% (Scheme 3), respectively. The substrate scope was extended to the sterically hindered amines 42 and 43, morpholine 44, aminoalcohol 45, biguanide 46 and aniline 47. Acetamide 39 was obtained in a higher yield compared to the DMSO/*t*-BuOK system. The opening of lactone 1f and the amidation of 2-hydroxybenzoate 1e were also successfully providing the desired products 60 and 61 in high yields. The amidation of 3-nitro-2-chloro benzoate 1g with aniline 2 rendered product 62 in poor yield due to extensive formation of side products, showing that this reaction conditions do not tolerate nitro substituents. Esters containing carboxy (1h) and hydroxyl (1i) groups were also successfully amidated.

**Scheme 5:**
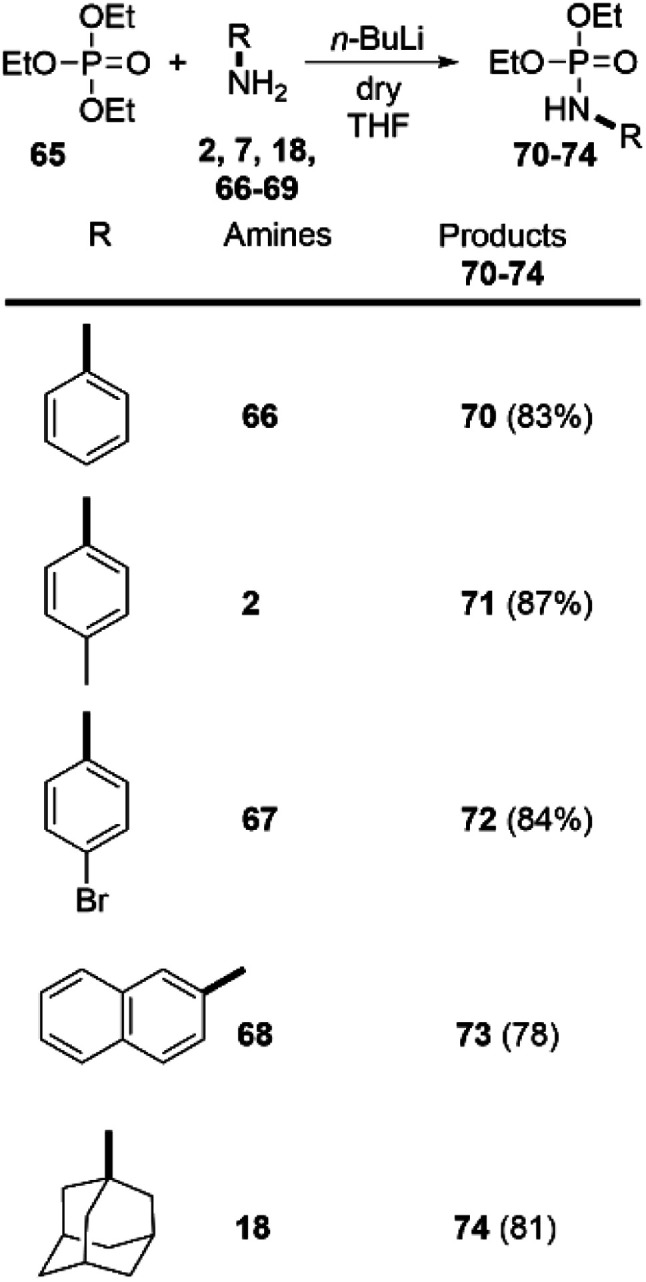
Synthesis of phosphoramidates by direct amidation of triethylphosphate. Reaction conditions: 1.0 eq. of the corresponding amine and 2.0 eq. triethylphosphate; THF as solvent, and *n*-BuLi (2.0 eq.) as a base (at r.t. in inert atmosphere). Reaction time 5 min.

Despite the numerous methods to achieve the synthesis of phosphoramidates^16^ the direct reaction of amines with triethylphosphate (65), without any catalyst, to the best of our knowledge, has never been reported before. Driven by these considerations, we decided to go beyond the carboxylic esters and apply our findings in the amidation of readily available 65. To our delight, this approach was successful and we were able to synthetize phosphoramidates 70–74. Both aromatic and aliphatic amines were applicable as substrates providing the desired products in very high yields (Scheme 5). Noteworthy, the synthesis of similar phosphoramidates was achieved by Tf_2_O-promoted activating strategy. However, this approach provided only low to moderate yields.^29^

### Methods comparison

Based on the extensive investigation of the reaction scope of the two methods, we outlined some advantages and drawbacks. The DMSO/*t*-BuOK-promoted amidation of esters is simple and affordable. It requires a minimum amount of solvent and no heating or cooling. The reactions are practically instantaneous and no inert atmosphere is required. The substrate scope cover wide range of aromatic amines, and its ability to acylate unreactive amines like 2-aminopyrimidine and *N*,*N*′-diphenylguanidine is particularly valuable.

Despite its great potential this method suffers from several drawbacks. It is generally not applicable to important classes of substrates, such as aliphatic and benzylic amines. It's worth mentioning that in all the unsuccessful experiments, the competing reaction of the esters with the DMSO, derived sulfur ylide, leading to the formation of product 4 (Scheme 1). This observation hampers further optimization of the reaction conditions (time, temperatures, *etc.*), due to the fact that in the case of slowly reacting amines, all the ester is predominantly consumed in the faster competitive reaction.

In contrast, the amidation of esters with THF/*n*-BuLi is applicable to a broader substrate scope. Various aliphatic and aromatic amines can be easily acylated with high to excellent yields. The reaction itself is clean and fast, with a straightforward work-up. The method is further applicable towards the synthesis of phosphoramidates. Nevertheless, the work with aggressive base such as *n*-BuLi that requires inert atmosphere is a major drawback. In several instances, the low solubility of some substrates in THF limited the scope of the reaction.

**Fig. 1:**
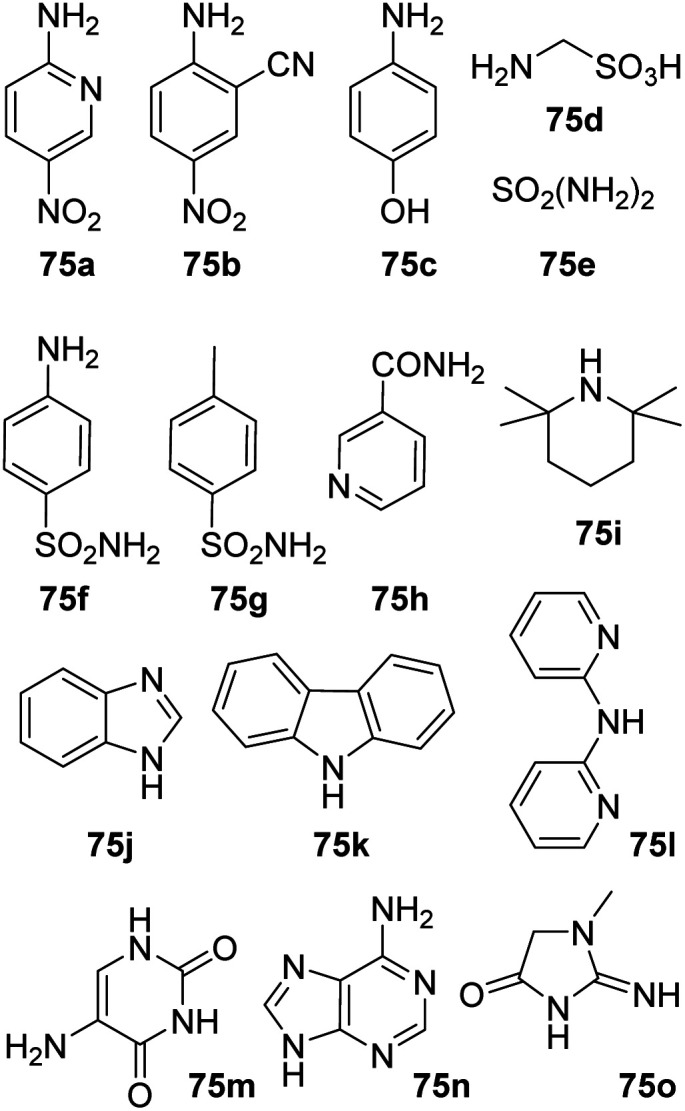
Not applicable substrates.

Amine substrates that were not applicable under both reaction condition are summarized in Fig. 1. Both reaction settings failed in the amidation of 1a with nitroanilines (75a–b) resulting in a complex mixture of products. No conversion was observed during our attempts to acylate aminophenol (75c), diphenylamines (75k–l) and primary amide (75h). Several amine substrates, containing sulfonamido group (75d–g) as well as some highly sterically hindered amines (75i) and heterocycles (75j, 75m–o) were also not applicable.

**Scheme 6:**
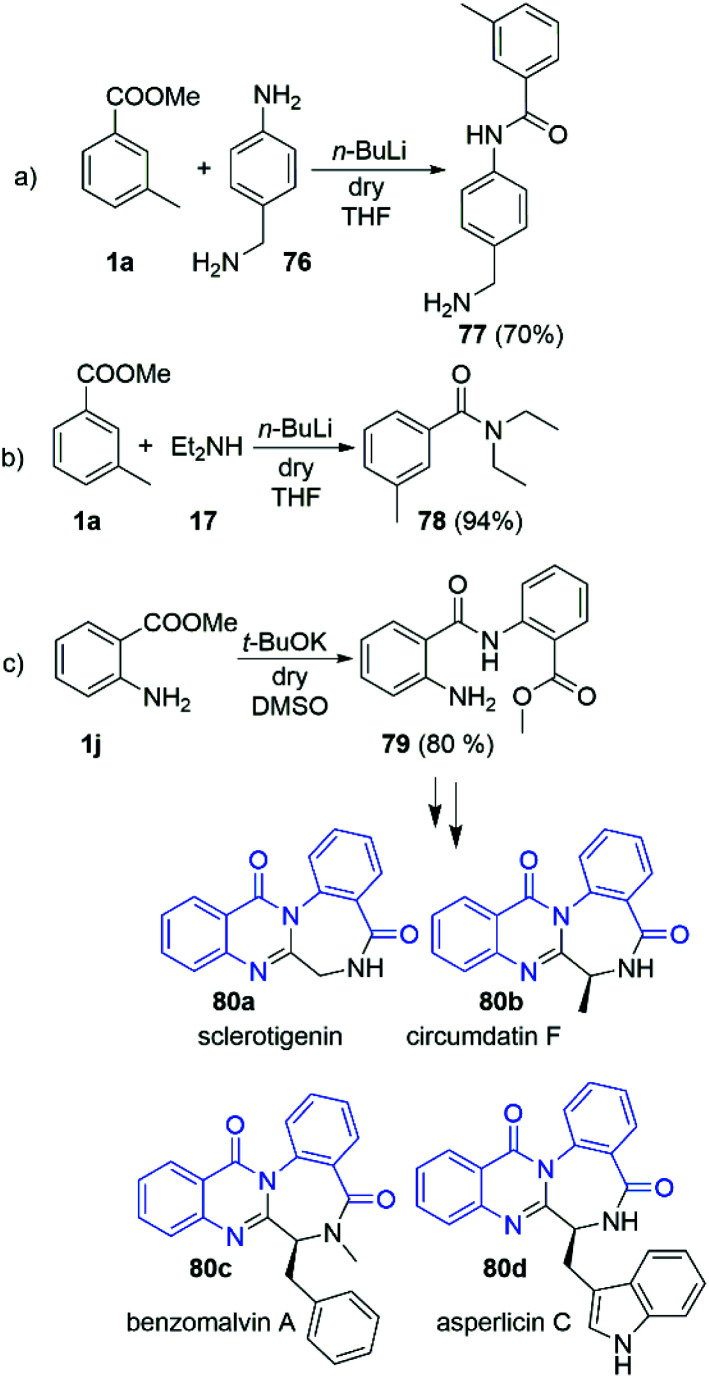
Practical implementations of the amidation reactions. Reaction conditions for (a) and (b): 1.0 eq. of the corresponding amine and 2.0 eq. ester; THF as solvent, and *n*-BuLi (2.0 eq.) as a base (at r.t. in inert atmosphere). Reaction conditions for (c): 1.0 eq. of methyl anthranilate was dissolved in dry DMSO, and then *t*-BuOK (2.5 eq.) was added slowly portionwise. Reaction time 5 min.

Having in hands this large amount of experimental data, we focused our attention towards some practical applications. Driven by the interesting selectivity of the DMSO/*t*-BuOK system that was found to effectively promote direct acylation of anilines, while not effective for alkyl and benzyl amines we attempted to selectively react 4-amino benzylamine (76) with 3-methyl methylbenzoate (1a) at the aniline nitrogen. Nevertheless, the reaction failed to provide the desired product 77. Surprisingly, when the same reaction was carried out in the presence of *n*-BuLi we observed remarkable selectivity at the aniline position, leading to the formation of 77 as a sole product in 70% yield (Scheme 6a). The structure of 77 was unambiguously confirmed by X-ray crystallography as hydrochloric acid salt, 77·HCl (See section X-ray and ESI†). This protocol represents a new way for protective group free functionalisation of anilines in a presence of competitive amino groups, which up to now has been achieved by protection/deprotection sequence,^30^ or through much more laborious synthetic protocols.^31–33^

We further demonstrated the potential of our findings in a *n*-BuLi promoted synthesis of *N*,*N*-diethyl-*meta*-toluamide (DEET) (78) (Scheme 6b). DEET is among the most common active ingredients in insect repellents. To date, it has been mostly prepared *via* classical amidation of the corresponding acylchloride^34^ and amidation of 3-methyl benzoic acid in presence of coupling agents^35^ Other methods based on transition metal catalysis^36–39^ and Grignard promoted amidation of 3-methyl benzonitrile^40^ could be also found in the literature. Herein, we report its synthesis in nearly quantitative yield (94%) from the readily available methyl 3-methylbenzoate and diethylamine (17) at r.t. for 5 min.

The practical potential of the DMSO/*t*-BuOK system was demonstrated in the “auto-amidation” of anthranylic ester 1j (Scheme 6c). The resulting compound 79 is a precursor in the synthesis of various natural compounds such as the alkaloids sclerotigenin (80a), circumdatin F (80b), benzomalvin A (80c), asperlicin C (80d) and others (Scheme 6). To date 79 was synthetized using a laborious two-step procedure, which includes reflux in H_2_SO_4_/MeOH for 4–5 days.^41,42^ In contrast, our protocol provided 79 in 5 minutes with 80% yield.

**Scheme 7:**
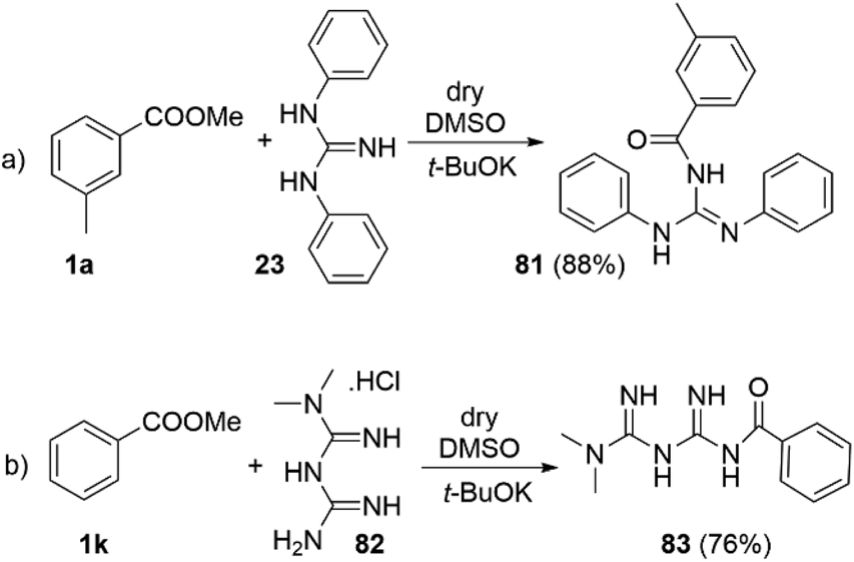
Acylation of guanidines using DMSO/*t*-BuOK. Reaction conditions: 1.0 eq. of amine and 2.0 eq. of ester were dissolved in dry DMSO, and then *t*-BuOK (2.5 eq.) was added slowly portionwise. Reaction time 5 min.

Traditionally the direct acylation of guanidines has been a challenging synthetic task. Therefore, we explored the utility of the DMSO/*t*-BuOK system for direct acylation of *N*,*N*′-diphenylguanidine 23 (Scheme 7a). Although several methods to achieve the synthesis of acylated diphenylguanidines exist, they require laborious synthetic procedures and use of metal catalysts (Pd, Hg, *etc.*).^43–46^ None of them is direct and they mostly exploit substituted thioureas as starting materials. To the best of our knowledge, the direct acylation of diphenylguanidine is being reported here for the first time. Furthermore, the reaction itself is very straightforward, fast, catalyst-free and high yielding. The structure of 81 was confirmed by single crystal X-ray diffraction (See section X-ray and ESI†).

The antiplatelet thrombotic agent 83 (Scheme 7b) was originally synthetized by acylation of *N*,*N*-dimethyl biguanide with benzoic anhydride in acetone at r.t. for 5 hours, with 71% yield.^47^ Our conditions provided its direct synthesis in 76% yield from *N*,*N*-dimethyl biguanide hydrochloride (82) and methyl benzoate at r.t. for 5 min.

### X-ray

Suitable single crystals of key compounds 77·HCl and 81 were prepared, in order to confirm their solid-state structures. Compound 77·HCl was found to crystallise in the *P*2_1_/*n* space group with a single molecule in the asymmetric unit cell. The packing included numerous intermolecular interactions, with the structure being observed as a hydrogen bonded dimer between a pair of (symmetry equivalent) bonds between the amide oxygen atom and the ammonium group (N⋯O = 2.832(4) Å). This network expanded three dimensionally *via* several N–H⋯Cl contacts from both the amide and ammonium groups present, with N⋯Cl distances of 3.544(3) Å and 3.084(3)/3.114(3) Å, respectively. These distances are all within the expected ranges for these types of intermolecular interactions.

**Fig. 2:**
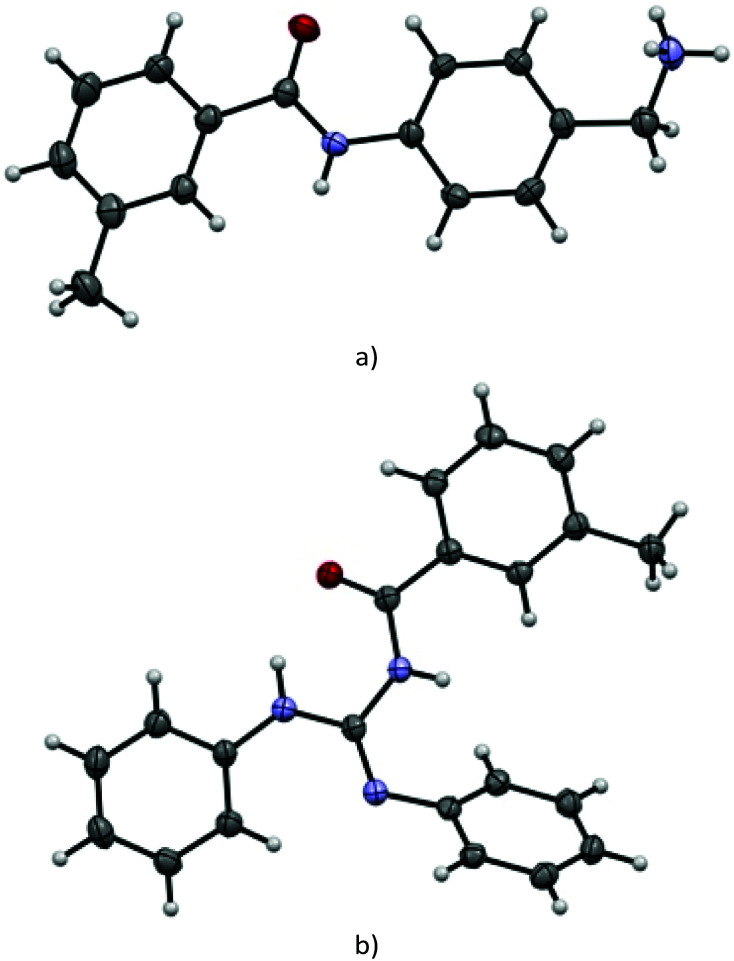
View of the molecular structure obtained by single-crystal X-ray analysis and atom-numbering scheme of compounds (a) 77·HCl (chloride anion omitted for clarity), (b) 81 (all thermal ellipsoids at 50% probability).

The structure of 81 was found to crystallise in the *P*2_1_/*c* space group, again with only a single molecule present in the asymmetric unit cell. Interestingly, despite the potential for hydrogen bonding, only a single intramolecular interaction is present, with all intermolecular contacts consisting of weaker C–H⋯O (C⋯O = 3.314(2) Å) or C–H⋯N (C⋯N = 3.483(2) Å) interactions, or π–π interactions (centroid to centroid distances = 3.88 Å) between the aromatic rings (Fig. 2).

## Conclusions

Herein we report a detailed study of base promoted direct amidation of unactivated esters. A new system (DMSO/*t*-BuOK) was employed and its scope and limitations were investigated. An alternative protocol (THF/*n*-BuLi) was developed in order to overcome its limitations. Our studies encompass a wide range of esters and amines that in full revealed the reaction scope and its limitations. Several practical applications of our findings were proposed. We have achieved straightforward synthesis of several important products, thus paving new ways for their preparation.

## Author contributions

Conceptualization: Svilen Simeonov and Georgi Dobrikov. Experimental: Ivaylo Slavchev and Georgi Dobrikov, drafting: Ivaylo Slavchev and Svilen Simeonov. X-ray: Jas. S. Ward and Kari Rissanen. Review and editing the final manuscript: Svilen Simeonov.

## Conflicts of interest

There are no conflicts to declare.

## Supplementary Material

RA-012-D2RA03524C-s001

RA-012-D2RA03524C-s002
