# Supplementary material for: Base-promoted direct amidation of esters: beyond the current scope and practical applications
Source: RSC Adv. 2022 Jul 15;12(32):20555–62. doi: 10.1039/d2ra03524c (PMC9284525; doi:10.1039/d2ra03524c)
Supplement: RA-012-D2RA03524C-s001 [file RA-012-D2RA03524C-s001.pdf]

Electronic Supplementary Material (ESI) for RSC Advances.
This journal is © The Royal Society of Chemistry 2022

Base-promoted direct amidation of esters: beyond the current scope and practical applications

Ivaylo Slavchev,[a] Jas. S. Ward,[b] Kari Rissanen,[b] Georgi Dobrikov*[a] and Svilen Simeonov*[a,c]

[a]*Institute of Organic Chemistry with Centre of Phytochemistry, Bulgarian Academy of Sciences, Acad. G. Bonchev str., bl. 9, Sofia 1113, Bulgaria*

[b]*University of Jyvaskyla, Department of Chemistry, Survontie 9 B, 40014 Jyväskylä, Finland*

[c]*Research Institute for Medicines (iMed.ULisboa), Faculty of Pharmacy, Universidade de Lisboa, Av. Prof. Gama Pinto, 1649-003 Lisbon, Portugal*

1. General and chemistry

For thin layer chromatography (TLC) aluminum sheets pre-coated with silica gel 60 F_{254} (Merck) were used. Flash column chromatography was carried out using silica gel 60 (0.040–0.063 mm, 230-400 mesh ASTM, Merck). Commercially available solvents for reactions, TLC and column chromatography were used after distillation (and were dried if needed) – water, acetic acid (AcOH), methanol (MeOH), petroleum ether (PE), diethyl ether (Et_2O), ethylacetate (EA), dichloromethane (DCM), chloroform, methyl *tert*-butyl ether (MTBE), diisopropyl ether (DIPE), tetrahydrofurane (THF), dimethylformamide (DMF), *iso*-propanol (*i*-PrOH), dimethylsulfoxyde (DMSO). Any fine chemical, whose synthesis is not described or mentioned in the references in this study, is commercially available from Sigma-Aldrich, Fluorochem, Fluka, Acros or Alfa Aesar. Melting temperatures were determined in capillary tubes on an Electrothermal MEL-TEMP 1102D-230 VAC apparatus without corrections. The NMR spectra were recorded on a Bruker Avance II+ 600 spectrometer (600.13 MHz for ^1H and 150.92 MHz for ^{13}C). In case of $CDCl_3$, TMS was used as internal standard (δ=0.00). For other deuterated solvents ^1H spectra were calibrated to the residual solvent peaks (DMSO-d_6 δ=2.50). ^{13}C spectra were calibrated in all cases to the residual solvent peaks ($CDCl_3$ δ=77.00, DMSO-d_6 δ=39.52). The following additional NMR techniques were used for all compounds: DEPT 135, COSY, HSQC and HMBC. Mass spectra (MS) were recorded in direct regime on Thermo Scientific High Resolution Magnetic Sector MS DFS by chemical ionization (CI), or on a Waters ZQ 2000 by electrospray ionization (ESI). Direct MS-regime for ESI: each compound was dissolved in MeOH with concentration 1 mg/ml; injection volume 3-5 μl; mobile phase – formic acid in MeOH. MS spectra are reported as fragmentation in m/z with relative intensities (%). Optical angle of rotation was measured with JASCO P-2000 apparatus. The compound names are in agreement with the IUPAC nomenclature.

General procedure

Method A (DMSO/*t*-BuOK system). 2 mmol of the respective amine were dissolved in 4 ml dry DMSO, together with 4 mmol of the respective ester at r.t, and were stirred vigorously. 5 mmol of *t*-BuOK were added slowly, portion wise to the mixture. After stirring for 5 min. 50 ml of DCM were added to the mixture and the organic layer was washed with brine 3x100 ml and with water 1x100 ml. After that the organic layer was dried over K_2CO_3 or Na_2SO_4 and the solvent was evaporated under reduced pressure. The crude product was later purified by column chromatography.

Method B (THF/*n*-BuLi system). 2 mmol of the respective amine were dissolved in 10 ml dry THF under argon at r.t. The solution was cooled on an ice bath, and 4 mmol of *n*-BuLi were added to it. The cooling was removed and the mixture was stirred at r.t. for 5 min. Then 4 mmol of the respective ester were added and the mixture was stirred for 5 more minutes. The mixture was concentrated under reduced pressure and 50 ml of DCM were added to it. It was washed with water 1x100 ml. the organic layer was dried over K_2CO_3 or Na_2SO_4 and the solvent was evaporated under reduced pressure. The crude product was later purified by column chromatography.

ANALYTICAL AND SPECTRAL DATA

3-methyl-*N*-(*p*-tolyl)benzamide (**3**) [1]:

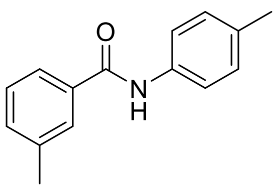

Pale yellow solid, 94% yield (Method A). TLC: DCM/PE 2:1 2x. Column chromatography: DCM/PE 2:1. ^{1}H NMR (600 MHz, $CDCl_3$) δ (ppm) = 8.06 (*s*, 1H), 7.65 – 7.62 (*m*, 1H), 7.60 (*dt*, *J* = 6.6, 2.1 Hz, 1H), 7.53 – 7.49 (*m*, 2H), 7.31 – 7.25 (*m*, 2H), 7.11 (*dd*, *J* = 7.4, 1.5 Hz, 2H), 2.35 (*s*, 3H), 2.31 (*s*, 3H). ^{13}C NMR (151 MHz, $CDCl_3$) δ (ppm) = 166.0, 138.4, 135.4, 134.9, 134.0, 132.3, 129.4, 129.4, 129.4, 128.4, 127.8, 123.9, 120.3, 21.3, 20.8.

- 3-methyl-*N*-(pyridin-2-yl)benzamide (**24**) [2]:

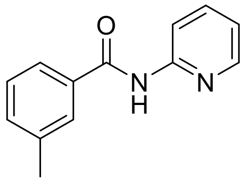

White solid, 89% yield. (Method A). TLC: DCM/MTBE 20:1, 2x. Column chromatography: DCM-DCM/MTBE 10:1. ^{1}H NMR (600 MHz, $CDCl_3$) δ (ppm) = 9.12 (*s*, 1H), 8.41 (*dt*, *J* = 8.4, 1.0 Hz, 1H), 8.15 (*ddd*, *J* = 4.9, 1.9, 0.9 Hz, 1H), 7.77 – 7.68 (*m*, 3H), 7.38 – 7.32 (*m*, 2H), 7.03 (*ddd*, *J* = 7.3, 4.9, 1.0 Hz, 1H), 2.39 (*s*, 3H). ^{13}C NMR (150 MHz, $CDCl_3$) δ (ppm) = 166.1, 151.7, 147.8, 138.6, 138.4, 134.2, 132.9, 128.6, 127.9, 124.3, 119.7, 114.2, 21.3.

N-(3,5-dibromopyridin-2-yl)-3-methylbenzamide (**25**):

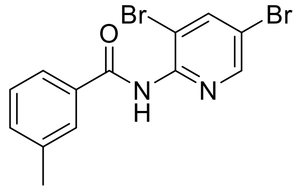

White solid, 72% yield. (Method A). M.p. 148-149ºC. TLC: DCM/MTBE 20:1, 1x. Column chromatography: DCM/MTBE 50:1. ^{1}H NMR (600 MHz, $CDCl_3$) δ (ppm) = 8.49 (*d*, *J* = 2.2 Hz, 1H), 8.46 (*s*, 1H), 8.06 (*d*, *J* = 2.2 Hz, 1H), 7.75 (*d*, *J* = 2.1 Hz, 1H), 7.71 (*dt*, *J* = 6.6, 2.1 Hz, 1H), 7.43 – 7.36 (*m*, 2H), 2.44 (*s*, 3H). ^{13}C NMR (151 MHz, $CDCl_3$) δ (ppm) = 165.1, 148.5, 147.7, 143.3, 139.0, 133.9, 133.5, 128.9, 128.4, 124.5, 115.7, 112.8, 21.5. MS (CI+) m/z (rel. int.): 289 (M-Br, 33), 291 (M-Br, 34), 369 (M+1, 52), 371 (M+1, 100).

N-(3-cyanophenyl)-3-methylbenzamide (**26**):

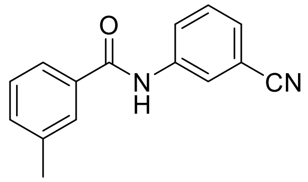

Pale yellow solid, 94% yield. (Method A). M.p. 119-120ºC. TLC: DCM, 1x. Column chromatography: DCM. ^{1}H NMR (600 MHz, DMSO-d^{6}) δ (ppm) = 10.52 (*s*, 1H), 8.28 – 8.24 (*m*, 1H), 8.05 (*dt*, *J* = 7.4, 2.2 Hz, 1H), 7.79 – 7.72 (*m*, 2H), 7.60 – 7.53 (*m*, 2H), 7.46 – 7.40 (*m*, 2H), 2.40 (*s*, 3H). ^{13}C NMR (151 MHz, DMSO-d^{6}) δ (ppm) = 166.1, 140.1, 137.9, 134.4, 132.6, 130.1, 128.4, 128.2, 127.1, 125.0, 124.8, 122.9, 118.8, 111.5, 40.1, 21.0. MS (ESI+) m/z (rel. int.): 237 (M+1, 100), 259 (M+Na, 34), 282 (M+2Na, 43).

3-methyl-*N*-(2,3,5,6-tetrafluorophenyl)benzamide (**27**):

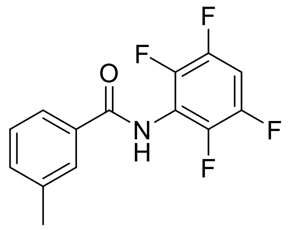

White solid, 75% yield. (Method A). M.p. 146-147ºC. TLC: DCM/PE 1:1, 1x. Column chromatography: DCM/PE 5:1 - 1:1. ^{1}H NMR (400 MHz, $CDCl_3$) δ (ppm) = 7.76 – 7.64 (*m*, 2H), 7.62 (*s*, 1H), 7.45 – 7.33 (*m*, 2H), 7.01 (*tt*, *J* = 9.8, 7.2 Hz, 1H), 2.42 (*s*, 3H). ^{13}C NMR (101 MHz, $CDCl_3$, C-F-decoupling) δ (ppm) = 165.7, 146.2, 142.3, 139.1, 133.7, 132.7, 128.9, 128.5, 124.8, 117.4, 103.9, 21.5. MS (CI+) m/z (rel. int.): 284 (M+1, 100).

3-methyl-*N*-(perfluorophenyl)benzamide (**28**):

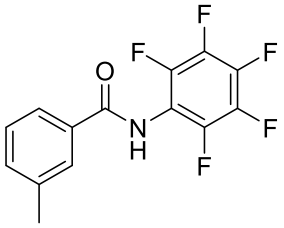

White solid, 78% yield. (Method A). M.p. 144-145ºC. TLC: DCM/PE 1:1, 1x. Column chromatography: DCM/PE 5:1 - 1:1. ^{1}H NMR (400 MHz, $CDCl_3$) δ (ppm) =7.76 – 7.66 (*m*, 2H), 7.66 (*s*, 1H), 7.45 – 7.35 (*m*, 2H), 2.44 (*s*, 3H). ^{13}C NMR (101 MHz, $CDCl_3$, C-F Decoupling) δ (ppm) = 166.1, 139.1, 133.7, 132.5, 128.9, 128.5, 124.8, 21.4. MS (CI+) m/z (rel. int.): 302 (M+1, 100).

3-methyl-*N*-(pyrimidin-2-yl)benzamide (**29**):

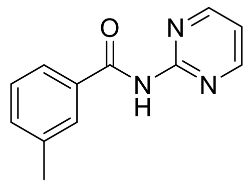

White solid, 94% yield. (Method A). M.p. 154-155ºC. TLC: DCM/MTBE 20:1, 2x. Column chromatography: DCM - DCM/MTBE 10:1. ^{1}H NMR (600 MHz, DMSO-d^{6}) δ (ppm) = 10.95 (*s*, 1H), 8.73 (*d*, *J* = 4.8 Hz, 2H), 7.80 (*d*, *J* = 2.2 Hz, 1H), 7.76 (*dt, J* = 7.1, 2.0 Hz, 1H), 7.43 – 7.36 (*m*, 2H), 7.25 (*t*, *J* = 4.8 Hz, 1H), 2.37 (*s*, 3H). ^{13}C NMR (151 MHz, DMSO-d^{6}) δ (ppm) = 165.61, 158.40, 158.25, 137.70, 134.23, 132.68, 128.79, 128.28, 125.38, 117.32, 40.06, 20.93. MS (ESI+) m/z (rel. int.): 214 (M+1, 100), 236 (M+Na, 57), 449 (2M+Na, 26).

N-(9,10-dioxo-9,10-dihydroanthracen-1-yl)-3-methylbenzamide (**30**):

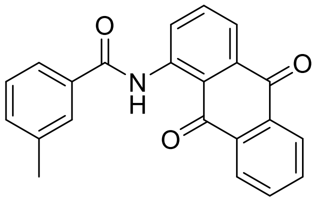

Yellow-orange solid, 55% yield. (Method A). M.p. 198-199ºC. TLC: PE/MTBE 10:1, 3x. Column chromatography: PE/MTBE 10:1. ^{1}H NMR (600 MHz, $CDCl_3$) δ (ppm) = 13.22 (*s*, 1H), 9.33 (*dd*, *J* = 8.5, 1.2 Hz, 1H), 8.36 – 8.30 (*m*, 1H), 8.30 – 8.24 (*m*, 1H), 8.07 (*dd*, *J* = 7.6, 1.2 Hz, 1H), 7.95 (*d*, *J* = 7.1 Hz, 2H), 7.80 (*qd*, *J* = 7.5, 3.8 Hz, 3H), 7.47 (*td*, *J* = 7.2, 1.5 Hz, 1H), 7.42 (*d*, *J* = 7.5 Hz, 1H), 2.50 (*s*, 3H). ^{13}C NMR (151 MHz, $CDCl_3$) δ (ppm) = 187.6, 182.8, 166.9, 142.6, 138.9, 136.0, 134.6, 134.5, 134.4, 134.1, 133.3, 132.9, 128.9, 128.7, 127.6, 127.2, 126.4, 124.7, 122.7, 118.0, 29.8, 21.7. MS (CI+) m/z (rel. int.): 342 (M+1, 100).

3-methyl-*N*-(1,3,4-thiadiazol-2-yl)benzamide (**31**) [3]:

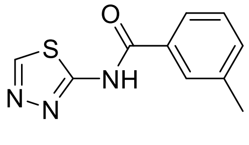

White solid (Method A - 30% yield; Method B – 72% yield). TLC: DCM/MTBE 10:1, 1x. Column chromatography: DCM/MTBE 20:1. ^{1}H NMR (400 MHz, DMSO-d^{6}) δ (ppm) = 13.01 (s, 1H), 9.23 (s, 1H), 7.98 – 7.88 (m, 2H), 7.50 – 7.39 (m, 2H), 2.39 (s, 3H). ^{13}C NMR (101 MHz, DMSO-d^{6}) δ (ppm) = 165.3, 159.5, 149.0, 138.0, 133.5, 131.5, 128.9, 128.5, 125.5, 20.9.

N-((4-carbamoylphenyl)(imino)methyl)-3-methylbenzamide (**32**):

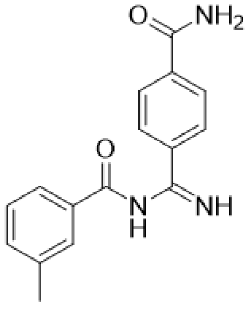

White solid, 69% yield. (Method A). M.p. 191-192ºC. TLC: DCM/MTBE 10:1, 1x. Column chromatography: DCM/MTBE 10:1. ^{1}H NMR (600 MHz, DMSO-d^{6}) δ (ppm) = 10.40 (*s*, 1H), 9.40 (*s*, 1H), 8.25 – 8.21 (m, 2H), 8.15 (*s*, 1H), 8.06 (*dd*, *J* = 8.8, 3.2 Hz, 2H), 8.04 – 7.99 (*m*, 2H), 7.57 (*s*, 1H), 7.41 – 7.36 (*m*, 2H), 2.39 (*s*, 3H). ^{13}C NMR (151 MHz, DMSO-d^{6}) δ (ppm) = 178.6, 167.2, 165.3, 137.7, 137.4, 137.0, 132.6, 129.6, 128.1, 127.94, 127.6, 126.5, 40.1, 21.1. MS (ESI+) m/z (rel. int.): 119 (3-methylbenzoyl, 55), 282 (M+1, 100).

N-(*N*,*N*-dimethylcarbamimidoyl)-3-methylbenzamide (**33**):

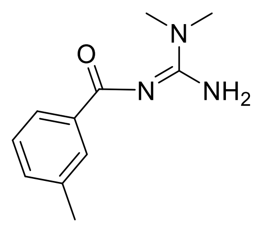

White solid, 60% yield. (Method A) M.p. 180-181ºC. TLC: DCM/MTBE 1:1, 1x. Column chromatography: DCM/MTBE 2:1. ^{1}H NMR (600 MHz, DMSO-d^{6}) δ (ppm) = 8.39 (s, 2H), 7.96 – 7.85 (m, 2H), 7.28 – 7.20 (m, 2H), 2.35 (s, 3H). ^{13}C NMR (151 MHz, DMSO-d^{6}) δ (ppm) = 174.5, 160.8, 139.2, 136.1, 130.5, 128.6, 127.0, 125.3, 36.0, 20.5. MS (ESI+) m/z (rel. int.): 119 (3-methylbenzoyl, 55), 206 (M+1, 100).

N-(1*H*-benzo[*d*]imidazol-2-yl)-3-methylbenzamide (**34**) [4]:

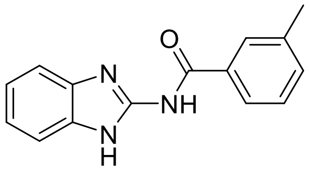

White solid, 62% yield. (Method A) TLC: DCM/MTBE 5:1, 1x. Column chromatography: DCM/MTBE10:1. ^{1}H NMR (400 MHz, DMSO-d^{6}) δ (ppm) = 11.98 (s, 2H), 7.98 (tt, *J* = 1.6, 0.9 Hz, 1H), 7.94 (tdd, *J* = 4.4, 1.8, 0.7 Hz, 1H), 7.52 – 7.43 (m, 2H), 7.42 – 7.38 (m, 2H), 7.17 – 7.09 (m, 2H), 2.40 (d, *J* = 0.7 Hz, 3H). ^{13}C NMR (101 MHz, DMSO-d^{6}) δ (ppm) = 168.2, 148.6, 137.2, 134.3, 134.1, 132.1, 128.4, 127.7, 125.0, 121.0, 113.0, 20.5.

Piperidin-1-yl(*m*-tolyl)methanone (**35**) [5]:

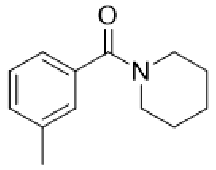

Pale yellow oil. (Method A - 71% yield; Method B – 85% yield). TLC: DCM/MTBE 5:1, 1x. Column chromatography: DCM/MTBE 15:1 - 10:1. ^{1}H NMR (600 MHz, $CDCl_3$) δ (ppm) = 7.31 – 7.24 (*m*, 1H), 7.22 – 7.18 (*m*, 2H), 7.18 – 7.14 (*m*, 1H), 3.71 (*s*, 2H), 3.34 (*s*, 2H), 2.37 (*d*, *J* = 0.8 Hz, 2H), 1.68 (*dd*, *J* = 7.5, 3.8 Hz, 4H). ^{13}C NMR (151 MHz, $CDCl_3$) δ (ppm) = 170.5, 138.2, 136.4, 130.0, 128.2, 127.4, 123.6, 48.7, 43.0, 26.5, 25.6, 24.6, 21.4.

2-methoxy-*N*-(pyrimidin-2-yl)benzamide (**36**):

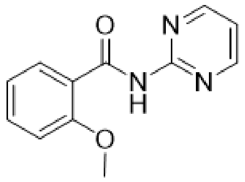

White solid, 63% yield. (Method A). M.p. 102-103ºC. TLC: DCM/MTBE 5:1, 1x. Column chromatography: DCM/MTBE 10:1. ^{1}H NMR (600 MHz, $CDCl_3$) δ (ppm) = 10.54 (*s*, 1H), 8.68 (*d*, *J* = 4.8 Hz, 2H), 8.33 (*dd*, *J* = 7.8, 1.9 Hz, 1H), 7.52 (*ddd*, *J* = 8.8, 7.3, 1.9 Hz, 1H), 7.17 – 7.11 (*m*, 1H), 7.07 – 7.01 (*m*, 2H), 4.09 (*s*, 3H). ^{13}C NMR (151 MHz, $CDCl_3$) δ (ppm) = 162.8, 158.5, 158.1, 157.4, 1334.0, 133.2, 121.8, 121.6, 116.7, 111.6, 56.4. MS (CI+) m/z (rel. int.): 135 (2-methoxybenzoyl, 100), 198 (M-OMe, 52), 230 (M+1, 92).

N-(bis(phenylamino)methylene)-2-methoxybenzamide (**37**):

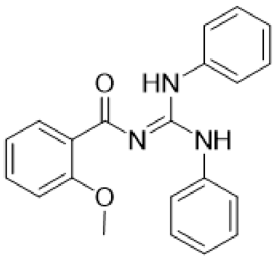

Pale pink solid, 85% yield. (Method A). M.p. 137-138ºC. TLC: DCM/MTBE 5:1, 2x. Column chromatography: DCM/MTBE 5:1. ^{1}HNMR (600 MHz, $CDCl_3$) δ (ppm) = 10.69 (*s*, 1H), 10.14 (*s*, 1H), 8.21 (*dd*, *J* = 7.9, 1.8 Hz, 1H), 7.78 (*d*, *J* = 8.0 Hz, 2H), 7.48 (*ddd*, *J* = 8.7, 7.3, 1.9 Hz, 1H), 7.37 (*t*, *J* = 7.7 Hz, 2H), 7.32 (*t*, *J* = 7.7 Hz, 2H), 7.13 – 7.06 (*m*, 2H), 7.04 (*d*, *J* = 7.4 Hz, 1H), 7.00 (*d*, J=7.5 Hz, 2H), 6.83 (*d*, *J* = 8.4 Hz, 1H), 3.26 (*s*, 3H). ^{13}C NMR (151 MHz, $CDCl_3$) δ (ppm) = 165.7, 157.8, 147.9, 141.6, 139.5, 134.8, 132.6, 129.7, 128.9, 123.1, 122.8, 122.7, 121.6, 120.1, 119.5, 111.52, 55.6. MS (CI+) m/z (rel. int.): 135 (2-methoxybenzoyl, 72), 314 (M-OMe, 66), 346 (M+1, 100).

2-phenyl-*N*-(pyrimidin-2-yl)acetamide (**38**) [6]:

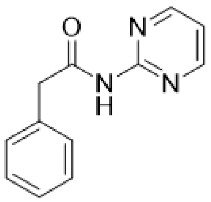

White solid, 58% yield. (Method A). TLC: DCM/MTBE 10:1, 2x. Column chromatography: DCM/MTBE 10:1. ^{1}H NMR (600 MHz, $CDCl_3$) δ (ppm) = 8.74 (s, 1H), 8.53 (d, *J* = 4.8 Hz, 2H), 7.38 – 7.33 (m, 4H), 7.30 (m, 1H), 6.91 (t, *J* = 4.9 Hz, 1H), 3.97 (s, 2H). ^{13}C NMR (151 MHz, $CDCl_3$) δ (ppm) = 158.48, 157.53, 134.29, 129.72, 129.06, 127.53, 116.62, 44.67.

N-(*p*-tolyl)acetamide (**39**) [7]:

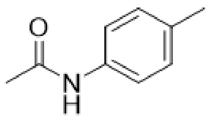

White solid. (Method A - 65% yield; Method B - 83% yield). TLC: DCM:MTBE 5:1, 1x. Column chromatography: DCM/MTBE 10:1. ^{1}H NMR (600 MHz, $CDCl_3$) δ (ppm) = 7.42 (*s*, 1H), 7.40 – 7.34 (*m*, 2H), 7.13 – 7.08 (*m*, 2H), 2.30 (*s*, 3H), 2.15 (*s*, 3H). ^{13}C NMR (151 MHz, $CDCl_3$) δ (ppm) = 168.3, 135.3, 133.9, 129.4, 120.0, 24.5, 20.8.

N-(2-methoxybenzyl)-3-methylbenzamide (**48**) [6]:

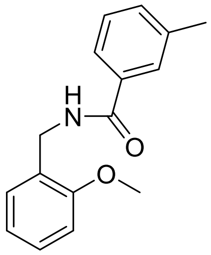

White solid, 78% yield. (Method B). TLC: DCM, 1x. Column chromatography: DCM. ^{1}H NMR (600 MHz, $CDCl_3$) δ 7.59 (tt, *J* = 1.9, 0.9 Hz, 1H), 7.55 – 7.50 (m, 1H), 7.34 (dd, *J* = 7.4, 1.7 Hz, 1H), 7.30 – 7.24 (m, 3H), 6.93 (td, *J* = 7.4, 1.1 Hz, 1H), 6.89 (dd, *J* = 8.2, 1.1 Hz, 1H), 6.67 (s, 1H), 4.63 (d, *J* = 5.8 Hz, 2H), 3.87 (s, 3H), 2.37 (s, 3H). ^{13}C NMR (151 MHz, $CDCl_3$) δ 167.5, 157.7, 138.4, 134.9, 132.1, 130.0, 129.0, 128.5, 128.5, 127.9, 126.3, 123.9, 120.9, 110.5, 55.5, 40.0, 21.5.

N-(adamantan-1-yl)-3-methylbenzamide (**49**):

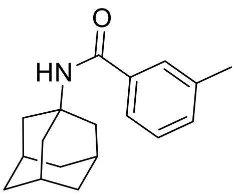

White solid, 70% yield. (Method B). M.p. 139-140ºC. TLC: PE/DCM 1:2, 2x. Column chromatography: PE/DCM 1:2. ^{1}H NMR (600 MHz, $CDCl_3$) δ 7.53 (d, *J* = 2.2 Hz, 1H), 7.48 (dt, *J* = 7.1, 2.0 Hz, 1H), 7.32 – 7.22 (m, 2H), 5.80 (s, 1H), 2.38 (s, 3H), 2.12 (s, 9H), 1.78 – 1.65 (m, 6H). ^{13}C NMR (151 MHz, $CDCl_3$) δ 166.9, 138.4, 136.1, 131.8, 128.4, 127.6, 123.8, 52.3, 41.8, 36.5, 29.6, 21.5. MS (CI+) m/z (rel. int.): 135 (59, 2-methoxybenzoyl), 270 (M+1, 100).

N,*N*-dibutyl-3-methylbenzamide (**50**) [8]:

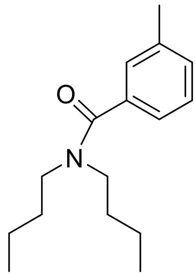

Colorless oil, 89% yield. (Method B). TLC: DCM/MTBE 10:1 1x. Column chromatography: DCM-DCM/MTBE 50:1. ^{1}H NMR (600 MHz, $CDCl_3$) δ (ppm) = 7.39 – 7.33 (*m*, 1H), 7.30 – 7.27 (*m*, 1H), 7.26 (*d*, *J* = 2.0 Hz, 1H), 7.25 – 7.21 (*m*, 1H), 3.58 (*t*, *J* = 7.7 Hz, 2H), 3.28 (*t*, *J* = 7.6 Hz, 2H), 2.47 (*s*, 3H), 1.81 – 1.67 (*m*, 2H), 1.59 (*p*, *J* = 7.6 Hz, 2H), 1.50 (*q*, *J* = 7.6 Hz, 2H), 1.24 (*q*, *J* = 7.3 Hz, 2H), 1.08 (*t*, *J* = 7.3 Hz, 3H), 0.89 (*t*, *J* = 7.5 Hz, 3H). ^{13}C NMR (151 MHz, $CDCl_3$) δ (ppm) = 171.9, 138.2, 137.5, 129.8, 128.3, 127.2, 123.5, 48.8, 44.5, 30.9, 29.8, 21.5, 20.4, 19.9, 14.1, 13.8.

N-ethyl-3-methyl-*N*-phenylbenzamide (**51**):

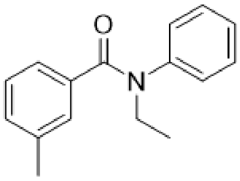

Colorless oil, 80% yield. (Method B). TLC: DCM, 1x. Column chromatography: DCM/PE 1:1 – DCM. ^{1}H NMR (600 MHz, $CDCl_3$) δ (ppm) = 7.25 – 7.18 (*m*, 3H), 7.17 – 7.11 (*m*, 1H), 7.05 – 6.97 (*m*, 5H), 3.98 (*q*, *J* = 7.1 Hz, 2H), 2.22 (*s*, 3H), 1.22 (*t*, *J* = 7.1 Hz, 3H). ^{13}C NMR (151 MHz, $CDCl_3$) δ (ppm) = 170.5, 143.4, 137.6, 136.3, 130.3, 129.5, 129.1, 128.0, 127.5, 126.7, 125.8, 45.5, 21.3, 13.1. MS (CI+) m/z (rel. int.): 240 (M+1, 100).

<u>*N*-(bis(dimethylamino)methylene)-3-methylbenzamide (**52**):</u>

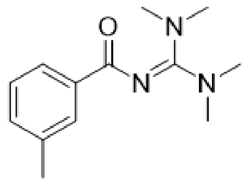

Colorless oil, 98% yield. (Method B). TLC: DCM/MTBE 1:1, 1x. Column chromatography: DCM/MTBE 1:1. ^{1}H NMR (600 MHz, $CDCl_3$) δ (ppm) = 7.97 – 7.91 (*m*, 2H), 7.33 – 7.22 (*m*, 2H), 2.94 (*s*, 12H), 2.39 (*s*, 3H). ^{13}C NMR (151 MHz, $CDCl_3$) δ (ppm) = 173.4, 168.5, 138.0, 137.4, 131.5, 129.7, 127.8, 126.3, 40.2, 21.4. MS (CI+) m/z (rel. int.): 119 (3-methylbenzoyl, 100) 234 (M+1, 64).

<u>(4-benzhydrylpiperazin-1-yl)(m-tolyl)methanone (**53**):</u>

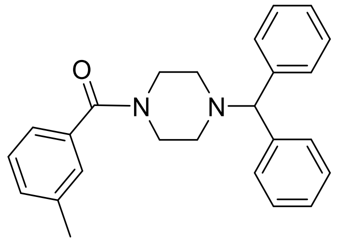

White solid, 70% yield. (Method B). M.p. 123-124ºC. TLC: DCM/MTBE 10:1 1x. Column chromatography: DCM - DCM/MTBE 20:1. ^{1}H NMR (400 MHz, DMSO-d^6) δ (ppm) = 7.45 – 7.40 (m, 4H), 7.33 – 7.25 (m, 5H), 7.24 – 7.11 (m, 5H), 4.40 (s, 1H), 3.52 (s, 4H), 2.39 – 2.33 (m, 4H), 2.32 (s, 3H). ^{13}C NMR (101 MHz, DMSO-d^6) δ (ppm) = 168.6, 141.9, 137.3, 135.6, 129.5, 128.0, 127.7, 127.3, 126.9, 126.5, 123.5, 74.3, 51.0, 38.9, 20.4. MS (CI+) m/z (rel. int.): 167 ($CH(Ph)_2$, 100), 208 (93), 369 (M+1, 16).

<u>(2,6-dimethylpiperidin-1-yl)(m-tolyl)methanone (**54**):</u>

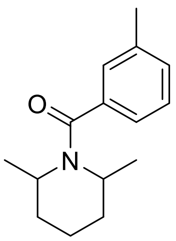

Colorless solid, 63% yield. (Method B). M.p. 78-79ºC. TLC: DCM 1x. Column chromatography: DCM. ^{1}H NMR (600 MHz, DMSO-d^6) δ (ppm) = 7.29 (t, *J* = 7.5 Hz, 1H), 7.23 – 7.19 (m, 1H), 7.17 – 7.06 (m, 2H), 4.31 (t, *J* = 7.2 Hz, 2H), 2.34 (s, 3H), 1.84 (qt, *J* = 12.9, 3.6 Hz, 1H), 1.63 (tdd, *J* = 12.9, 6.1, 4.0 Hz, 2H), 1.57 – 1.44 (m, 3H), 1.22 (d, *J* = 7.1 Hz, 6H). ^{13}C NMR (151 MHz, DMSO-d^6) δ (ppm) = 169.8, 137.6, 137.3, 128.7, 127.7, 125.8, 122.3, 45.7, 29.3, 20.5, 20.4, 13.5. MS (CI+) m/z (rel. int.): 232 (M+1, 100).

<u>*N*-(2,6-diisopropylphenyl)-3-methylbenzamide (**55**):</u>

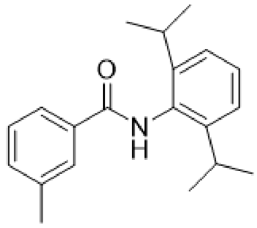

White solid, 70% yield. (Method B). M.p.= 207-208ºC. TLC: DCM/PE 1:1, 1x. Column chromatography: DCM/PE 1:1 – DCM. ^{1}H NMR (600 MHz, DMSO-d^6) δ (ppm) = 9.81 (s, 1H), 7.93 – 7.89 (m, 2H), 7.55 – 7.48 (m, 2H), 7.40 (t, *J* = 7.7 Hz, 1H), 7.30 (d, *J* = 7.7 Hz, 2H), 3.18 (*hept*, *J* = 6.9 Hz, 2H), 2.50 (*s*, 3H), 1.27 (*d*, *J* = 6.9 Hz, 6H), 1.20 (*d*, *J* = 6.9 Hz, 6H). ^{13}C NMR (151 MHz, DMSO-d^6) δ (ppm) = 166.3, 146.2, 137.8, 134.4, 132.9, 132.1, 128.4, 128.1, 127.6, 124.6, 122.9, 40.1, 39.9, 28.2, 23.6, 23.3, 21.0. MS (ESI+) m/z (rel. int.): 206 (88), 296 (M+1, 100), 318 (M+Na, 79).

<u>Morpholino(*m*-tolyl)methanone (**56**) [9]:</u>

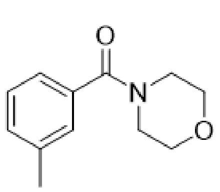

Colorless oil, 75% yield. (Method B). TLC: DCM/MTBE 5:1, 1x. Column chromatography: DCM - DCM/MTBE 5:1. ^{1}H NMR (600 MHz, $CDCl_3$) δ (ppm) = 7.32 – 7.26 (*m*, 1H), 7.28 – 7.21 (*m*, 2H), 7.20 – 7.15 (*m*, 1H), 3.78 (*s*, 4H), 3.65 – 3.60 (*m*, 2H), 3.45 (s, 2H), 2.37 (*s*, 3H). ^{13}C NMR (151 MHz, $CDCl_3$) δ (ppm) = 170.6, 138.4, 135.1, 130.5, 128.3, 127.6, 123.9, 66.8, 48.1, 42.4, 21.3.

(*S*)-*N*-(1-hydroxybutan-2-yl)-3-methylbenzamide (**57**):

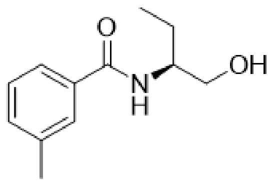

Pale yellow solid, 64% yield. (Method B). M.p. 93-94ºC. $[\alpha]_D^{20}$ = +33.1 (*c*=1.0, $CHCl_3$) TLC: DCM/MTBE 5:1, 1x. Column chromatography: DCM/MTBE 10:1. ^{1}H NMR (600 MHz, $CDCl_3$) δ (ppm) = 7.57 (*tt*, *J* = 1.6, 0.8 Hz, 1H), 7.57 – 7.50 (*m*, 1H), 7.31 – 7.23 (*m*, 2H), 6.54 (*d*, *J* = 8.0 Hz, 1H), 4.04 (*tddd*, *J* = 8.2, 6.3, 5.2, 3.5 Hz, 1H), 3.75 (*dd*, *J* = 11.2, 3.6 Hz, 1H), 3.68 (*dd*, *J* = 11.1, 5.2 Hz, 1H), 3.42 (*s*, 1H), 2.35 (*s*, 3H), 1.75 – 1.56 (*m*, 2H), 0.99 (*t*, *J* = 7.5 Hz, 3H). ^{13}C NMR (151 MHz, $CDCl_3$) δ 168.6, 138.5, 134.5, 132.4, 128.5, 127.8, 124.1, 65.1, 53.7, 53.7, 24.4, 21.4, 10.8. MS (CI+) m/z (rel. int.): 119 (3-methylbenzoyl, 100), 208 (M+1, 45).

3-methyl-*N*-(*N*-(*N*-phenylcarbamimidoyl)carbamimidoyl)benzamide (**58**):

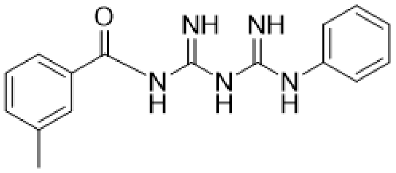

White solid, 76% yield. (Method B). M.p. 79-80ºC. TLC: DCM/MTBE 10:1, 1x. Column chromatography: DCM - DCM/MTBE 10:1. ^{1}H NMR (600 MHz, $CDCl_3$) δ (ppm) = 8.16 – 8.11 (m, 2H), 7.65 (s, 1H), 7.57 (d, *J* = 7.9 Hz, 2H), 7.36 – 7.29 (m, 2H), 7.32 – 7.27 (m, 2H), 7.09 – 7.03 (m, 1H), 5.67 (s, 2H), 2.40 (s, 3H). ^{13}C NMR (151 MHz, $CDCl_3$) δ (ppm) = 172.4, 167.5, 165.0, 138.5, 138.1, 136.5, 132.6, 129.0, 128.9, 128.4, 125.7, 123.6, 120.8, 53.6, 21.6. MS (CI+) m/z (rel. int.): 278 (M-OH, 100).

N-(4-(3-hydroxypropyl)phenyl)-3-methylbenzamide (**59**):

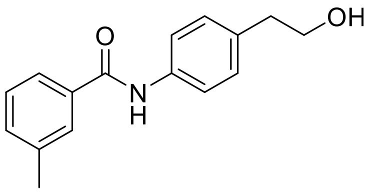

Pale brown solid, 42% yield. (Method B). M.p. 110-111ºC. TLC: DCM/MTBE 10:1, 1x. Column chromatography: DCM - DCM/MTBE 20:1. ^{1}H NMR (600 MHz, DMSO-d^6) δ (ppm) = 10.25 (*s*, 1H), 7.89 – 7.82 (*m*, 2H), 7.80 – 7.75 (*m*, 2H), 7.54 – 7.47 (*m*, 2H), 7.31 – 7.27 (*m*, 2H), 4.76 (*t*, *J* = 5.2 Hz, 1H), 3.70 (*td*, *J* = 7.2, 5.2 Hz, 2H), 2.80 (*t*, *J* = 7.1 Hz, 2H), 2.50 (*s*, 3H). ^{13}C NMR (151 MHz, DMSO-d^6) δ (ppm) = 165.5, 137.7, 137.2, 135.1, 134.8, 132.1, 129.0, 128.3, 128.1, 124.8, 120.3, 62.3, 40.1, 38.6, 21.0. MS (ESI+) m/z (rel. int.): 256 (M+1, 45), 278 (M+Na, 100).

2-(hydroxymethyl)-*N*-(*p*-tolyl)benzamide (**60**):

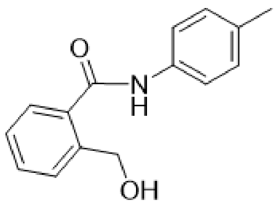

White solid, 81% yield. (Method B). M.p. 169-170ºC. TLC: DCM/MTBE 5:1, 1x. Column chromatography: DCM/MTBE 10:1. ^{1}H NMR (600 MHz, DMSO-d^6) δ (ppm) = 10.29 (*s*, 1H), 7.64 – 7.57 (*m*, 3H), 7.54 – 7.46 (*m*, 2H), 7.37 (*td*, *J* = 7.5, 1.3 Hz, 1H), 7.14 (*d*, *J* = 8.2 Hz, 2H), 5.32 (*t*, *J* = 5.5 Hz, 1H), 4.66 (*d*, *J* = 5.4 Hz, 2H), 2.27 (*s*, 3H). ^{13}C NMR (151 MHz, DMSO-d^6) δ (ppm) = 167.2, 140.3, 136.8, 135.3, 132.5, 129.9, 129.1, 127.5, 127.5, 126.7, 119.8, 60.8, 40.1, 20.5. MS (ESI+) m/z (rel. int.): 224 (M-OH, 55), 242 (M+1, 25), 264 (M+23, 100).

2-hydroxy-*N*-(*p*-tolyl)benzamide (**61**) [10]:

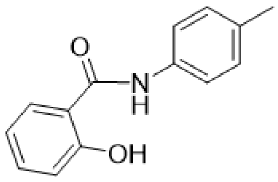

White solid, 81% yield. (Method B). M.p. 169-170ºC. TLC: DCM/MTBE 5:1, 1x. Column chromatography: DCM/MTBE 10:1. ^{1}H NMR (600 MHz, $CDCl_3$) δ (ppm) = 12.06 (*s*, 1H), 7.89 (*s*, 1H), 7.50 (dd, *J* = 8.0, 1.6 Hz, 1H), 7.47 – 7.41 (m, 3H), 7.22 – 7.17 (*m*, 2H), 7.03 (dd, *J* = 8.3, 1.2 Hz, 1H), 6.91 (*ddd*, *J* = 8.2, 7.2, 1.2 Hz, 1H), 2.35 (*s*, 3H). ^{13}C NMR (151 MHz, $CDCl_3$) δ (ppm) = 168.3, 161.8, 135.2, 134.6, 133.9, 129.7, 125.3, 121.3, 118.9, 118.9, 114.5, 20.9.

2-chloro-5-nitro-*N*-(*p*-tolyl)benzamide (**62**) [11]:

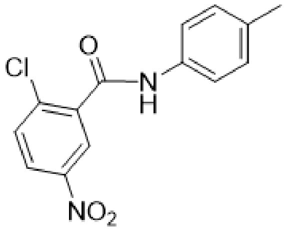

Pale yellow solid, 25% yield. (Method B). TLC: DCM/PE 1:1, 1x. Column chromatography: DCM/PE 1:1. ^{1}H NMR (600 MHz, $CDCl_3$) δ (ppm) = 8.57 (*d*, *J* = 2.7 Hz, 1H), 8.23 (*dd*, *J* = 8.8, 2.7 Hz, 1H), 7.86 (*s*, 1H), 7.63 (*d*, *J* = 8.8 Hz, 1H), 7.52 – 7.47 (*m*, 2H), 7.22 – 7.17 (*m*, 2H), 2.36 (*s*, 3H). ^{13}C NMR (151 MHz, $CDCl_3$) δ (ppm) = 162.0, 146.6, 137.5, 136.6, 135.3, 134.3, 131.5, 129.7, 125.9, 125.3, 120.4, 21.0.

6-oxo-6-(*p*-tolylamino)hexanoic acid (**63**):

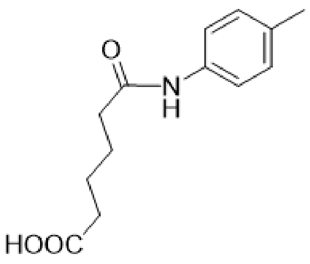

White solid, 63% yield. (Method B). M.p. 164-165ºC. TLC: DCM/*i*PrOH 20:1. Column chromatography: DCM/*i*PrOH 40:1. ^{1}H NMR (600 MHz, DMSO-d^6) δ (ppm) = 12.03 (*s*, 1H), 9.78 (*s*, 1H), 7.48 – 7.44 (*m*, 2H), 7.07 (*d*, *J* = 8.2 Hz, 2H), 2.33 – 2.16 (*m*, 9H), 1.63 – 1.48 (*m*, 2H). ^{13}C NMR (151 MHz, DMSO-d^6) δ (ppm) = 174.4, 170.8, 136.9, 131.8, 129.1, 119.1, 72.2, 40.1, 38.6, 36.1, 33.5, 25.3, 25.0, 24.7, 24.2, 23.0, 20.5, 14.2. MS (CI+) m/z (rel. int.): 107 (4-methylaniline, 32), 218 (M-OH, 100), 235 (M+1, 19).

2-hydroxy-N-(p-tolyl)acetamide (**64**) [12]:

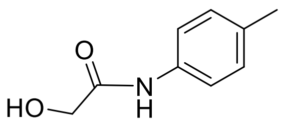

White solid, 53% yield. (Method B). TLC: DCM/MTBE 5:1 1x. Column chromatography: DCM/MTBE 10:1. ^{1}H NMR (600 MHz, CD_3OD) δ (ppm) = 7.45 – 7.48 (m, 2H), 7.12 – 7.15 (m, 2H), 4.09 (s, 2H), 2.30 (s, 3H). ^{13}C NMR (151 MHz, CD_3OD) δ (ppm) = 173.2, 136.3, 135.3, 130.3, 121.6, 62.9, 20.9.

Diethyl phenylphosphoramidate (**70**) [13]:

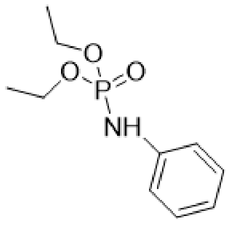

White solid, 81% yield. (Method B). TLC: DCM/MTBE 5:1 1x. Column chromatography: DCM/MTBE 20:1. ^{1}H NMR (600 MHz, $CDCl_3$) δ (ppm) = 7.27 – 7.21 (*m*, 2H), 7.06 – 7.01 (*m*, 2H), 6.94 (*tt*, *J* = 7.4, 1.1 Hz, 1H), 6.89 (*d*, *J* = 9.6 Hz, 1H), 4.18 (*dp*, *J* = 10.1, 7.2 Hz, 2H), 4.09 (*ddq*, *J* = 10.1, 7.9, 7.1 Hz, 2H), 1.31 (*td*, *J* = 7.1, 0.9 Hz, 6H). ^{13}C NMR (151 MHz, $CDCl_3$) δ (ppm) = 139.9, 129.2, 121.3, 117.2 (J_{CP} = 7.4 Hz), 62.6, 62.6, 16.1, 16.0.

Diethyl *p*-tolylphosphoramidate (**71**) [14]:

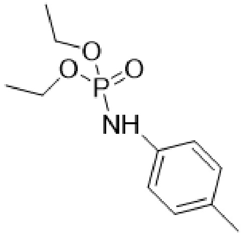

White solid, 87% yield. (Method B). TLC: DCM/MTBE 10:1. Column chromatography: DCM/MTBE 10:1. ^{1}H NMR (600 MHz, $CDCl_3$) δ (ppm) = 7.06 – 7.01 (*m*, 2H), 6.96 – 6.90 (*m*, 2H), 6.61 (d, *J* = 9.6 Hz, 1H), 4.17 (*dp*, *J* = 10.1, 7.2 Hz, 2H), 4.07 (*ddq*, *J* = 10.1, 7.9, 7.1 Hz, 2H), 2.27 (*s*, 3H), 1.30 (*td*, *J* = 7.0, 0.9 Hz, 6H). ^{13}C NMR (151 MHz, $CDCl_3$) δ (ppm) = 137.2, 130.7, 129.7, 117.2 (J_{CP} = 7.1 Hz), 62.5, 62.5, 20.5, 16.1, 16.0.

Diethyl (4-bromophenyl)phosphoramidate (**72**) [15]:

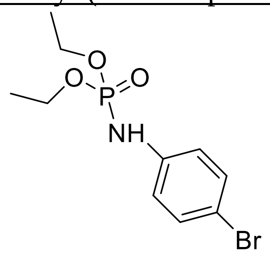

White solid, 84% yield. (Method B). TLC: DCM/MTBE 10:1 2x. Column chromatography: DCM, DCM/MTBE 20:1-10:1. ^{1}H NMR (600 MHz, $CDCl_3$) δ (ppm) = 7.35 – 7.29 (m, 2H), 7.26 (d, *J* = 9.6 Hz, 1H), 6.95 – 6.89 (m, 2H), 4.20 – 4.01 (m, 4H), 1.30 (td, *J* = 7.1, 0.9 Hz, 6H). ^{13}C NMR (151 MHz, $CDCl_3$) δ (ppm) = 139.4, 132.2, 118.8 (J_{CP} = 7.6 Hz), 113.9, 62.77, 62.74, 16.07, 16.02.

Diethyl naphthalen-2-ylphosphoramidate (**73**):

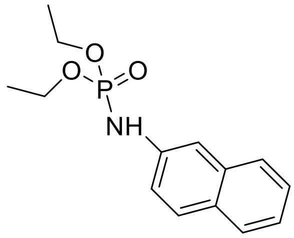

Colorless oil, 78% yield. (Method B). TLC: DCM/MTBE 10:1 2x. Column chromatography: DCM, DCM/MTBE 20:1-10:1. ^{1}H NMR (600 MHz, $CDCl_3$) δ (ppm) = 7.80 – 7.73 (*m*, 3H), 7.49 – 7.43 (*m*, 2H), 7.39 – 7.25 (*m*, 2H), 4.27 (*dp*, *J* = 10.2, 7.2 Hz, 2H), 4.16 (*ddt*, *J* = 15.1, 10.1, 7.1 Hz, 2H), 1.36 (*td*, *J* = 7.1, 0.9 Hz, 6H). ^{13}C NMR (151 MHz, $CDCl_3$) δ (ppm) = 137.8, 134.4, 129.3, 127.7, 126.9, 126.6, 124.0, 119.2, 119.1, 112.4, 112.3, 62.9, 62.9, 16.3, 16.2. MS (ESI+) m/z (rel. int.): 280 (M+1, 100), 302 (M+Na, 46), 581 (2M+Na, 30).

Diethyl-(adamantan-1-yl)phosphoramidate (**74**) [16]:

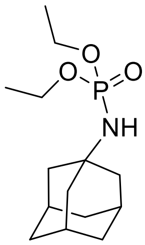

White solid, 81% yield. (Method B). M.p 96-97ºC. TLC: DCM/MTBE 5:1 1x. Column chromatography: DCM/MTBE 10:1. ^{1}H NMR (600 MHz, $CDCl_3$) δ (ppm) = 4.13 – 4.00 (m, 4H), 2.50 (d, *J* = 7.8 Hz, 1H), 2.06 (q, *J* = 3.3 Hz, 3H), 1.81 (d, *J* = 2.9 Hz, 6H), 1.68 – 1.58 (m, 6H), 1.33 (td, *J* = 7.1, 0.8 Hz, 6H). ^{13}C NMR (151 MHz, $CDCl_3$) δ (ppm) =62.2, 62.1, 50.9, 44.33 (J_{CP} = 4.5 Hz), 36.2, 29.8, 16.4, 16.3. MS (CI+) m/z (rel. int.): 135 (adamantane-1, 23), 230 (M-H-2Et, 28), 288 (M+1, 100).

N-(4-(aminomethyl)phenyl)-3-methylbenzamide (**77**):

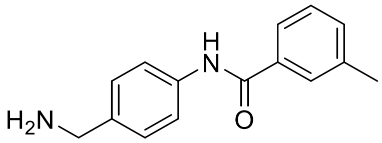

White solid, 70% yield. (Method B). TLC: MTBE/i-PrOH 10:1 1x. Column chromatography: MTBE/i-PrOH 10:1. ^{1}H NMR (600 MHz, DMSO-d^6) δ (ppm) = 10.16 (s, 1H), 7.78 – 7.72 (m, 2H), 7.72 – 7.66 (m, 2H), 7.44 – 7.36 (m, 2H), 7.35 – 7.28 (m, 2H), 3.70 (s, 2H), 2.40 (s, 3H). ^{13}C NMR (151 MHz, DMSO-d^6) δ (ppm) = 165.5, 139.0, 137.7, 137.5, 135.0, 132.2, 128.3, 128.1, 127.3, 124.8, 120.2, 5.1, 40.1, 21.0. MS (CI+) m/z (rel. int.): 224 (M-NH_3, 100), 241 (M+1, 49).

N,*N*-diethyl-3-methylbenzamide (**78**) [17]:

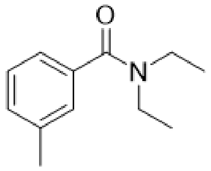

Colorless oil, 94% yield. (Method B). TLC: DCM/MTBE 10:1, 1x. Column chromatography: DCM-DCM/MTBE 20:1. ^{1}H NMR (600 MHz, $CDCl_3$) δ (ppm) = 7.28-7.24 (*m*, 1H), 7.22 – 7.12 (*m*, 3H), 3.54 (br. *s*, 2H), 3.25 (br. *s*, 2H), 2.37 (*s*, 3H), 1.24 (br. *s*, 3H), 1.10 (br. *s*, 3H). ^{13}C NMR (151 MHz, $CDCl_3$) δ (ppm) = 171.5, 138.2, 137.2, 129.7, 128.2, 126.9, 123.1, 43.2, 39.1, 21.4, 14.2, 12.9.

Methyl 2-(2-aminobenzamido)benzoate (**79**) [18]:

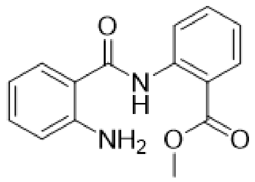

White solid, 80% yield. (Method A). TLC: DCM/MTBE 5:1, 2x. Column chromatography: DCM/MTBE 10:1. ^{1}H NMR (600 MHz, $CDCl_3$) δ (ppm) = 11.80 (*s*, 1H), 8.80 (*dd*, *J* = 8.6, 1.1 Hz, 1H), 8.08 – 8.02 (*m*, 1H), 7.72 (*dd*, *J* = 8.0, 1.4 Hz, 1H), 7.57 (*ddd*, *J* = 8.8, 7.2, 1.7 Hz, 1H), 7.27 – 7.22 (*m*, 1H), 7.11 – 7.05 (*m*, 1H), 6.76 (*ddd*, *J* = 8.1, 7.1, 1.2 Hz, 1H), 6.69 (*dd*, *J* = 8.2, 1.1 Hz, 1H), 5.75 (*s*, 2H), 3.93 (*s*, 3H). ^{13}C NMR (151 MHz, $CDCl_3$) δ (ppm) = 168.9, 168.1, 149.8, 141.9, 134.6, 132.8, 130.9, 127.6, 122.3, 120.4, 117.5, 116.9, 115.7, 115.3, 52.4.

N-(bis(phenylamino)methylene)-3-methylbenzamide (**81**):

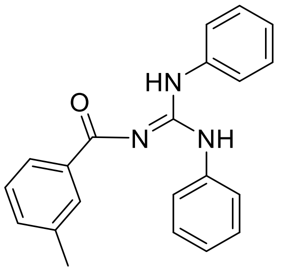

White solid, 88% yield. (Method A). M.p. 96-97ºC. TLC: DCM/MTBE 20:1, 1x. Column chromatography: DCM - DCM/MTBE 20:1. ^{1}H NMR (600 MHz, DMSO-d[6]) δ (ppm) = 10.29 (br s, 2H, NH), 7.81 (br s, 2H), 7.00-7.67 (m, 12H), 2.35 (s, 3H). ^{13}C NMR (151 MHz, DMSO-d[6]) δ (ppm) = 136.66, 131.46, 128.81, 128.29, 127.40, 125.27, 122.84, 20.45. MS (ESI+) m/z (rel. int.): 330 (M+1, 100).

N-(*N*-(*N*,*N*-dimethylcarbamimidoyl)carbamimidoyl)benzamide (**83**) [19]:

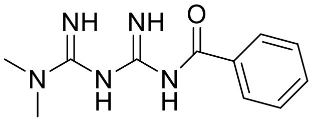

White solid, 76% yield. (Method A). TLC: DCM/MTBE 10:1 1x. Column chromatography: DCM/i-PrOH. ^{1}H NMR (600 MHz, DMSO-d[6]): δ (ppm) = 8.30 (*m*, 2H), 7.54 – 7.49 (*m*, 1H), 7.49 – 7.44 (*m*, 2H), 6.87 (*s*, 2H), 3.20 (*s*, 3H), 3.09 (*s*, 3H). ^{13}C NMR (151 MHz, DMSO-d[6]) δ (ppm) = 169.3, 167.1, 165.5, 137.2, 131.1, 128.2, 127.8, 40.1, 35.8.

References:

1. *Org. Lett.*, **2019**, *21*, pp 4878–4881. https://doi.org/10.1021/acs.orglett.9b01772
2. *J. Org. Chem.*, **2018**, *83*, pp 14307–14313. https://doi.org/10.1021/acs.joc.8b01956
3. *Tetrahedron*, **2018**, *74*, pp 2030-2040. https://doi.org/10.1016/j.tet.2018.03.007
4. *Tetrahedron*, **2020**, *76*, 131079. https://doi.org/10.1016/j.tet.2020.131079
5. *J. Org. Chem.*, **2016**, *81*, pp 10688–10697. https://doi.org/10.1021/acs.joc.6b01686
6. *Org. Biomol. Chem.*, **2015**, *13*, pp 10888-10894. https://doi.org/10.1039/C5OB01801C
7. *J. Org. Chem.*, **2012**, *77*, pp 9553–9561. https://doi.org/10.1021/jo301466w
8. *Chem. Commun.*, **2014**, *50*, pp 14775-14777. https://doi.org/10.1039/C4CC07378A
9. *Green Chem.*, **2015**, *17*, pp 2741-2744. https://doi.org/10.1039/C5GC00299K
10. *Synth. Comm.*, **2011**, *41*, pp 1275-1266. https://doi.org/10.1080/00397911.2010.481745
11. *Bioorg. Med. Chem. Lett.*, **2010**, *20*, pp 149-152. https://doi.org/10.1016/j.bmcl.2009.11.022
12. *J. Am. Chem. Soc.*, **2016**, *138*, pp 14218–14221. https://doi.org/10.1021/jacs.6b09482
13. *J. Org. Chem.*, **2020**, *85*, pp 14545–14558. https://doi.org/10.1021/acs.joc.0c01151
14. *Org. Lett.*, **2020**, *22*, pp 6143–6149. https://doi.org/10.1021/acs.orglett.0c02207
15. *Org. Biomol. Chem.*, **2021**, *24*, pp 5342-5347. https://doi.org/10.1039/D1OB00779C
16. *Tetrahedron Lett.*, **2006**, *47*, pp 6281-6284. https://doi.org/10.1016/j.tetlet.2006.06.126
17. *Synthesis*, **2016**, *48*, pp 2069-2078. http://dx.doi.org/10.1055/s-0035-1561427
18. *RSC Adv.***, 2016,** *6*, pp 27378-27387. https://doi.org/10.1039/C6RA00855K
19. *Eur. J. Med. Chem.*, **2020**, *200*, 112462. https://doi.org/10.1016/j.ejmech.2020.112462

2. X-ray

X-ray data for **77·HCl** was measured using a Bruker-Nonius KappaCCD diffractometer with an APEX-II detector with graphite-monochromatized Mo-*K*α (λ = 0.71073 Å) radiation. Data collection and reduction were performed using the program *COLLECT*[1] and *HKL DENZO AND SCALEPACK*,[2] respectively, and the intensities were corrected for absorption using *SADABS*.[3] Single-crystal X-ray data for **81** was measured using an Agilent SuperNova dual-source Oxford diffractometer equipped with an Eos detector using mirror-monochromated Cu-*K*α (λ = 1.54184 Å) radiation. The data collection and reduction were performed using the program *CrysAlisPro* and Gaussian face index absorption correction method was applied.[4] The structures were solved with intrinsic phasing (ShelXT)[5] and refined by full-matrix least squares on F^2 using the *Olex2* software,[6] which utilises the ShelXL-2015 module.[7] Anisotropic displacement parameters were assigned to non-H atoms and isotropic displacement parameters for all H atoms were constrained to multiples of the equivalent displacement parameters of their parent atoms with $U_{iso}(H) = 1.2\ U_{eq}$ (aromatic) or $U_{iso}(H) = 1.5\ U_{eq}$ (alkyl) of their respective parent atoms. The X-ray single crystal data and CCDC numbers of all new structures are included below.

Crystals suitable for single crystal X-ray diffraction were obtained by slow evaporation of a 4:1 dichloromethane:acetonitrile solution of **77**. Crystal data for **77·HCl**: CCDC-2127766, $[C_{15}H_{17}N_2O]Cl$, M = 276.75, colourless needle, 0.04 × 0.06 × 0.28=2 mm^3, monoclinic, space group $P2_1/n$, a = 4.8798(3) Å, b = 17.8602(9) Å, c = 15.7045(8) Å, β = 90.206(3)°, V = 1368.70(13) Å^3, Z = 4, D_{calc} = 1.343 gcm^{-3}, F(000) = 584, μ = 0.27 mm^{-1}, T = 170.0(1) K, θ_{max} = 27.9°, 2490 total reflections, 1526 with $I_o > 2\sigma(I_o)$, R_{int} = 0.088, 2490 data, 177 parameters, no restraints, GooF = 1.03, 0.30 < dΔρ < –0.30 eÅ^{-3}, $R[F^2 > 2\sigma(F^2)] = 0.071$, $wR(F^2) = 0.151$.

Crystals suitable for single crystal X-ray diffraction were obtained by slow evaporation of a diisopropyl ether solution of **81**. Crystal data for **81**: CCDC-2127765, $C_{21}H_{19}N_3O$, M = 329.39, colourless plate, 0.03 × 0.19 × 0.28 mm^3, monoclinic, space group $P2_1/c$, a = 8.7084(3) Å, b = 10.2040(5) Å, c = 19.0448(7) Å, β = 92.691(4)°, V = 1690.46(12) Å^3, Z = 4, D_{calc} = 1.294 gcm^{-3}, F(000) = 696, μ = 0.64 mm^{-1}, T = 120.0(1) K, θ_{max} = 76.0°, 3428 total reflections, 2754 with $I_o > 2\sigma(I_o)$, R_{int} = 0.029, 3428 data, 233 parameters, no restraints, GooF = 1.03, 0.26 < dΔρ < –0.23 eÅ^{-3}, $R[F^2 > 2\sigma(F^2)] = 0.043$, $wR(F^2) = 0.118$.

[1] R.W.W. Hooft, *COLLECT* **1998**, Nonius BV, Delft, The Netherlands.
(computer program - no DOI: https://iucrdata.iucr.org/x/services/stdswrefs.html)
[2] Otwinowski, Z.; Minor, W. *Methods in Enzymology, Macromolecular Crystallography, Part A* **1997**, 276, 307-326. Edited by C. W. Carter Jr & R. M. Sweet, New York: Academic Press.
(https://doi.org/10.1016/S0076-6879(97)76066-X)
[3] Sheldrick, G. M. *SADABS* Version 2008/2 **1996**, University of Göttingen, Germany.
(computer program - no DOI: https://iucrdata.iucr.org/x/services/stdswrefs.html)
[4] Rigaku Oxford Diffraction, 2018, *CrysAlisPro* Software system, Rigaku Corporation, Oxford, UK.
(computer program - no DOI: https://iucrdata.iucr.org/x/services/stdswrefs.html)
[5] G. M. Sheldrick, *Acta Crystallogr. Sect. A*, 2015, **71**, 3–8.
(https://doi.org/10.1107/S2053273314026370)
[6] O. V Dolomanov, L. J. Bourhis, R. J. Gildea, J. A. K. Howard and H. Puschmann, *J. Appl. Crystallogr.*, 2009, **42**, 339–341.
(https://doi.org/10.1107/S0021889808042726)
[7] G. M. Sheldrick, *Acta Crystallogr. Sect. C*, 2015, **71**, 3–8.
(https://doi.org/10.1107/S2053229614024218)

^{1}H and ^{13}C NMR images

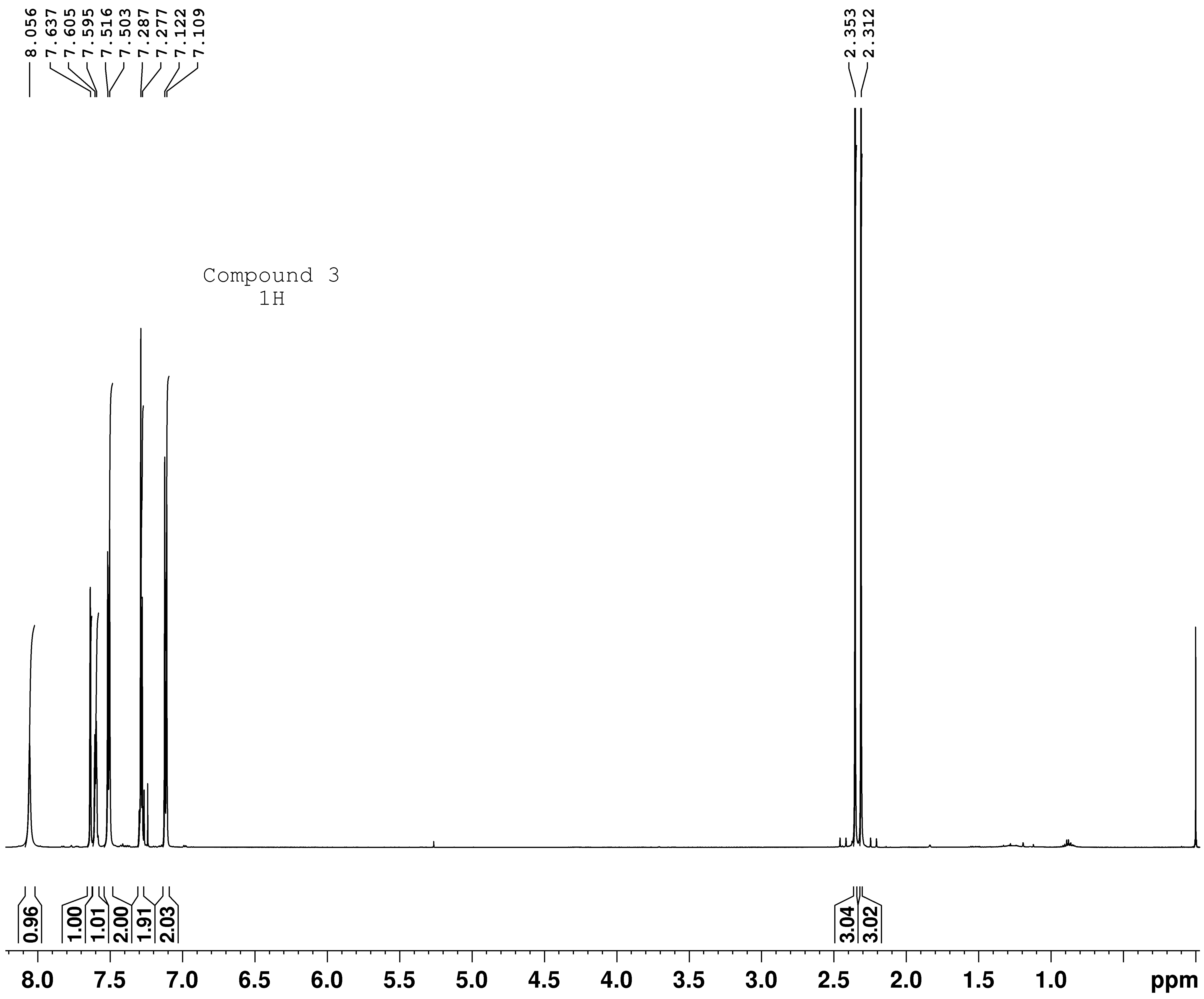


```
Current Data Parameters
NAME          03-CD-81A
EXPNO                11
PROCNO                1

F2 - Acquisition Parameters
Date_          20220111
Time              16.12 h
INSTRUM           spect
PROBHD   Z847801_0047 (
PULPROG            zg30
TD                32768
SOLVENT           CDCl3
NS                   16
DS                    0
SWH            9615.385 Hz
FIDRES         0.586877 Hz
AQ            1.7039360 sec
RG                   64
DW               52.000 usec
DE                14.38 usec
TE                293.0 K
D1           1.00000000 sec
TD0                   1
SFO1        599.9845606 MHz
NUC1                 1H
P0                 3.60 usec
P1                10.80 usec
PLW1        20.00000000 W

F2 - Processing parameters
SI                65536
SF          599.9800260 MHz
WDW                  EM
SSB                   0
LB                    0 Hz
GB                    0
PC                 1.00
```

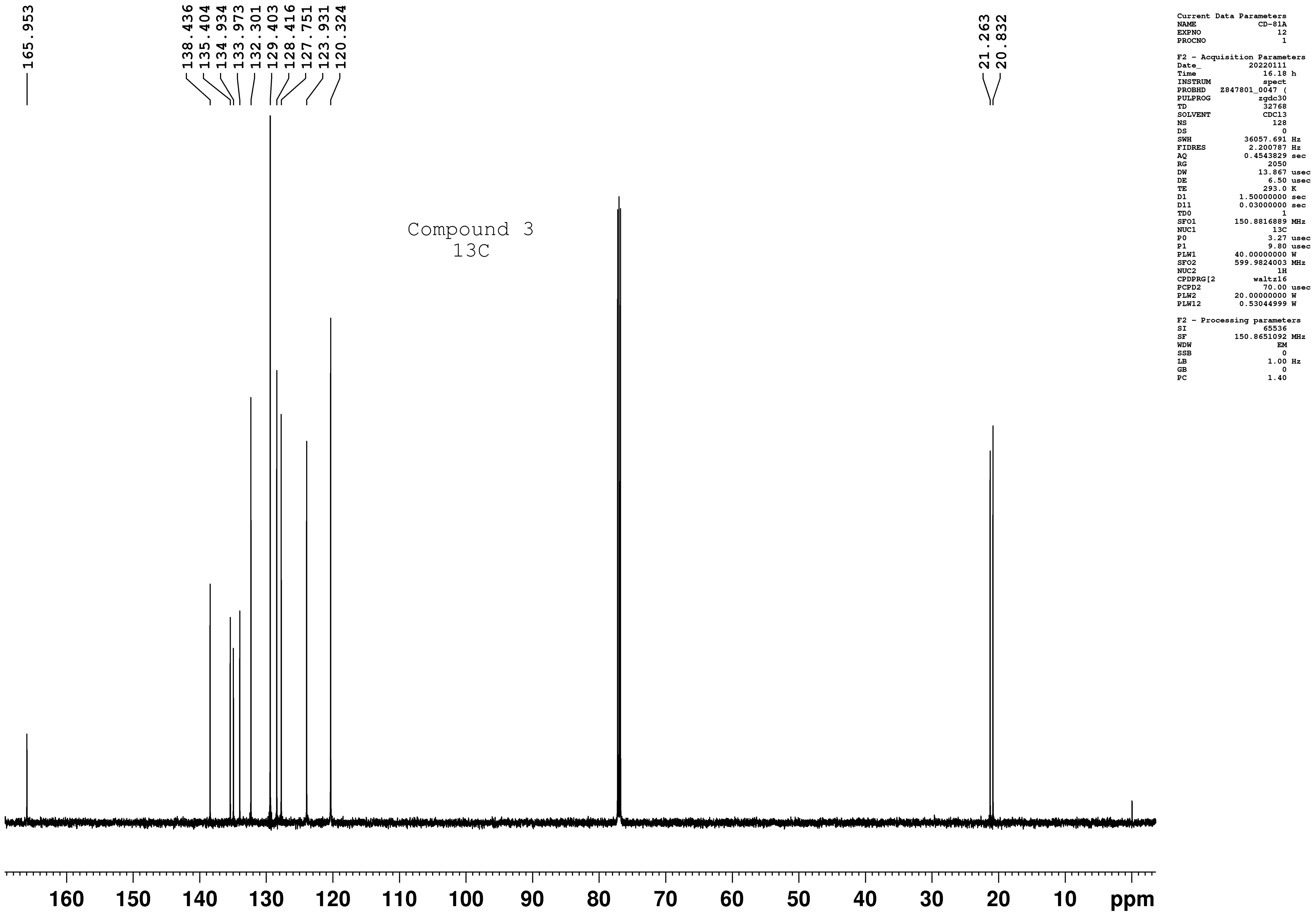
165.953
138.436
135.404
134.934
133.973
132.301
129.403
128.416
127.751
123.931
120.324
21.263
20.832
Compound 3
13C
160
150
140
130
120
110
100
90
80
70
60
50
40
30
20
10
ppm
Current Data Parameters
NAME CD-81A
EXPNO 12
PROCNO 1
F2 - Acquisition Parameters
Date_ 20220111
Time 16.18 h
INSTRUM spect
PROBHD Z847801_0047 (
PULPROG zgdc30
TD 32768
SOLVENT CDCl3
NS 128
DS 0
SWH 36057.691 Hz
FIDRES 2.200787 Hz
AQ 0.4543829 sec
RG 2050
DW 13.867 usec
DE 6.50 usec
TE 293.0 K
D1 1.50000000 sec
D11 0.03000000 sec
TD0 1
SFO1 150.8816889 MHz
NUC1 13C
P0 3.27 usec
P1 9.80 usec
PLW1 40.00000000 W
SFO2 599.9824003 MHz
NUC2 1H
CPDPRG[2 waltz16
PCPD2 70.00 usec
PLW2 20.00000000 W
PLW12 0.53044999 W
F2 - Processing parameters
SI 65536
SF 150.8651092 MHz
WDW EM
SSB 0
LB 1.00 Hz
GB 0
PC 1.40

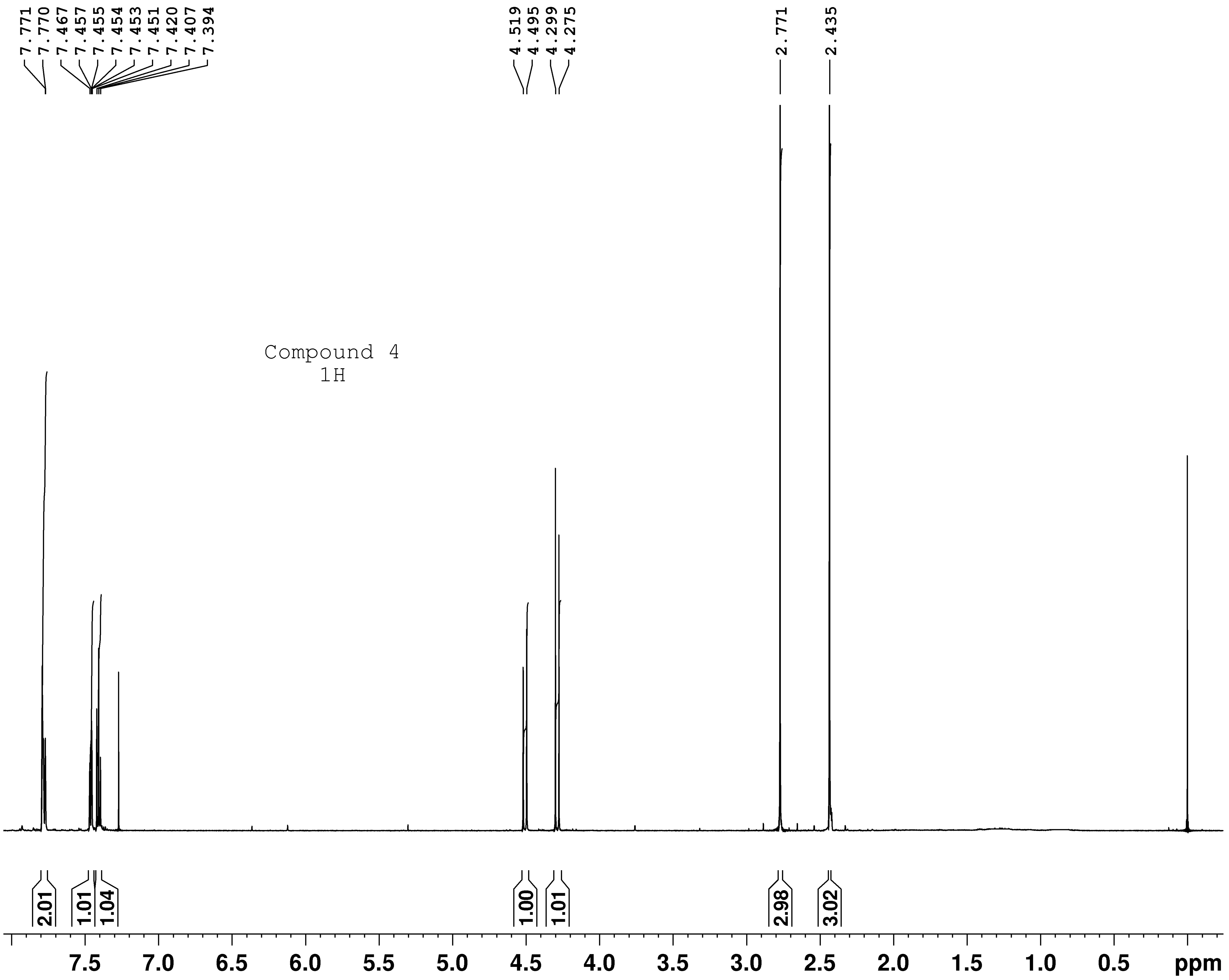


```
Current Data Parameters
NAME                CD-78
EXPNO                  10
PROCNO                  1

F2 - Acquisition Parameters
Date_            20191203
Time                17.08 h
INSTRUM             spect
PROBHD   Z847801_0047 (
PULPROG              zg30
TD                  32768
SOLVENT             CDCl3
NS                      1
DS                      0
SWH              9615.385 Hz
FIDRES           0.586877 Hz
AQ              1.7039360 sec
RG                    161
DW                 52.000 usec
DE                  13.95 usec
TE                  293.0 K
D1             1.00000000 sec
TD0                     1
SFO1          600.0145608 MHz
NUC1                   1H
P1                  10.85 usec
PLW1          20.00000000 W

F2 - Processing parameters
SI                  65536
SF            600.0100088 MHz
WDW                    EM
SSB                     0
LB                      0 Hz
GB                      0
PC                   1.00
```

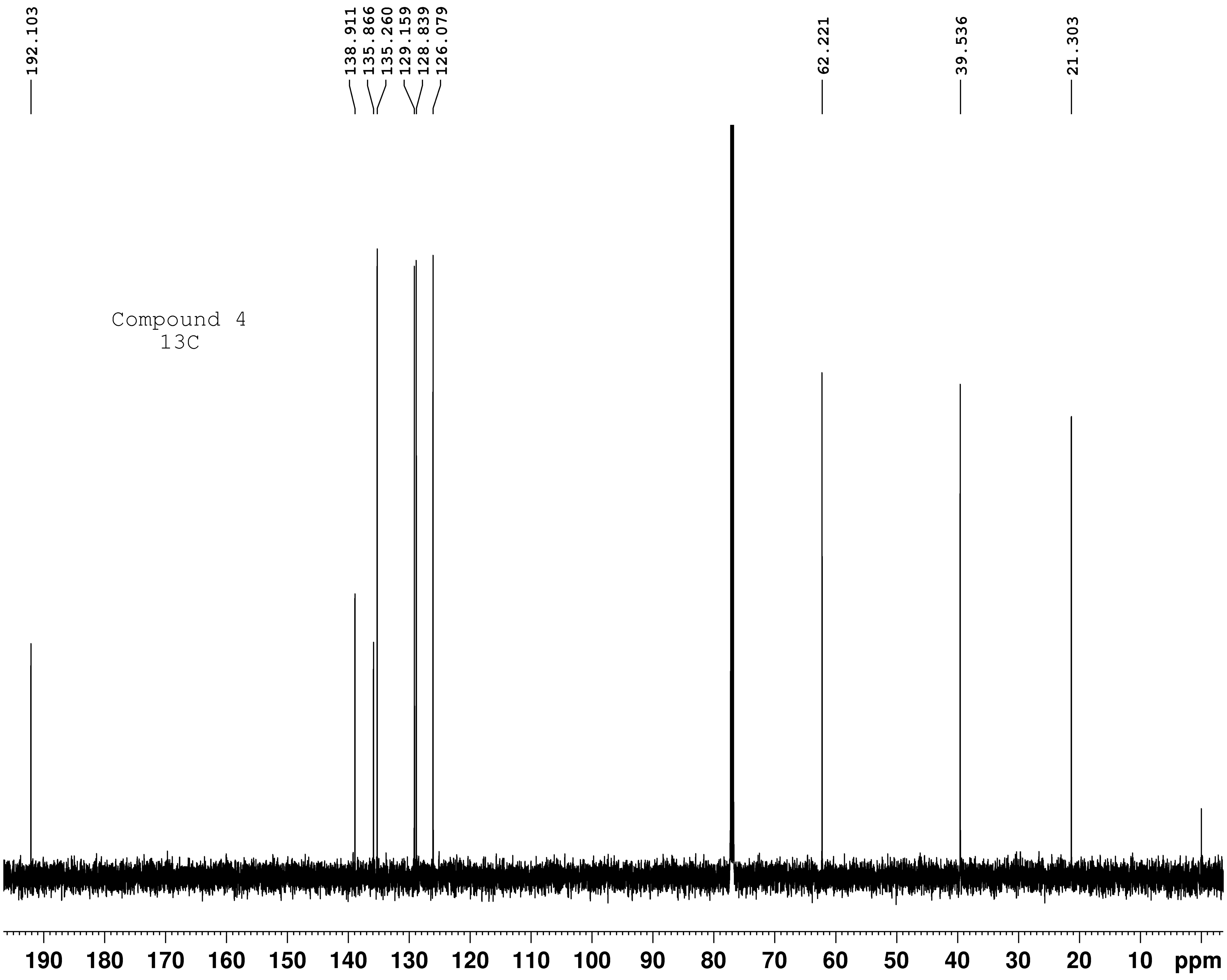


```
Current Data Parameters
NAME                CD-78
EXPNO                  12
PROCNO                  1

F2 - Acquisition Parameters
Date_            20191203
Time                17.15 h
INSTRUM             spect
PROBHD   Z847801_0047 (
PULPROG           zgdc30
TD                  32768
SOLVENT             CDCl3
NS                    128
DS                      0
SWH             36057.691 Hz
FIDRES           2.200787 Hz
AQ              0.4543829 sec
RG                   2050
DW                 13.867 usec
DE                   6.50 usec
TE                  293.0 K
D1             1.50000000 sec
D11            0.03000000 sec
TD0                     1
SFO1          150.8892338 MHz
NUC1                  13C
P1                   9.80 usec
PLW1          40.00000000 W
SFO2          600.0124004 MHz
NUC2                   1H
CPDPRG[2          waltz16
PCPD2               90.00 usec
PLW2          20.00000000 W
PLW12          0.33800000 W

F2 - Processing parameters
SI                  65536
SF            150.8726450 MHz
WDW                    EM
SSB                     0
LB                   1.00 Hz
GB                      0
PC                   1.40
```

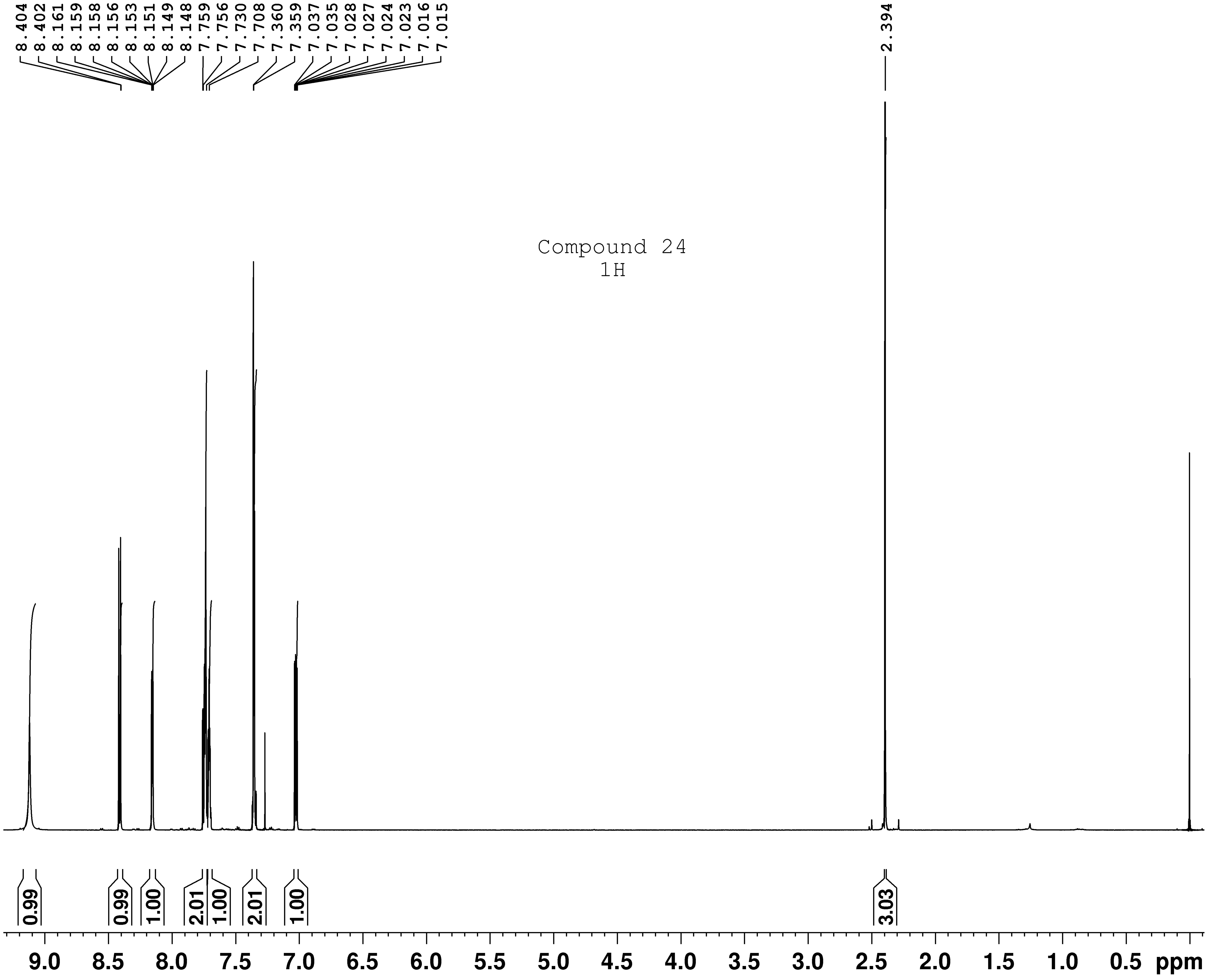

Current Data Parameters
NAME 24-CD-109
EXPNO 11
PROCNO 1

F2 - Acquisition Parameters
Date_ 20220111
Time 16.43 h
INSTRUM spect
PROBHD Z847801_0047 (
PULPROG zg30
TD 32768
SOLVENT CDCl3
NS 16
DS 0
SWH 9615.385 Hz
FIDRES 0.586877 Hz
AQ 1.7039360 sec
RG 90.5
DW 52.000 usec
DE 14.38 usec
TE 293.0 K
D1 1.00000000 sec
TD0 1
SFO1 599.9845606 MHz
NUC1 1H
P0 3.60 usec
P1 10.80 usec
PLW1 20.00000000 W

F2 - Processing parameters
SI 65536
SF 599.9800087 MHz
WDW EM
SSB 0
LB 0 Hz
GB 0
PC 1.00

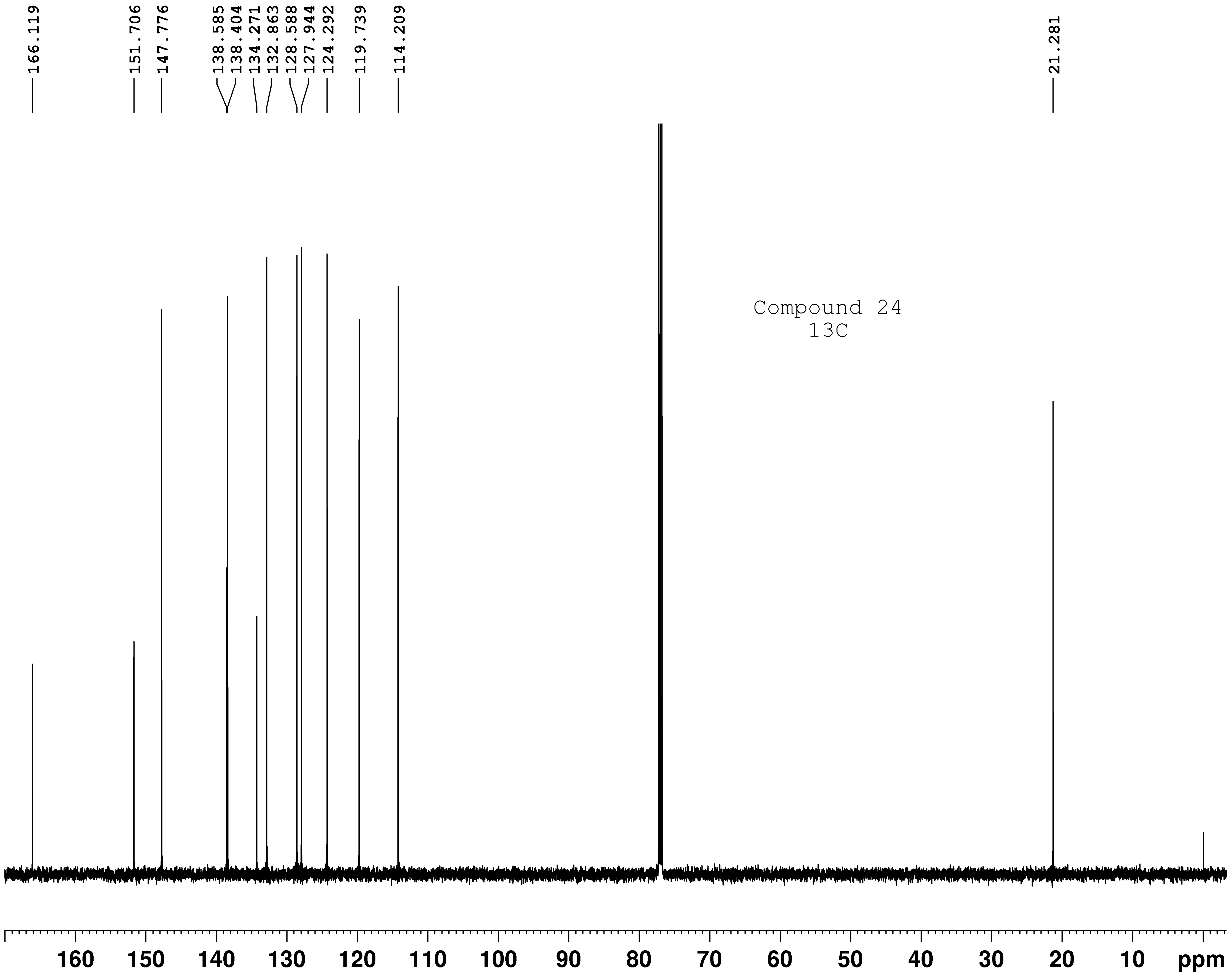

Current Data Parameters
NAME CD-109
EXPNO 12
PROCNO 1

F2 - Acquisition Parameters
Date_ 20220111
Time 16.49 h
INSTRUM spect
PROBHD Z847801_0047 (
PULPROG zgdc30
TD 32768
SOLVENT CDCl3
NS 128
DS 0
SWH 36057.691 Hz
FIDRES 2.200787 Hz
AQ 0.4543829 sec
RG 2050
DW 13.867 usec
DE 6.50 usec
TE 293.0 K
D1 1.50000000 sec
D11 0.03000000 sec
TD0 1
SFO1 150.8816889 MHz
NUC1 13C
P0 3.27 usec
P1 9.80 usec
PLW1 40.00000000 W
SFO2 599.9824003 MHz
NUC2 1H
CPDPRG[2 waltz16
PCPD2 70.00 usec
PLW2 20.00000000 W
PLW12 0.53044999 W

F2 - Processing parameters
SI 65536
SF 150.8651044 MHz
WDW EM
SSB 0
LB 1.00 Hz
GB 0
PC 1.40

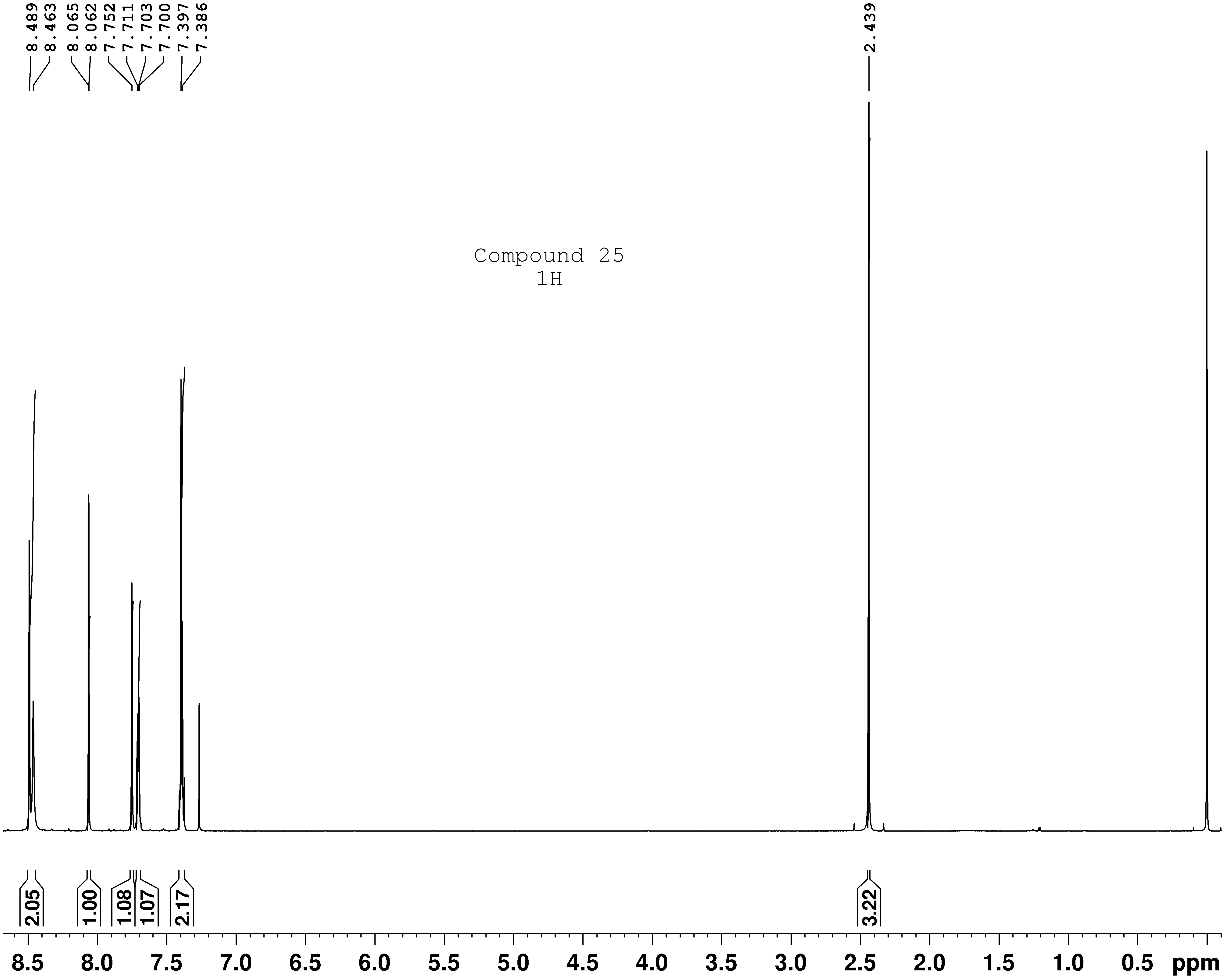

Current Data Parameters

NAME	CD-99A	
EXPNO	11	
PROCNO	1	

F2 - Acquisition Parameters

Date_	20210217	
Time	18.33	h
INSTRUM	spect	
PROBHD	Z847801_0047 (	
PULPROG	zg30	
TD	32768	
SOLVENT	CDCl3	
NS	32	
DS	0	
SWH	9615.385	Hz
FIDRES	0.586877	Hz
AQ	1.7039360	sec
RG	161	
DW	52.000	usec
DE	14.37	usec
TE	293.0	K
D1	1.00000000	sec
TD0	1	
SFO1	600.0145608	MHz
NUC1	1H	
P0	3.62	usec
P1	10.85	usec
PLW1	20.00000000	W

F2 - Processing parameters

SI	65536	
SF	600.0100104	MHz
WDW	EM	
SSB	0	
LB	0	Hz
GB	0	
PC	1.00	

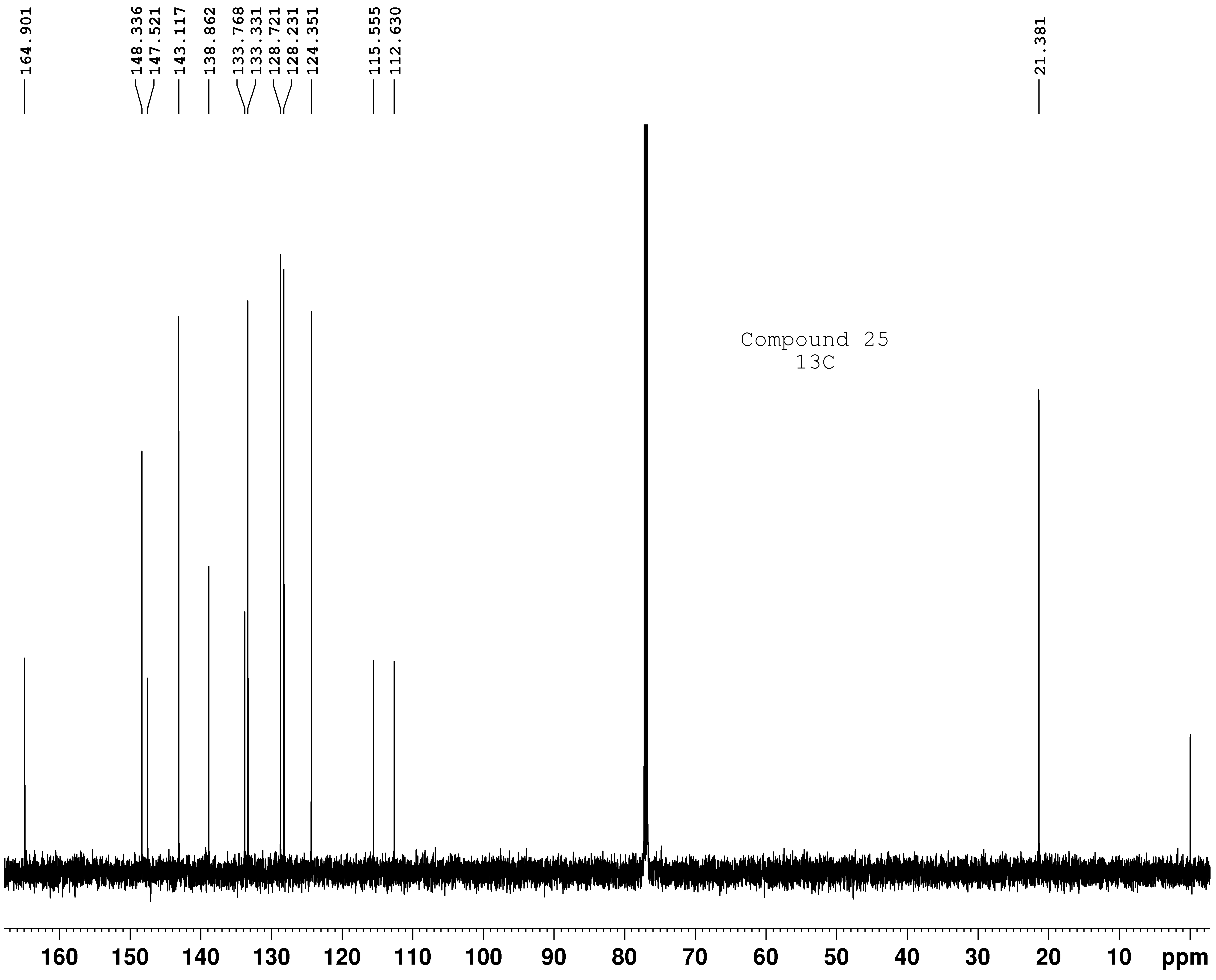


```
Current Data Parameters
NAME              CD-99A
EXPNO                 16
PROCNO                 1

F2 - Acquisition Parameters
Date_           20210217
Time               19.08 h
INSTRUM            spect
PROBHD   Z847801_0047 (
PULPROG           zgdc30
TD                 32768
SOLVENT            CDCl3
NS                   128
DS                     0
SWH            36057.691 Hz
FIDRES          2.200787 Hz
AQ             0.4543829 sec
RG                  2050
DW                13.867 usec
DE                  6.50 usec
TE                 293.0 K
D1            1.50000000 sec
D11           0.03000000 sec
TD0                    1
SFO1         150.8892338 MHz
NUC1                 13C
P0                  3.27 usec
P1                  9.80 usec
PLW1         40.00000000 W
SFO2         600.0124004 MHz
NUC2                  1H
CPDPRG[2         waltz16
PCPD2              90.00 usec
PLW2         20.00000000 W
PLW12         0.33800000 W

F2 - Processing parameters
SI                 65536
SF           150.8726459 MHz
WDW                   EM
SSB                    0
LB                  1.00 Hz
GB                     0
PC                  1.40
```

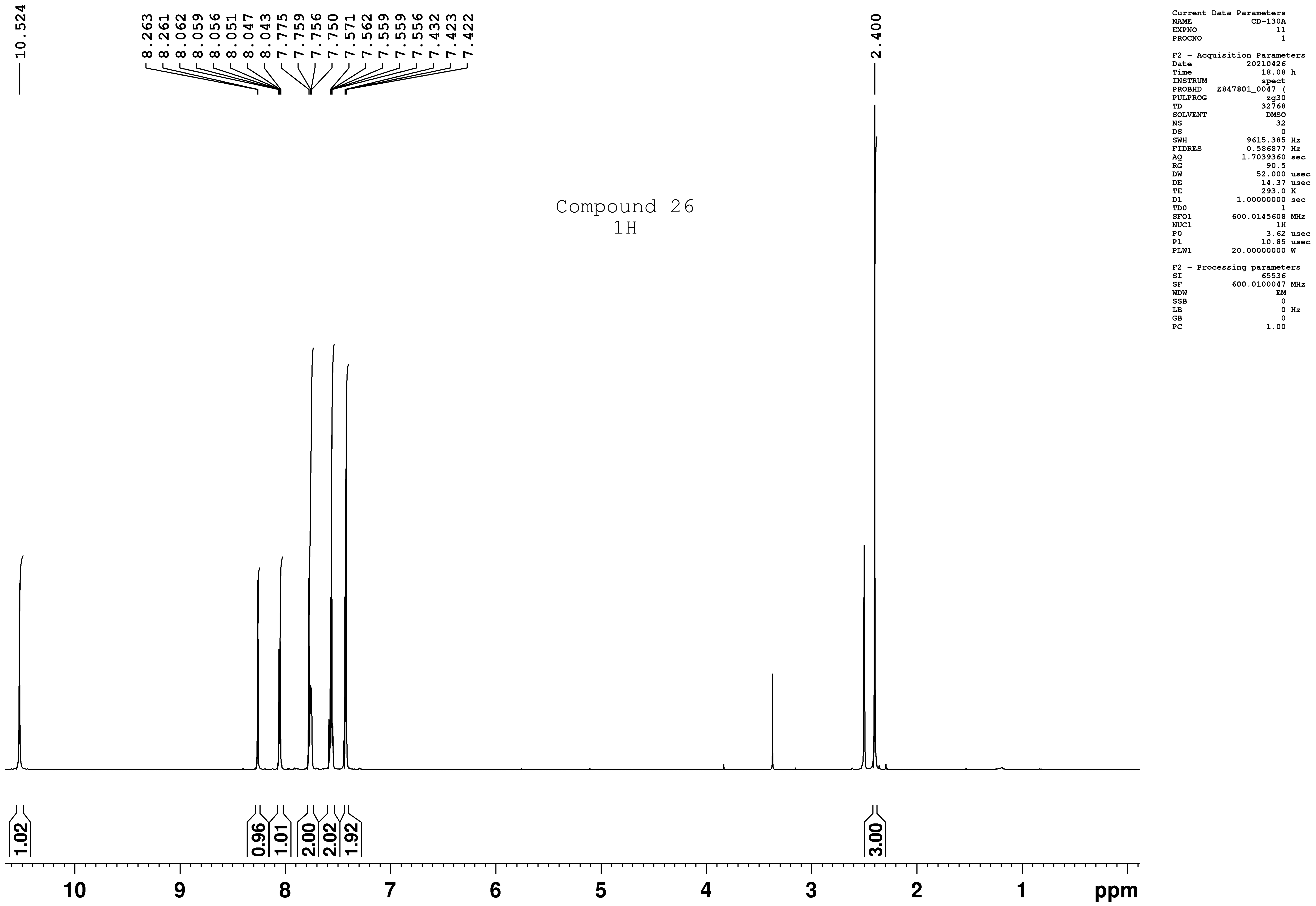
10.524
8.263
8.261
8.062
8.059
8.056
8.051
8.047
8.043
7.775
7.759
7.756
7.750
7.571
7.562
7.559
7.559
7.556
7.432
7.423
7.422
2.400
Compound 26
1H
1.02
0.96
1.01
2.00
2.02
1.92
3.00
10
9
8
7
6
5
4
3
2
1
ppm
Current Data Parameters
NAME CD-130A
EXPNO 11
PROCNO 1
F2 - Acquisition Parameters
Date_ 20210426
Time 18.08 h
INSTRUM spect
PROBHD Z847801_0047 (
PULPROG zg30
TD 32768
SOLVENT DMSO
NS 32
DS 0
SWH 9615.385 Hz
FIDRES 0.586877 Hz
AQ 1.7039360 sec
RG 90.5
DW 52.000 usec
DE 14.37 usec
TE 293.0 K
D1 1.00000000 sec
TD0 1
SFO1 600.0145608 MHz
NUC1 1H
P0 3.62 usec
P1 10.85 usec
PLW1 20.00000000 W
F2 - Processing parameters
SI 65536
SF 600.0100047 MHz
WDW EM
SSB 0
LB 0 Hz
GB 0
PC 1.00

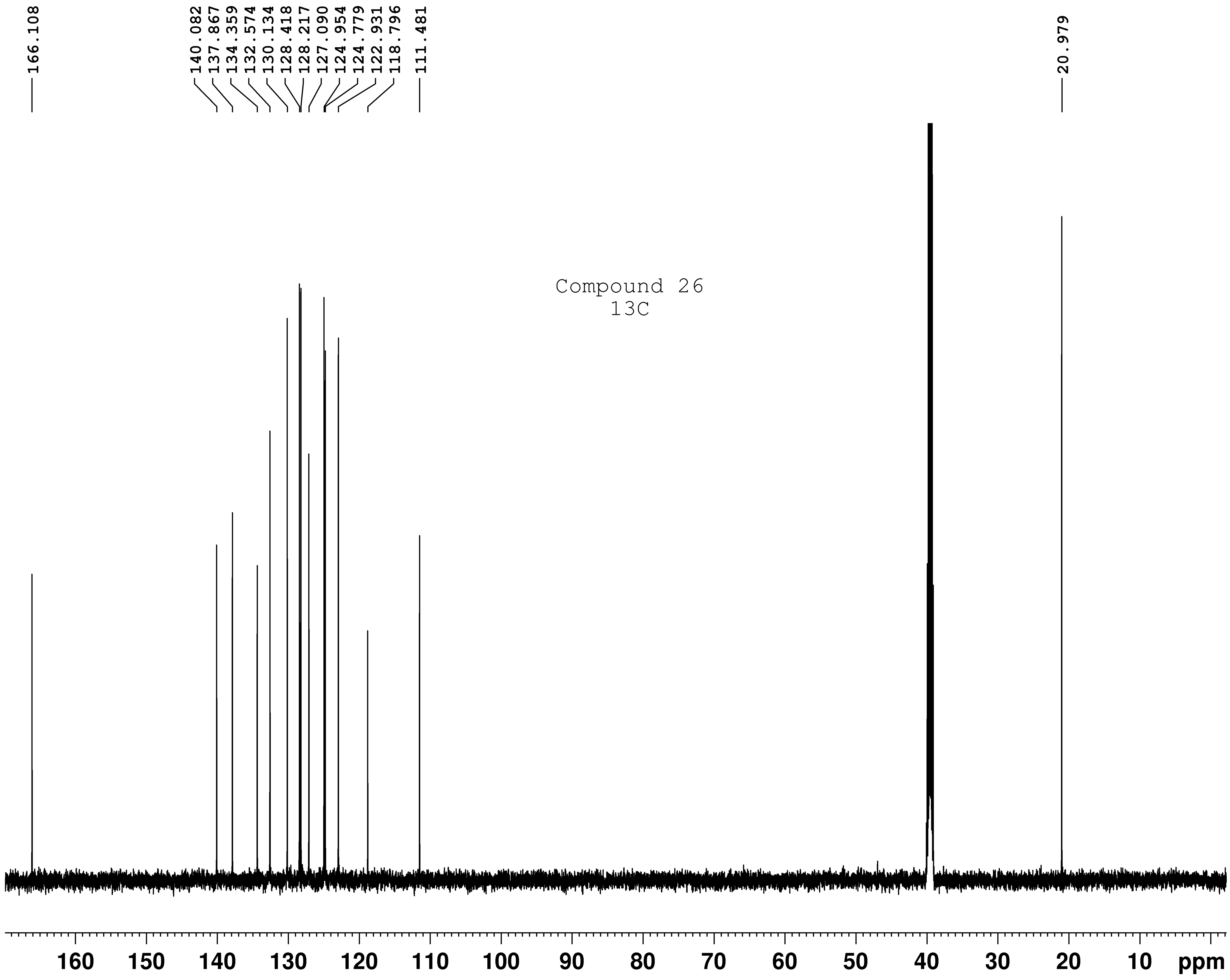


```
Current Data Parameters
NAME              CD-130A
EXPNO                  16
PROCNO                  1

F2 - Acquisition Parameters
Date_            20210426
Time                18.43 h
INSTRUM             spect
PROBHD   Z847801_0047 (
PULPROG           zgdc30
TD                 32768
SOLVENT              DMSO
NS                    128
DS                      0
SWH            36057.691 Hz
FIDRES          2.200787 Hz
AQ             0.4543829 sec
RG                   2050
DW                 13.867 usec
DE                   6.50 usec
TE                  293.0 K
D1            1.50000000 sec
D11           0.03000000 sec
TD0                     1
SFO1         150.8892338 MHz
NUC1                  13C
P0                   3.27 usec
P1                   9.80 usec
PLW1         40.00000000 W
SFO2         600.0124004 MHz
NUC2                   1H
CPDPRG[2          waltz16
PCPD2               90.00 usec
PLW2         20.00000000 W
PLW12         0.33800000 W

F2 - Processing parameters
SI                  65536
SF           150.8727014 MHz
WDW                    EM
SSB                     0
LB                   1.00 Hz
GB                      0
PC                   1.40
```

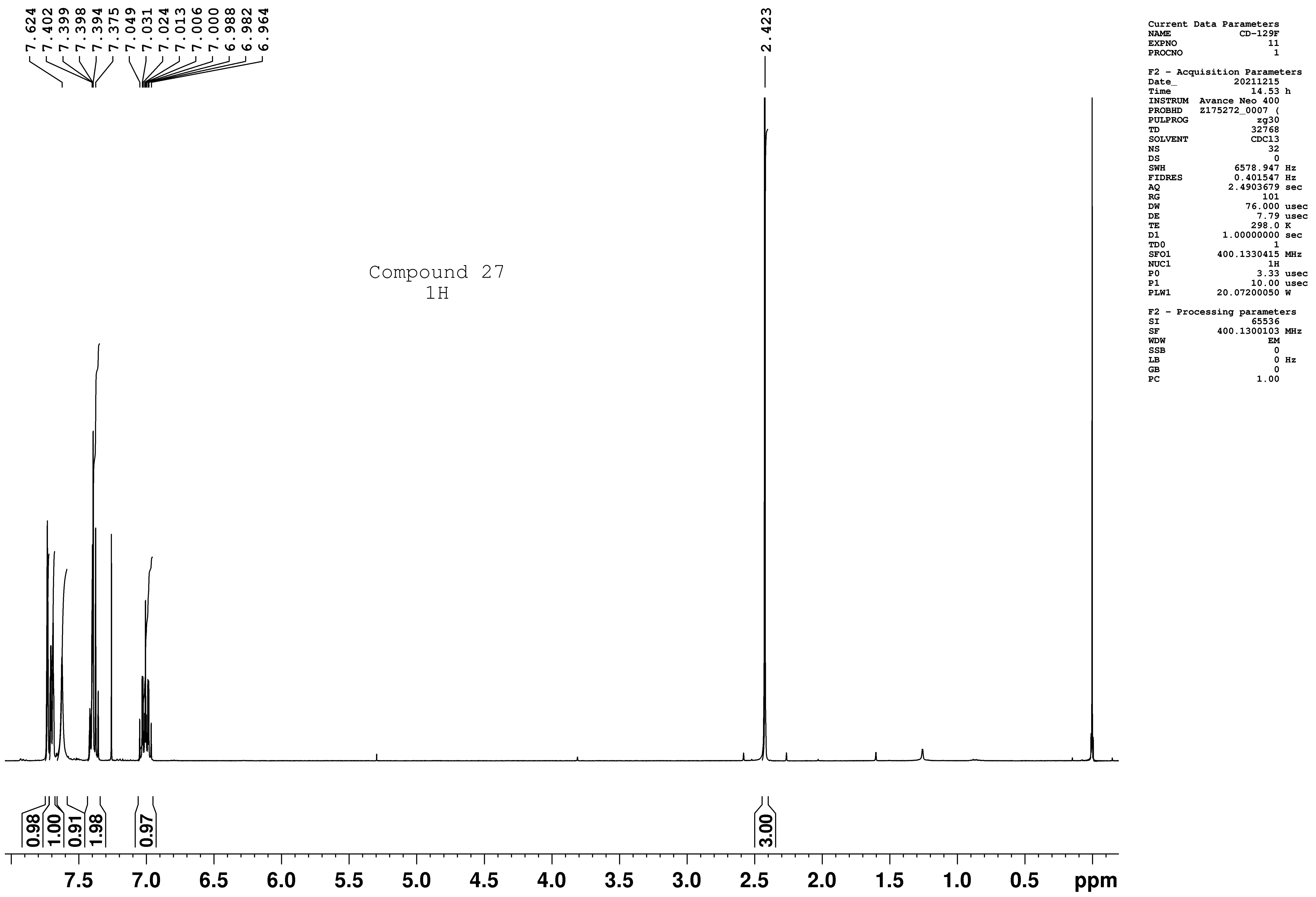
7.624
7.402
7.399
7.398
7.394
7.375
7.049
7.031
7.024
7.013
7.006
7.000
6.988
6.982
6.964
2.423
Compound 27
1H
0.98
1.00
0.91
1.98
0.97
3.00
7.5
7.0
6.5
6.0
5.5
5.0
4.5
4.0
3.5
3.0
2.5
2.0
1.5
1.0
0.5
ppm
Current Data Parameters
NAME CD-129F
EXPNO 11
PROCNO 1
F2 - Acquisition Parameters
Date_ 20211215
Time 14.53 h
INSTRUM Avance Neo 400
PROBHD Z175272_0007 (
PULPROG zg30
TD 32768
SOLVENT CDCl3
NS 32
DS 0
SWH 6578.947 Hz
FIDRES 0.401547 Hz
AQ 2.4903679 sec
RG 101
DW 76.000 usec
DE 7.79 usec
TE 298.0 K
D1 1.00000000 sec
TD0 1
SFO1 400.1330415 MHz
NUC1 1H
P0 3.33 usec
P1 10.00 usec
PLW1 20.07200050 W
F2 - Processing parameters
SI 65536
SF 400.1300103 MHz
WDW EM
SSB 0
LB 0 Hz
GB 0
PC 1.00

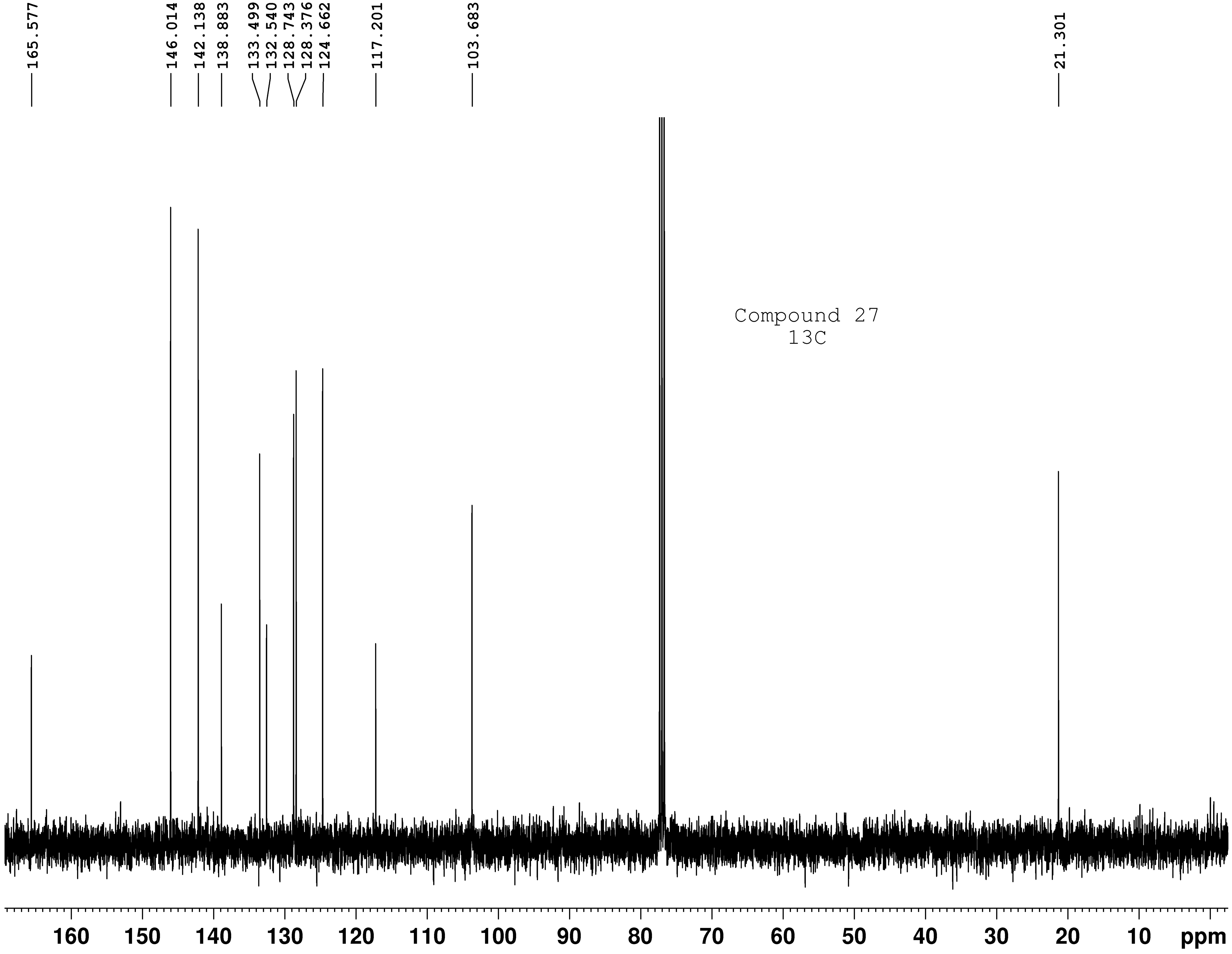


```
Current Data Parameters
NAME              CD-129F
EXPNO                  16
PROCNO                  1

F2 - Acquisition Parameters
Date_            20211215
Time                15.37 h
INSTRUM  Avance Neo 400
PROBHD   Z175272_0007 (
PULPROG  zg30igf2igf3.yavor
TD                  32768
SOLVENT             CDCl3
NS                    128
DS                      0
SWH             23809.523 Hz
FIDRES           1.453218 Hz
AQ              0.6881280 sec
RG                25.7622
DW                 21.000 usec
DE                   6.50 usec
TE                  298.4 K
D1             1.50000000 sec
D11            0.03000000 sec
P1                  10.00 usec
TD0                     1
SFO1          100.6238383 MHz
NUC1                  13C
P0                   3.30 usec
PLW1          58.46900177 W
SFO2          376.4607162 MHz
NUC2                  19F
CPDPRG[2 bi_p5m4sp_4sp.2
P63               1500.00 usec
PLW2          65.31900024 W
PLW12                   0 W
SPNAM[14 Crp120,0.75,30.4
SPOAL14             0.500
SPOFFS14                0 Hz
SPW14         23.06747055 W
SPNAM[31 Crp120,1.5,30.4
SPOAL31             0.500
SPOFFS31                0 Hz
SPW31         11.53349972 W
SFO3          400.1328009 MHz
NUC3                   1H
CPDPRG[3          waltz16
PCPD3               90.00 usec
PLW3          20.00000000 W
PLW16          0.24717240 W

F2 - Processing parameters
SI                  65536
SF            100.6127716 MHz
WDW                    EM
SSB                     0
LB                   1.00 Hz
GB                      0
PC                   1.40
```

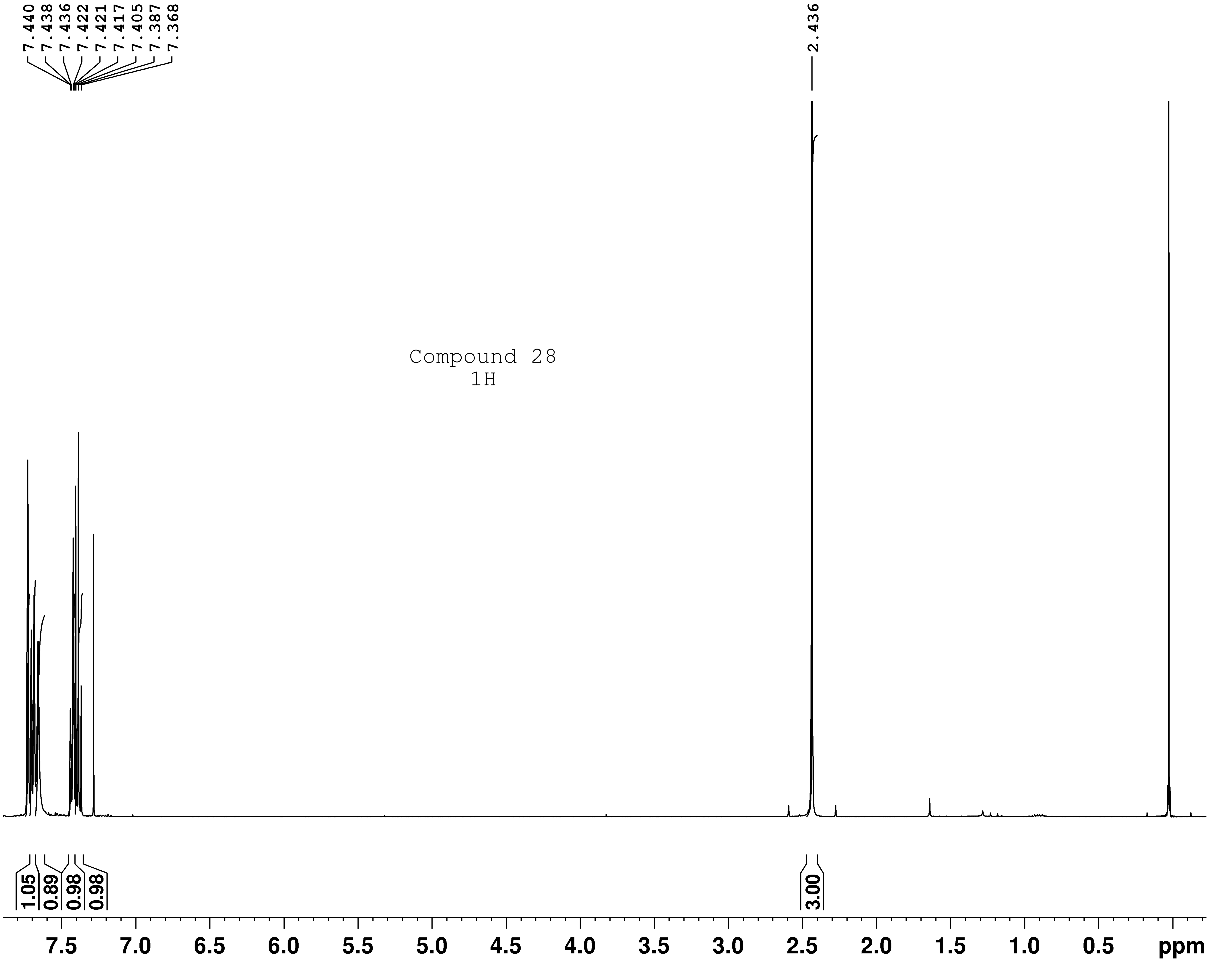

Current Data Parameters		
NAME	CD-114A	
EXPNO	11	
PROCNO	1	
F2 - Acquisition Parameters		
Date_	20211208	
Time	15.06	h
INSTRUM	Avance Neo 400	
PROBHD	Z175272_0007 (	
PULPROG	zg30	
TD	32768	
SOLVENT	CDCl3	
NS	32	
DS	0	
SWH	6578.947	Hz
FIDRES	0.401547	Hz
AQ	2.4903679	sec
RG	101	
DW	76.000	usec
DE	7.79	usec
TE	298.0	K
D1	1.00000000	sec
TD0	1	
SFO1	400.1330415	MHz
NUC1	1H	
P0	3.33	usec
P1	10.00	usec
PLW1	20.07200050	W
F2 - Processing parameters		
SI	65536	
SF	400.1300000	MHz
WDW	EM	
SSB	0	
LB	0	Hz
GB	0	
PC	1.00	

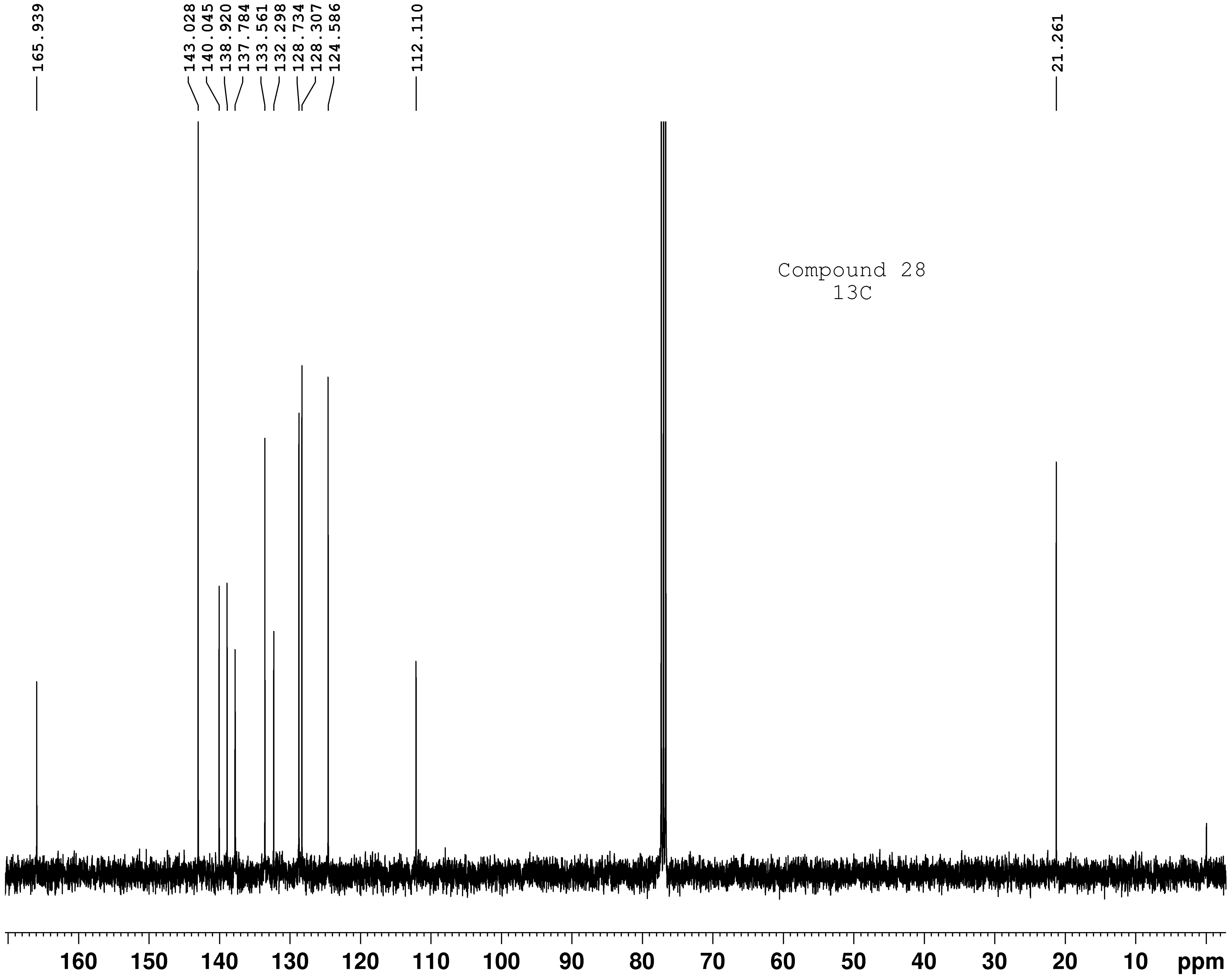


```
Current Data Parameters
NAME            CD-114A
EXPNO               100
PROCNO                1

F2 - Acquisition Parameters
Date_          20211208
Time              15.17 h
INSTRUM  Avance Neo 400
PROBHD   Z175272_0007 (
PULPROG  zg30igf2igf3.yavor
TD                32768
SOLVENT           CDCl3
NS                  128
DS                    0
SWH           23809.523 Hz
FIDRES         1.453218 Hz
AQ            0.6881280 sec
RG                16.25
DW               21.000 usec
DE                 6.50 usec
TE                298.2 K
D1           1.50000000 sec
D11          0.03000000 sec
P1                10.00 usec
TD0                   1
SFO1        100.6238383 MHz
NUC1                13C
P0                 3.30 usec
PLW1        58.46900177 W
SFO2        376.4494212 MHz
NUC2                19F
CPDPRG[2 bi_p5m4sp_4sp.2
P63             1500.00 usec
PLW2        65.31900024 W
PLW12                 0 W
SPNAM[14 Crp32,0.75,20.2
SPOAL14           0.500
SPOFFS14              0 Hz
SPW14        6.00000000 W
SPNAM[31 Crp32,1.5,20.2
SPOAL31           0.500
SPOFFS31              0 Hz
SPW31        3.00000000 W
SFO3        400.1328009 MHz
NUC3                 1H
CPDPRG[3        waltz16
PCPD3             90.00 usec
PLW3        20.00000000 W
PLW16        0.24717240 W

F2 - Processing parameters
SI                65536
SF          100.6127715 MHz
WDW                  EM
SSB                   0
LB                 1.00 Hz
GB                    0
PC                 1.40
```

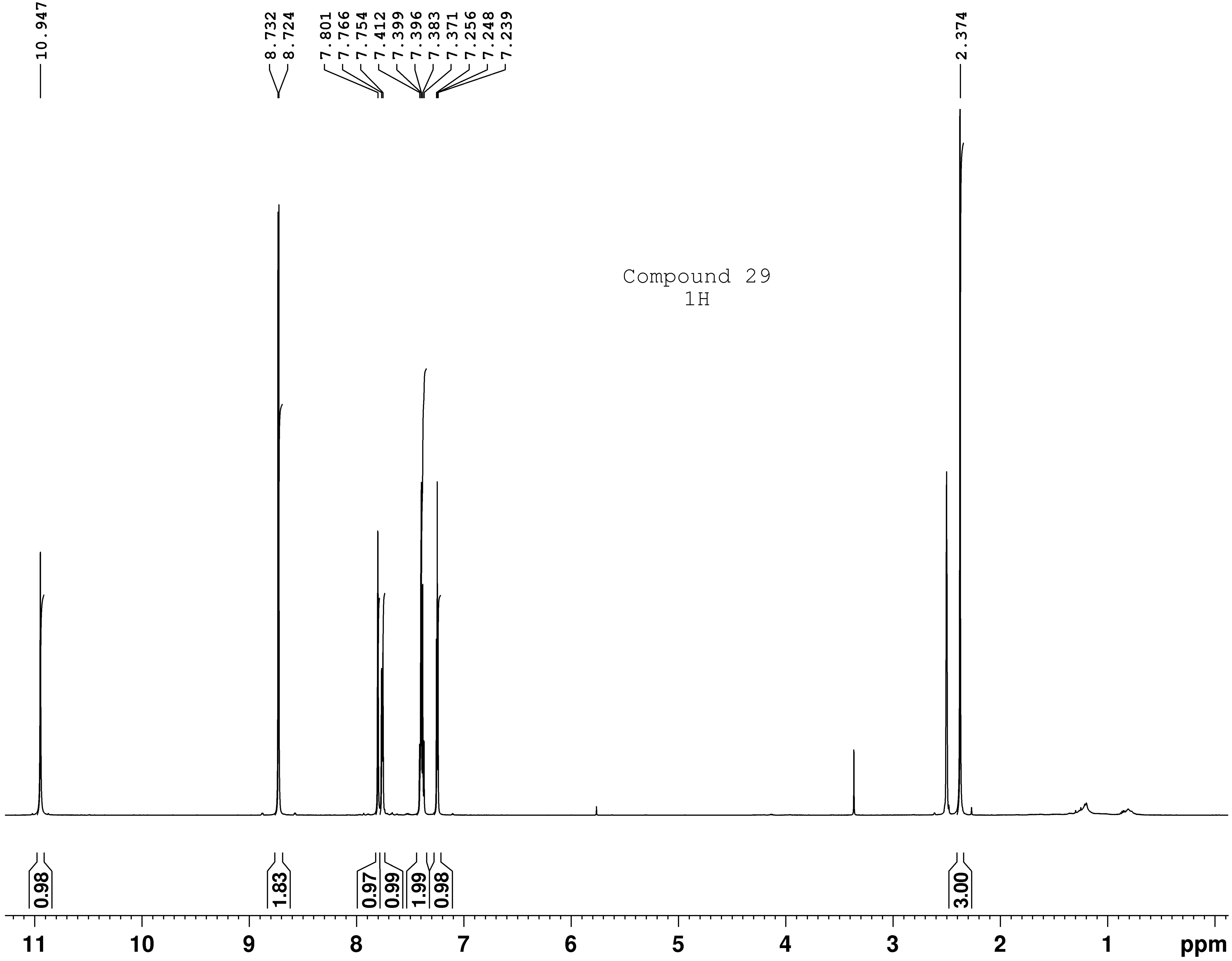

Current Data Parameters
NAME CD-69A
EXPNO 11
PROCNO 1

F2 - Acquisition Parameters
Date_ 20210426
Time 17.23 h
INSTRUM spect
PROBHD Z847801_0047 (
PULPROG zg30
TD 32768
SOLVENT DMSO
NS 16
DS 0
SWH 9615.385 Hz
FIDRES 0.586877 Hz
AQ 1.7039360 sec
RG 101
DW 52.000 usec
DE 14.37 usec
TE 293.1 K
D1 1.00000000 sec
TD0 1
SFO1 600.0145608 MHz
NUC1 1H
P0 3.62 usec
P1 10.85 usec
PLW1 20.00000000 W

F2 - Processing parameters
SI 65536
SF 600.0100049 MHz
WDW EM
SSB 0
LB 0 Hz
GB 0
PC 1.00

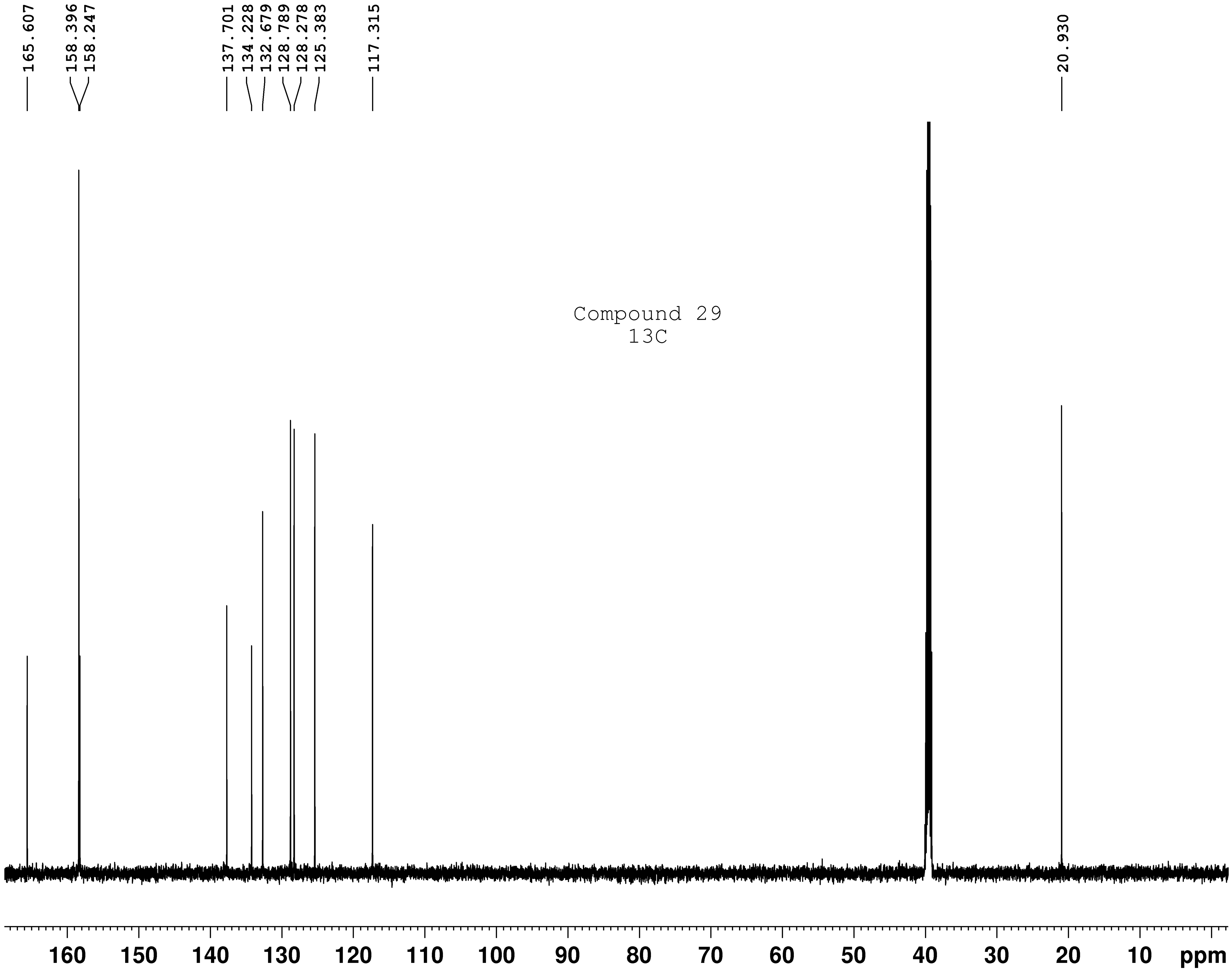


```
Current Data Parameters
NAME                CD-69A
EXPNO                   16
PROCNO                   1

F2 - Acquisition Parameters
Date_             20210426
Time                 17.57 h
INSTRUM              spect
PROBHD   Z847801_0047 (
PULPROG            zgdc30
TD                   32768
SOLVENT               DMSO
NS                     128
DS                       0
SWH              36057.691 Hz
FIDRES            2.200787 Hz
AQ               0.4543829 sec
RG                    2050
DW                  13.867 usec
DE                    6.50 usec
TE                   293.0 K
D1              1.50000000 sec
D11             0.03000000 sec
TD0                      1
SFO1          150.8892338 MHz
NUC1                   13C
P0                    3.27 usec
P1                    9.80 usec
PLW1           40.00000000 W
SFO2          600.0124004 MHz
NUC2                    1H
CPDPRG[2           waltz16
PCPD2                90.00 usec
PLW2           20.00000000 W
PLW12           0.33800000 W

F2 - Processing parameters
SI                   65536
SF            150.8727007 MHz
WDW                     EM
SSB                      0
LB                    1.00 Hz
GB                       0
PC                    1.40
```

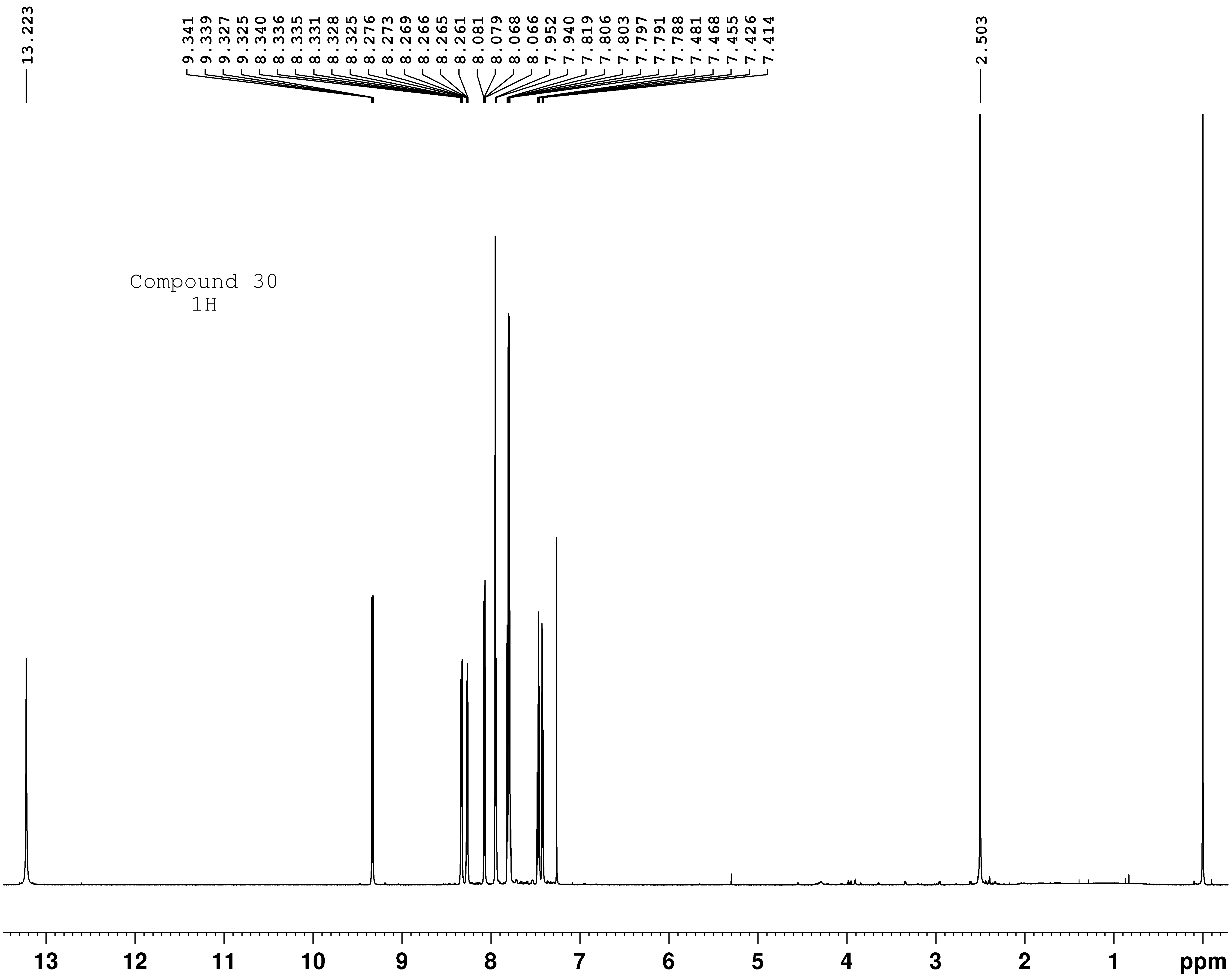

Current Data Parameters

NAME	CD-118A
EXPNO	11
PROCNO	1

F2 - Acquisition Parameters

Date_	20210219
Time	17.14 h
INSTRUM	spect
PROBHD	Z847801_0047 (
PULPROG	zg30
TD	32768
SOLVENT	CDCl3
NS	32
DS	0
SWH	9615.385 Hz
FIDRES	0.586877 Hz
AQ	1.7039360 sec
RG	161
DW	52.000 usec
DE	14.37 usec
TE	293.0 K
D1	1.00000000 sec
TD0	1
SFO1	600.0145608 MHz
NUC1	1H
P0	3.62 usec
P1	10.85 usec
PLW1	20.00000000 W

F2 - Processing parameters

SI	65536
SF	600.0100136 MHz
WDW	EM
SSB	0
LB	0 Hz
GB	0
PC	1.00

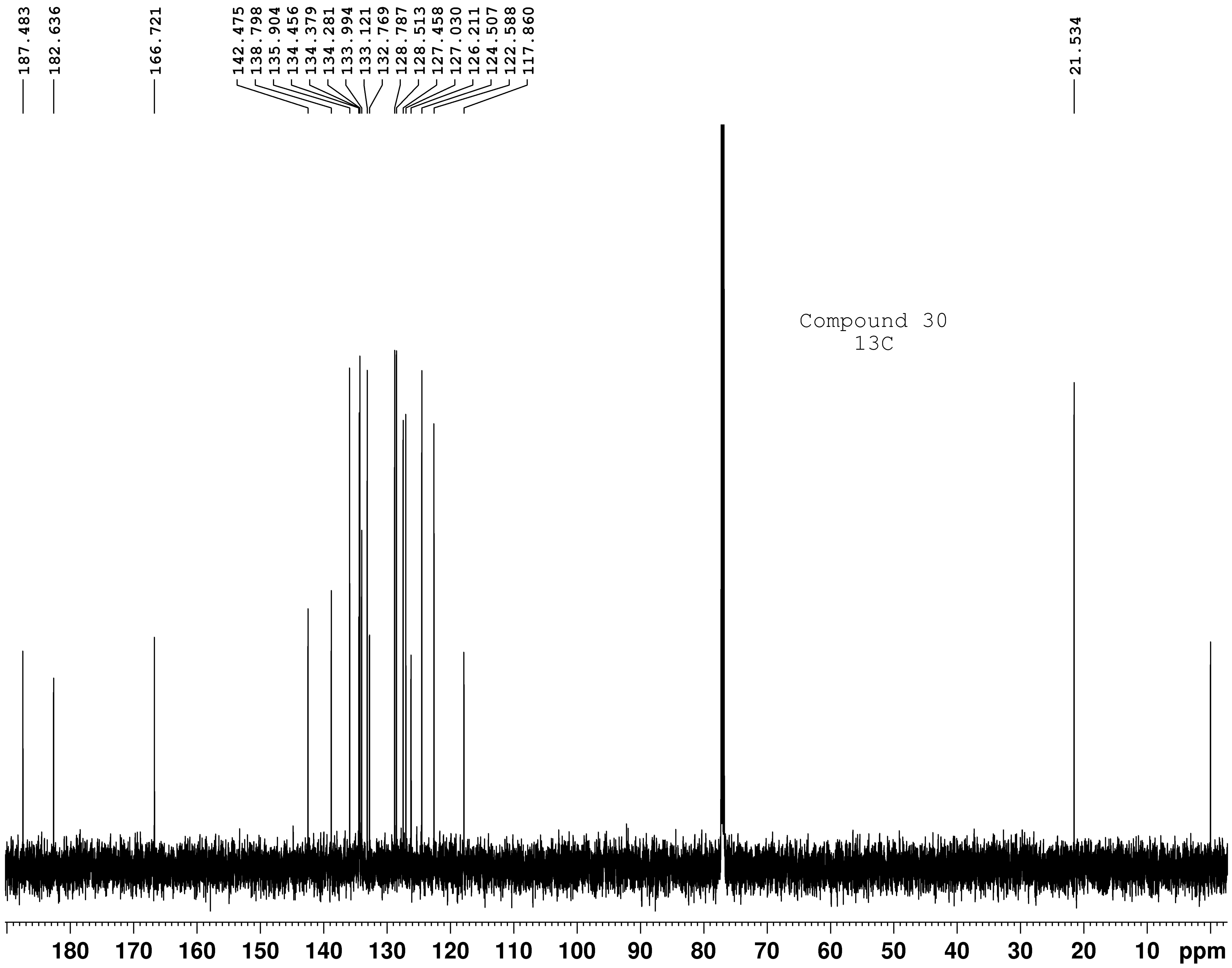


```
Current Data Parameters
NAME              CD-118A
EXPNO                  16
PROCNO                  1

F2 - Acquisition Parameters
Date_            20210219
Time                17.50 h
INSTRUM             spect
PROBHD   Z847801_0047 (
PULPROG           zgdc30
TD                 32768
SOLVENT            CDCl3
NS                   128
DS                     0
SWH            36057.691 Hz
FIDRES          2.200787 Hz
AQ             0.4543829 sec
RG                  2050
DW                13.867 usec
DE                  6.50 usec
TE                 293.0 K
D1            1.50000000 sec
D11           0.03000000 sec
TD0                    1
SFO1         150.8892338 MHz
NUC1                 13C
P0                  3.27 usec
P1                  9.80 usec
PLW1         40.00000000 W
SFO2         600.0124004 MHz
NUC2                  1H
CPDPRG[2         waltz16
PCPD2              90.00 usec
PLW2         20.00000000 W
PLW12         0.33800000 W

F2 - Processing parameters
SI                 65536
SF           150.8726390 MHz
WDW                   EM
SSB                    0
LB                  1.00 Hz
GB                     0
PC                  1.40
```

Compound 31
1H

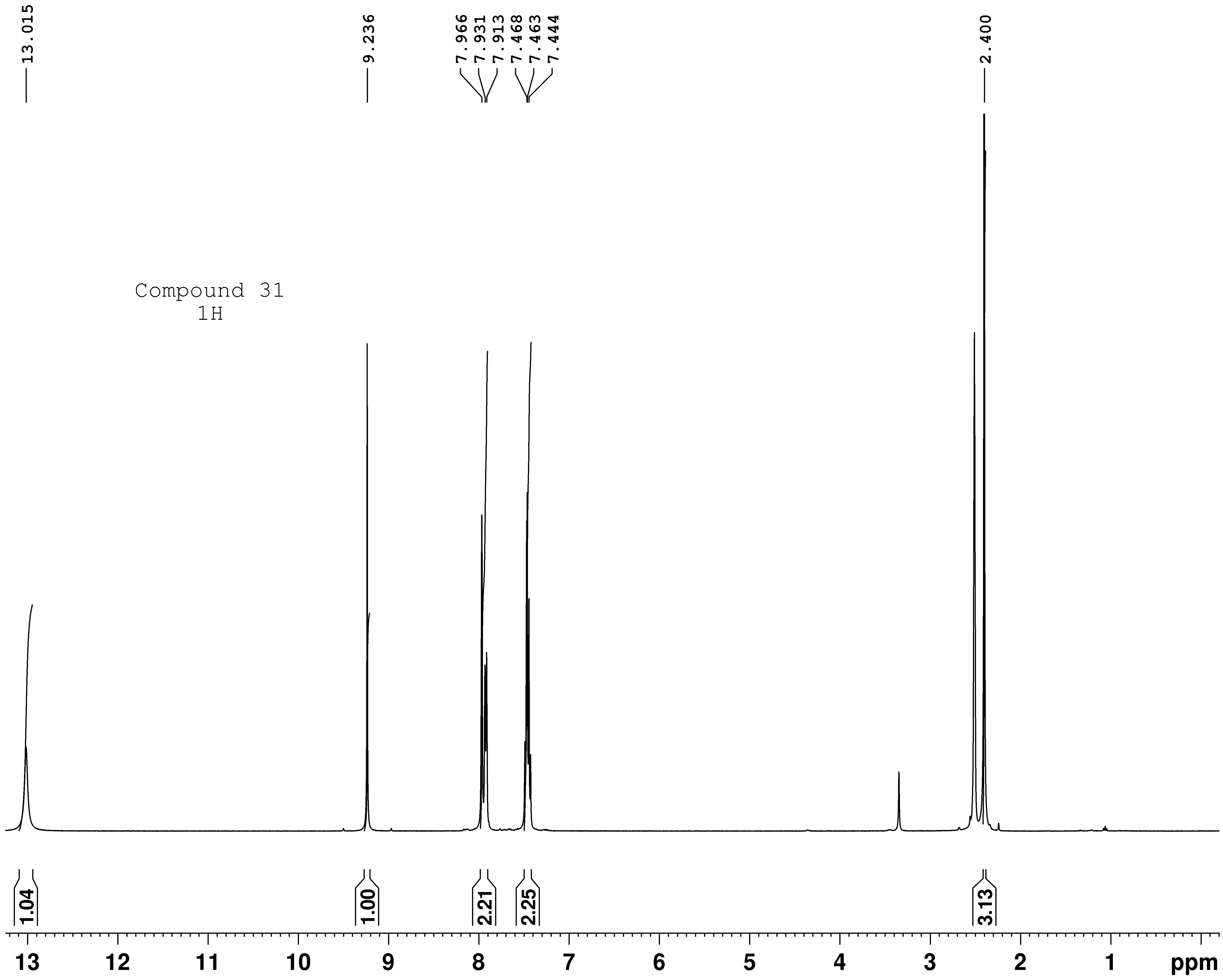

Current Data Parameters
NAME CD-185B
EXPNO 11
PROCNO 1

F2 - Acquisition Parameters
Date_ 20220414
Time 18.13 h
INSTRUM Avance Neo 400
PROBHD Z175272_0007 (
PULPROG zg30
TD 32768
SOLVENT DMSO
NS 32
DS 0
SWH 6578.947 Hz
FIDRES 0.401547 Hz
AQ 2.4903679 sec
RG 101
DW 76.000 usec
DE 7.79 usec
TE 298.0 K
D1 2.00000000 sec
TD0 1
SFO1 400.1330415 MHz
NUC1 1H
P0 3.33 usec
P1 10.00 usec
PLW1 20.07200050 W

F2 - Processing parameters
SI 65536
SF 400.1300000 MHz
WDW EM
SSB 0
LB 0 Hz
GB 0
PC 1.00

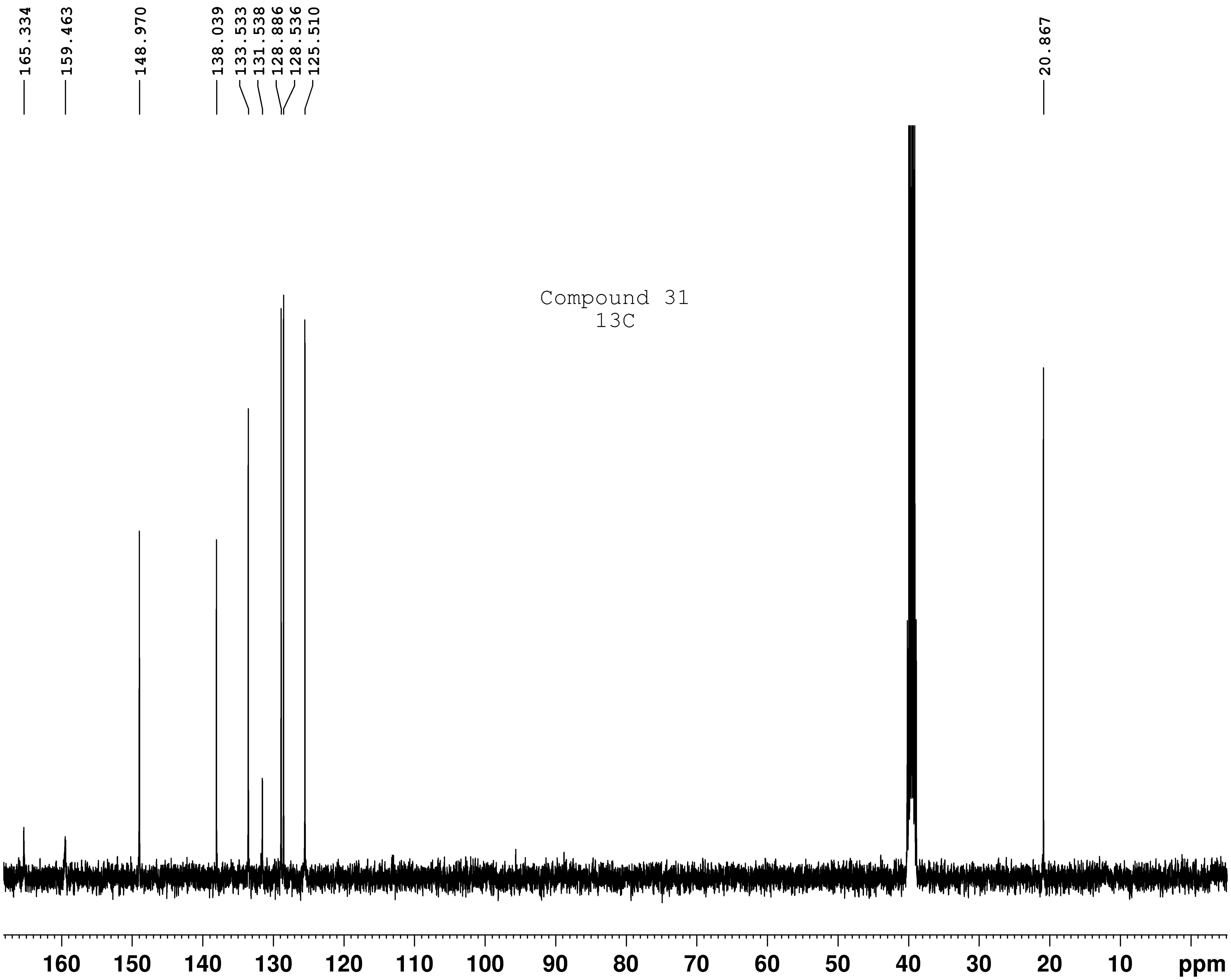

Current Data Parameters

NAME	CD-185B
EXPNO	12
PROCNO	1

F2 - Acquisition Parameters

Date_	20220414
Time	18.18 h
INSTRUM	Avance Neo 400
PROBHD	Z175272_0007 (
PULPROG	zgdc30
TD	32768
SOLVENT	DMSO
NS	128
DS	0
SWH	23809.523 Hz
FIDRES	1.453218 Hz
AQ	0.6881280 sec
RG	11.3
DW	21.000 usec
DE	6.50 usec
TE	298.0 K
D1	1.50000000 sec
D11	0.03000000 sec
TD0	1
SFO1	100.6238383 MHz
NUC1	13C
P0	3.33 usec
P1	10.00 usec
PLW1	58.46900177 W
SFO2	400.1316008 MHz
NUC2	1H
CPDPRG[2	waltz16
PCPD2	90.00 usec
PLW2	20.07200050 W
PLW12	0.24781001 W

F2 - Processing parameters

SI	65536
SF	100.6128162 MHz
WDW	EM
SSB	0
LB	1.00 Hz
GB	0
PC	1.40

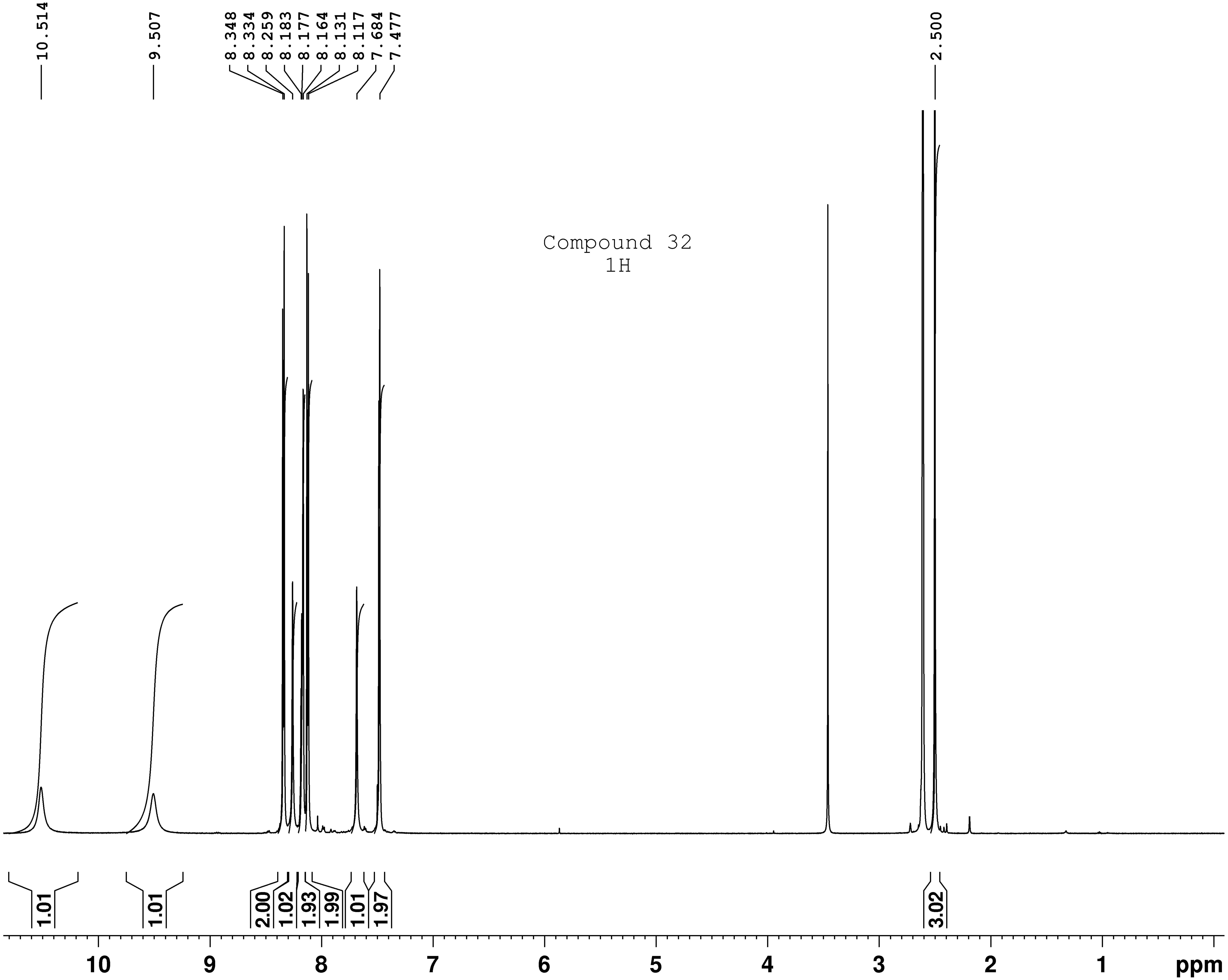

Current Data Parameters
NAME CD-112-A
EXPNO 11
PROCNO 1

F2 - Acquisition Parameters
Date_ 20210316
Time 19.23 h
INSTRUM spect
PROBHD Z847801_0047 (
PULPROG zg30
TD 32768
SOLVENT DMSO
NS 32
DS 0
SWH 9615.385 Hz
FIDRES 0.586877 Hz
AQ 1.7039360 sec
RG 161
DW 52.000 usec
DE 14.37 usec
TE 293.0 K
D1 1.00000000 sec
TD0 1
SFO1 600.0145608 MHz
NUC1 1H
P0 3.62 usec
P1 10.85 usec
PLW1 20.00000000 W

F2 - Processing parameters
SI 65536
SF 600.0099403 MHz
WDW EM
SSB 0
LB 0 Hz
GB 0
PC 1.00

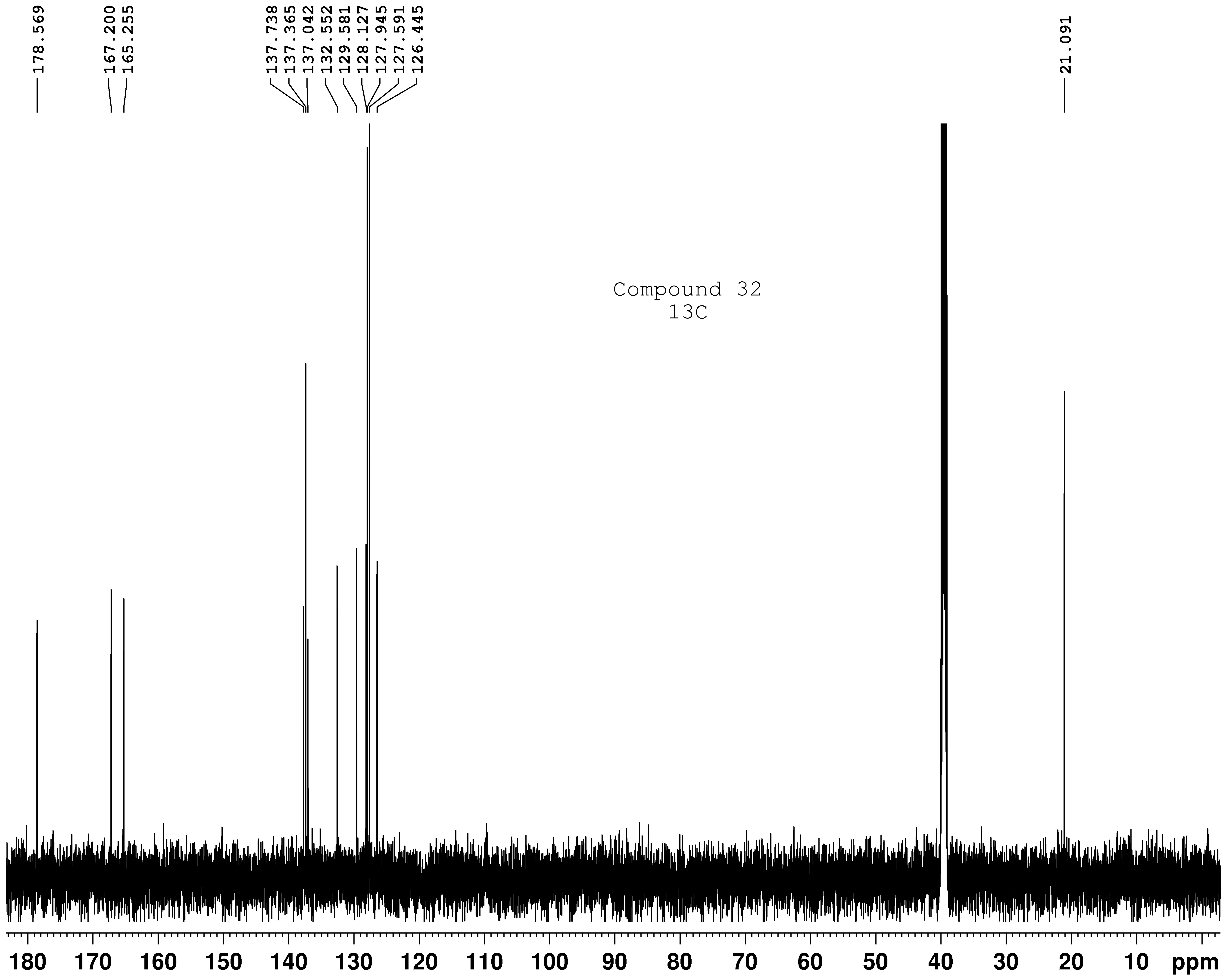

Current Data Parameters

NAME	CD-112-A
EXPNO	16
PROCNO	1

F2 - Acquisition Parameters

Date_	20210316
Time	19.58 h
INSTRUM	spect
PROBHD	Z847801_0047 (
PULPROG	zgdc30
TD	32768
SOLVENT	DMSO
NS	128
DS	0
SWH	36057.691 Hz
FIDRES	2.200787 Hz
AQ	0.4543829 sec
RG	2050
DW	13.867 usec
DE	6.50 usec
TE	293.0 K
D1	1.50000000 sec
D11	0.03000000 sec
TD0	1
SFO1	150.8892338 MHz
NUC1	13C
P0	3.27 usec
P1	9.80 usec
PLW1	40.00000000 W
SFO2	600.0124004 MHz
NUC2	1H
CPDPRG[2	waltz16
PCPD2	90.00 usec
PLW2	20.00000000 W
PLW12	0.33800000 W

F2 - Processing parameters

SI	65536
SF	150.8727009 MHz
WDW	EM
SSB	0
LB	1.00 Hz
GB	0
PC	1.40

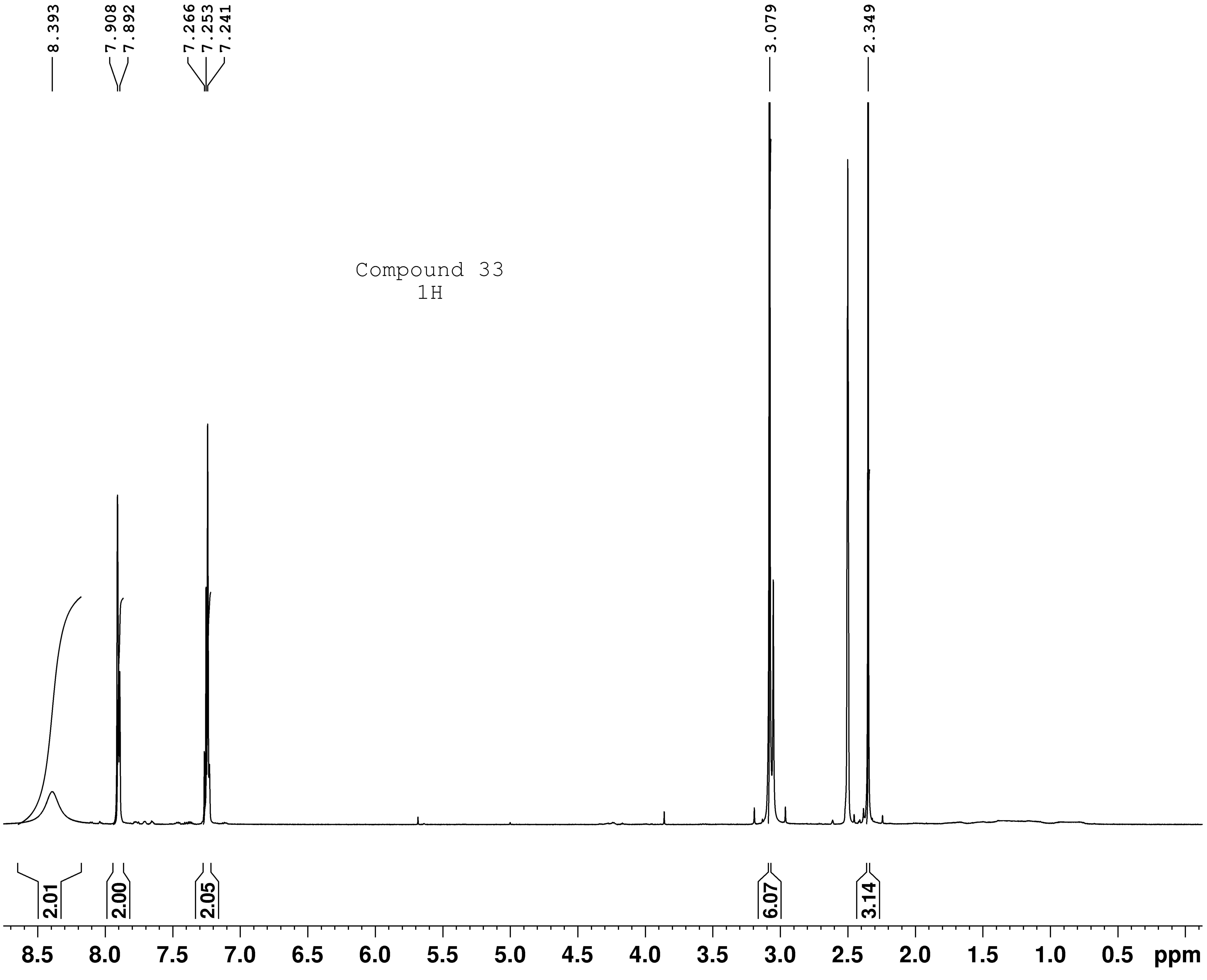


```
Current Data Parameters
NAME             CD-163A
EXPNO                 11
PROCNO                 1

F2 - Acquisition Parameters
Date_           20220225
Time               21.00 h
INSTRUM            spect
PROBHD   Z847801_0047 (
PULPROG             zg30
TD                 32768
SOLVENT             DMSO
NS                    32
DS                     0
SWH             9615.385 Hz
FIDRES          0.586877 Hz
AQ             1.7039360 sec
RG                   181
DW                52.000 usec
DE                 14.38 usec
TE                 353.1 K
D1            1.00000000 sec
TD0                    1
SFO1         599.9845606 MHz
NUC1                  1H
P0                  3.60 usec
P1                 10.80 usec
PLW1         20.00000000 W

F2 - Processing parameters
SI                 65536
SF           599.9800045 MHz
WDW                   EM
SSB                    0
LB                     0 Hz
GB                     0
PC                  1.00
```

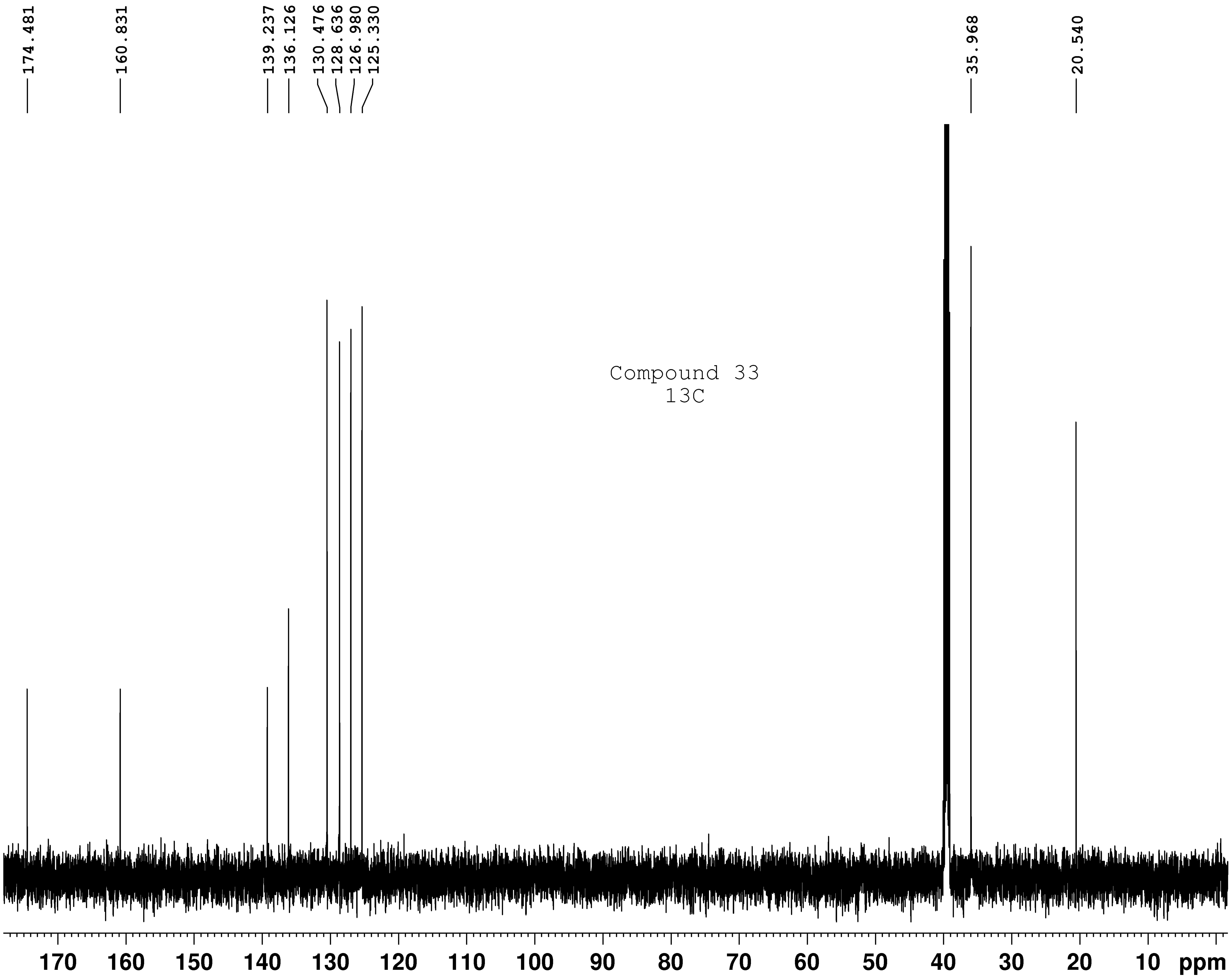


```
Current Data Parameters
NAME             CD-163A
EXPNO                 12
PROCNO                 1

F2 - Acquisition Parameters
Date_           20220225
Time               21.06 h
INSTRUM            spect
PROBHD   Z847801_0047 (
PULPROG          zgdc30
TD                 32768
SOLVENT             DMSO
NS                   128
DS                     0
SWH            36057.691 Hz
FIDRES          2.200787 Hz
AQ             0.4543829 sec
RG                  2050
DW                13.867 usec
DE                  6.50 usec
TE                 353.1 K
D1            1.50000000 sec
D11           0.03000000 sec
TD0                    1
SFO1         150.8816889 MHz
NUC1                 13C
P0                  3.27 usec
P1                  9.80 usec
PLW1         40.00000000 W
SFO2         599.9824003 MHz
NUC2                  1H
CPDPRG[2         waltz16
PCPD2              70.00 usec
PLW2         20.00000000 W
PLW12         0.53044999 W

F2 - Processing parameters
SI                 65536
SF           150.8652273 MHz
WDW                   EM
SSB                    0
LB                  1.00 Hz
GB                     0
PC                  1.40
```

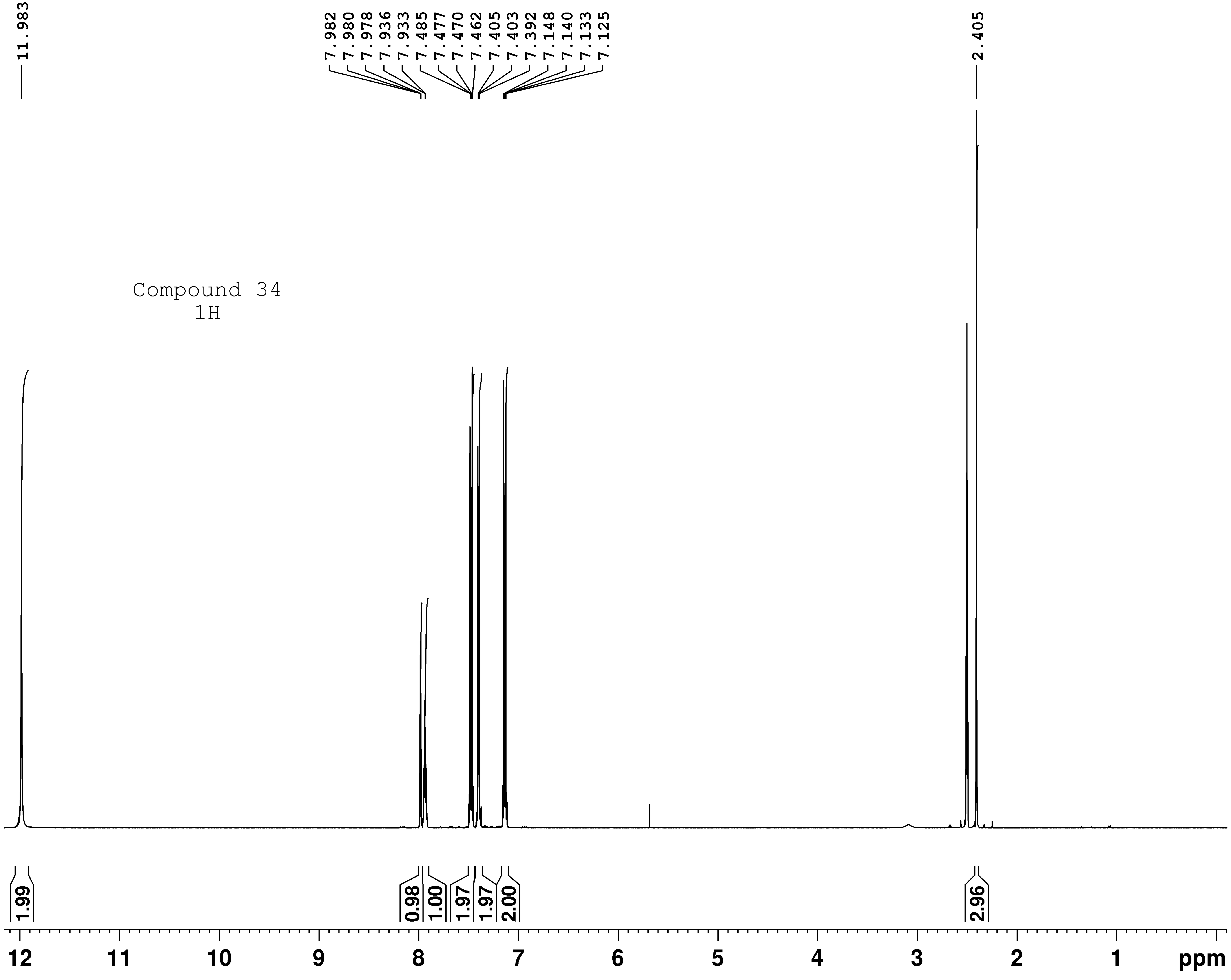


```
Current Data Parameters
NAME              CD-102B
EXPNO                  11
PROCNO                  1

F2 - Acquisition Parameters
Date_            20220416
Time                10.51 h
INSTRUM   Avance Neo 400
PROBHD    Z175272_0007 (
PULPROG              zg30
TD                  32768
SOLVENT              DMSO
NS                     32
DS                      0
SWH              6578.947 Hz
FIDRES           0.401547 Hz
AQ              2.4903679 sec
RG                    101
DW                 76.000 usec
DE                   7.79 usec
TE                  353.0 K
D1             2.00000000 sec
TD0                     1
SFO1          400.1330415 MHz
NUC1                   1H
P0                   3.33 usec
P1                  10.00 usec
PLW1          20.07200050 W

F2 - Processing parameters
SI                  65536
SF            400.1300037 MHz
WDW                    EM
SSB                     0
LB                      0 Hz
GB                      0
PC                   1.00
```

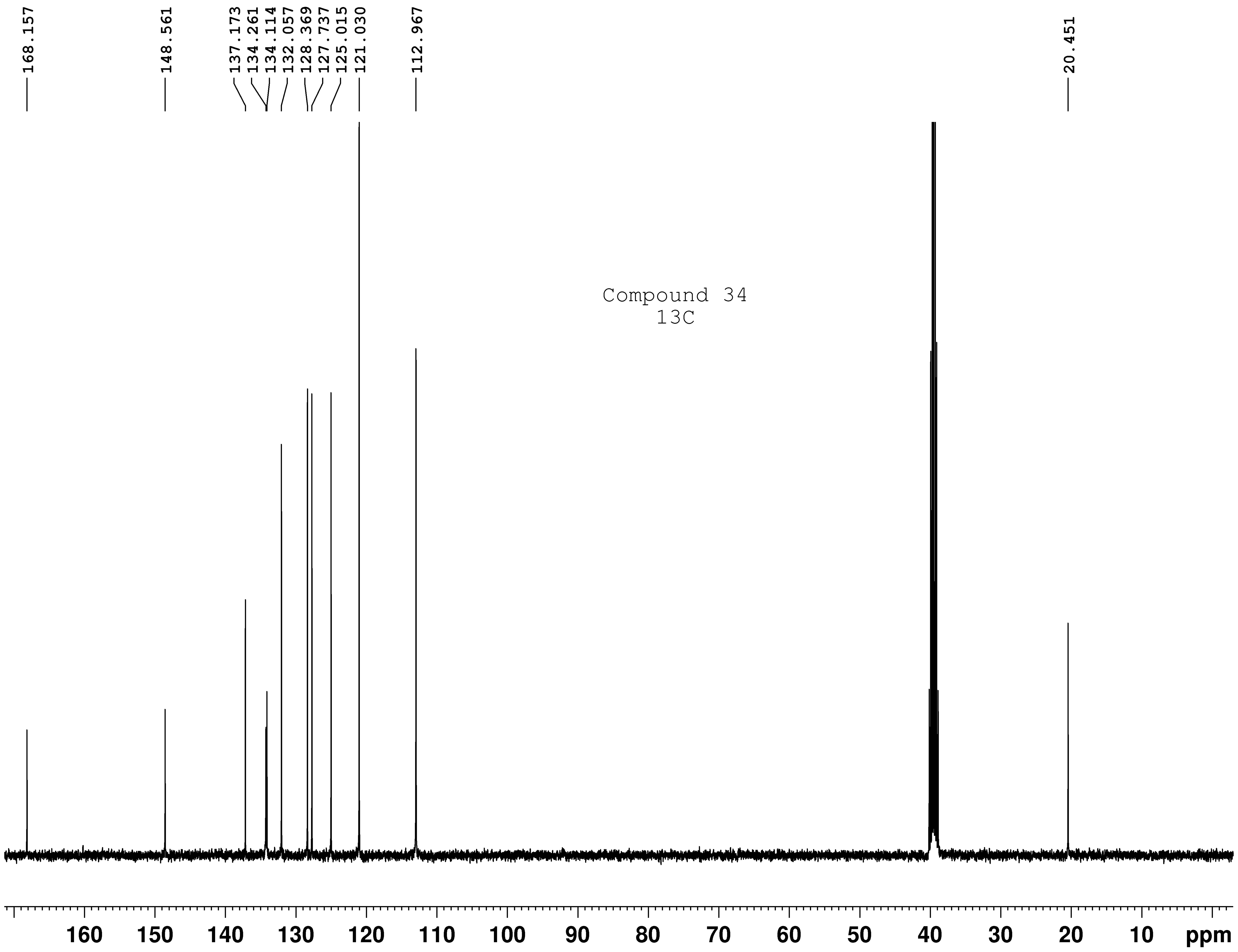

Current Data Parameters
NAME CD-102B
EXPNO 12
PROCNO 1

F2 - Acquisition Parameters
Date_ 20220416
Time 11.30 h
INSTRUM Avance Neo 400
PROBHD Z175272_0007 (
PULPROG zgdc30
TD 32768
SOLVENT DMSO
NS 1024
DS 0
SWH 23809.523 Hz
FIDRES 1.453218 Hz
AQ 0.6881280 sec
RG 45.2
DW 21.000 usec
DE 6.50 usec
TE 353.0 K
D1 1.50000000 sec
D11 0.03000000 sec
TD0 1
SFO1 100.6238383 MHz
NUC1 13C
P0 3.33 usec
P1 10.00 usec
PLW1 58.46900177 W
SFO2 400.1316008 MHz
NUC2 1H
CPDPRG[2 waltz16
PCPD2 90.00 usec
PLW2 20.07200050 W
PLW12 0.24781001 W

F2 - Processing parameters
SI 65536
SF 100.6128564 MHz
WDW EM
SSB 0
LB 1.00 Hz
GB 0
PC 1.40

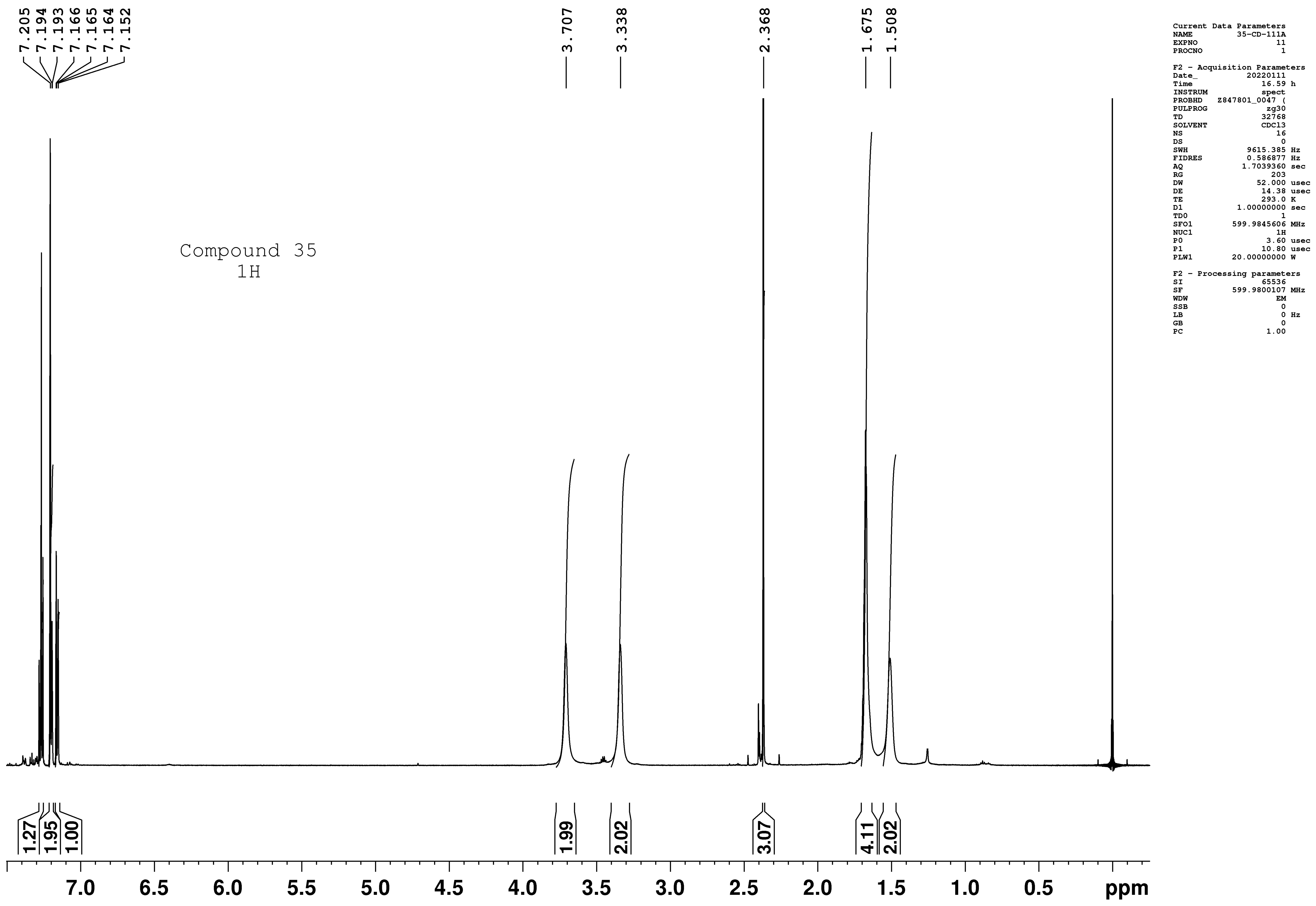

7.205
7.194
7.193
7.166
7.165
7.164
7.152
3.707
3.338
2.368
1.675
1.508
Compound 35
1H
1.27
1.95
1.00
1.99
2.02
3.07
4.11
2.02
7.0
6.5
6.0
5.5
5.0
4.5
4.0
3.5
3.0
2.5
2.0
1.5
1.0
0.5
ppm
Current Data Parameters
NAME 35-CD-111A
EXPNO 11
PROCNO 1
F2 - Acquisition Parameters
Date_ 20220111
Time 16.59 h
INSTRUM spect
PROBHD Z847801_0047 (
PULPROG zg30
TD 32768
SOLVENT CDCl3
NS 16
DS 0
SWH 9615.385 Hz
FIDRES 0.586877 Hz
AQ 1.7039360 sec
RG 203
DW 52.000 usec
DE 14.38 usec
TE 293.0 K
D1 1.00000000 sec
TD0 1
SFO1 599.9845606 MHz
NUC1 1H
P0 3.60 usec
P1 10.80 usec
PLW1 20.00000000 W
F2 - Processing parameters
SI 65536
SF 599.9800107 MHz
WDW EM
SSB 0
LB 0 Hz
GB 0
PC 1.00

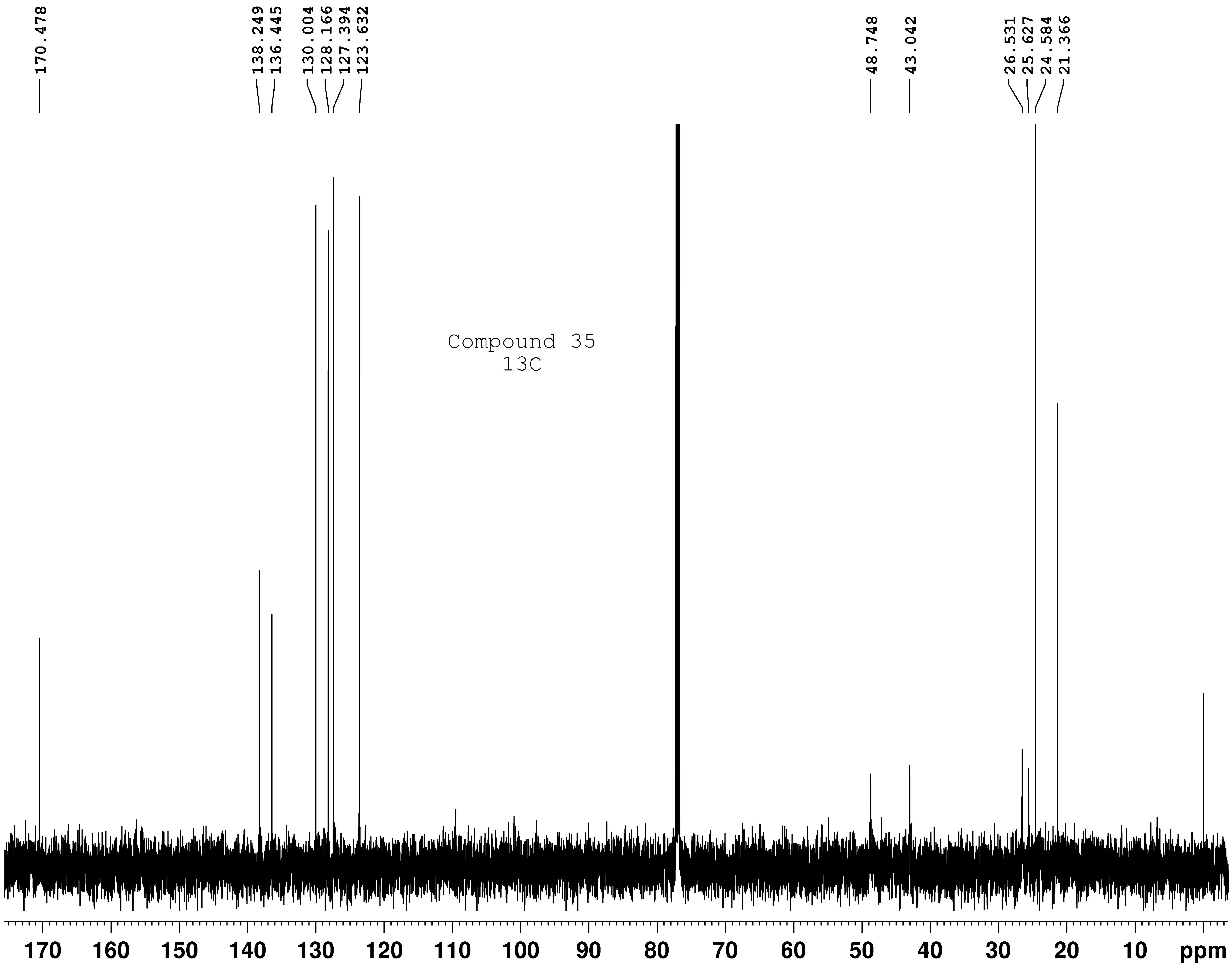

Current Data Parameters

NAME	CD-111A	
EXPNO	12	
PROCNO	1	

F2 - Acquisition Parameters

Date_	20220111	
Time	17.04	h
INSTRUM	spect	
PROBHD	Z847801_0047 (	
PULPROG	zgdc30	
TD	32768	
SOLVENT	CDCl3	
NS	128	
DS	0	
SWH	36057.691	Hz
FIDRES	2.200787	Hz
AQ	0.4543829	sec
RG	2050	
DW	13.867	usec
DE	6.50	usec
TE	293.0	K
D1	1.50000000	sec
D11	0.03000000	sec
TD0	1	
SFO1	150.8816889	MHz
NUC1	13C	
P0	3.27	usec
P1	9.80	usec
PLW1	40.00000000	W
SFO2	599.9824003	MHz
NUC2	1H	
CPDPRG[2	waltz16	
PCPD2	70.00	usec
PLW2	20.00000000	W
PLW12	0.53044999	W

F2 - Processing parameters

SI	65536	
SF	150.8650981	MHz
WDW	EM	
SSB	0	
LB	1.00	Hz
GB	0	
PC	1.40	

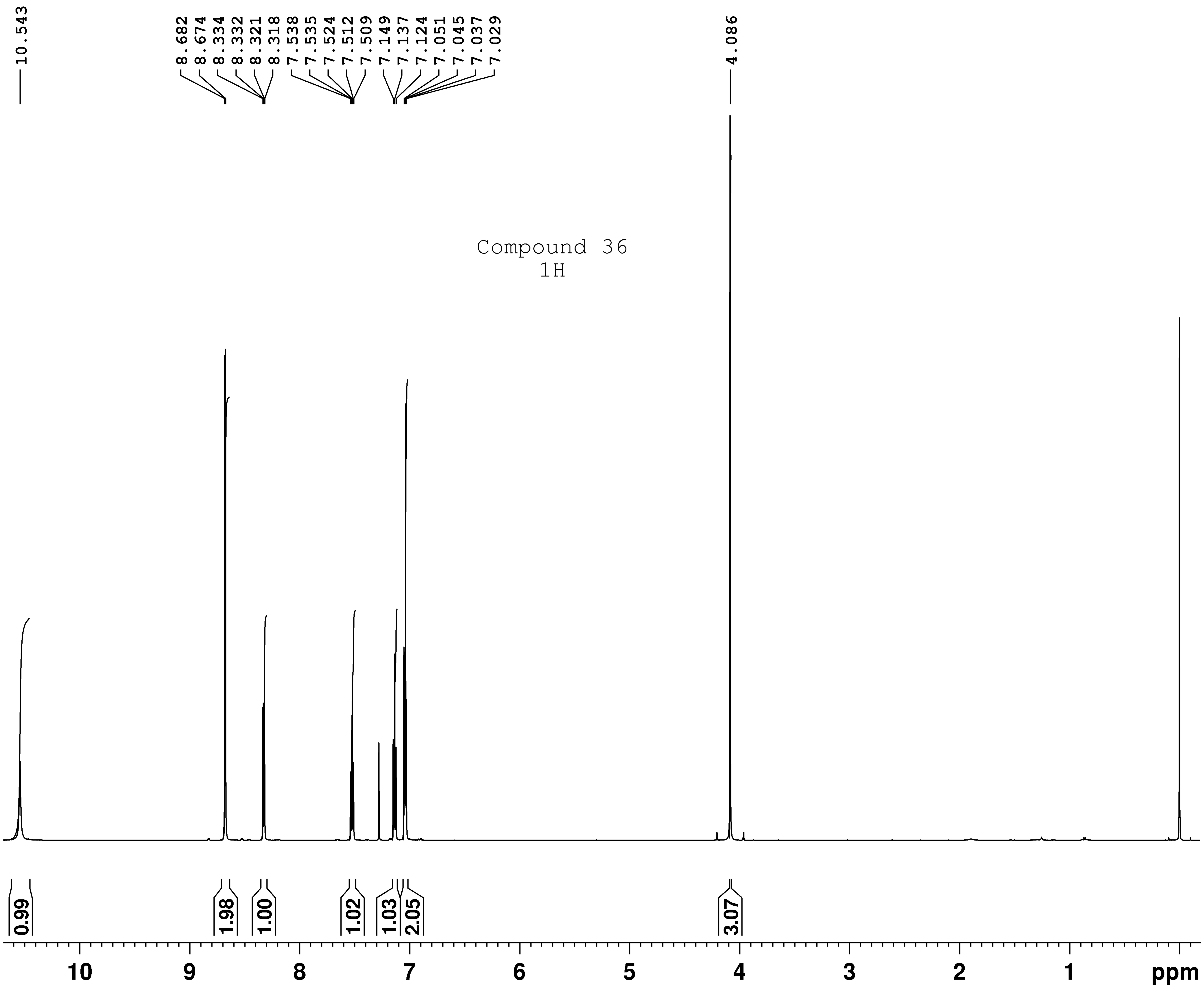

Current Data Parameters
NAME CD-62A
EXPNO 11
PROCNO 1

F2 - Acquisition Parameters
Date_ 20210217
Time 17.48 h
INSTRUM spect
PROBHD Z847801_0047 (
PULPROG zg30
TD 32768
SOLVENT CDCl3
NS 32
DS 0
SWH 9615.385 Hz
FIDRES 0.586877 Hz
AQ 1.7039360 sec
RG 161
DW 52.000 usec
DE 14.37 usec
TE 293.0 K
D1 1.00000000 sec
TD0 1
SFO1 600.0145608 MHz
NUC1 1H
P0 3.62 usec
P1 10.85 usec
PLW1 20.00000000 W

F2 - Processing parameters
SI 65536
SF 600.0100031 MHz
WDW EM
SSB 0
LB 0 Hz
GB 0
PC 1.00

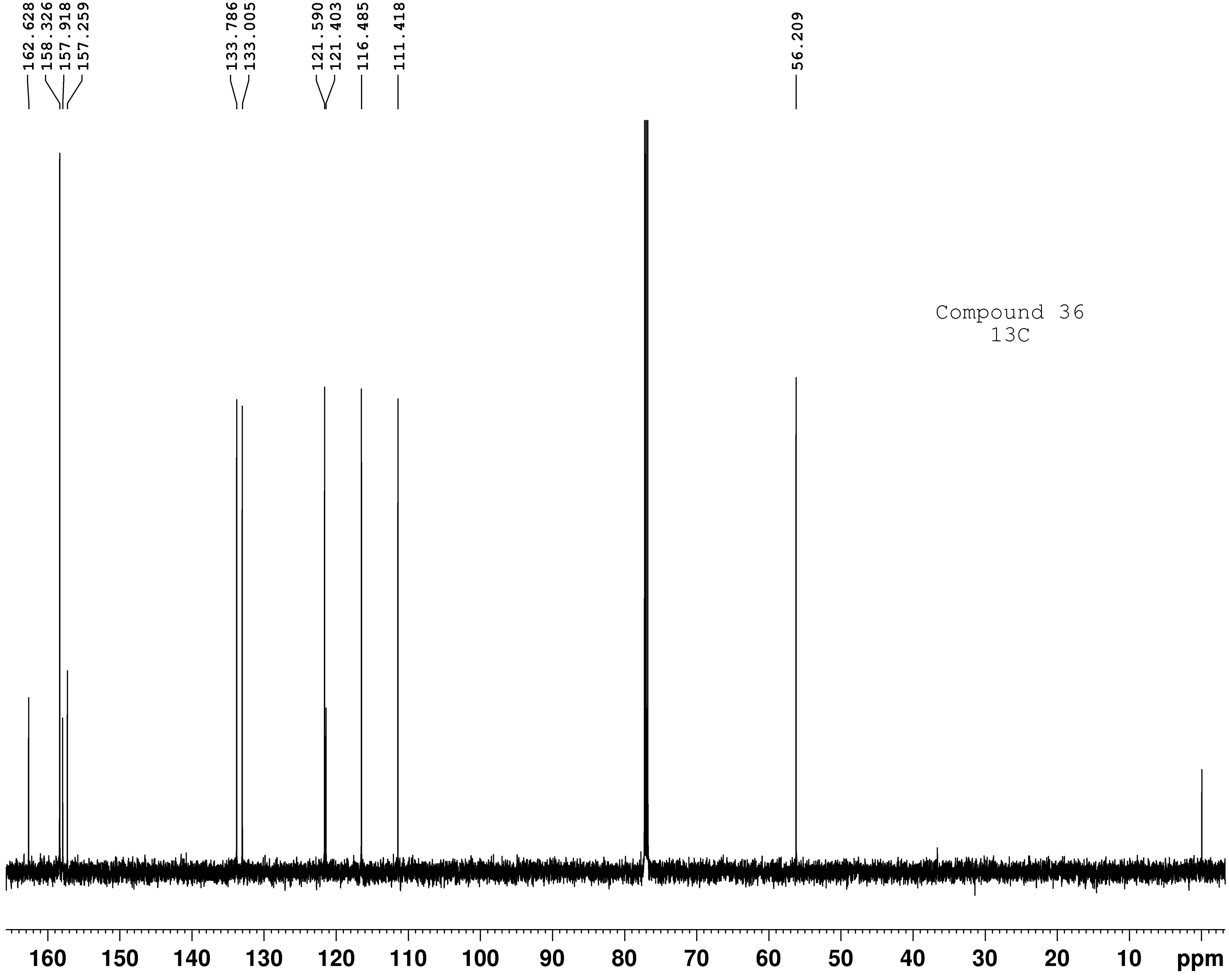


```
Current Data Parameters
NAME              CD-62A
EXPNO                 16
PROCNO                 1

F2 - Acquisition Parameters
Date_           20210217
Time               18.22 h
INSTRUM            spect
PROBHD   Z847801_0047 (
PULPROG          zgdc30
TD                 32768
SOLVENT            CDCl3
NS                   128
DS                     0
SWH            36057.691 Hz
FIDRES          2.200787 Hz
AQ             0.4543829 sec
RG                  2050
DW                13.867 usec
DE                  6.50 usec
TE                 293.0 K
D1            1.50000000 sec
D11           0.03000000 sec
TD0                    1
SFO1         150.8892338 MHz
NUC1                 13C
P0                  3.27 usec
P1                  9.80 usec
PLW1         40.00000000 W
SFO2         600.0124004 MHz
NUC2                  1H
CPDPRG[2         waltz16
PCPD2              90.00 usec
PLW2         20.00000000 W
PLW12         0.33800000 W

F2 - Processing parameters
SI                 65536
SF           150.8726472 MHz
WDW                   EM
SSB                    0
LB                  1.00 Hz
GB                     0
PC                  1.40
```

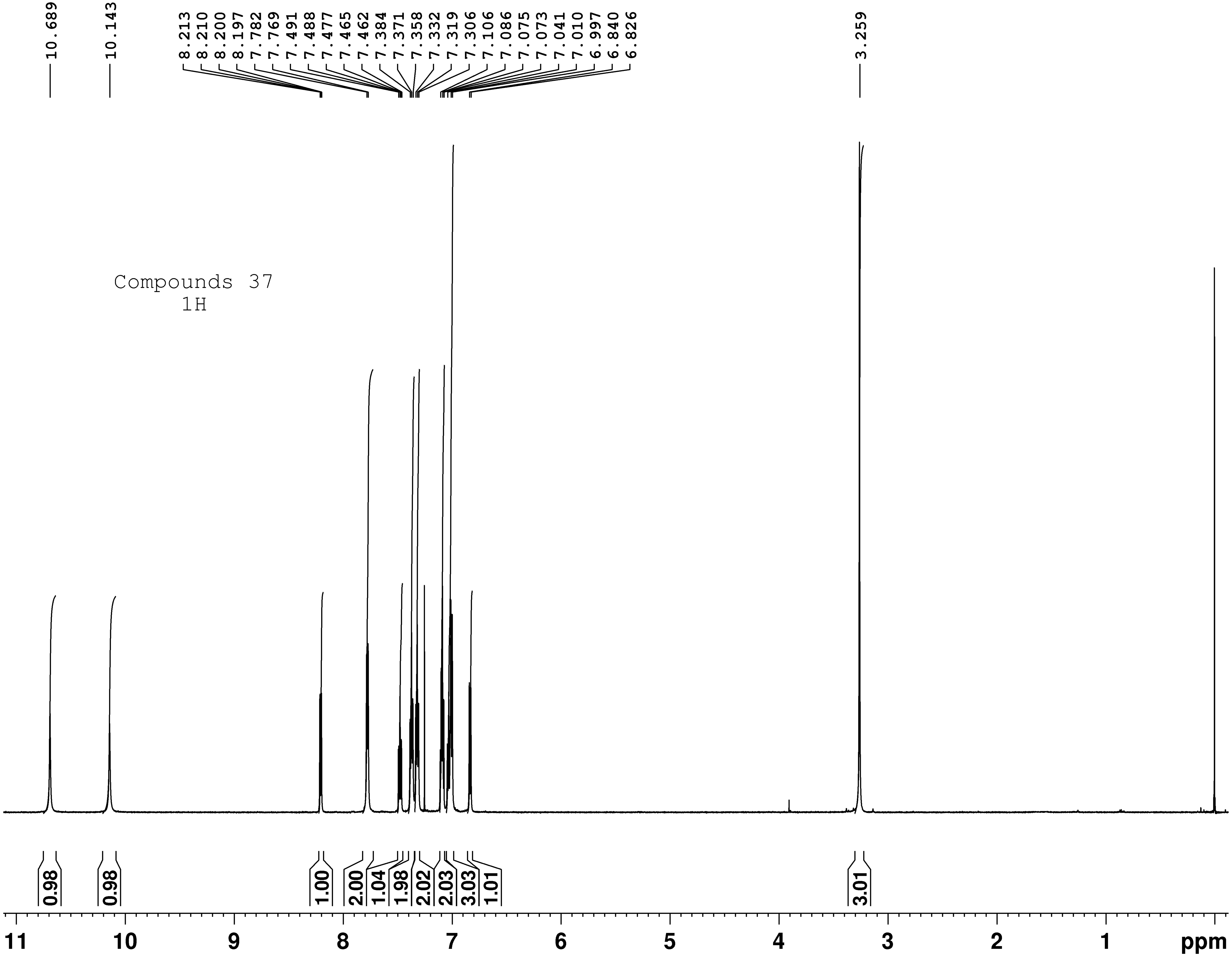

Current Data Parameters
NAME CD-63A
EXPNO 10
PROCNO 1

F2 - Acquisition Parameters
Date_ 20191111
Time 8.55 h
INSTRUM spect
PROBHD Z847801_0047 (
PULPROG zg30
TD 32768
SOLVENT CDCl3
NS 1
DS 0
SWH 9615.385 Hz
FIDRES 0.586877 Hz
AQ 1.7039360 sec
RG 161
DW 52.000 usec
DE 13.95 usec
TE 293.0 K
D1 1.00000000 sec
TD0 1
SFO1 600.0145608 MHz
NUC1 1H
P1 10.85 usec
PLW1 20.00000000 W

F2 - Processing parameters
SI 65536
SF 600.0100204 MHz
WDW EM
SSB 0
LB 0 Hz
GB 0
PC 1.00

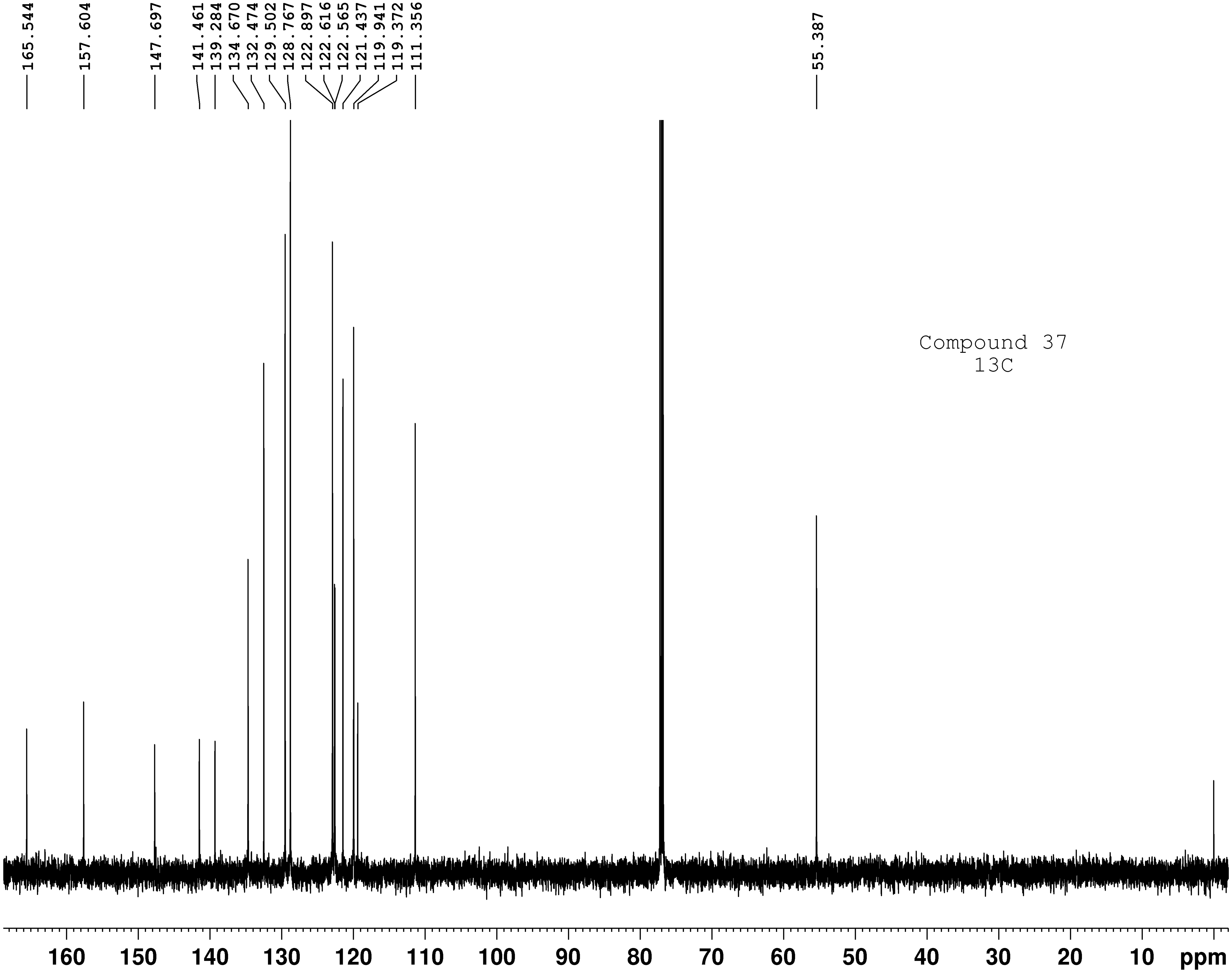

Current Data Parameters

NAME	CD-63A	
EXPNO	16	
PROCNO	1	

F2 - Acquisition Parameters

Date_	20191111	
Time	9.38	h
INSTRUM	spect	
PROBHD	Z847801_0047 (	
PULPROG	zgdc30	
TD	32768	
SOLVENT	CDCl3	
NS	256	
DS	0	
SWH	36057.691	Hz
FIDRES	2.200787	Hz
AQ	0.4543829	sec
RG	2050	
DW	13.867	usec
DE	6.50	usec
TE	293.0	K
D1	1.50000000	sec
D11	0.03000000	sec
TD0	1	
SFO1	150.8892338	MHz
NUC1	13C	
P1	9.80	usec
PLW1	40.00000000	W
SFO2	600.0124004	MHz
NUC2	1H	
CPDPRG[2	waltz16	
PCPD2	90.00	usec
PLW2	20.00000000	W
PLW12	0.33800000	W

F2 - Processing parameters

SI	65536	
SF	150.8726444	MHz
WDW	EM	
SSB	0	
LB	1.00	Hz
GB	0	
PC	1.40	

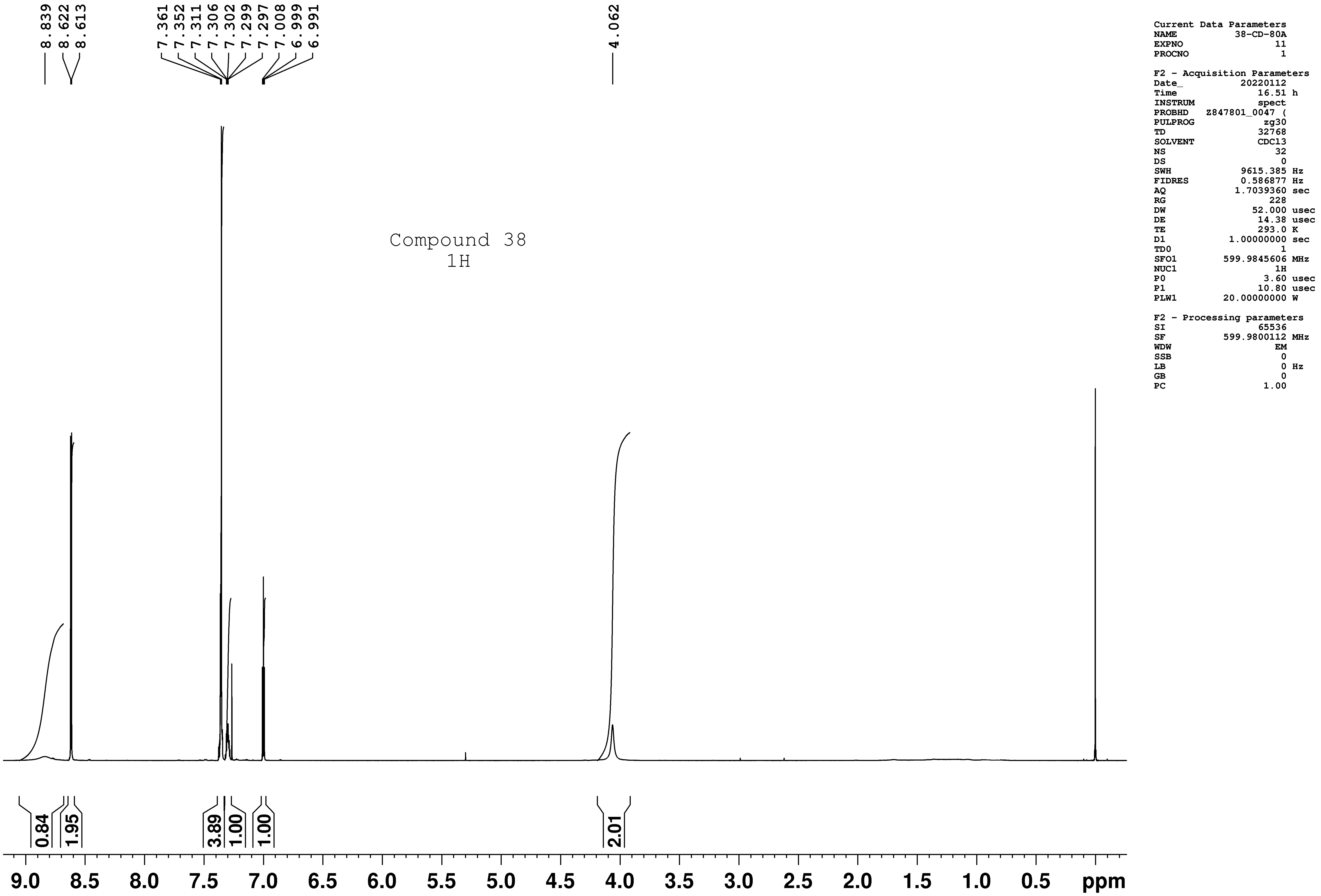
8.839
8.622
8.613
7.361
7.352
7.311
7.306
7.302
7.299
7.297
7.008
6.999
6.991
4.062
Compound 38
1H
0.84
1.95
3.89
1.00
1.00
2.01
9.0 8.5 8.0 7.5 7.0 6.5 6.0 5.5 5.0 4.5 4.0 3.5 3.0 2.5 2.0 1.5 1.0 0.5 ppm
Current Data Parameters
NAME 38-CD-80A
EXPNO 11
PROCNO 1
F2 - Acquisition Parameters
Date_ 20220112
Time 16.51 h
INSTRUM spect
PROBHD Z847801_0047 (
PULPROG zg30
TD 32768
SOLVENT CDCl3
NS 32
DS 0
SWH 9615.385 Hz
FIDRES 0.586877 Hz
AQ 1.7039360 sec
RG 228
DW 52.000 usec
DE 14.38 usec
TE 293.0 K
D1 1.00000000 sec
TD0 1
SFO1 599.9845606 MHz
NUC1 1H
P0 3.60 usec
P1 10.80 usec
PLW1 20.00000000 W
F2 - Processing parameters
SI 65536
SF 599.9800112 MHz
WDW EM
SSB 0
LB 0 Hz
GB 0
PC 1.00

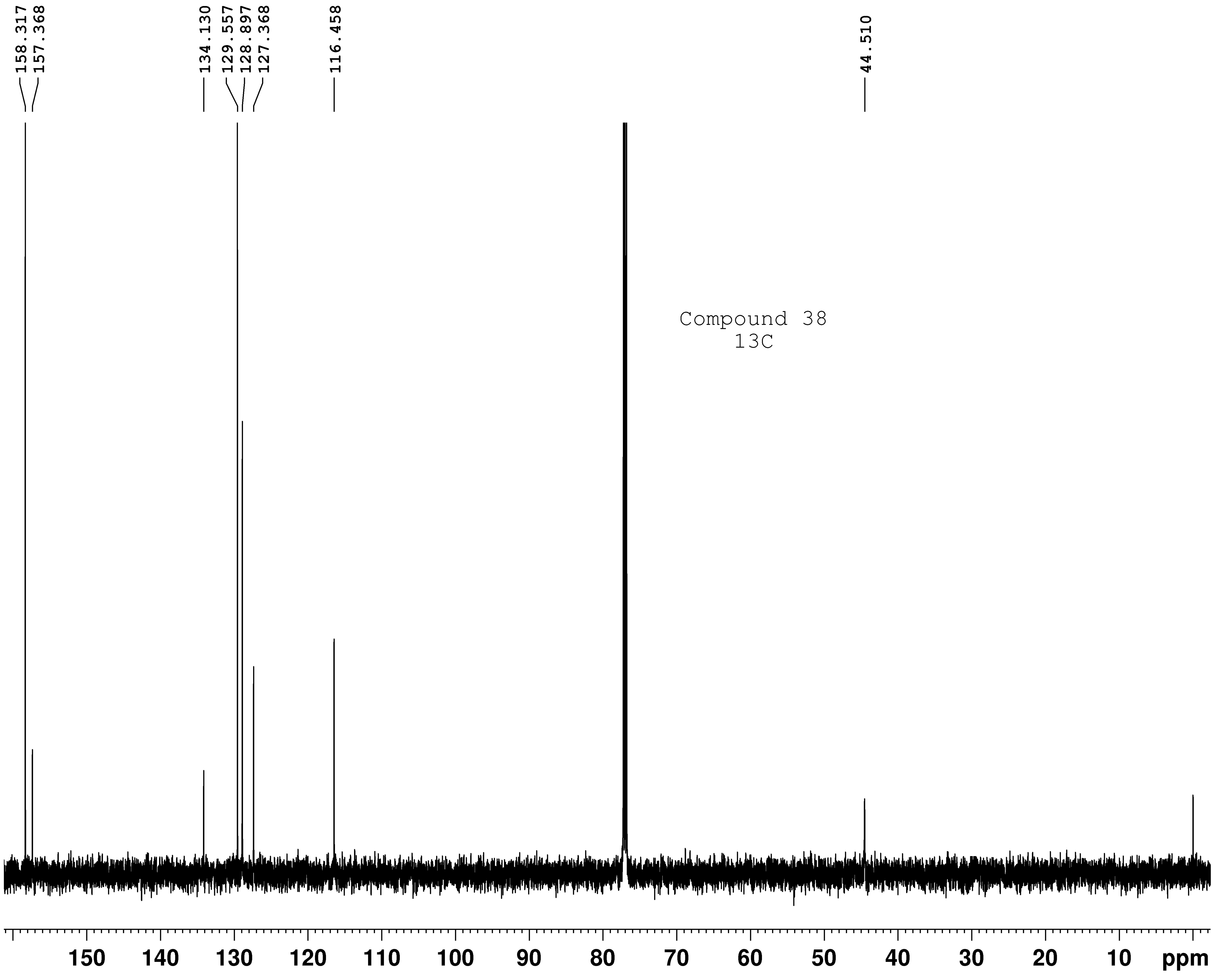

Current Data Parameters
NAME CD-80A
EXPNO 12
PROCNO 1

F2 - Acquisition Parameters
Date_ 20220112
Time 16.56 h
INSTRUM spect
PROBHD Z847801_0047 (
PULPROG zgdc30
TD 32768
SOLVENT CDCl3
NS 128
DS 0
SWH 36057.691 Hz
FIDRES 2.200787 Hz
AQ 0.4543829 sec
RG 2050
DW 13.867 usec
DE 6.50 usec
TE 293.0 K
D1 1.50000000 sec
D11 0.03000000 sec
TD0 1
SFO1 150.8816889 MHz
NUC1 13C
P0 3.27 usec
P1 9.80 usec
PLW1 40.00000000 W
SFO2 599.9824003 MHz
NUC2 1H
CPDPRG[2 waltz16
PCPD2 70.00 usec
PLW2 20.00000000 W
PLW12 0.53044999 W

F2 - Processing parameters
SI 65536
SF 150.8650999 MHz
WDW EM
SSB 0
LB 1.00 Hz
GB 0
PC 1.40

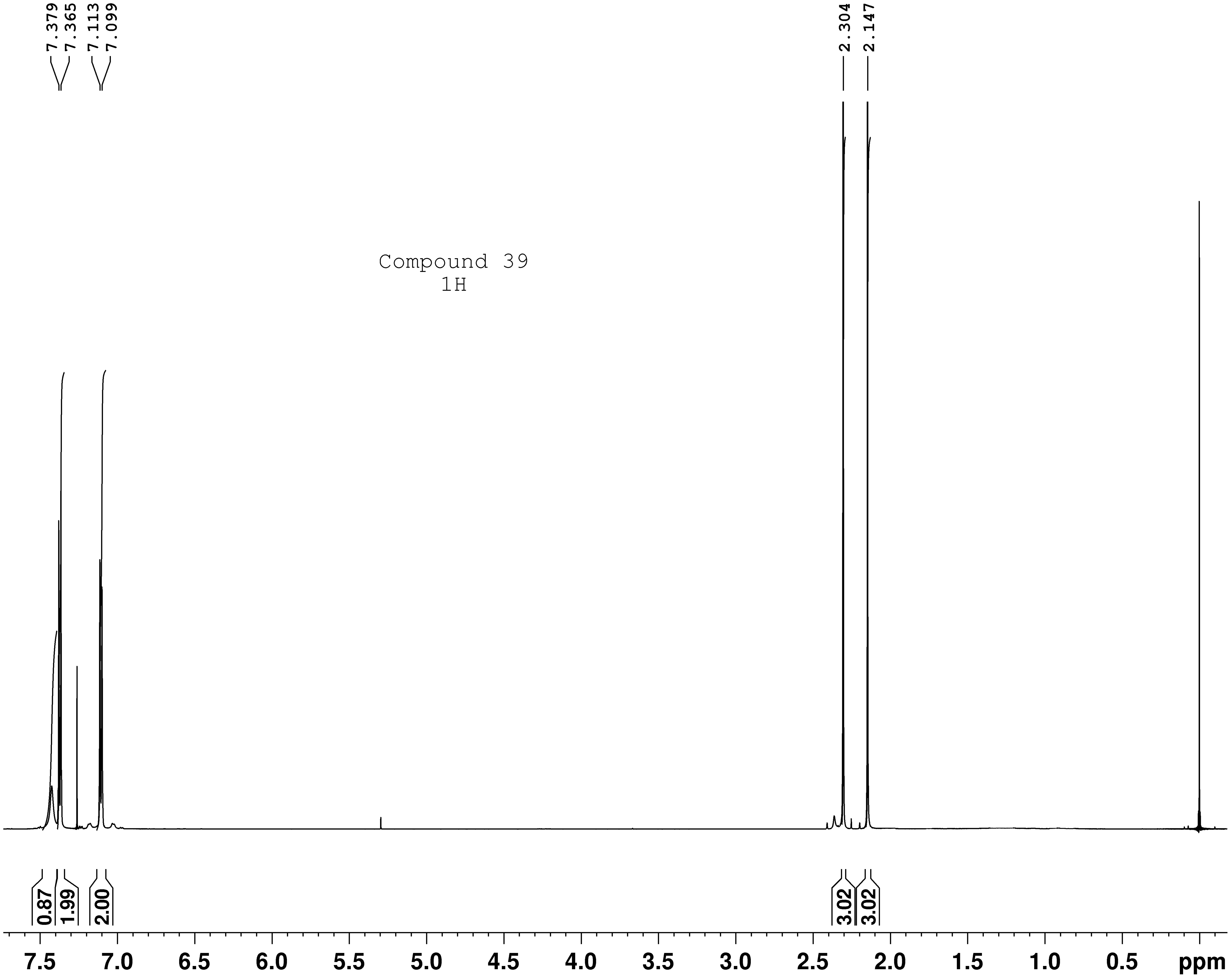


```
Current Data Parameters
NAME          39-CD-125A
EXPNO                 11
PROCNO                 1

F2 - Acquisition Parameters
Date_           20220112
Time               17.07 h
INSTRUM            spect
PROBHD   Z847801_0047 (
PULPROG             zg30
TD                 32768
SOLVENT            CDCl3
NS                    32
DS                     0
SWH             9615.385 Hz
FIDRES          0.586877 Hz
AQ             1.7039360 sec
RG                   161
DW                52.000 usec
DE                 14.38 usec
TE                 293.0 K
D1            1.00000000 sec
TD0                    1
SFO1         599.9845606 MHz
NUC1                  1H
P0                  3.60 usec
P1                 10.80 usec
PLW1         20.00000000 W

F2 - Processing parameters
SI                 65536
SF           599.9800136 MHz
WDW                   EM
SSB                    0
LB                     0 Hz
GB                     0
PC                  1.00
```

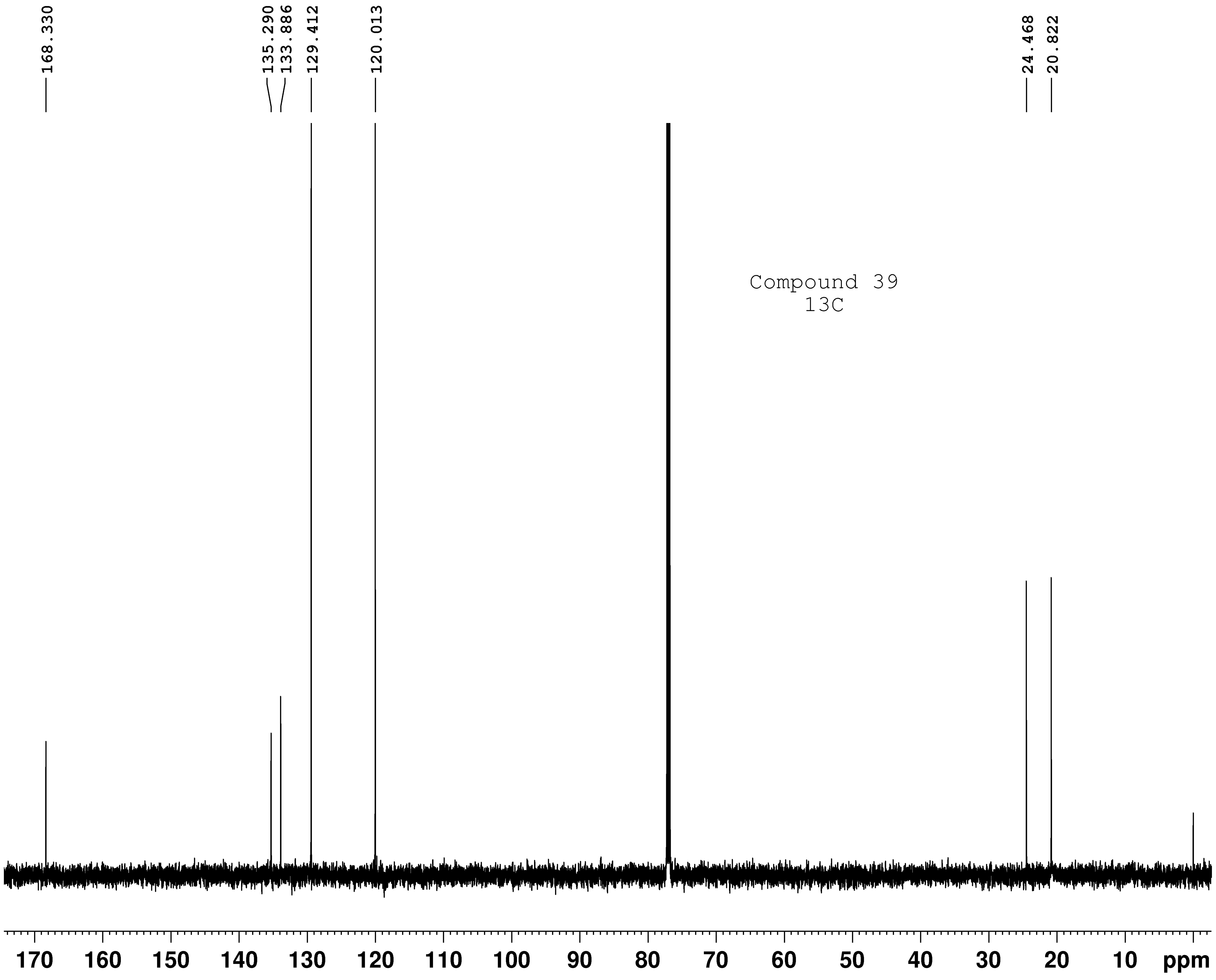

Current Data Parameters
NAME CD-125A
EXPNO 12
PROCNO 1

F2 - Acquisition Parameters

Date_	20220112
Time	17.13 h
INSTRUM	spect
PROBHD	Z847801_0047 (
PULPROG	zgdc30
TD	32768
SOLVENT	CDCl3
NS	128
DS	0
SWH	36057.691 Hz
FIDRES	2.200787 Hz
AQ	0.4543829 sec
RG	2050
DW	13.867 usec
DE	6.50 usec
TE	293.1 K
D1	1.50000000 sec
D11	0.03000000 sec
TD0	1
SFO1	150.8816889 MHz
NUC1	13C
P0	3.27 usec
P1	9.80 usec
PLW1	40.00000000 W
SFO2	599.9824003 MHz
NUC2	1H
CPDPRG[2	waltz16
PCPD2	70.00 usec
PLW2	20.00000000 W
PLW12	0.53044999 W

F2 - Processing parameters

SI	65536
SF	150.8651004 MHz
WDW	EM
SSB	0
LB	1.00 Hz
GB	0
PC	1.40

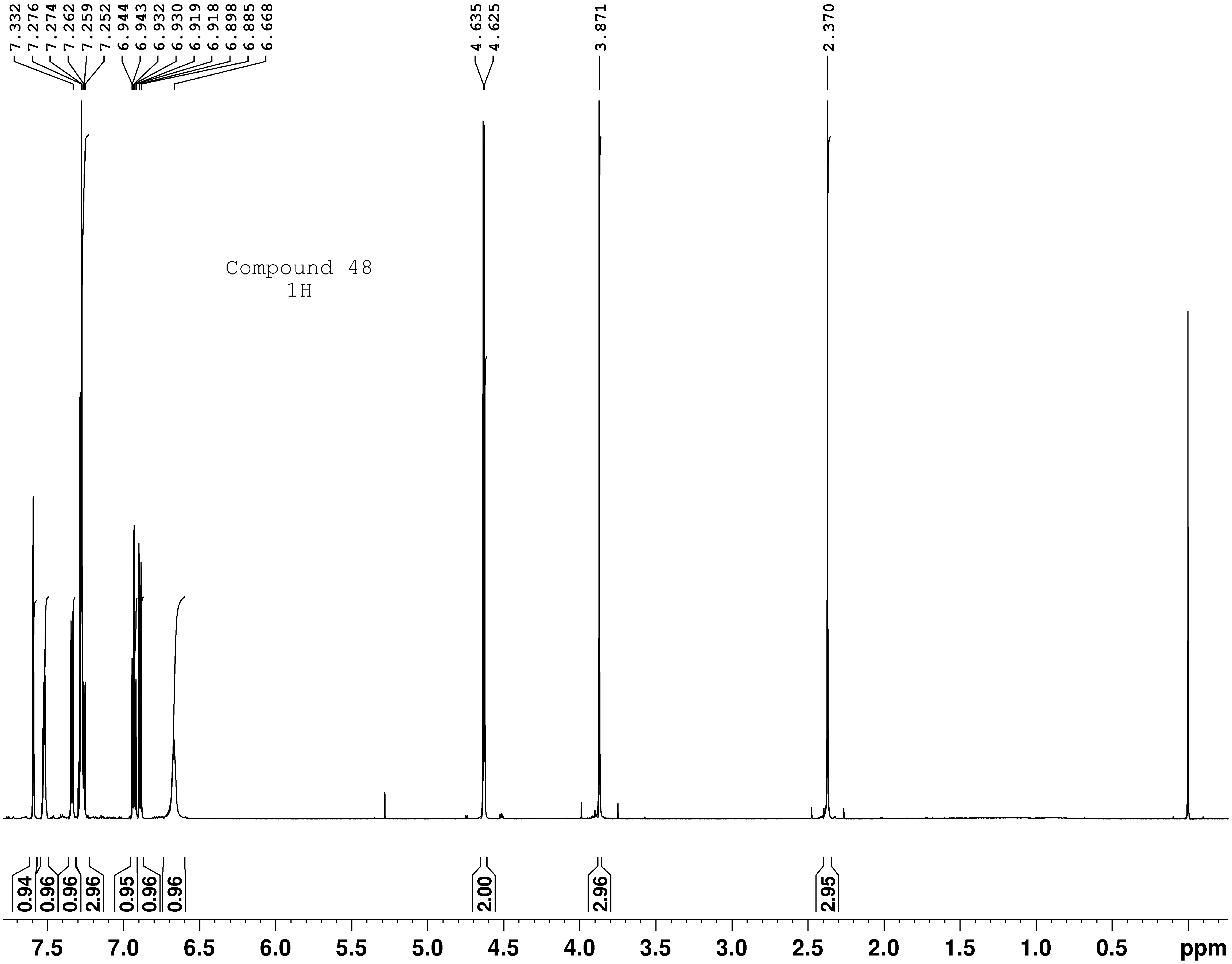

Current Data Parameters
NAME CE-103A
EXPNO 11
PROCNO 1

F2 - Acquisition Parameters
Date_ 20220224
Time 20.53 h
INSTRUM spect
PROBHD Z847801_0047 (
PULPROG zg30
TD 32768
SOLVENT CDCl3
NS 16
DS 0
SWH 9615.385 Hz
FIDRES 0.586877 Hz
AQ 1.7039360 sec
RG 101
DW 52.000 usec
DE 14.38 usec
TE 293.0 K
D1 1.00000000 sec
TD0 1
SFO1 599.9845606 MHz
NUC1 1H
P0 3.60 usec
P1 10.80 usec
PLW1 20.00000000 W

F2 - Processing parameters
SI 65536
SF 599.9800185 MHz
WDW EM
SSB 0
LB 0 Hz
GB 0
PC 1.00

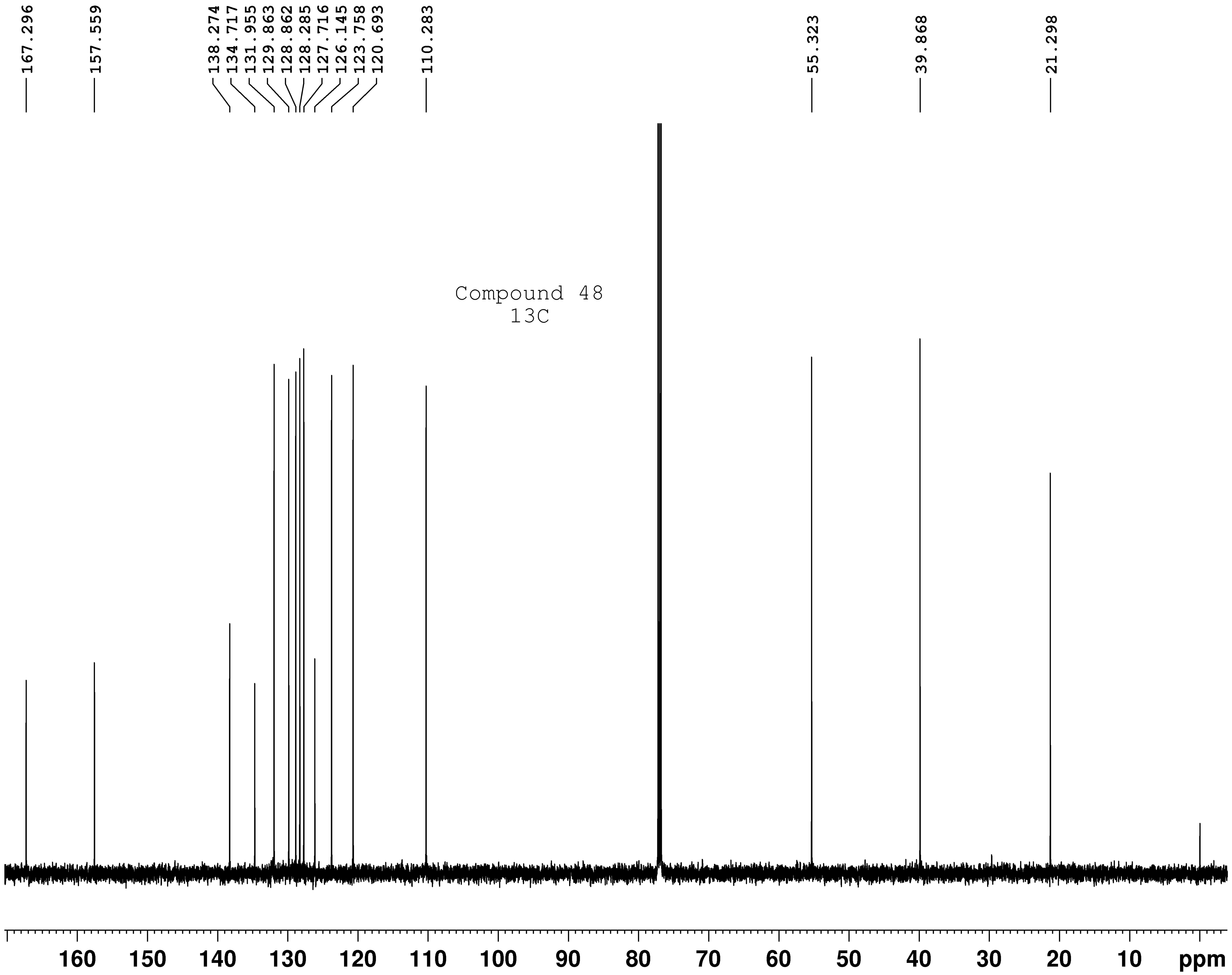


```
Current Data Parameters
NAME            CE-103A
EXPNO                12
PROCNO                1

F2 - Acquisition Parameters
Date_          20220224
Time              20.58 h
INSTRUM           spect
PROBHD   Z847801_0047 (
PULPROG          zgdc30
TD                32768
SOLVENT           CDCl3
NS                  128
DS                    0
SWH           36057.691 Hz
FIDRES         2.200787 Hz
AQ            0.4543829 sec
RG                 2050
DW               13.867 usec
DE                 6.50 usec
TE                293.0 K
D1           1.50000000 sec
D11          0.03000000 sec
TD0                   1
SFO1        150.8816889 MHz
NUC1                13C
P0                 3.27 usec
P1                 9.80 usec
PLW1        40.00000000 W
SFO2        599.9824003 MHz
NUC2                 1H
CPDPRG[2        waltz16
PCPD2             70.00 usec
PLW2        20.00000000 W
PLW12        0.53044999 W

F2 - Processing parameters
SI                65536
SF          150.8651046 MHz
WDW                  EM
SSB                   0
LB                 1.00 Hz
GB                    0
PC                 1.40
```

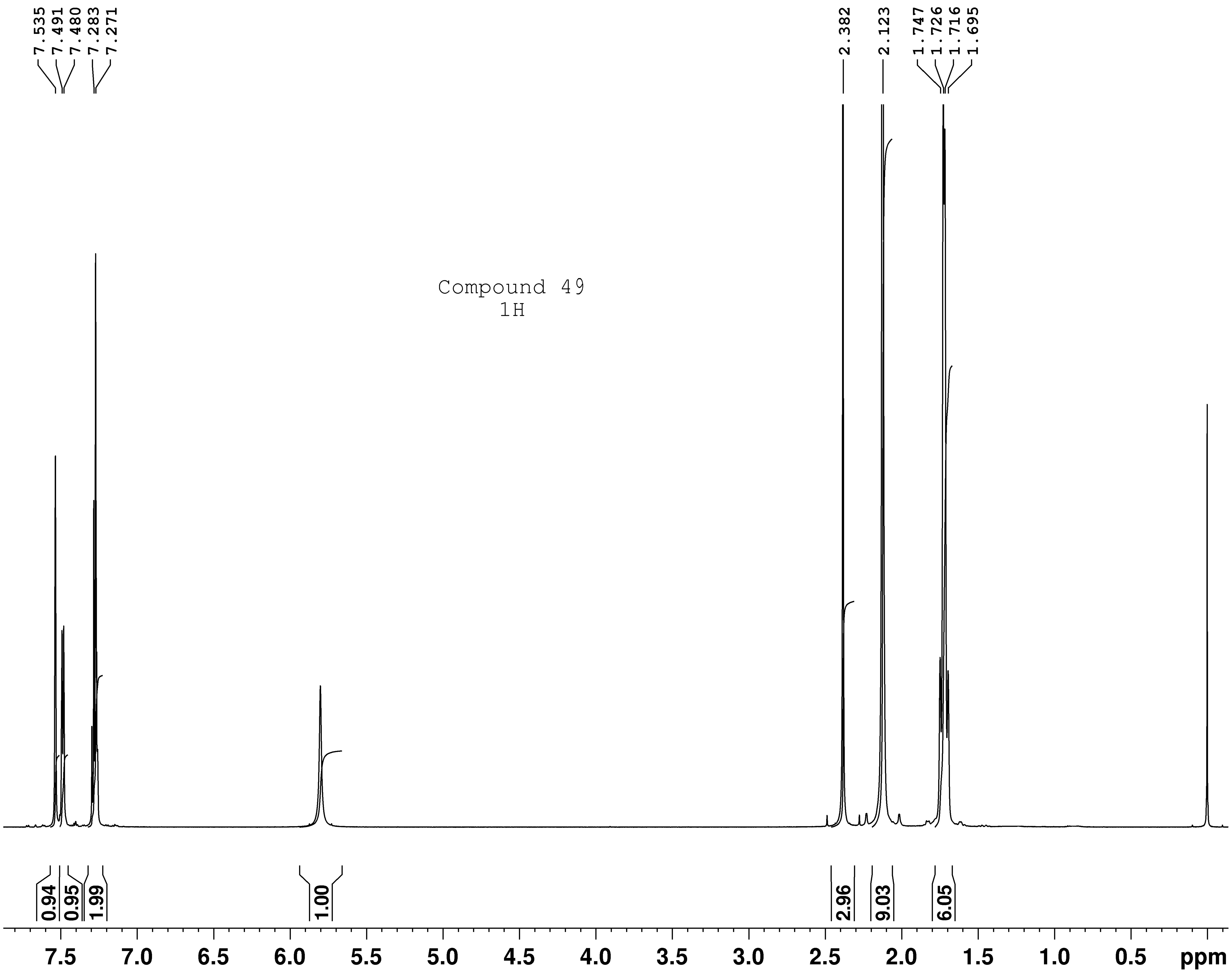

Current Data Parameters
NAME CD-170A
EXPNO 11
PROCNO 1

F2 - Acquisition Parameters
Date_ 20220224
Time 20.21 h
INSTRUM spect
PROBHD Z847801_0047 (
PULPROG zg30
TD 32768
SOLVENT CDCl3
NS 16
DS 0
SWH 9615.385 Hz
FIDRES 0.586877 Hz
AQ 1.7039360 sec
RG 80.6
DW 52.000 usec
DE 14.38 usec
TE 293.0 K
D1 1.00000000 sec
TD0 1
SFO1 599.9845606 MHz
NUC1 1H
P0 3.60 usec
P1 10.80 usec
PLW1 20.00000000 W

F2 - Processing parameters
SI 65536
SF 599.9800112 MHz
WDW EM
SSB 0
LB 0 Hz
GB 0
PC 1.00

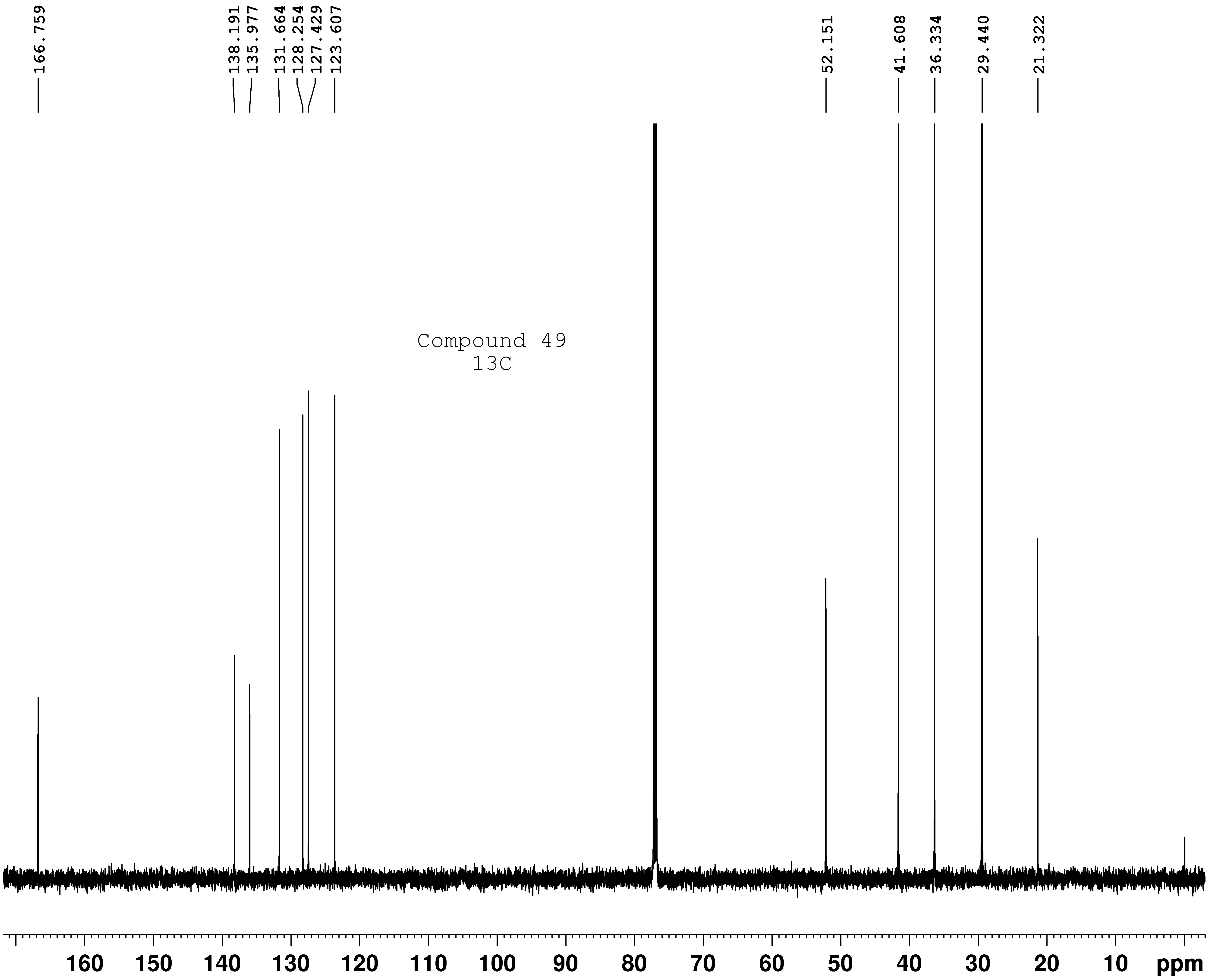


```
Current Data Parameters
NAME             CD-170A
EXPNO                 12
PROCNO                 1

F2 - Acquisition Parameters
Date_           20220224
Time               20.26 h
INSTRUM            spect
PROBHD   Z847801_0047 (
PULPROG          zgdc30
TD                 32768
SOLVENT            CDCl3
NS                   128
DS                     0
SWH            36057.691 Hz
FIDRES          2.200787 Hz
AQ             0.4543829 sec
RG                  2050
DW                13.867 usec
DE                  6.50 usec
TE                 293.0 K
D1            1.50000000 sec
D11           0.03000000 sec
TD0                    1
SFO1         150.8816889 MHz
NUC1                 13C
P0                  3.27 usec
P1                  9.80 usec
PLW1         40.00000000 W
SFO2         599.9824003 MHz
NUC2                  1H
CPDPRG[2         waltz16
PCPD2              70.00 usec
PLW2         20.00000000 W
PLW12         0.53044999 W

F2 - Processing parameters
SI                 65536
SF           150.8651018 MHz
WDW                   EM
SSB                    0
LB                  1.00 Hz
GB                     0
PC                  1.40
```

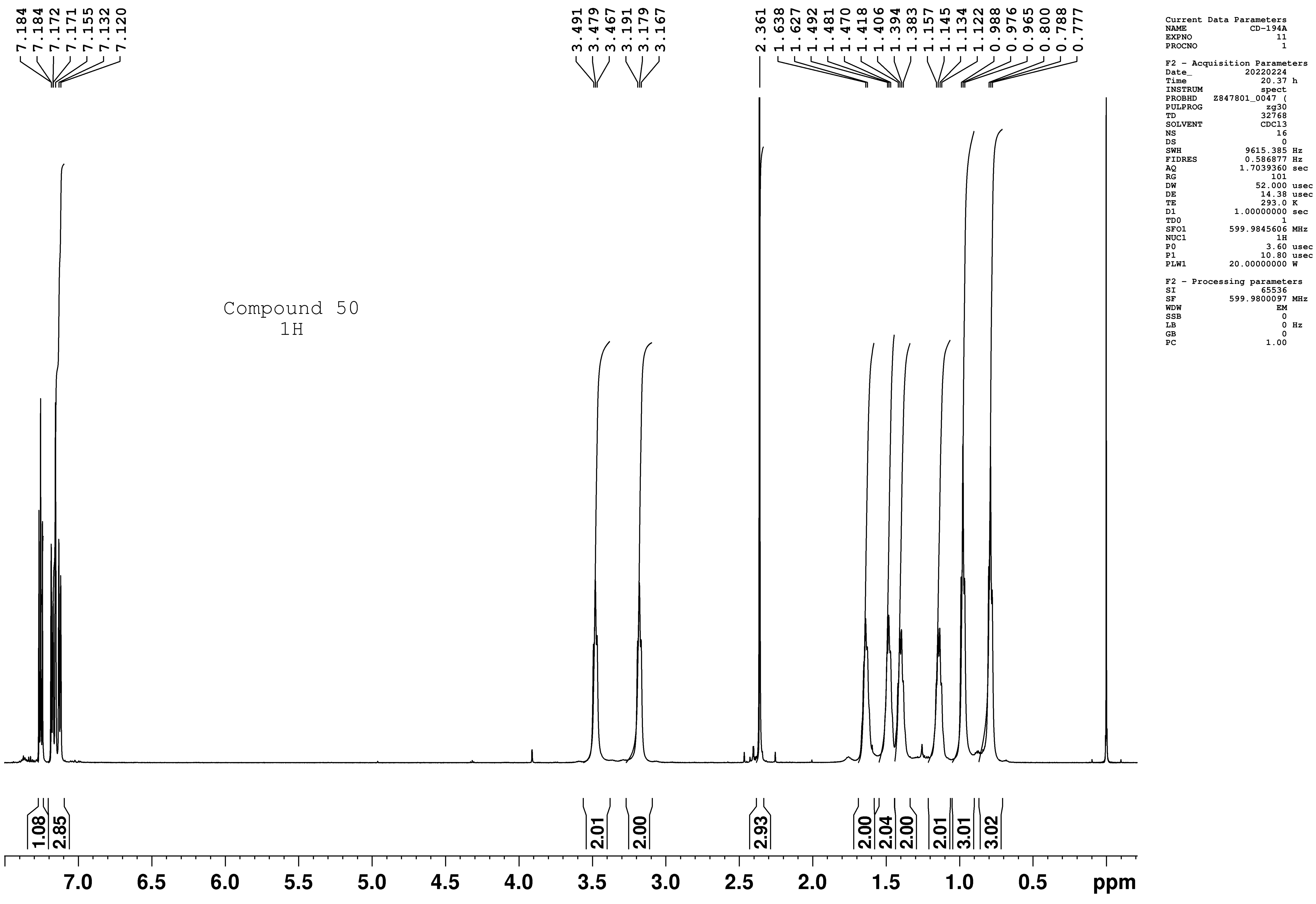
Compound 50
1H
7.184
7.184
7.172
7.171
7.155
7.132
7.120
3.491
3.479
3.467
3.191
3.179
3.167
2.361
1.638
1.627
1.492
1.481
1.470
1.418
1.406
1.394
1.383
1.157
1.145
1.134
1.122
0.988
0.976
0.965
0.800
0.788
0.777
1.08
2.85
2.01
2.00
2.93
2.00
2.04
2.00
2.01
3.01
3.02
7.0 6.5 6.0 5.5 5.0 4.5 4.0 3.5 3.0 2.5 2.0 1.5 1.0 0.5 ppm
Current Data Parameters
NAME CD-194A
EXPNO 11
PROCNO 1
F2 - Acquisition Parameters
Date_ 20220224
Time 20.37 h
INSTRUM spect
PROBHD Z847801_0047 (
PULPROG zg30
TD 32768
SOLVENT CDCl3
NS 16
DS 0
SWH 9615.385 Hz
FIDRES 0.586877 Hz
AQ 1.7039360 sec
RG 101
DW 52.000 usec
DE 14.38 usec
TE 293.0 K
D1 1.00000000 sec
TD0 1
SFO1 599.9845606 MHz
NUC1 1H
P0 3.60 usec
P1 10.80 usec
PLW1 20.00000000 W
F2 - Processing parameters
SI 65536
SF 599.9800097 MHz
WDW EM
SSB 0
LB 0 Hz
GB 0
PC 1.00

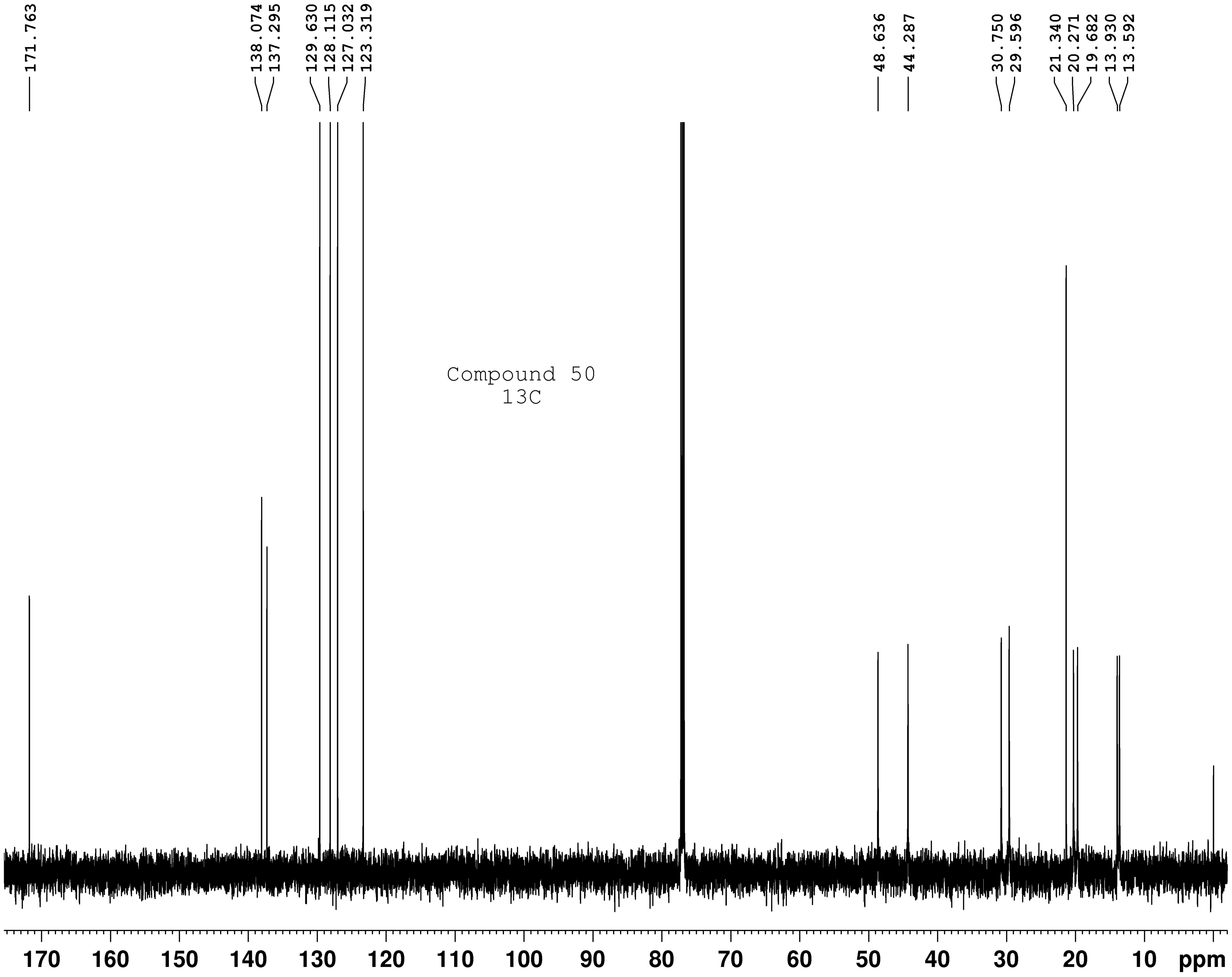

Current Data Parameters
NAME CD-194A
EXPNO 12
PROCNO 1

F2 - Acquisition Parameters
Date_ 20220224
Time 20.43 h
INSTRUM spect
PROBHD Z847801_0047 (
PULPROG zgdc30
TD 32768
SOLVENT CDCl3
NS 128
DS 0
SWH 36057.691 Hz
FIDRES 2.200787 Hz
AQ 0.4543829 sec
RG 2050
DW 13.867 usec
DE 6.50 usec
TE 293.1 K
D1 1.50000000 sec
D11 0.03000000 sec
TD0 1
SFO1 150.8816889 MHz
NUC1 13C
P0 3.27 usec
P1 9.80 usec
PLW1 40.00000000 W
SFO2 599.9824003 MHz
NUC2 1H
CPDPRG[2 waltz16
PCPD2 70.00 usec
PLW2 20.00000000 W
PLW12 0.53044999 W

F2 - Processing parameters
SI 65536
SF 150.8650996 MHz
WDW EM
SSB 0
LB 1.00 Hz
GB 0
PC 1.40

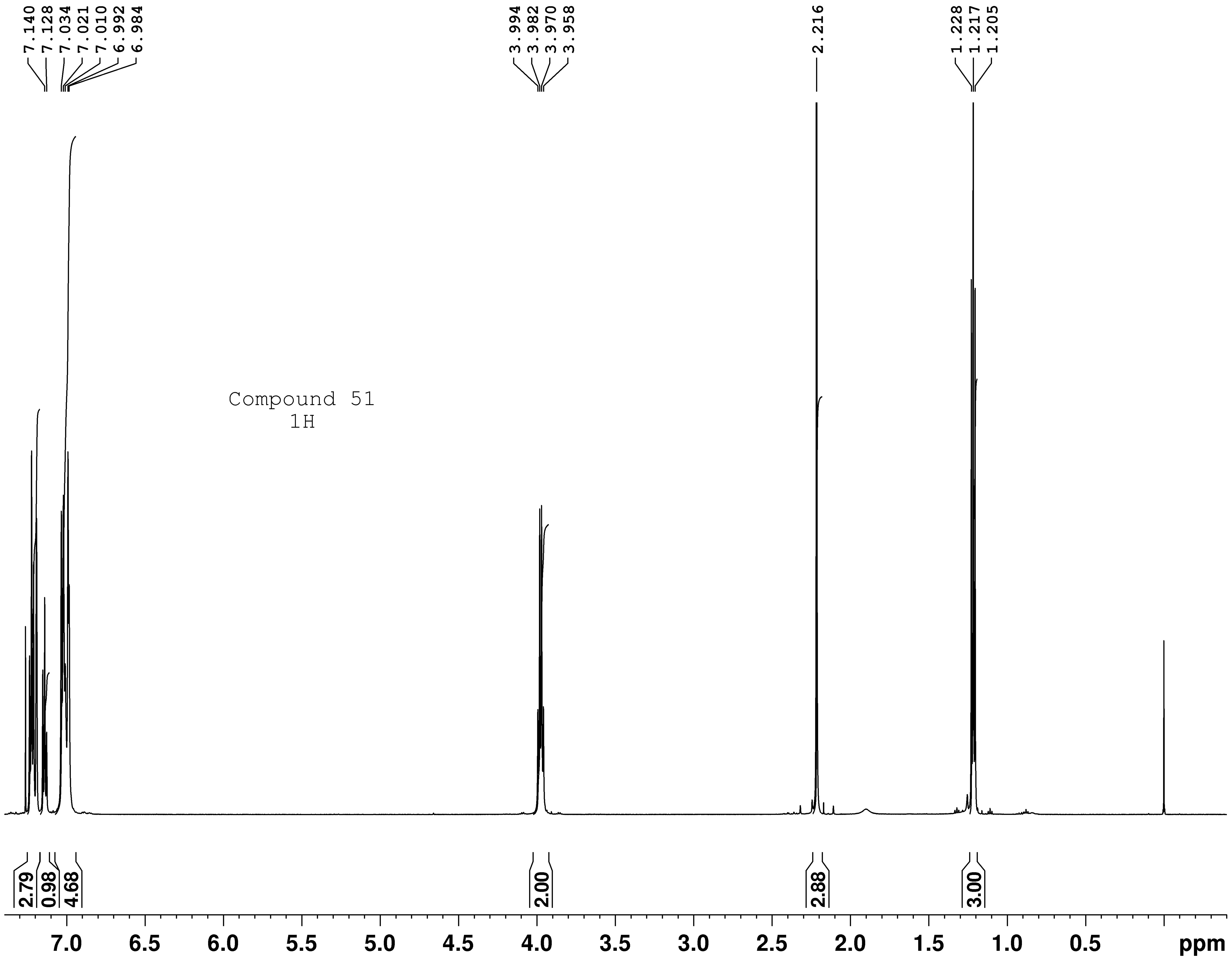


```
Current Data Parameters
NAME            CD-175A
EXPNO                11
PROCNO                1

F2 - Acquisition Parameters
Date_          20210316
Time              20.10 h
INSTRUM           spect
PROBHD   Z847801_0047 (
PULPROG            zg30
TD                32768
SOLVENT           CDCl3
NS                   32
DS                    0
SWH            9615.385 Hz
FIDRES         0.586877 Hz
AQ            1.7039360 sec
RG                 80.6
DW               52.000 usec
DE                14.37 usec
TE                293.0 K
D1           1.00000000 sec
TD0                   1
SFO1        600.0145608 MHz
NUC1                 1H
P0                 3.62 usec
P1                10.85 usec
PLW1        20.00000000 W

F2 - Processing parameters
SI                65536
SF          600.0100129 MHz
WDW                  EM
SSB                   0
LB                    0 Hz
GB                    0
PC                 1.00
```

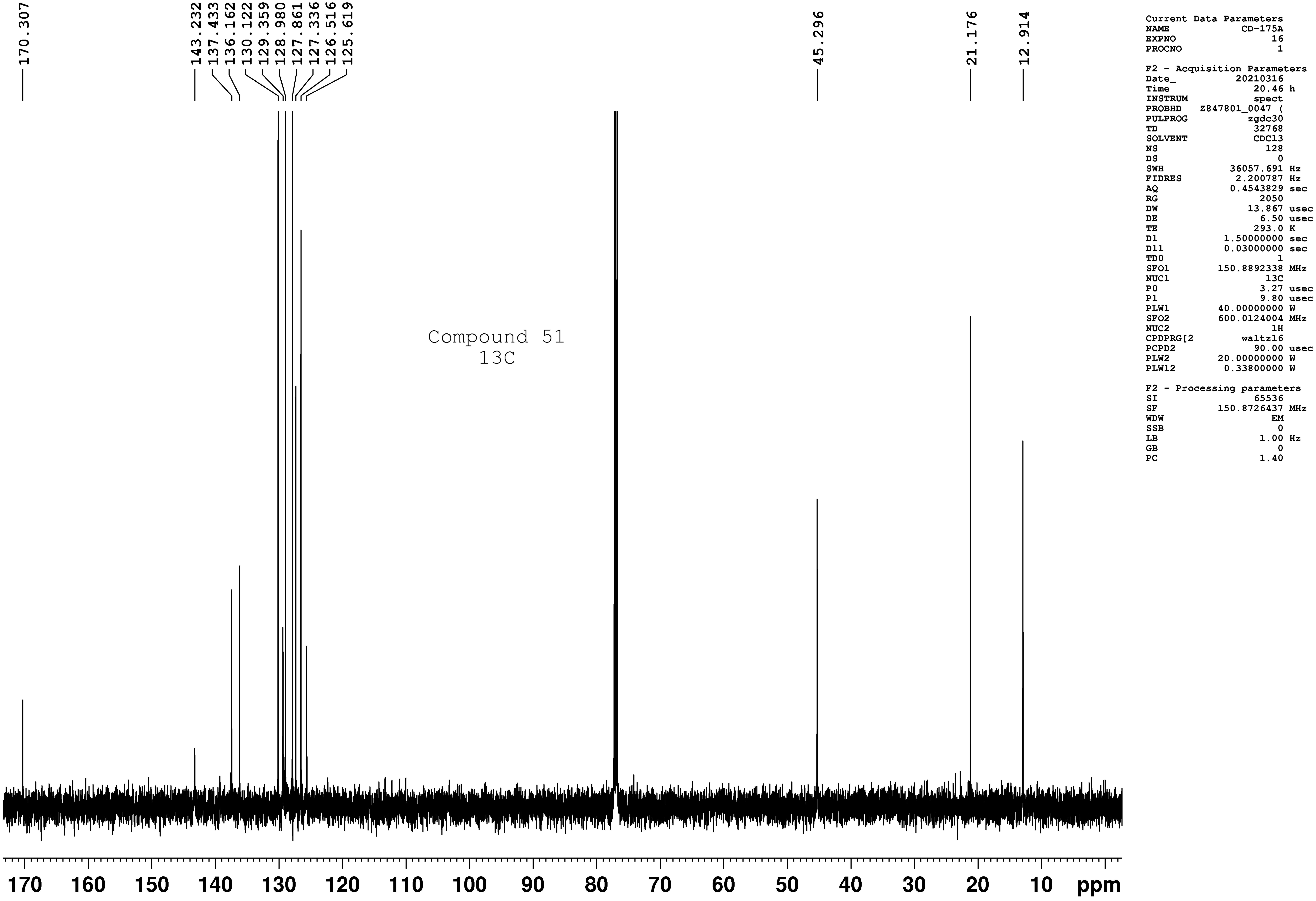
170.307
143.232
137.433
136.162
130.122
129.359
128.980
127.861
127.336
126.516
125.619
45.296
21.176
12.914
Compound 51
13C
170
160
150
140
130
120
110
100
90
80
70
60
50
40
30
20
10
ppm
Current Data Parameters
NAME CD-175A
EXPNO 16
PROCNO 1
F2 - Acquisition Parameters
Date_ 20210316
Time 20.46 h
INSTRUM spect
PROBHD Z847801_0047 (
PULPROG zgdc30
TD 32768
SOLVENT CDCl3
NS 128
DS 0
SWH 36057.691 Hz
FIDRES 2.200787 Hz
AQ 0.4543829 sec
RG 2050
DW 13.867 usec
DE 6.50 usec
TE 293.0 K
D1 1.50000000 sec
D11 0.03000000 sec
TD0 1
SFO1 150.8892338 MHz
NUC1 13C
P0 3.27 usec
P1 9.80 usec
PLW1 40.00000000 W
SFO2 600.0124004 MHz
NUC2 1H
CPDPRG[2 waltz16
PCPD2 90.00 usec
PLW2 20.00000000 W
PLW12 0.33800000 W
F2 - Processing parameters
SI 65536
SF 150.8726437 MHz
WDW EM
SSB 0
LB 1.00 Hz
GB 0
PC 1.40

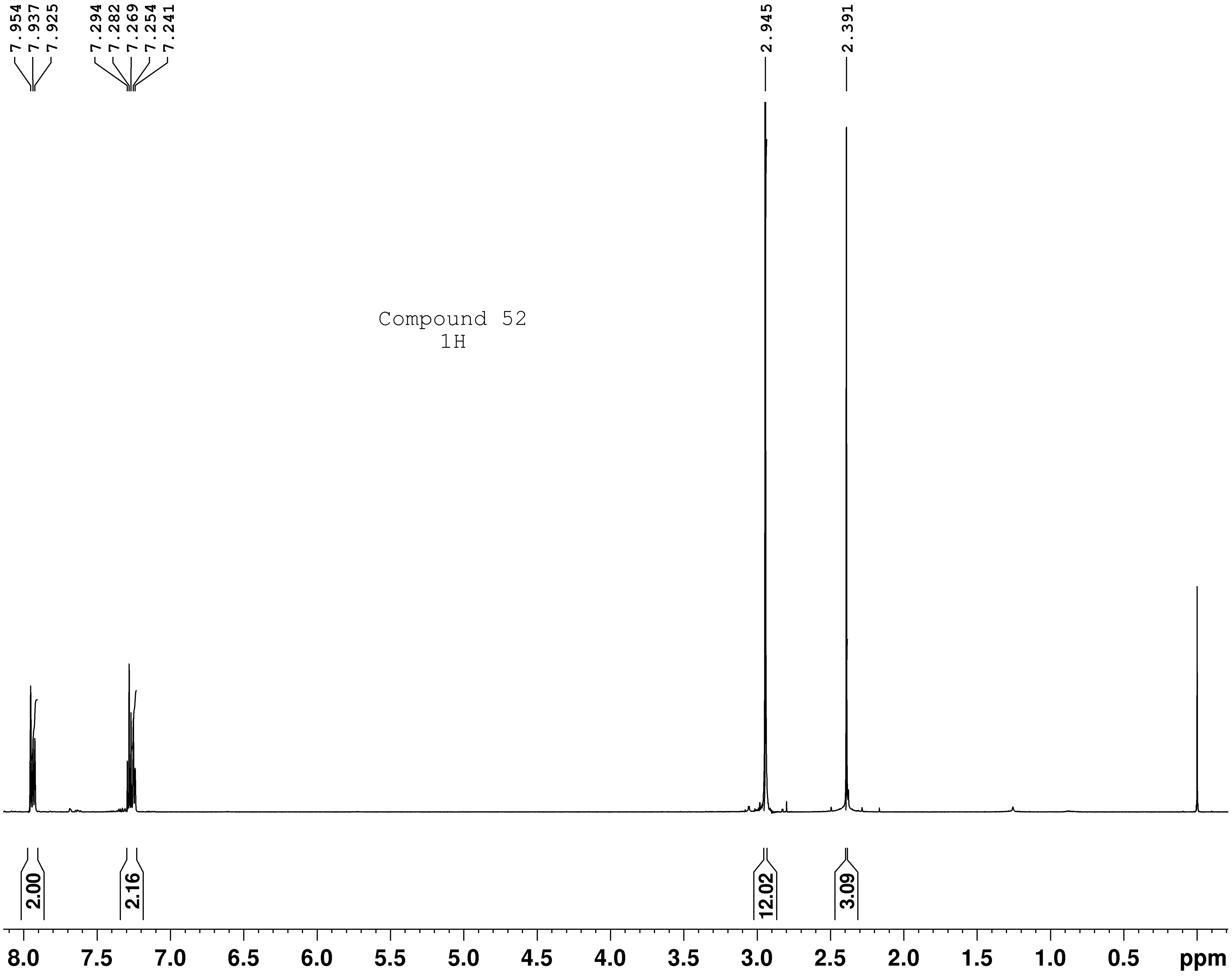

Current Data Parameters
NAME CE-23A
EXPNO 11
PROCNO 1

F2 - Acquisition Parameters
Date_ 20210222
Time 18.40 h
INSTRUM spect
PROBHD Z847801_0047 (
PULPROG zg30
TD 32768
SOLVENT CDCl3
NS 1
DS 0
SWH 9615.385 Hz
FIDRES 0.586877 Hz
AQ 1.7039360 sec
RG 50.8
DW 52.000 usec
DE 14.37 usec
TE 293.0 K
D1 1.00000000 sec
TD0 1
SFO1 600.0145608 MHz
NUC1 1H
P0 3.62 usec
P1 10.85 usec
PLW1 20.00000000 W

F2 - Processing parameters
SI 65536
SF 600.0100013 MHz
WDW EM
SSB 0
LB 0 Hz
GB 0
PC 1.00

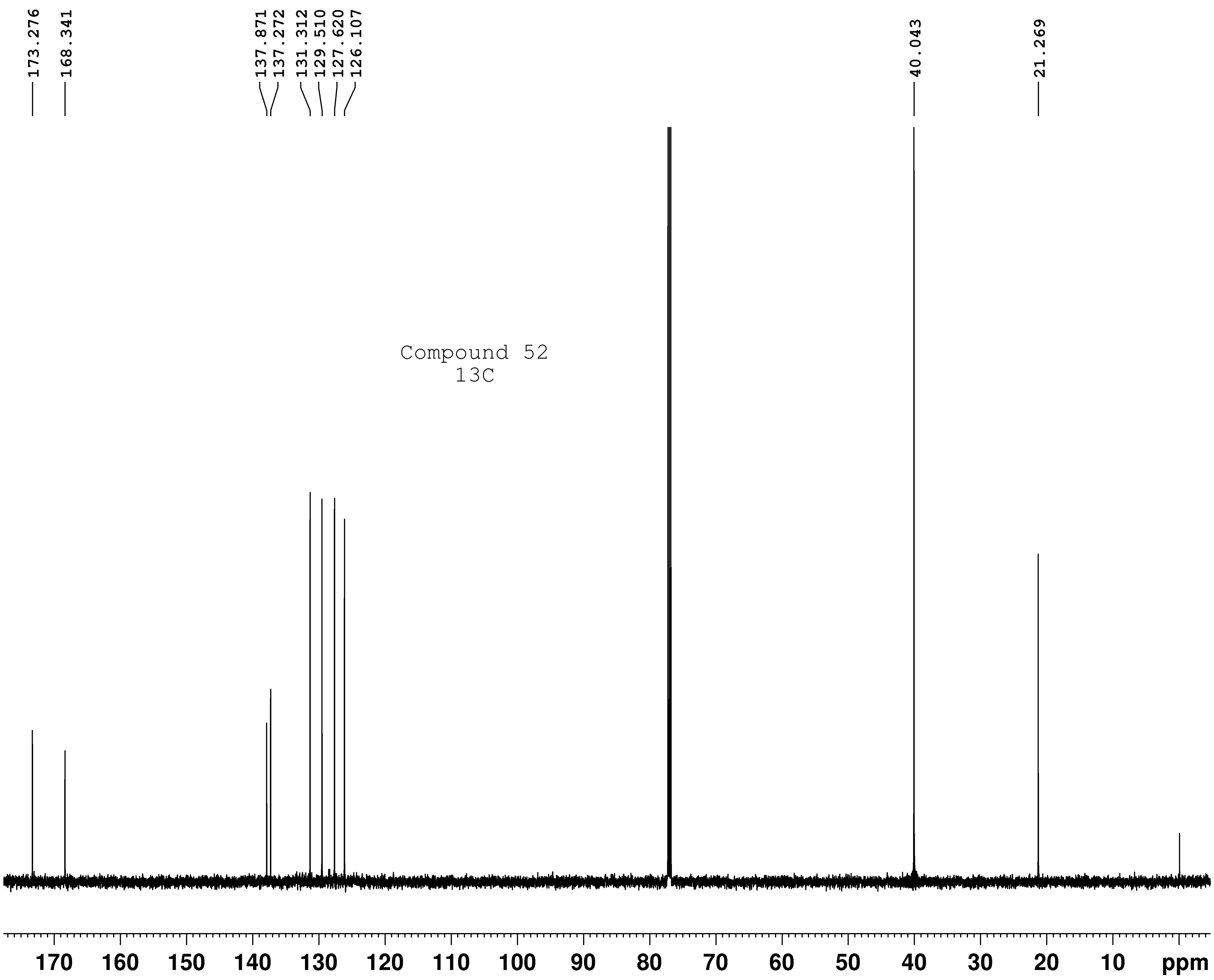

Current Data Parameters
NAME CE-23A
EXPNO 16
PROCNO 1

F2 - Acquisition Parameters
Date_ 20210222
Time 19.15 h
INSTRUM spect
PROBHD Z847801_0047 (
PULPROG zgdc30
TD 32768
SOLVENT CDCl3
NS 128
DS 0
SWH 36057.691 Hz
FIDRES 2.200787 Hz
AQ 0.4543829 sec
RG 2050
DW 13.867 usec
DE 6.50 usec
TE 293.1 K
D1 1.50000000 sec
D11 0.03000000 sec
TD0 1
SFO1 150.8892338 MHz
NUC1 13C
P0 3.27 usec
P1 9.80 usec
PLW1 40.00000000 W
SFO2 600.0124004 MHz
NUC2 1H
CPDPRG[2 waltz16
PCPD2 90.00 usec
PLW2 20.00000000 W
PLW12 0.33800000 W

F2 - Processing parameters
SI 65536
SF 150.8726510 MHz
WDW EM
SSB 0
LB 1.00 Hz
GB 0
PC 1.40

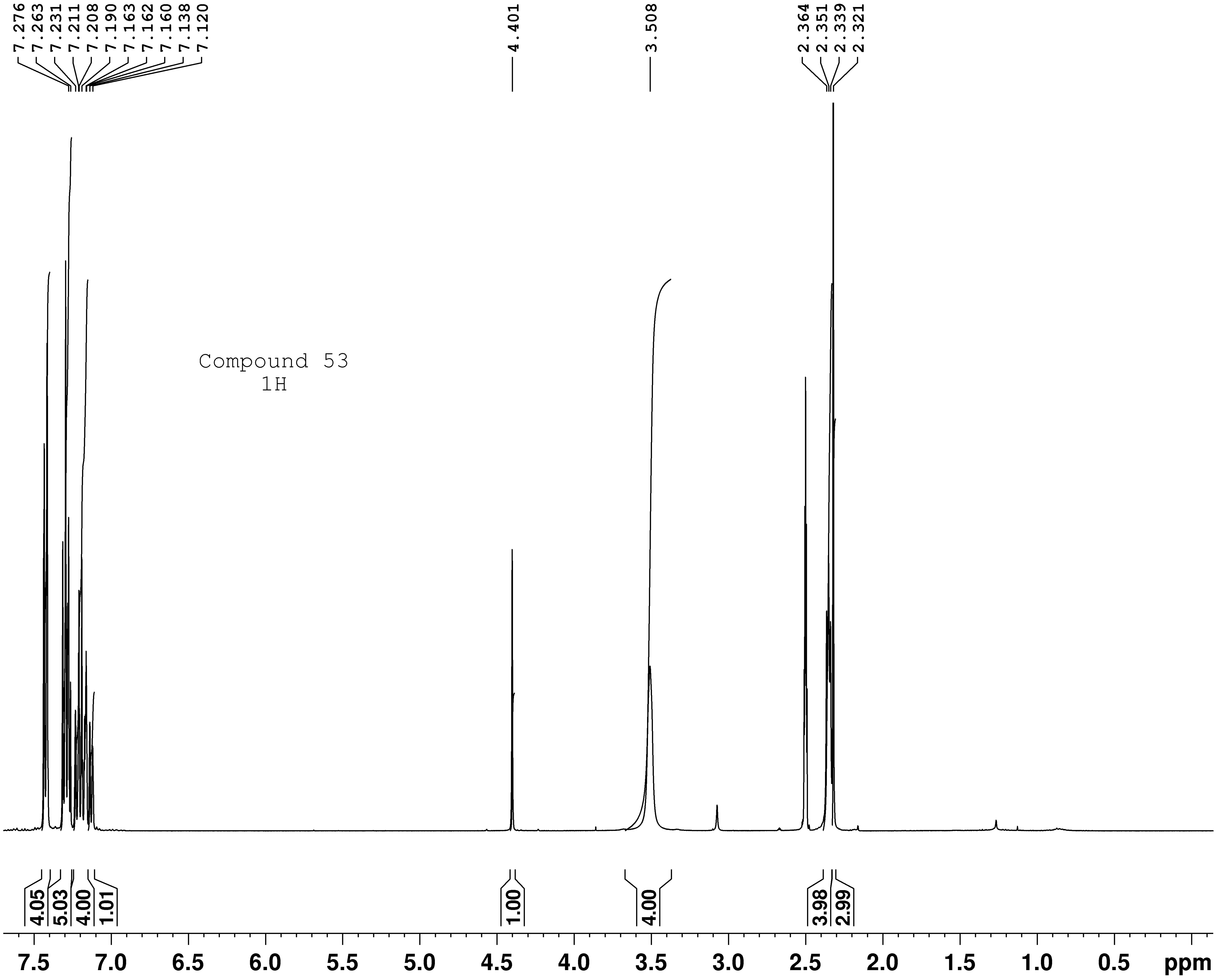


```
Current Data Parameters
NAME            CD-197B
EXPNO                11
PROCNO                1

F2 - Acquisition Parameters
Date_          20220416
Time            12.56 h
INSTRUM  Avance Neo 400
PROBHD   Z175272_0007 (
PULPROG            zg30
TD                32768
SOLVENT            DMSO
NS                   32
DS                    0
SWH          6578.947 Hz
FIDRES       0.401547 Hz
AQ          2.4903679 sec
RG                  101
DW            76.000 usec
DE              7.79 usec
TE             353.0 K
D1         2.00000000 sec
TD0                   1
SFO1      400.1330415 MHz
NUC1                 1H
P0              3.33 usec
P1             10.00 usec
PLW1      20.07200050 W

F2 - Processing parameters
SI                65536
SF        400.1300034 MHz
WDW                  EM
SSB                   0
LB                    0 Hz
GB                    0
PC                 1.00
```

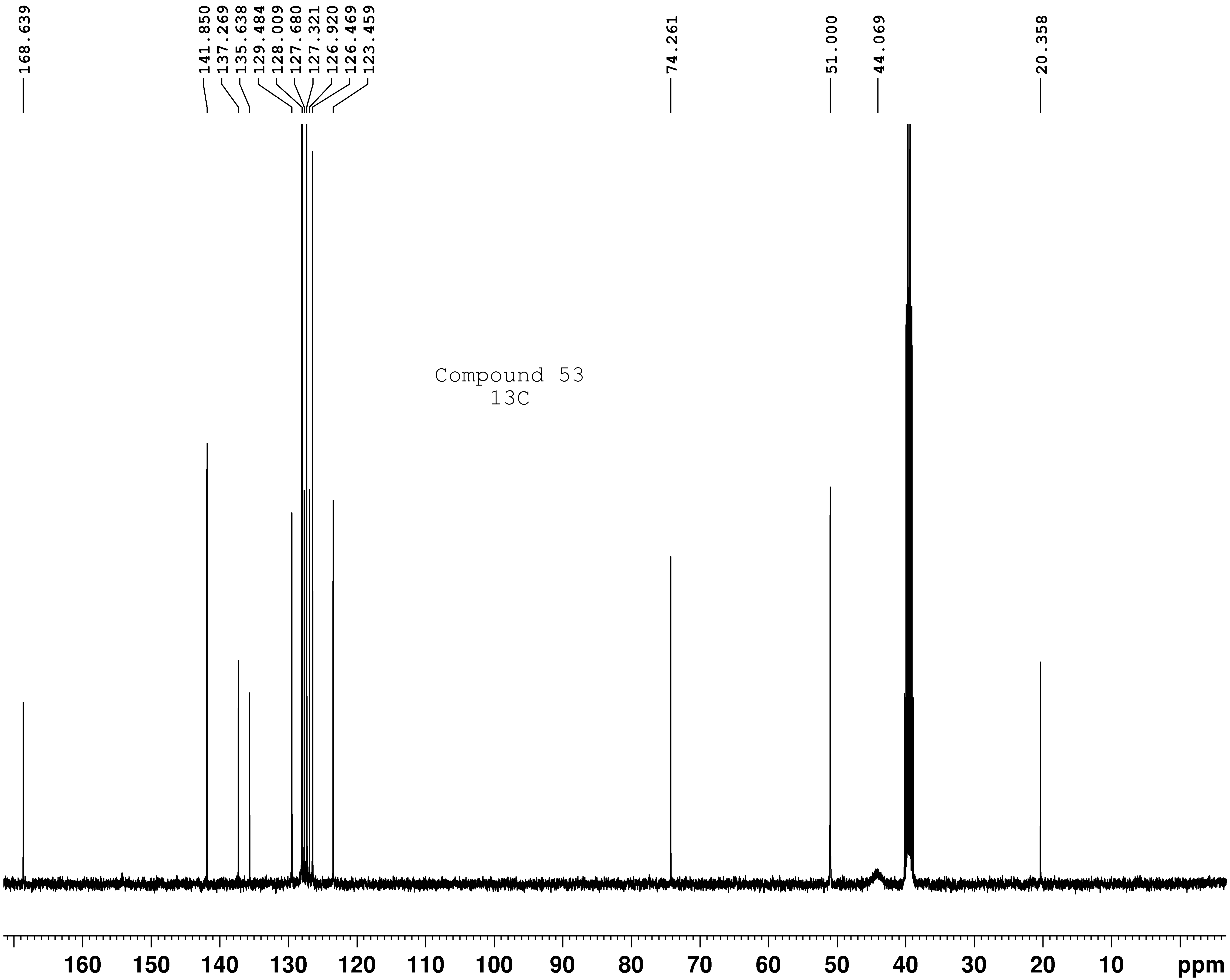


```
Current Data Parameters
NAME              CD-197B
EXPNO                  12
PROCNO                  1

F2 - Acquisition Parameters
Date_            20220416
Time                13.35 h
INSTRUM  Avance Neo 400
PROBHD   Z175272_0007 (
PULPROG           zgdc30
TD                 32768
SOLVENT             DMSO
NS                  1024
DS                     0
SWH            23809.523 Hz
FIDRES          1.453218 Hz
AQ             0.6881280 sec
RG                  45.2
DW                21.000 usec
DE                  6.50 usec
TE                 353.0 K
D1            1.50000000 sec
D11           0.03000000 sec
TD0                    1
SFO1         100.6238383 MHz
NUC1                 13C
P0                  3.33 usec
P1                 10.00 usec
PLW1         58.46900177 W
SFO2         400.1316008 MHz
NUC2                  1H
CPDPRG[2         waltz16
PCPD2              90.00 usec
PLW2         20.07200050 W
PLW12         0.24781001 W

F2 - Processing parameters
SI                 65536
SF           100.6128587 MHz
WDW                   EM
SSB                    0
LB                  1.00 Hz
GB                     0
PC                  1.40
```

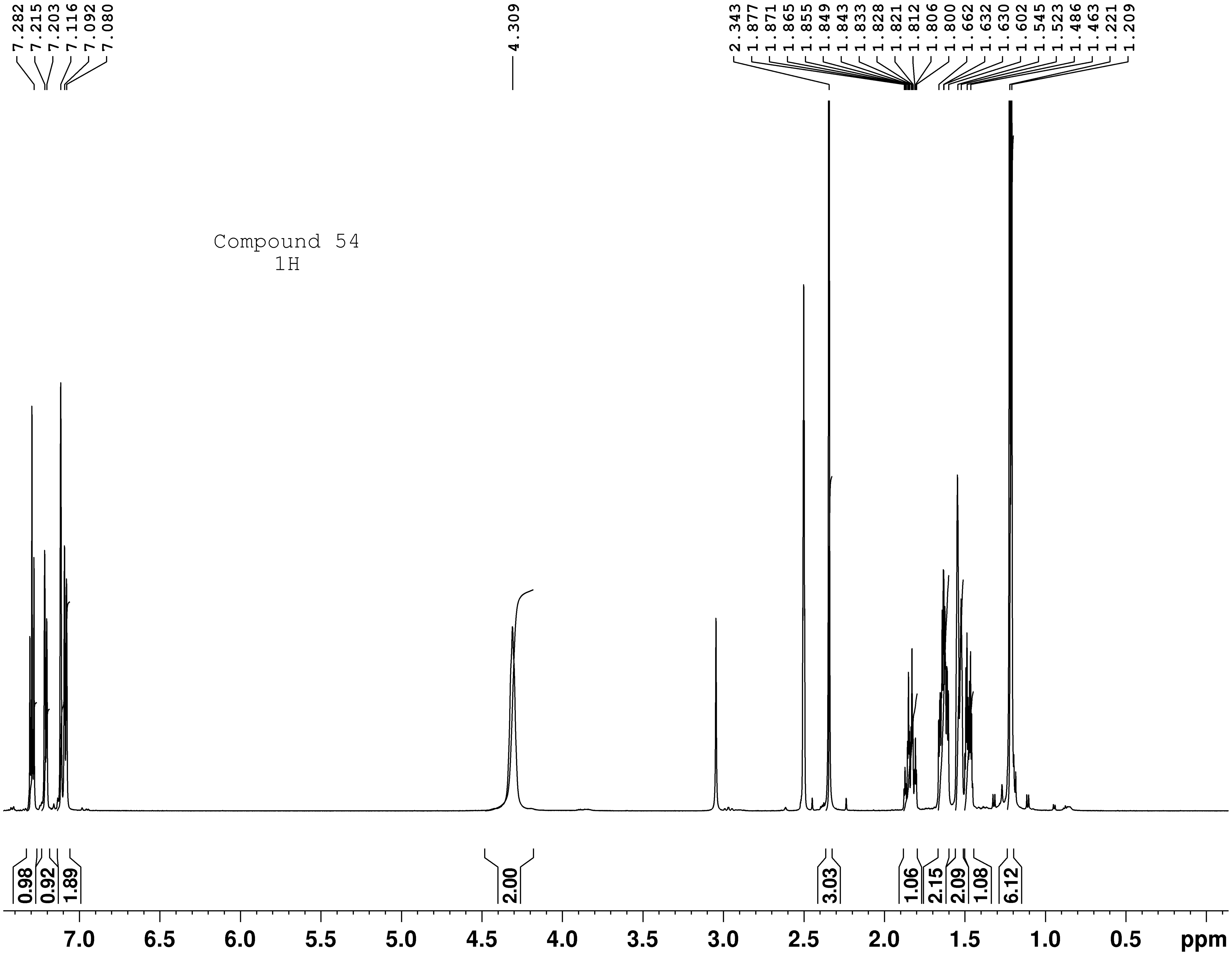

Current Data Parameters		
NAME	CD-192A	
EXPNO	11	
PROCNO	1	
**F2 - Acquisition Parameters**		
Date_	20220225	
Time	21.18	h
INSTRUM	spect	
PROBHD	Z847801_0047 (	
PULPROG	zg30	
TD	32768	
SOLVENT	DMSO	
NS	32	
DS	0	
SWH	9615.385	Hz
FIDRES	0.586877	Hz
AQ	1.7039360	sec
RG	90.5	
DW	52.000	usec
DE	14.38	usec
TE	353.1	K
D1	1.00000000	sec
TD0	1	
SFO1	599.9845606	MHz
NUC1	1H	
P0	3.60	usec
P1	10.80	usec
PLW1	20.00000000	W
**F2 - Processing parameters**		
SI	65536	
SF	599.9800046	MHz
WDW	EM	
SSB	0	
LB	0	Hz
GB	0	
PC	1.00	

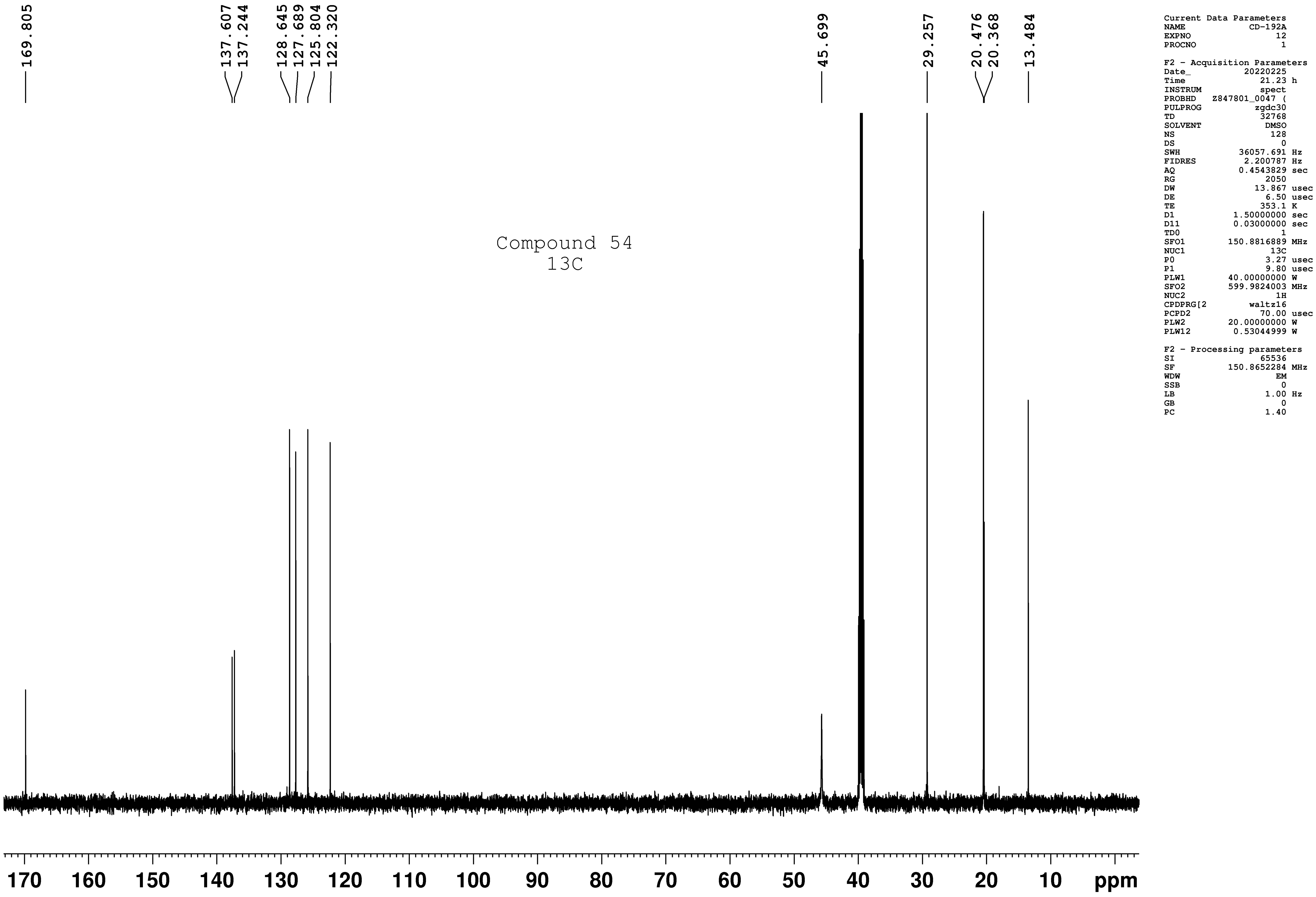
169.805
137.607
137.244
128.645
127.689
125.804
122.320
45.699
29.257
20.476
20.368
13.484
Compound 54
13C
170 160 150 140 130 120 110 100 90 80 70 60 50 40 30 20 10 ppm
Current Data Parameters
NAME CD-192A
EXPNO 12
PROCNO 1
F2 - Acquisition Parameters
Date_ 20220225
Time 21.23 h
INSTRUM spect
PROBHD Z847801_0047 (
PULPROG zgdc30
TD 32768
SOLVENT DMSO
NS 128
DS 0
SWH 36057.691 Hz
FIDRES 2.200787 Hz
AQ 0.4543829 sec
RG 2050
DW 13.867 usec
DE 6.50 usec
TE 353.1 K
D1 1.50000000 sec
D11 0.03000000 sec
TD0 1
SFO1 150.8816889 MHz
NUC1 13C
P0 3.27 usec
P1 9.80 usec
PLW1 40.00000000 W
SFO2 599.9824003 MHz
NUC2 1H
CPDPRG[2 waltz16
PCPD2 70.00 usec
PLW2 20.00000000 W
PLW12 0.53044999 W
F2 - Processing parameters
SI 65536
SF 150.8652284 MHz
WDW EM
SSB 0
LB 1.00 Hz
GB 0
PC 1.40

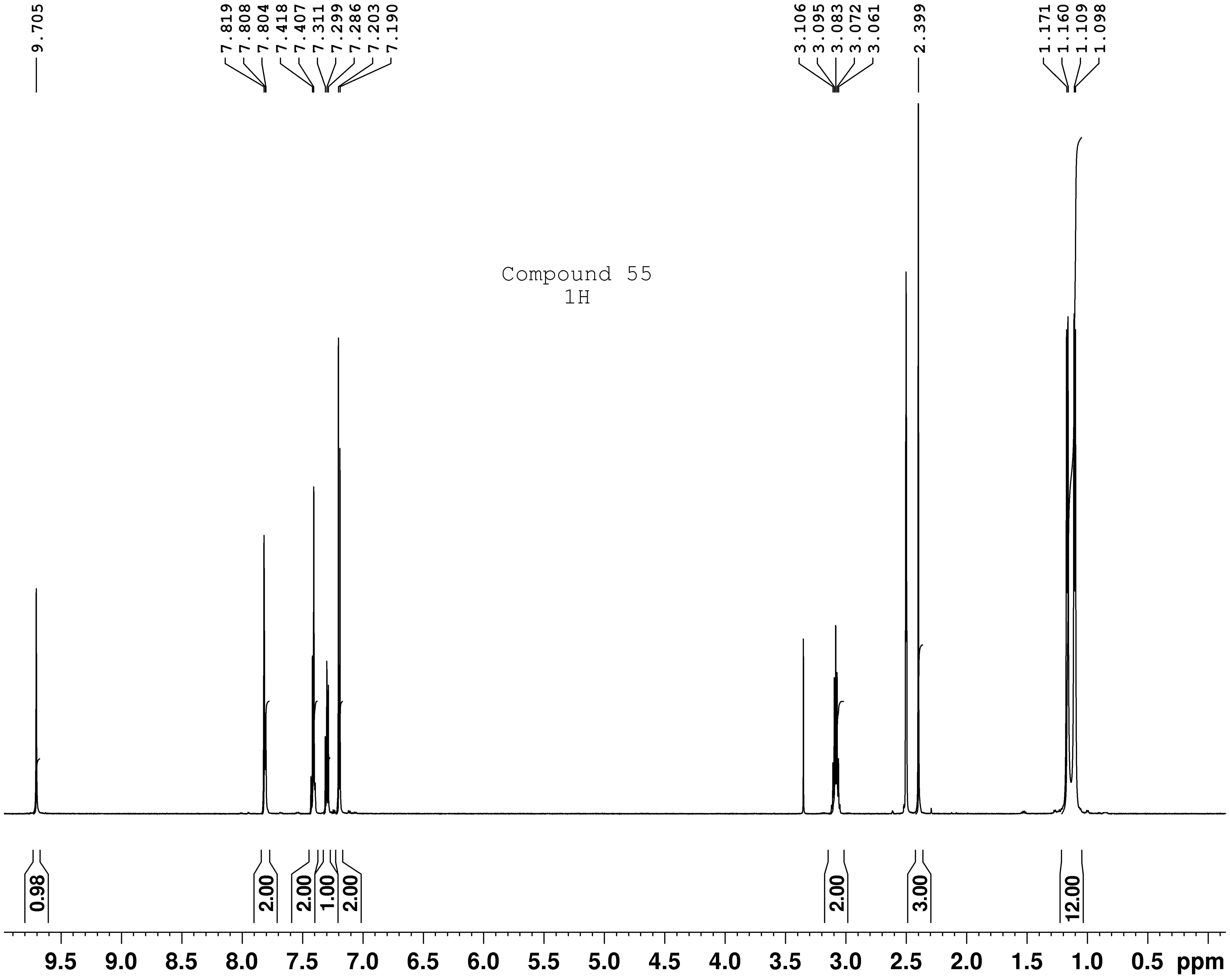


```
Current Data Parameters
NAME             CD-181A
EXPNO                 11
PROCNO                 1

F2 - Acquisition Parameters
Date_           20210428
Time                0.15 h
INSTRUM            spect
PROBHD   Z847801_0047 (
PULPROG             zg30
TD                 32768
SOLVENT             DMSO
NS                     1
DS                     0
SWH             9615.385 Hz
FIDRES          0.586877 Hz
AQ             1.7039360 sec
RG                  90.5
DW                52.000 usec
DE                 14.37 usec
TE                 293.0 K
D1            1.00000000 sec
TD0                    1
SFO1         600.0145608 MHz
NUC1                  1H
P0                  3.62 usec
P1                 10.85 usec
PLW1         20.00000000 W

F2 - Processing parameters
SI                 65536
SF           600.0100047 MHz
WDW                   EM
SSB                    0
LB                     0 Hz
GB                     0
PC                  1.00
```

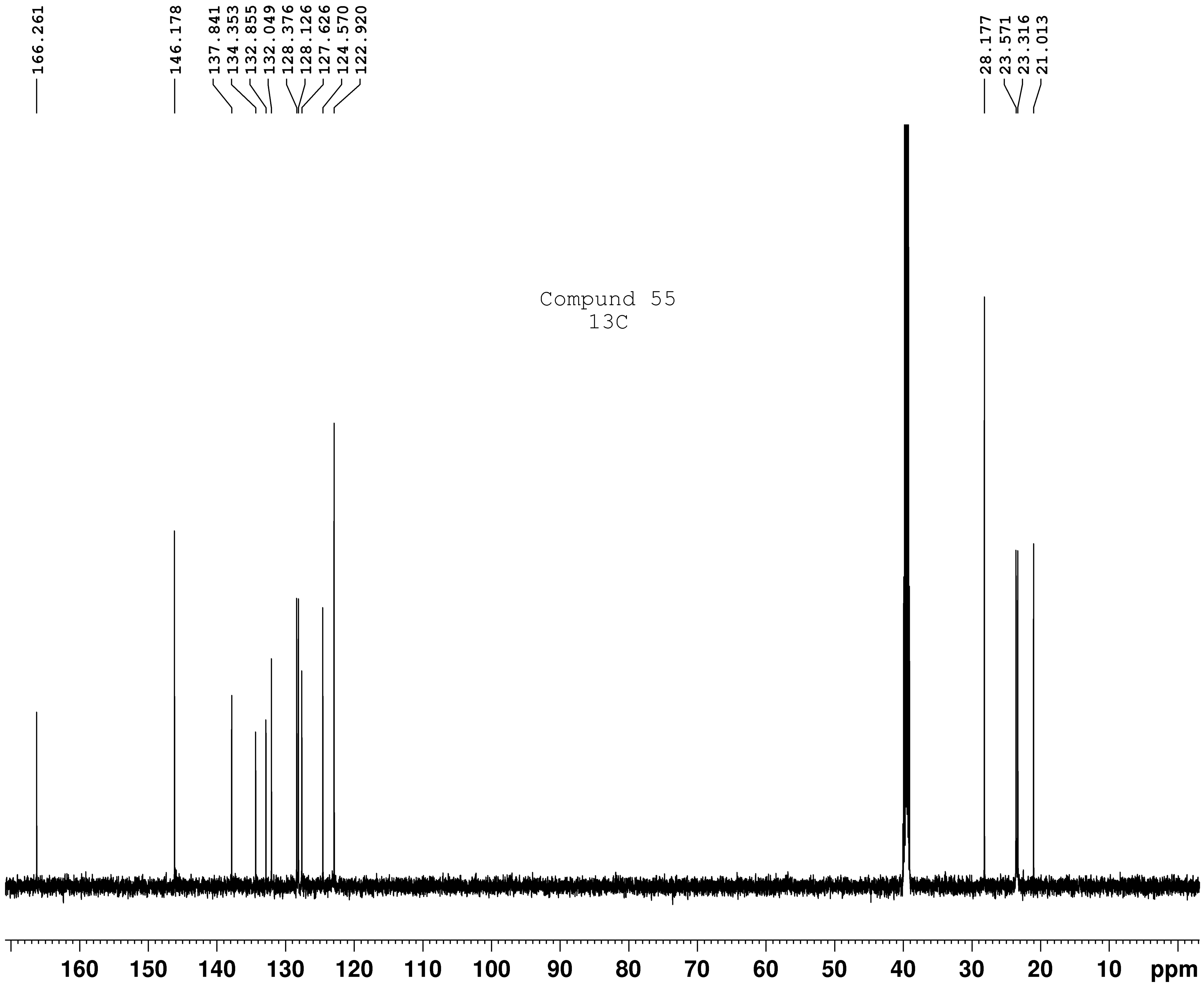

Current Data Parameters
NAME CD-181A
EXPNO 12
PROCNO 1

F2 - Acquisition Parameters
Date_ 20210428
Time 0.20 h
INSTRUM spect
PROBHD Z847801_0047 (
PULPROG zgdc30
TD 32768
SOLVENT DMSO
NS 128
DS 0
SWH 36057.691 Hz
FIDRES 2.200787 Hz
AQ 0.4543829 sec
RG 2050
DW 13.867 usec
DE 6.50 usec
TE 293.0 K
D1 1.50000000 sec
D11 0.03000000 sec
TD0 1
SFO1 150.8892338 MHz
NUC1 13C
P0 3.27 usec
P1 9.80 usec
PLW1 40.00000000 W
SFO2 600.0124004 MHz
NUC2 1H
CPDPRG[2 waltz16
PCPD2 90.00 usec
PLW2 20.00000000 W
PLW12 0.33800000 W

F2 - Processing parameters
SI 65536
SF 150.8727027 MHz
WDW EM
SSB 0
LB 1.00 Hz
GB 0
PC 1.40

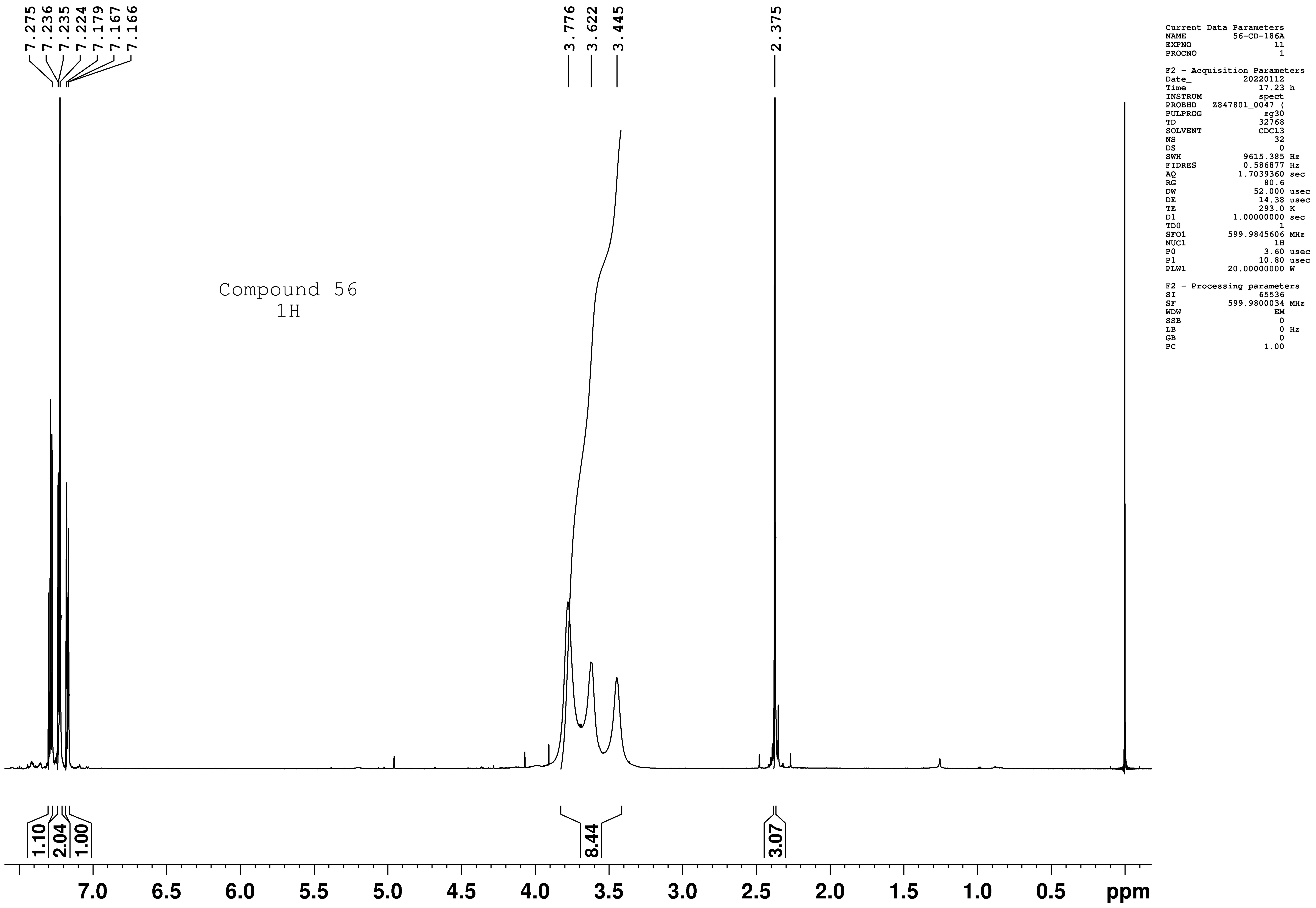
7.275
7.236
7.235
7.224
7.179
7.167
7.166
3.776
3.622
3.445
2.375
Compound 56
1H
1.10
2.04
1.00
8.44
3.07
7.0
6.5
6.0
5.5
5.0
4.5
4.0
3.5
3.0
2.5
2.0
1.5
1.0
0.5
ppm
Current Data Parameters
NAME 56-CD-186A
EXPNO 11
PROCNO 1
F2 - Acquisition Parameters
Date_ 20220112
Time 17.23 h
INSTRUM spect
PROBHD Z847801_0047 (
PULPROG zg30
TD 32768
SOLVENT CDCl3
NS 32
DS 0
SWH 9615.385 Hz
FIDRES 0.586877 Hz
AQ 1.7039360 sec
RG 80.6
DW 52.000 usec
DE 14.38 usec
TE 293.0 K
D1 1.00000000 sec
TD0 1
SFO1 599.9845606 MHz
NUC1 1H
P0 3.60 usec
P1 10.80 usec
PLW1 20.00000000 W
F2 - Processing parameters
SI 65536
SF 599.9800034 MHz
WDW EM
SSB 0
LB 0 Hz
GB 0
PC 1.00

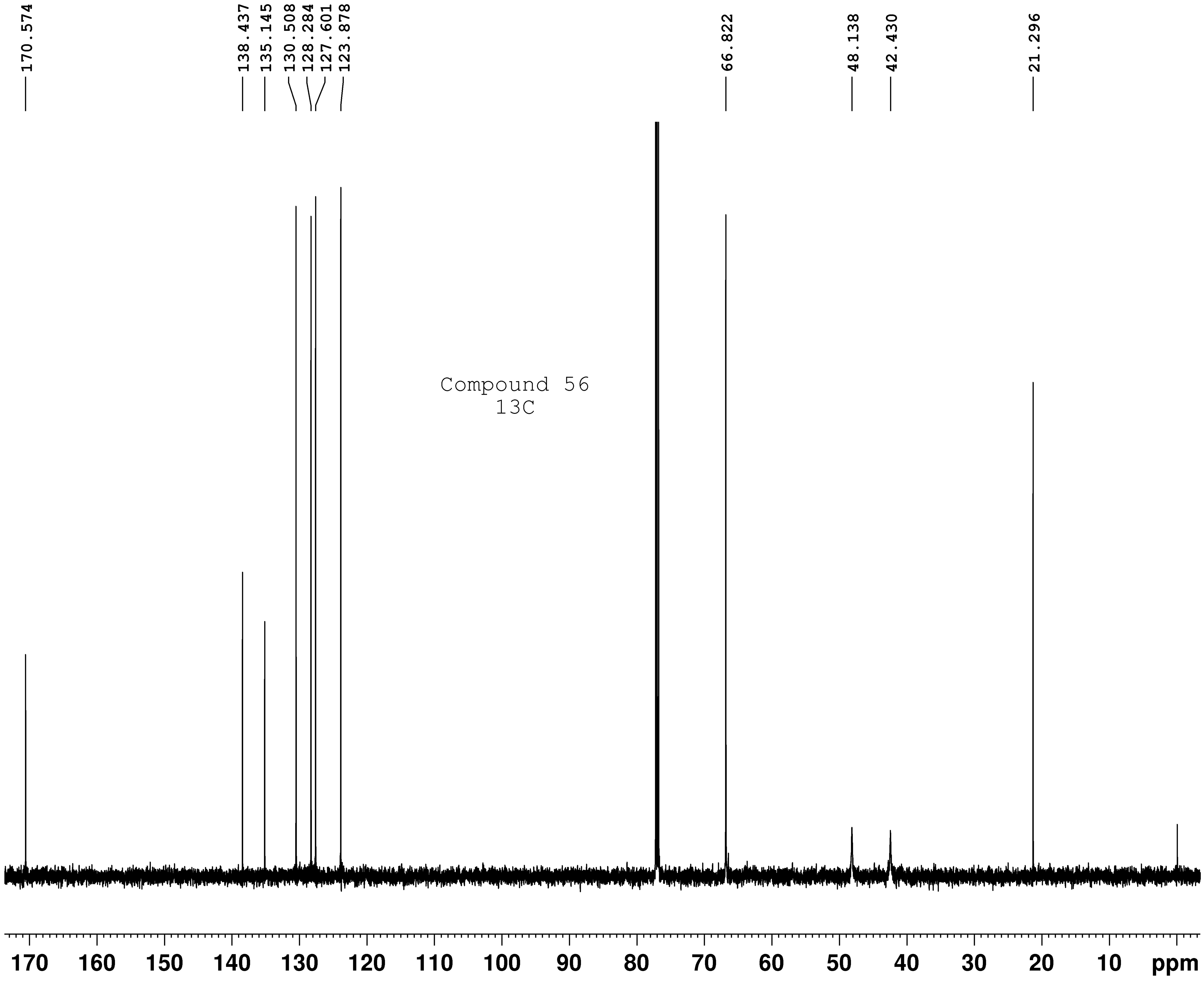


```
Current Data Parameters
NAME              CD-186A
EXPNO                  12
PROCNO                  1

F2 - Acquisition Parameters
Date_            20220112
Time                17.29 h
INSTRUM             spect
PROBHD   Z847801_0047 (
PULPROG            zgdc30
TD                  32768
SOLVENT             CDCl3
NS                    128
DS                      0
SWH             36057.691 Hz
FIDRES           2.200787 Hz
AQ              0.4543829 sec
RG                   2050
DW                 13.867 usec
DE                   6.50 usec
TE                  293.1 K
D1             1.50000000 sec
D11            0.03000000 sec
TD0                     1
SFO1          150.8816889 MHz
NUC1                  13C
P0                   3.27 usec
P1                   9.80 usec
PLW1          40.00000000 W
SFO2          599.9824003 MHz
NUC2                   1H
CPDPRG[2          waltz16
PCPD2               70.00 usec
PLW2          20.00000000 W
PLW12          0.53044999 W

F2 - Processing parameters
SI                  65536
SF            150.8651060 MHz
WDW                    EM
SSB                     0
LB                   1.00 Hz
GB                      0
PC                   1.40
```

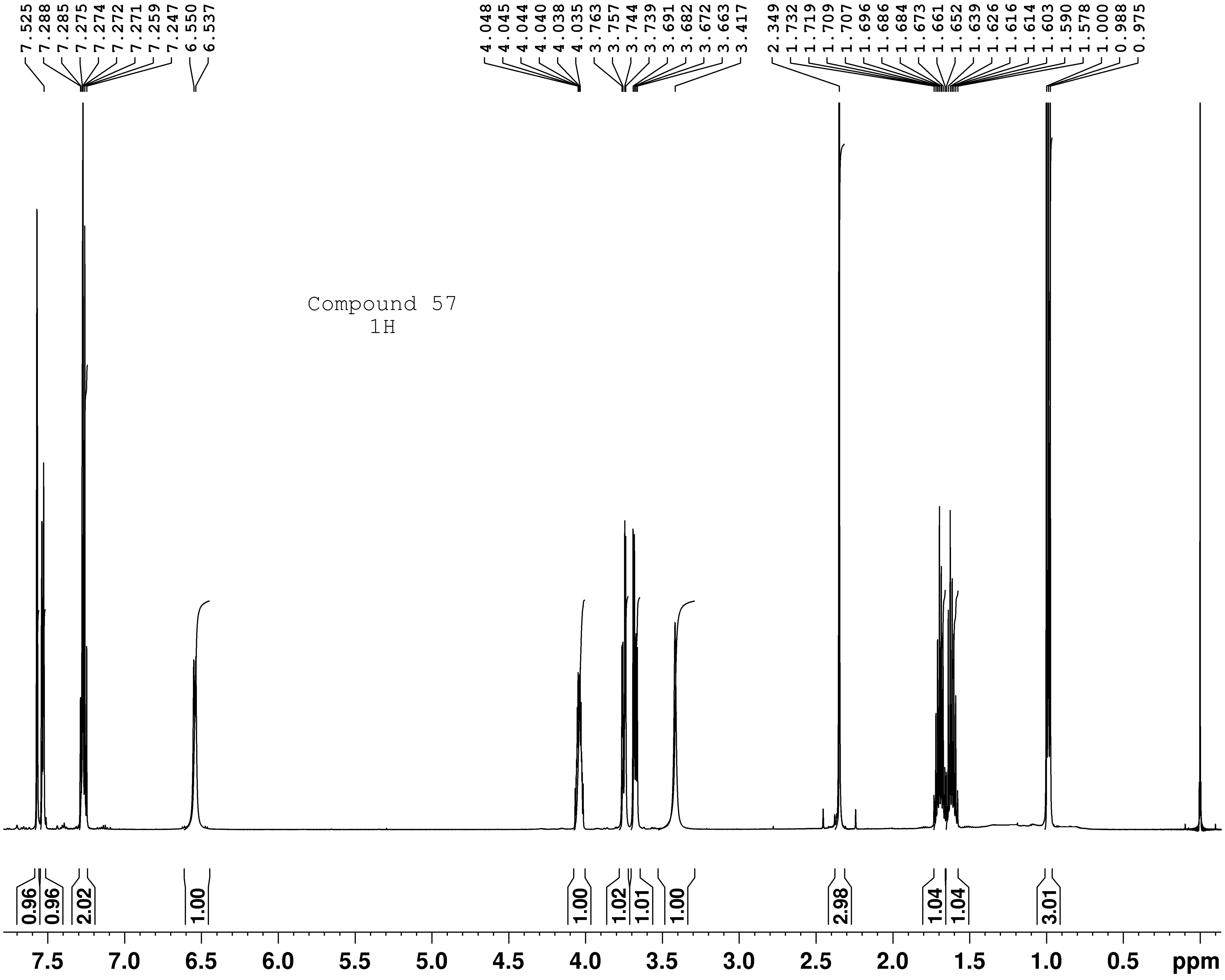

Current Data Parameters
NAME CE-15A
EXPNO 11
PROCNO 1

F2 - Acquisition Parameters
Date_ 20210219
Time 18.49 h
INSTRUM spect
PROBHD Z847801_0047 (
PULPROG zg30
TD 32768
SOLVENT CDCl3
NS 32
DS 0
SWH 9615.385 Hz
FIDRES 0.586877 Hz
AQ 1.7039360 sec
RG 50.8
DW 52.000 usec
DE 14.37 usec
TE 293.0 K
D1 1.00000000 sec
TD0 1
SFO1 600.0145608 MHz
NUC1 1H
P0 3.62 usec
P1 10.85 usec
PLW1 20.00000000 W

F2 - Processing parameters
SI 65536
SF 600.0100095 MHz
WDW EM
SSB 0
LB 0 Hz
GB 0
PC 1.00

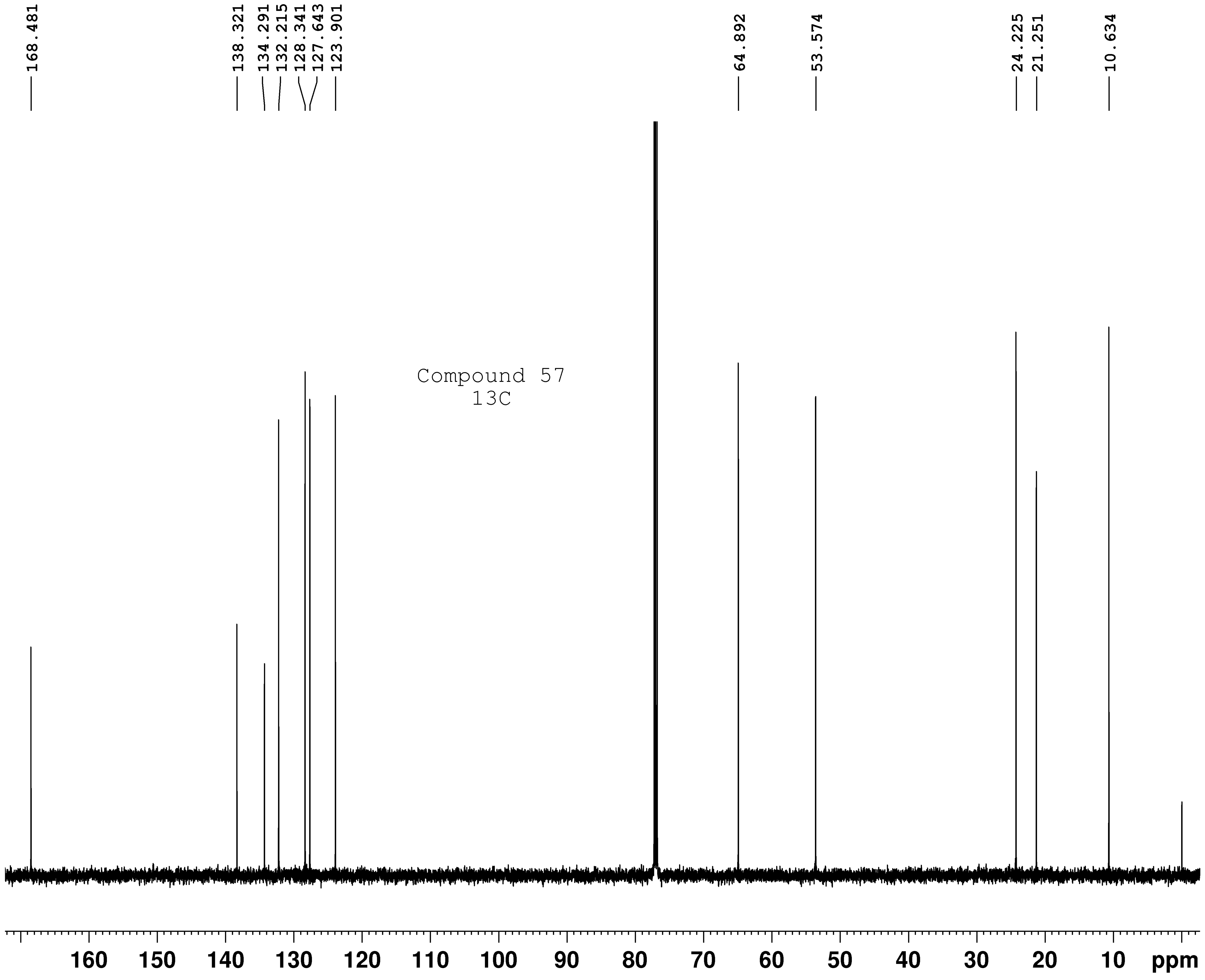


```
Current Data Parameters
NAME                 CE-15A
EXPNO                    16
PROCNO                    1

F2 - Acquisition Parameters
Date_              20210219
Time                  19.24 h
INSTRUM               spect
PROBHD   Z847801_0047 (
PULPROG             zgdc30
TD                    32768
SOLVENT               CDCl3
NS                      128
DS                        0
SWH              36057.691 Hz
FIDRES            2.200787 Hz
AQ               0.4543829 sec
RG                     2050
DW                   13.867 usec
DE                     6.50 usec
TE                    293.0 K
D1               1.50000000 sec
D11              0.03000000 sec
TD0                       1
SFO1           150.8892338 MHz
NUC1                    13C
P0                     3.27 usec
P1                     9.80 usec
PLW1            40.00000000 W
SFO2           600.0124004 MHz
NUC2                     1H
CPDPRG[2            waltz16
PCPD2                 90.00 usec
PLW2            20.00000000 W
PLW12            0.33800000 W

F2 - Processing parameters
SI                    65536
SF             150.8726471 MHz
WDW                      EM
SSB                       0
LB                     1.00 Hz
GB                        0
PC                     1.40
```

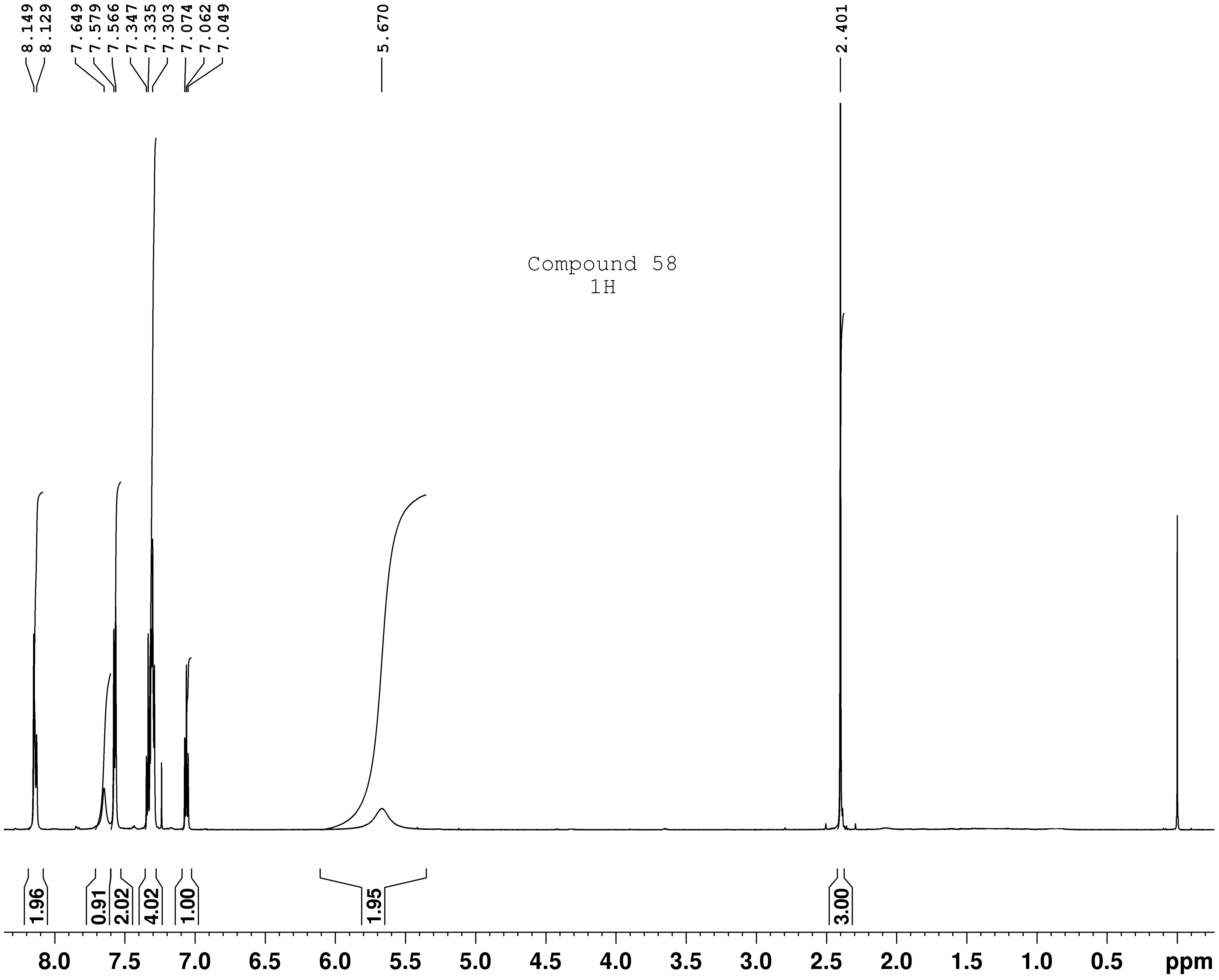


```
Current Data Parameters
NAME              CE-14A
EXPNO                 11
PROCNO                 1

F2 - Acquisition Parameters
Date_           20210222
Time               17.54 h
INSTRUM            spect
PROBHD   Z847801_0047 (
PULPROG             zg30
TD                 32768
SOLVENT            CDCl3
NS                     1
DS                     0
SWH             9615.385 Hz
FIDRES          0.586877 Hz
AQ             1.7039360 sec
RG                  50.8
DW                52.000 usec
DE                 14.37 usec
TE                 293.0 K
D1            1.00000000 sec
TD0                    1
SFO1         600.0145608 MHz
NUC1                  1H
P0                  3.62 usec
P1                 10.85 usec
PLW1         20.00000000 W

F2 - Processing parameters
SI                 65536
SF           600.0100278 MHz
WDW                   EM
SSB                    0
LB                     0 Hz
GB                     0
PC                  1.00
```

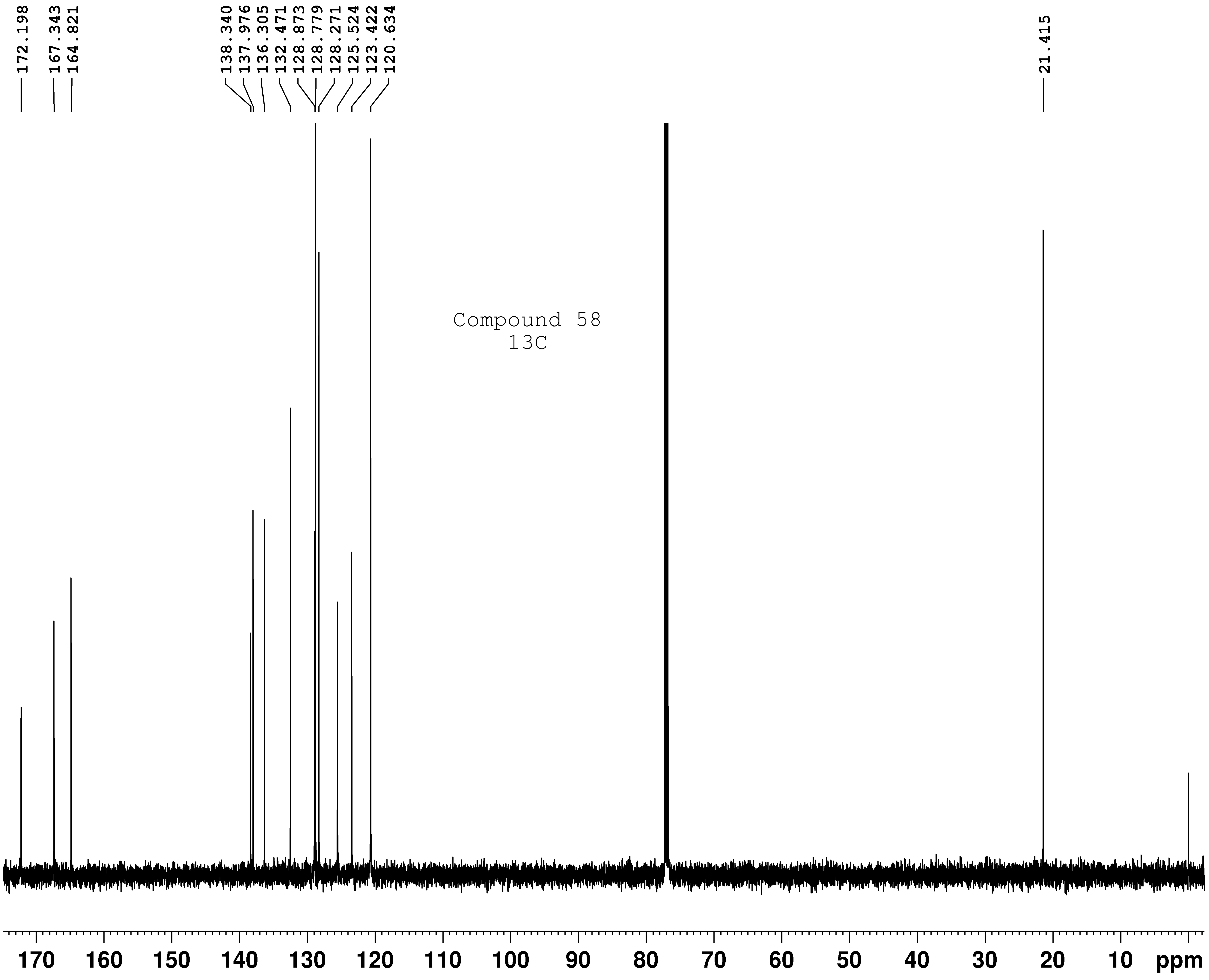


```
Current Data Parameters
NAME               CE-14A
EXPNO                  16
PROCNO                  1

F2 - Acquisition Parameters
Date_            20210222
Time                18.29 h
INSTRUM             spect
PROBHD   Z847801_0047 (
PULPROG           zgdc30
TD                 32768
SOLVENT            CDCl3
NS                    128
DS                      0
SWH            36057.691 Hz
FIDRES          2.200787 Hz
AQ             0.4543829 sec
RG                   2050
DW                 13.867 usec
DE                   6.50 usec
TE                  293.1 K
D1             1.50000000 sec
D11            0.03000000 sec
TD0                     1
SFO1         150.8892338 MHz
NUC1                  13C
P0                   3.27 usec
P1                   9.80 usec
PLW1          40.00000000 W
SFO2         600.0124004 MHz
NUC2                   1H
CPDPRG[2          waltz16
PCPD2               90.00 usec
PLW2          20.00000000 W
PLW12          0.33800000 W

F2 - Processing parameters
SI                  65536
SF           150.8726505 MHz
WDW                    EM
SSB                     0
LB                   1.00 Hz
GB                      0
PC                   1.40
```

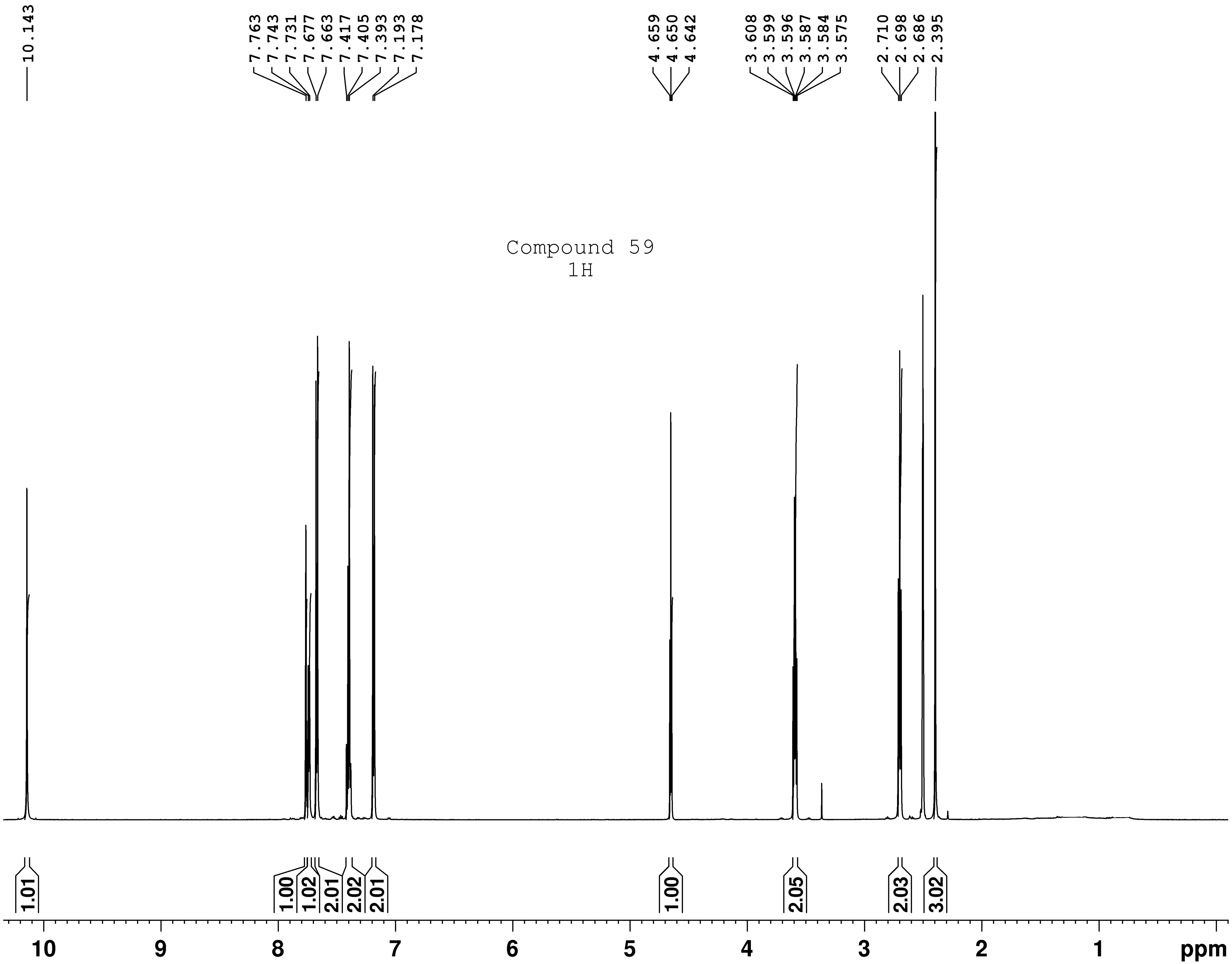

Current Data Parameters
NAME CE-03A
EXPNO 11
PROCNO 1

F2 - Acquisition Parameters
Date_ 20210217
Time 19.20 h
INSTRUM spect
PROBHD Z847801_0047 (
PULPROG zg30
TD 32768
SOLVENT DMSO
NS 32
DS 0
SWH 9615.385 Hz
FIDRES 0.586877 Hz
AQ 1.7039360 sec
RG 64
DW 52.000 usec
DE 14.37 usec
TE 293.0 K
D1 1.00000000 sec
TD0 1
SFO1 600.0145608 MHz
NUC1 1H
P0 3.62 usec
P1 10.85 usec
PLW1 20.00000000 W

F2 - Processing parameters
SI 65536
SF 600.0100050 MHz
WDW EM
SSB 0
LB 0 Hz
GB 0
PC 1.00

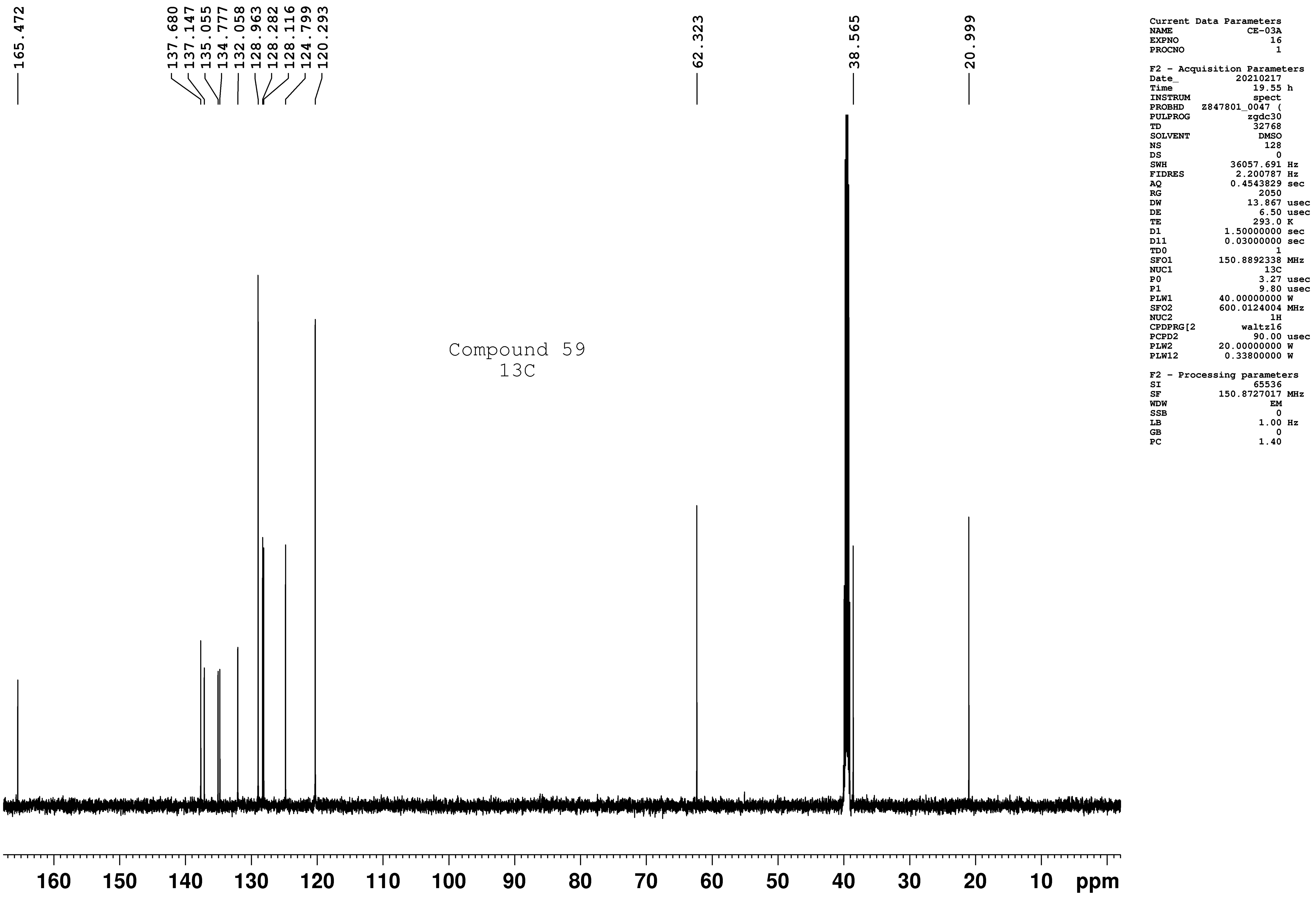
165.472
137.680
137.147
135.055
134.777
132.058
128.963
128.282
128.116
124.799
120.293
62.323
38.565
20.999
Compound 59
13C
160 150 140 130 120 110 100 90 80 70 60 50 40 30 20 10 ppm
Current Data Parameters
NAME CE-03A
EXPNO 16
PROCNO 1
F2 - Acquisition Parameters
Date_ 20210217
Time 19.55 h
INSTRUM spect
PROBHD Z847801_0047 (
PULPROG zgdc30
TD 32768
SOLVENT DMSO
NS 128
DS 0
SWH 36057.691 Hz
FIDRES 2.200787 Hz
AQ 0.4543829 sec
RG 2050
DW 13.867 usec
DE 6.50 usec
TE 293.0 K
D1 1.50000000 sec
D11 0.03000000 sec
TD0 1
SFO1 150.8892338 MHz
NUC1 13C
P0 3.27 usec
P1 9.80 usec
PLW1 40.00000000 W
SFO2 600.0124004 MHz
NUC2 1H
CPDPRG[2 waltz16
PCPD2 90.00 usec
PLW2 20.00000000 W
PLW12 0.33800000 W
F2 - Processing parameters
SI 65536
SF 150.8727017 MHz
WDW EM
SSB 0
LB 1.00 Hz
GB 0
PC 1.40

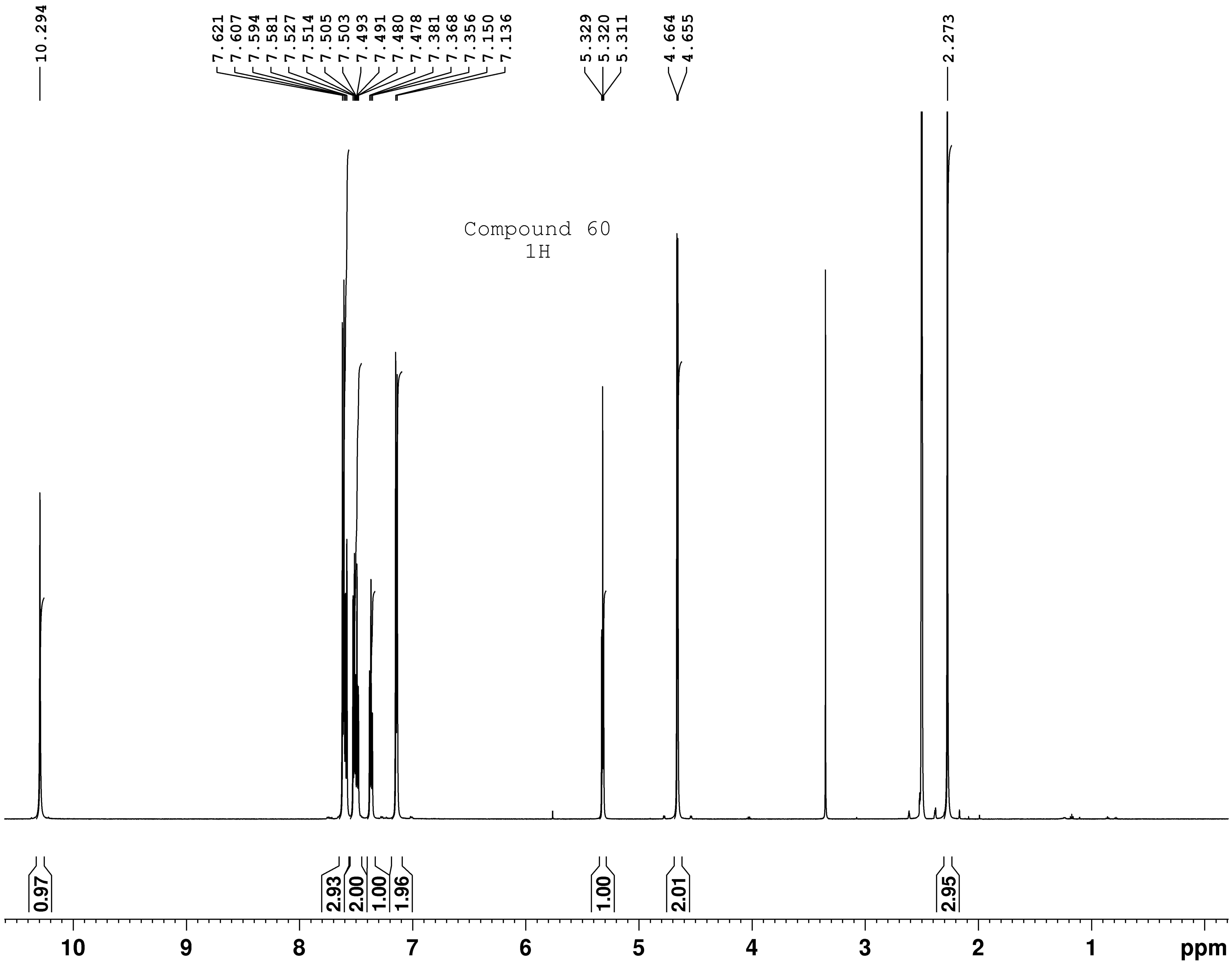

Current Data Parameters		
NAME	CE-08A	
EXPNO	11	
PROCNO	1	
F2 - Acquisition Parameters		
Date_	20210428	
Time	1.01	h
INSTRUM	spect	
PROBHD	Z847801_0047 (	
PULPROG	zg30	
TD	32768	
SOLVENT	DMSO	
NS	32	
DS	0	
SWH	9615.385	Hz
FIDRES	0.586877	Hz
AQ	1.7039360	sec
RG	144	
DW	52.000	usec
DE	14.37	usec
TE	293.0	K
D1	1.00000000	sec
TD0	1	
SFO1	600.0145608	MHz
NUC1	1H	
P0	3.62	usec
P1	10.85	usec
PLW1	20.00000000	W
F2 - Processing parameters		
SI	65536	
SF	600.0100048	MHz
WDW	EM	
SSB	0	
LB	0	Hz
GB	0	
PC	1.00	

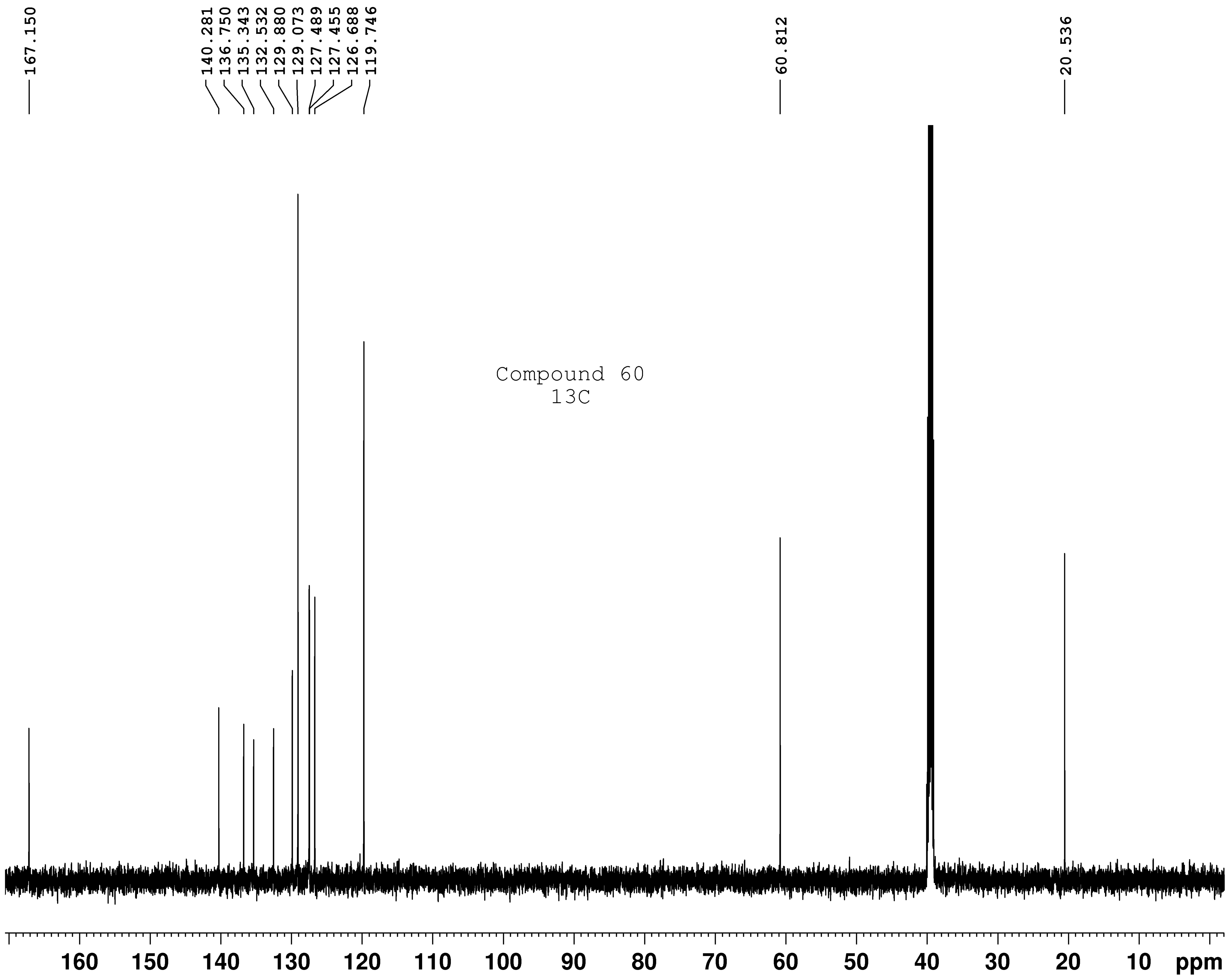


```
Current Data Parameters
NAME             CE-08A
EXPNO                12
PROCNO                1

F2 - Acquisition Parameters
Date_          20210428
Time             1.06 h
INSTRUM           spect
PROBHD   Z847801_0047 (
PULPROG         zgdc30
TD               32768
SOLVENT            DMSO
NS                  128
DS                    0
SWH          36057.691 Hz
FIDRES        2.200787 Hz
AQ           0.4543829 sec
RG                 2050
DW              13.867 usec
DE                6.50 usec
TE               293.0 K
D1          1.50000000 sec
D11         0.03000000 sec
TD0                   1
SFO1       150.8892338 MHz
NUC1                13C
P0                3.27 usec
P1                9.80 usec
PLW1       40.00000000 W
SFO2       600.0124004 MHz
NUC2                 1H
CPDPRG[2        waltz16
PCPD2            90.00 usec
PLW2       20.00000000 W
PLW12       0.33800000 W

F2 - Processing parameters
SI                65536
SF         150.8727011 MHz
WDW                  EM
SSB                   0
LB                1.00 Hz
GB                    0
PC                 1.40
```

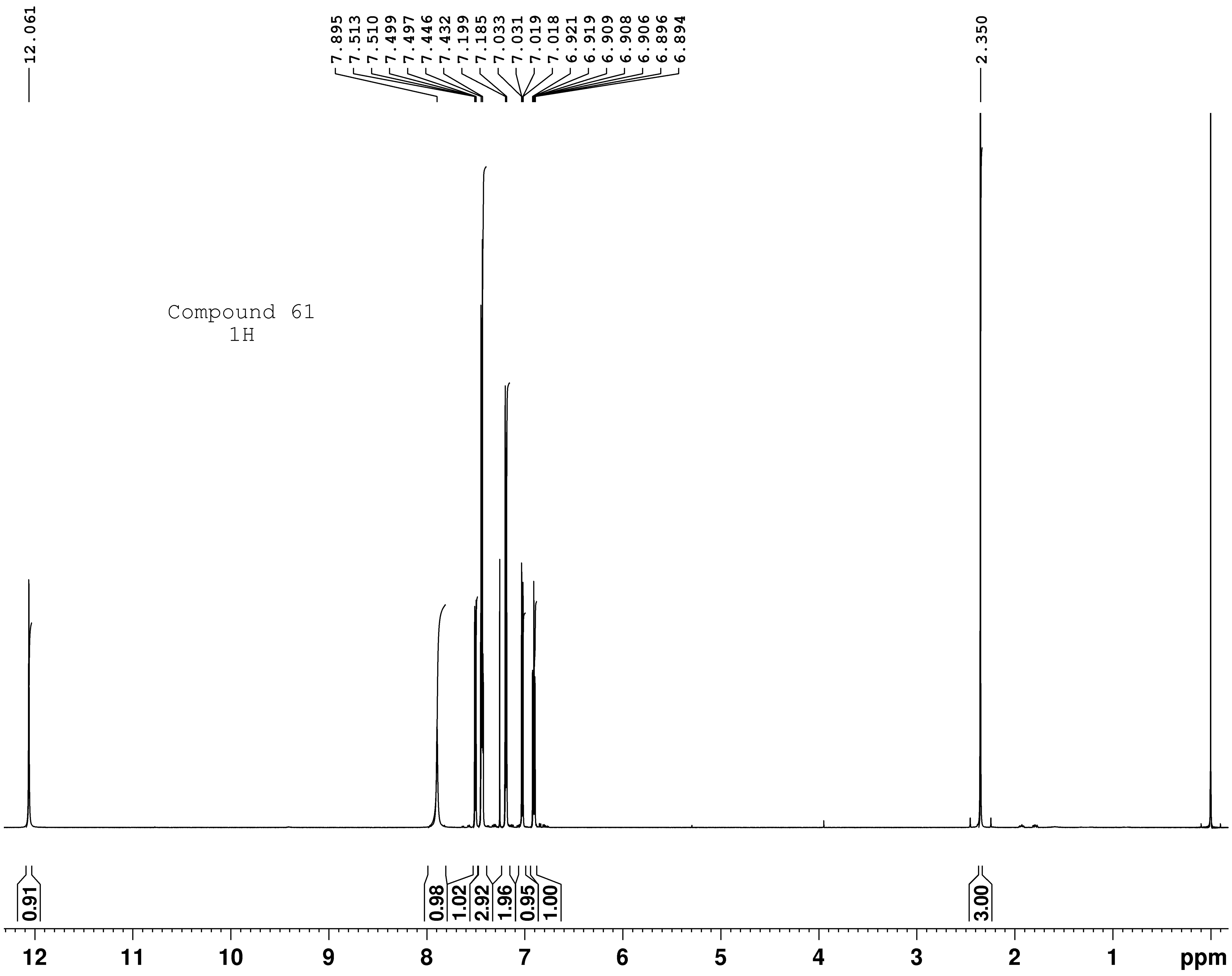

Current Data Parameters
NAME 61-CD-203A
EXPNO 11
PROCNO 1

F2 - Acquisition Parameters
Date_ 20220118
Time 18.02 h
INSTRUM spect
PROBHD Z847801_0047 (
PULPROG zg30
TD 32768
SOLVENT CDCl3
NS 32
DS 0
SWH 9615.385 Hz
FIDRES 0.586877 Hz
AQ 1.7039360 sec
RG 203
DW 52.000 usec
DE 14.38 usec
TE 293.0 K
D1 1.00000000 sec
TD0 1
SFO1 599.9845606 MHz
NUC1 1H
P0 3.60 usec
P1 10.80 usec
PLW1 20.00000000 W

F2 - Processing parameters
SI 65536
SF 599.9800167 MHz
WDW EM
SSB 0
LB 0 Hz
GB 0
PC 1.00

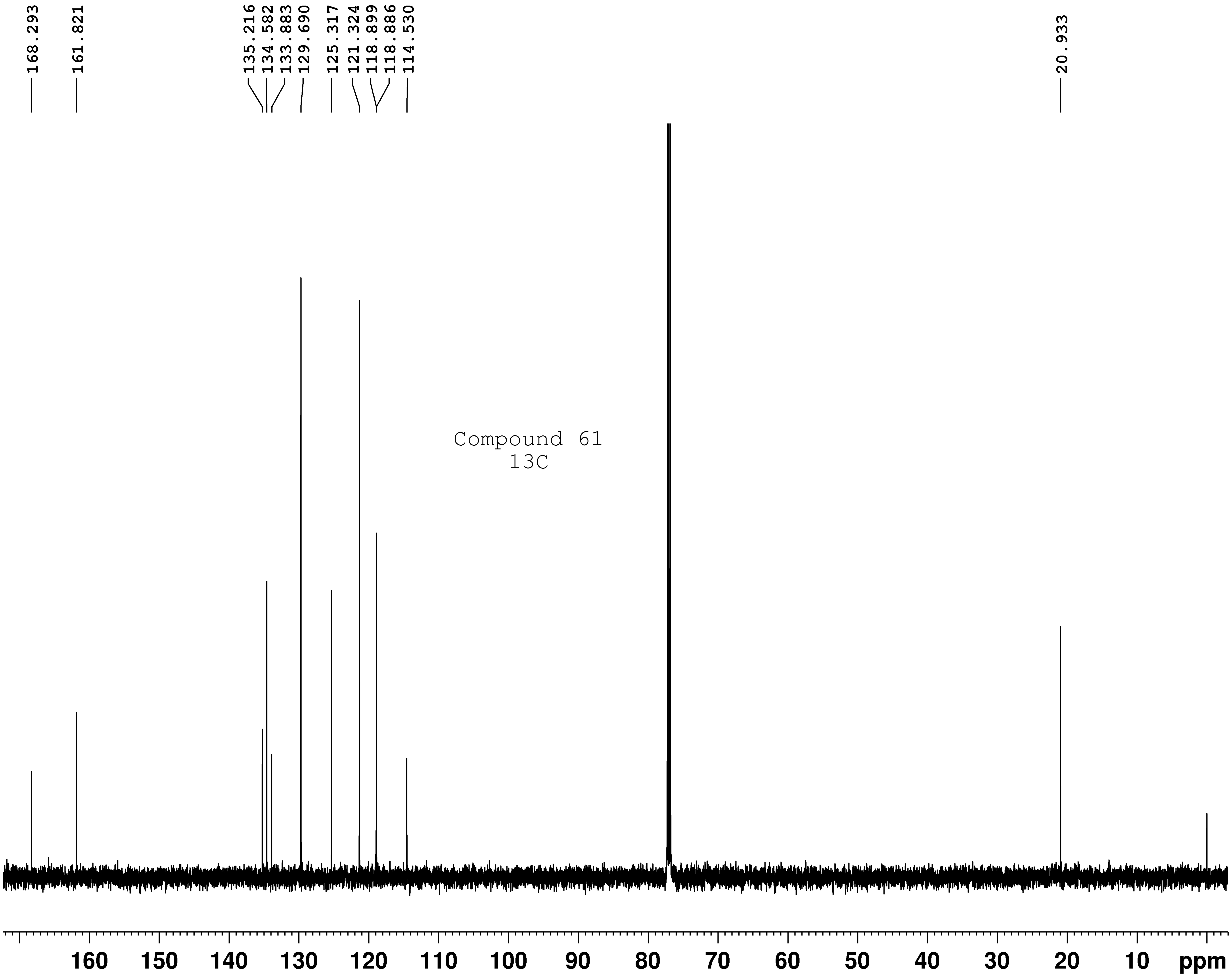


```
Current Data Parameters
NAME              CD-203A
EXPNO                  12
PROCNO                  1

F2 - Acquisition Parameters
Date_            20220118
Time                18.07 h
INSTRUM             spect
PROBHD   Z847801_0047 (
PULPROG           zgdc30
TD                 32768
SOLVENT            CDCl3
NS                   128
DS                     0
SWH            36057.691 Hz
FIDRES          2.200787 Hz
AQ             0.4543829 sec
RG                  2050
DW                13.867 usec
DE                  6.50 usec
TE                 293.1 K
D1            1.50000000 sec
D11           0.03000000 sec
TD0                    1
SFO1         150.8816889 MHz
NUC1                 13C
P0                  3.27 usec
P1                  9.80 usec
PLW1         40.00000000 W
SFO2         599.9824003 MHz
NUC2                  1H
CPDPRG[2         waltz16
PCPD2              70.00 usec
PLW2         20.00000000 W
PLW12         0.53044999 W

F2 - Processing parameters
SI                 65536
SF           150.8650988 MHz
WDW                   EM
SSB                    0
LB                  1.00 Hz
GB                     0
PC                  1.40
```

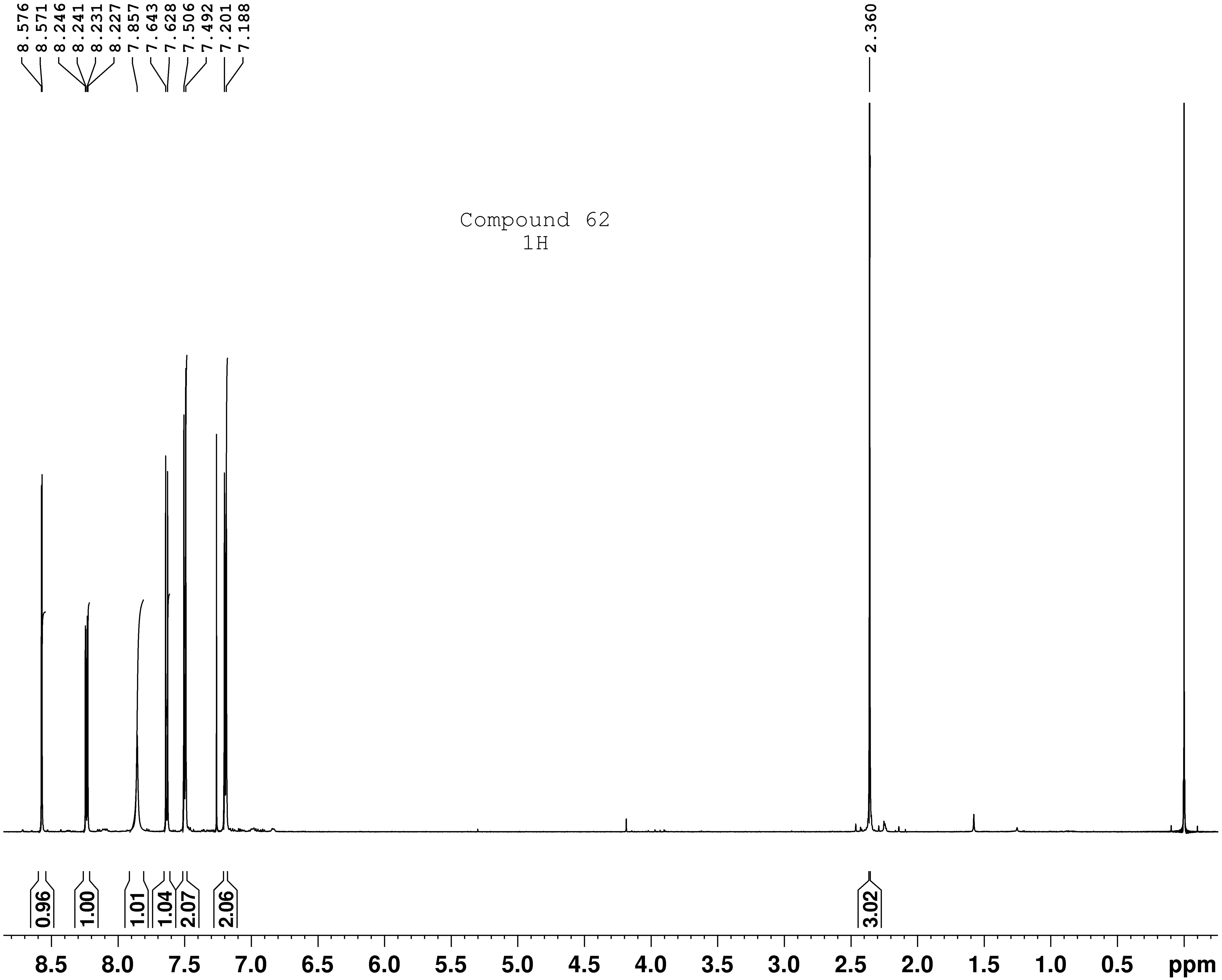


```
Current Data Parameters
NAME          62-CD-198A
EXPNO                 11
PROCNO                 1

F2 - Acquisition Parameters
Date_           20220118
Time               17.45 h
INSTRUM            spect
PROBHD   Z847801_0047 (
PULPROG             zg30
TD                 32768
SOLVENT            CDCl3
NS                    32
DS                     0
SWH             9615.385 Hz
FIDRES          0.586877 Hz
AQ             1.7039360 sec
RG                   287
DW                52.000 usec
DE                 14.38 usec
TE                 293.0 K
D1            1.00000000 sec
TD0                    1
SFO1         599.9845606 MHz
NUC1                  1H
P0                  3.60 usec
P1                 10.80 usec
PLW1         20.00000000 W

F2 - Processing parameters
SI                 65536
SF           599.9800131 MHz
WDW                   EM
SSB                    0
LB                     0 Hz
GB                     0
PC                  1.00
```

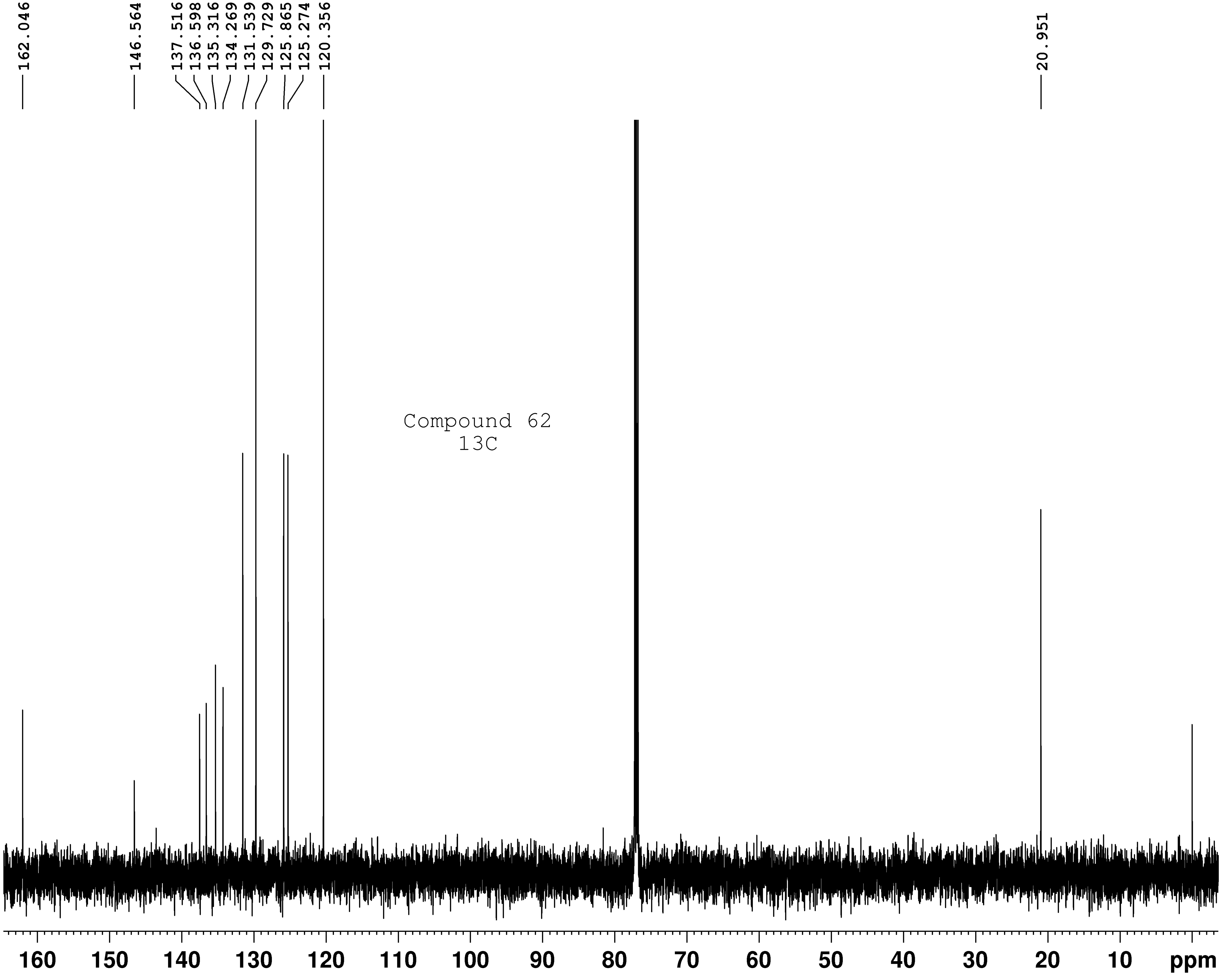


```
Current Data Parameters
NAME              CD-198A
EXPNO                  12
PROCNO                  1

F2 - Acquisition Parameters
Date_            20220118
Time                17.50 h
INSTRUM             spect
PROBHD   Z847801_0047 (
PULPROG           zgdc30
TD                  32768
SOLVENT             CDCl3
NS                    128
DS                      0
SWH             36057.691 Hz
FIDRES           2.200787 Hz
AQ              0.4543829 sec
RG                   2050
DW                 13.867 usec
DE                   6.50 usec
TE                  293.1 K
D1             1.50000000 sec
D11            0.03000000 sec
TD0                     1
SFO1          150.8816889 MHz
NUC1                  13C
P0                   3.27 usec
P1                   9.80 usec
PLW1          40.00000000 W
SFO2          599.9824003 MHz
NUC2                   1H
CPDPRG[2          waltz16
PCPD2               70.00 usec
PLW2          20.00000000 W
PLW12          0.53044999 W

F2 - Processing parameters
SI                  65536
SF            150.8650977 MHz
WDW                    EM
SSB                     0
LB                   1.00 Hz
GB                      0
PC                   1.40
```

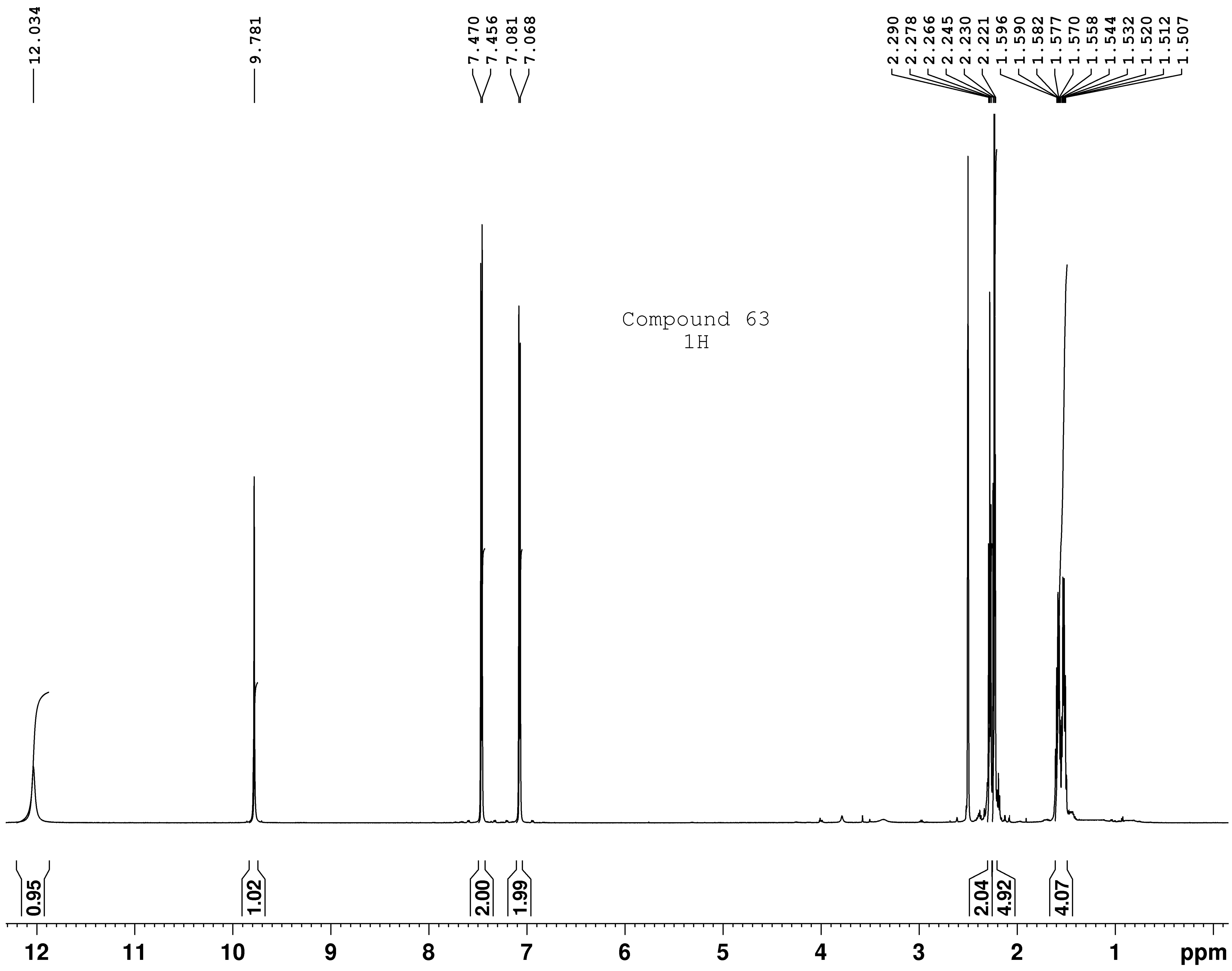

Current Data Parameters

NAME	63. CD-199A	
EXPNO	11	
PROCNO	1	

F2 - Acquisition Parameters

Date_	20210219	
Time	18.02	h
INSTRUM	spect	
PROBHD	Z847801_0047 (	
PULPROG	zg30	
TD	32768	
SOLVENT	DMSO	
NS	32	
DS	0	
SWH	9615.385	Hz
FIDRES	0.586877	Hz
AQ	1.7039360	sec
RG	57	
DW	52.000	usec
DE	14.37	usec
TE	293.0	K
D1	1.00000000	sec
TD0	1	
SFO1	600.0145608	MHz
NUC1	1H	
P0	3.62	usec
P1	10.85	usec
PLW1	20.00000000	W

F2 - Processing parameters

SI	65536	
SF	600.0100049	MHz
WDW	EM	
SSB	0	
LB	0	Hz
GB	0	
PC	1.00	

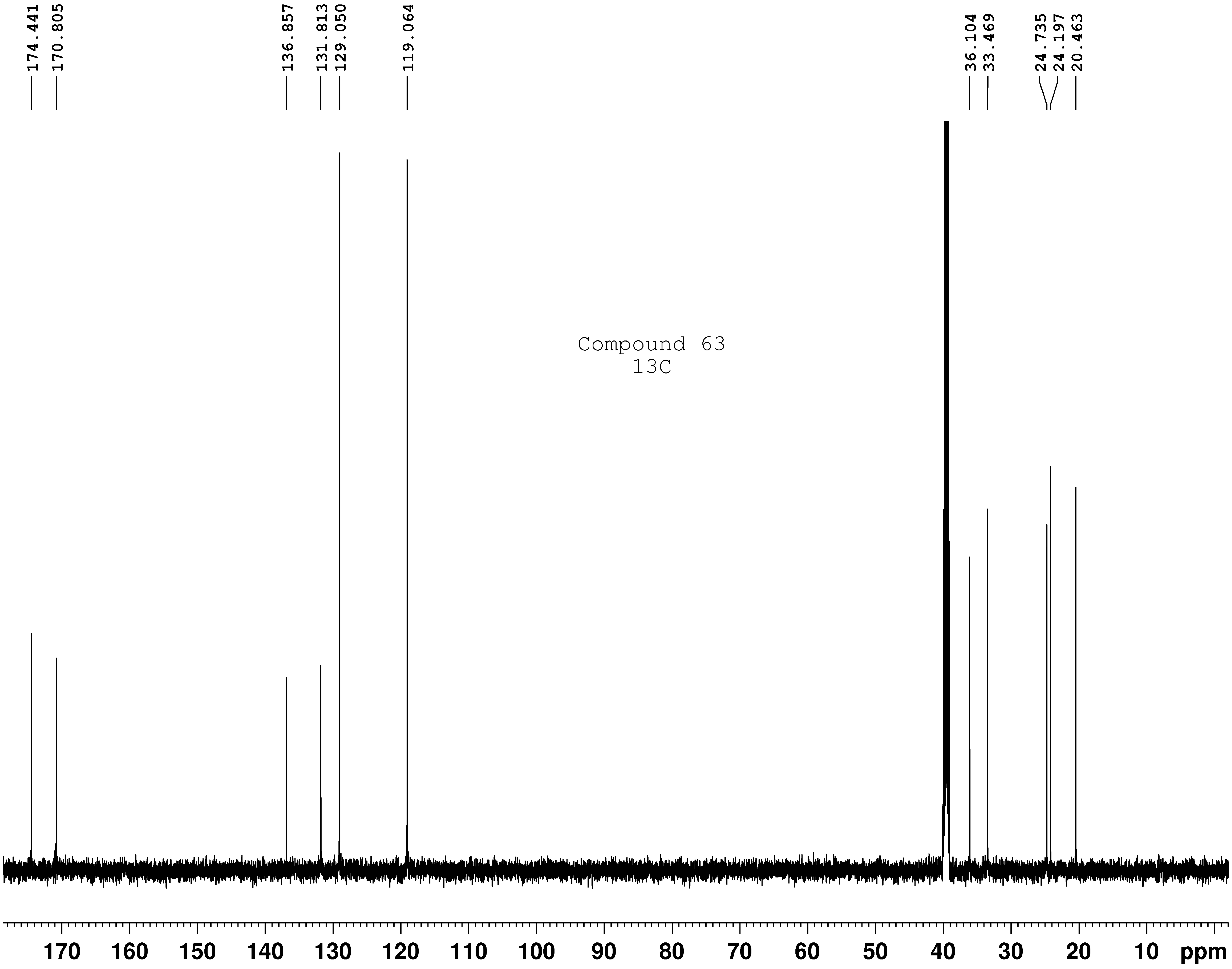

Compound 63
13C

```
Current Data Parameters
NAME              CD-199A
EXPNO                  16
PROCNO                  1

F2 - Acquisition Parameters
Date_            20210219
Time                18.36 h
INSTRUM             spect
PROBHD   Z847801_0047 (
PULPROG           zgdc30
TD                  32768
SOLVENT              DMSO
NS                    128
DS                      0
SWH             36057.691 Hz
FIDRES           2.200787 Hz
AQ              0.4543829 sec
RG                   2050
DW                 13.867 usec
DE                   6.50 usec
TE                  293.0 K
D1             1.50000000 sec
D11            0.03000000 sec
TD0                     1
SFO1          150.8892338 MHz
NUC1                  13C
P0                   3.27 usec
P1                   9.80 usec
PLW1          40.00000000 W
SFO2          600.0124004 MHz
NUC2                   1H
CPDPRG[2          waltz16
PCPD2               90.00 usec
PLW2          20.00000000 W
PLW12          0.33800000 W

F2 - Processing parameters
SI                  65536
SF            150.8726989 MHz
WDW                    EM
SSB                     0
LB                   1.00 Hz
GB                      0
PC                   1.40
```

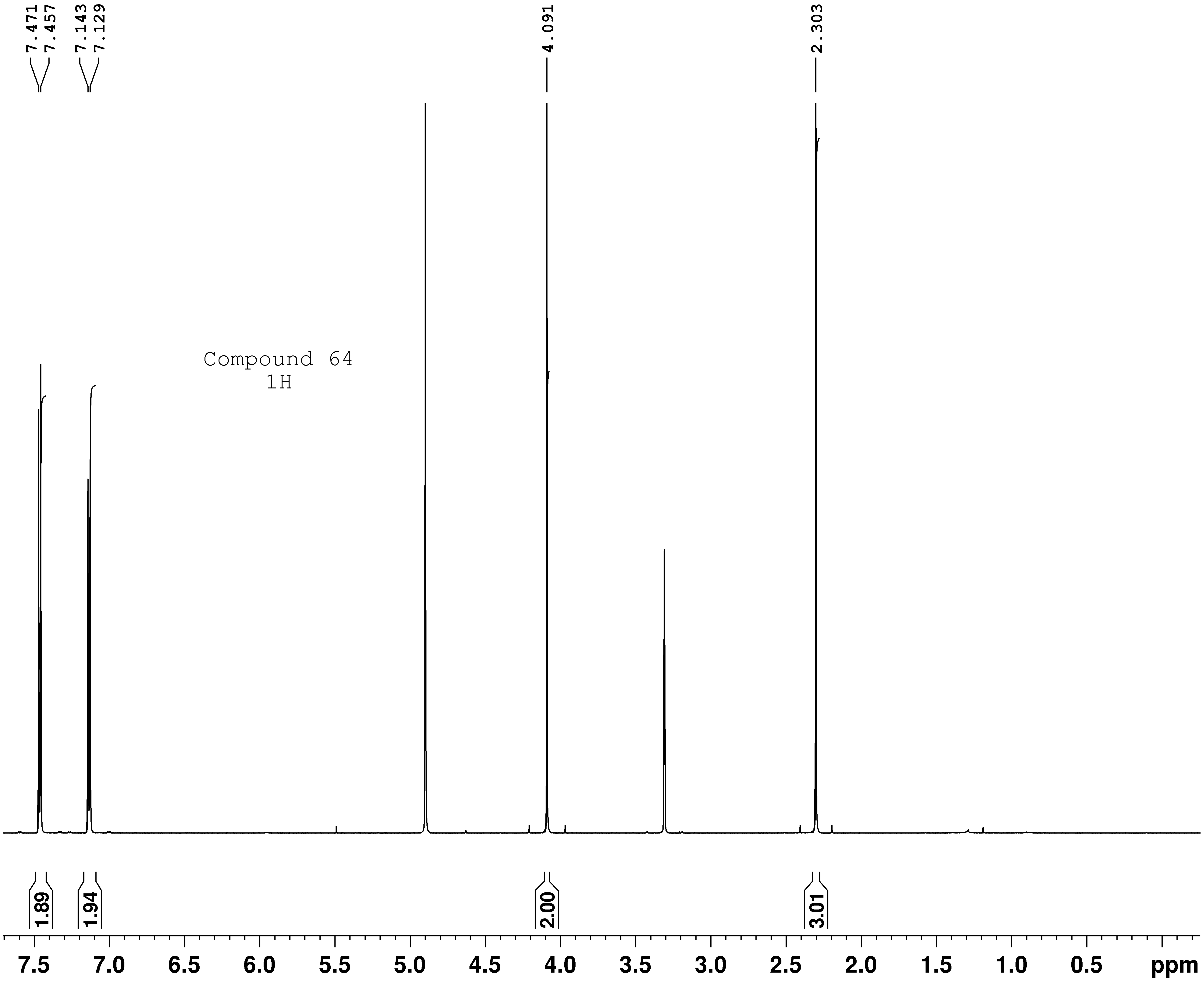


```
Current Data Parameters
NAME            CD-200A
EXPNO                11
PROCNO                1

F2 - Acquisition Parameters
Date_          20220224
Time              19.39 h
INSTRUM           spect
PROBHD   Z847801_0047 (
PULPROG            zg30
TD                32768
SOLVENT            MeOD
NS                   16
DS                    0
SWH            9615.385 Hz
FIDRES         0.586877 Hz
AQ            1.7039360 sec
RG                  256
DW               52.000 usec
DE                14.38 usec
TE                293.0 K
D1           1.00000000 sec
TD0                   1
SFO1        599.9845606 MHz
NUC1                 1H
P0                 3.60 usec
P1                10.80 usec
PLW1        20.00000000 W

F2 - Processing parameters
SI                65536
SF          599.9800114 MHz
WDW                  EM
SSB                   0
LB                    0 Hz
GB                    0
PC                 1.00
```

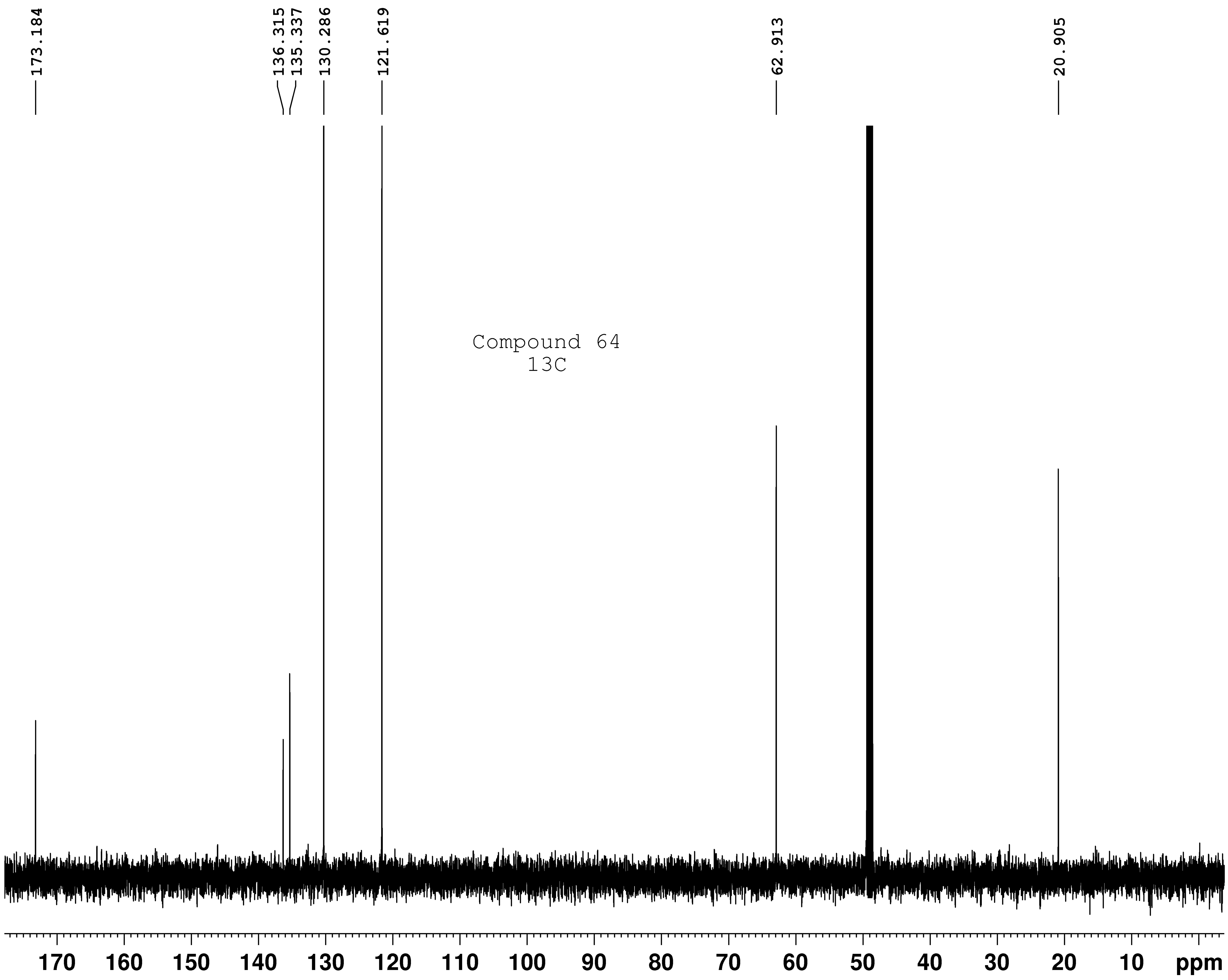


```
Current Data Parameters
NAME             CD-200A
EXPNO                 12
PROCNO                 1

F2 - Acquisition Parameters
Date_           20220224
Time               19.44 h
INSTRUM            spect
PROBHD   Z847801_0047 (
PULPROG          zgdc30
TD                 32768
SOLVENT             MeOD
NS                   128
DS                     0
SWH            36057.691 Hz
FIDRES          2.200787 Hz
AQ             0.4543829 sec
RG                  2050
DW                13.867 usec
DE                  6.50 usec
TE                 293.0 K
D1            1.50000000 sec
D11           0.03000000 sec
TD0                    1
SFO1         150.8816889 MHz
NUC1                 13C
P0                  3.27 usec
P1                  9.80 usec
PLW1         40.00000000 W
SFO2         599.9824003 MHz
NUC2                  1H
CPDPRG[2         waltz16
PCPD2              70.00 usec
PLW2         20.00000000 W
PLW12         0.53044999 W

F2 - Processing parameters
SI                 65536
SF           150.8648802 MHz
WDW                   EM
SSB                    0
LB                  1.00 Hz
GB                     0
PC                  1.40
```

Compound 70
1H

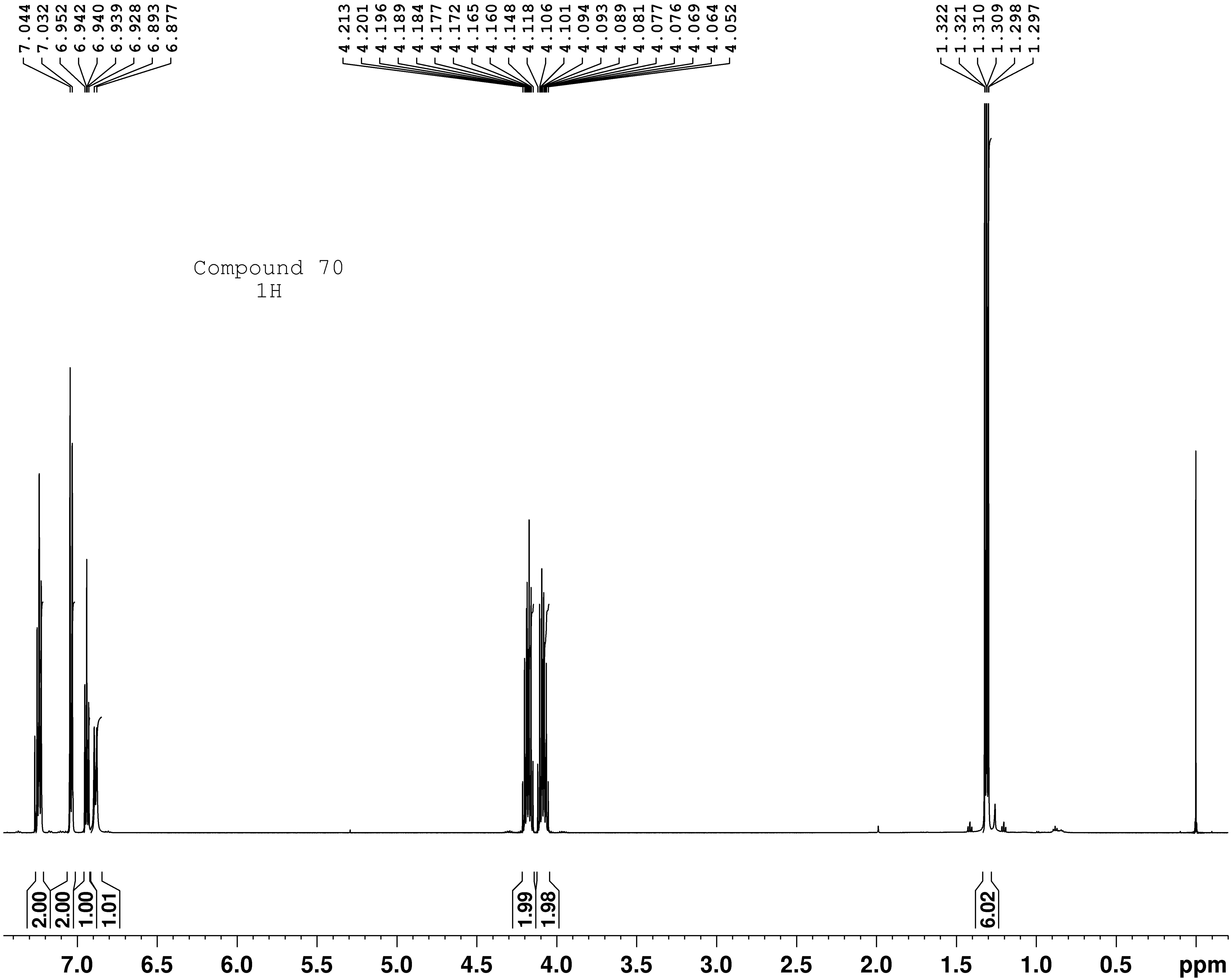

Current Data Parameters
NAME 70-CE-110A
EXPNO 11
PROCNO 1

F2 - Acquisition Parameters
Date_ 20220118
Time 18.36 h
INSTRUM spect
PROBHD Z847801_0047 (
PULPROG zg30
TD 32768
SOLVENT CDCl3
NS 32
DS 0
SWH 9615.385 Hz
FIDRES 0.586877 Hz
AQ 1.7039360 sec
RG 80.6
DW 52.000 usec
DE 14.38 usec
TE 293.0 K
D1 1.00000000 sec
TD0 1
SFO1 599.9845606 MHz
NUC1 1H
P0 3.60 usec
P1 10.80 usec
PLW1 20.00000000 W

F2 - Processing parameters
SI 65536
SF 599.9800107 MHz
WDW EM
SSB 0
LB 0 Hz
GB 0
PC 1.00

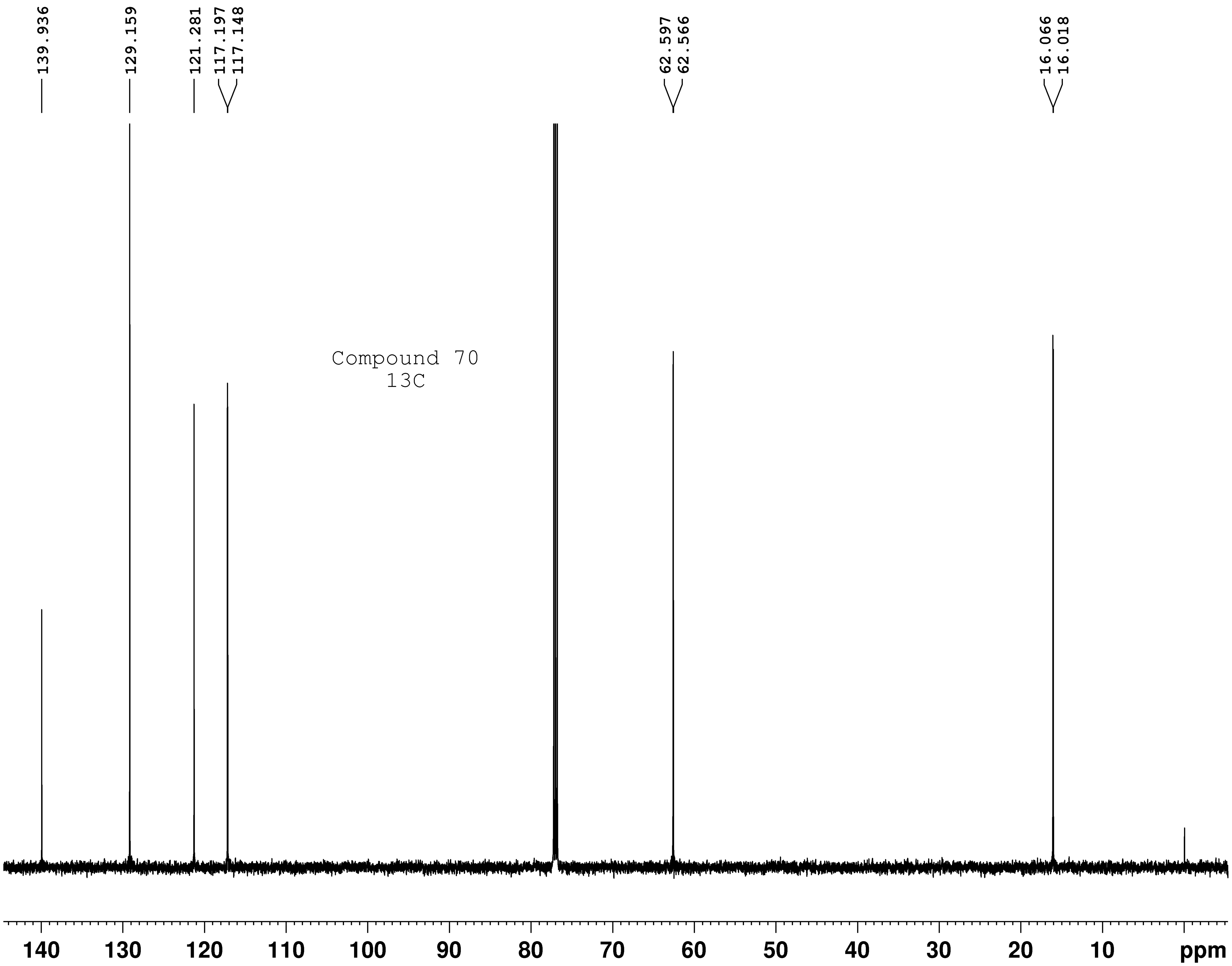

Current Data Parameters
NAME CE-110A
EXPNO 12
PROCNO 1

F2 - Acquisition Parameters
Date_ 20220118
Time 18.41 h
INSTRUM spect
PROBHD Z847801_0047 (
PULPROG zgdc30
TD 32768
SOLVENT CDCl3
NS 128
DS 0
SWH 36057.691 Hz
FIDRES 2.200787 Hz
AQ 0.4543829 sec
RG 2050
DW 13.867 usec
DE 6.50 usec
TE 293.1 K
D1 1.50000000 sec
D11 0.03000000 sec
TD0 1
SFO1 150.8816889 MHz
NUC1 13C
P0 3.27 usec
P1 9.80 usec
PLW1 40.00000000 W
SFO2 599.9824003 MHz
NUC2 1H
CPDPRG[2 waltz16
PCPD2 70.00 usec
PLW2 20.00000000 W
PLW12 0.53044999 W

F2 - Processing parameters
SI 65536
SF 150.8651021 MHz
WDW EM
SSB 0
LB 1.00 Hz
GB 0
PC 1.40

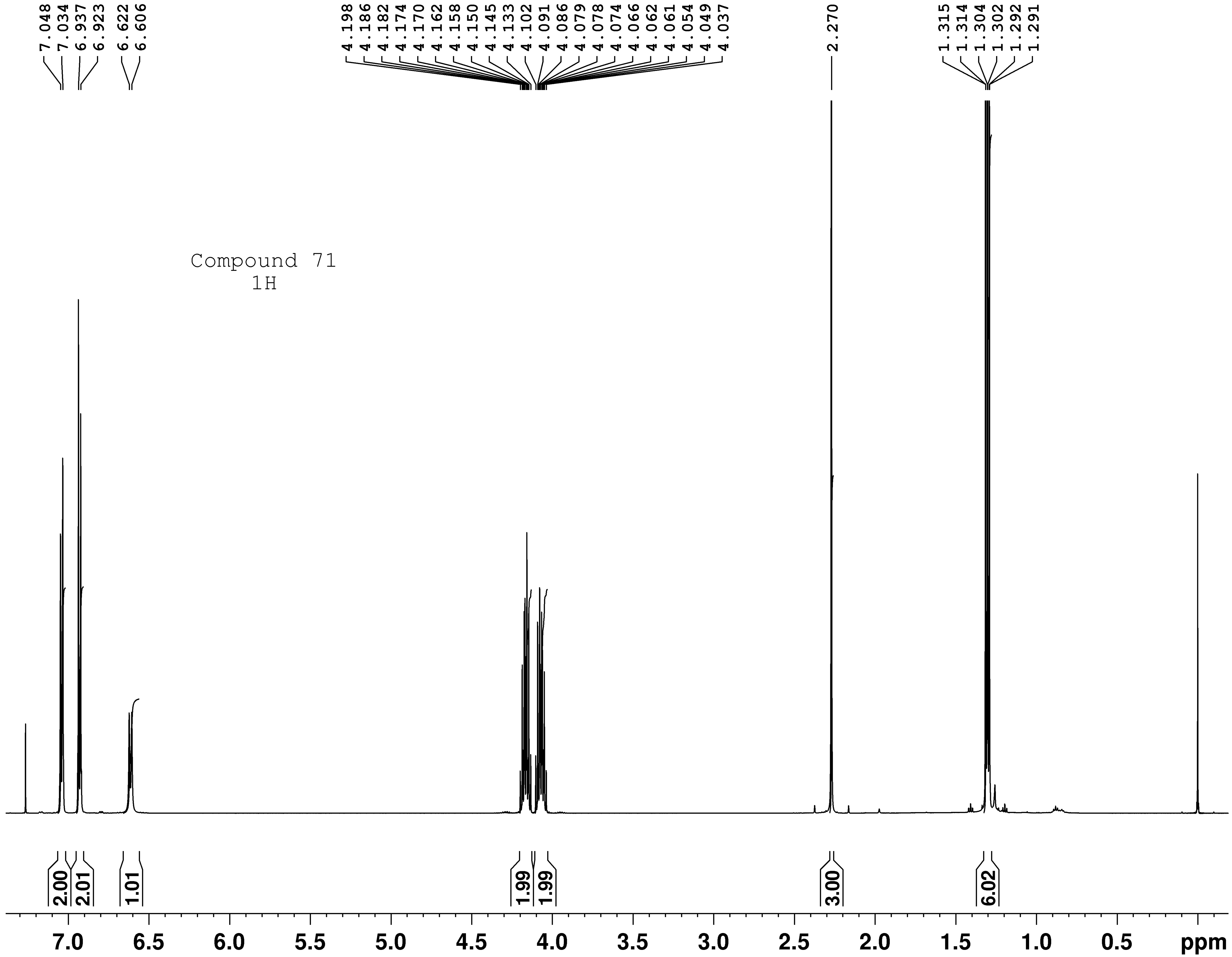

Current Data Parameters
NAME 71-CE-66A
EXPNO 11
PROCNO 1

F2 - Acquisition Parameters
Date_ 20220118
Time 18.19 h
INSTRUM spect
PROBHD Z847801_0047 (
PULPROG zg30
TD 32768
SOLVENT CDCl3
NS 32
DS 0
SWH 9615.385 Hz
FIDRES 0.586877 Hz
AQ 1.7039360 sec
RG 80.6
DW 52.000 usec
DE 14.38 usec
TE 293.0 K
D1 1.00000000 sec
TD0 1
SFO1 599.9845606 MHz
NUC1 1H
P0 3.60 usec
P1 10.80 usec
PLW1 20.00000000 W

F2 - Processing parameters
SI 65536
SF 599.9800111 MHz
WDW EM
SSB 0
LB 0 Hz
GB 0
PC 1.00

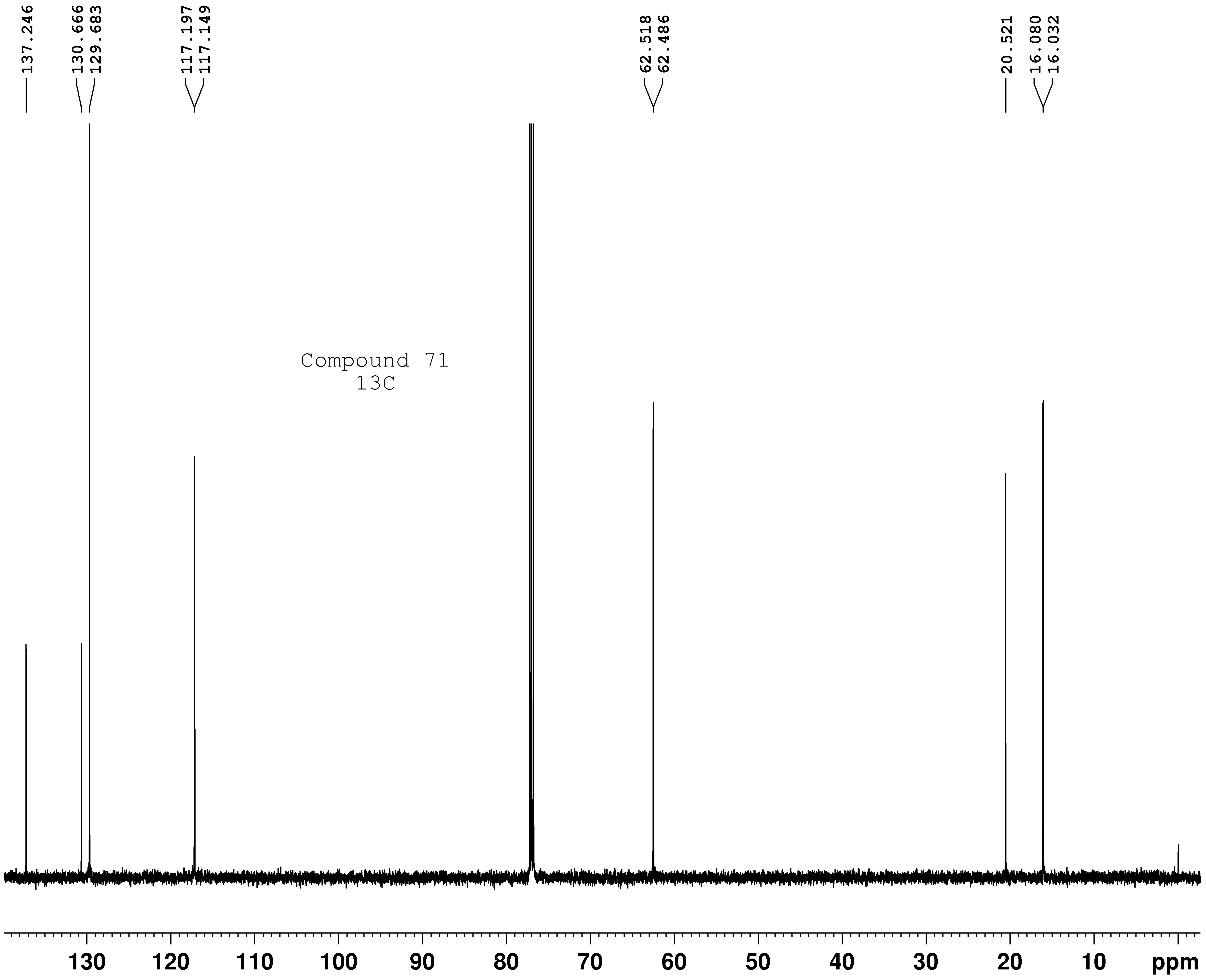

Current Data Parameters
NAME CE-66A
EXPNO 12
PROCNO 1

F2 - Acquisition Parameters
Date_ 20220118
Time 18.25 h
INSTRUM spect
PROBHD Z847801_0047 (
PULPROG zgdc30
TD 32768
SOLVENT CDCl3
NS 128
DS 0
SWH 36057.691 Hz
FIDRES 2.200787 Hz
AQ 0.4543829 sec
RG 2050
DW 13.867 usec
DE 6.50 usec
TE 293.1 K
D1 1.50000000 sec
D11 0.03000000 sec
TD0 1
SFO1 150.8816889 MHz
NUC1 13C
P0 3.27 usec
P1 9.80 usec
PLW1 40.00000000 W
SFO2 599.9824003 MHz
NUC2 1H
CPDPRG[2 waltz16
PCPD2 70.00 usec
PLW2 20.00000000 W
PLW12 0.53044999 W

F2 - Processing parameters
SI 65536
SF 150.8651015 MHz
WDW EM
SSB 0
LB 1.00 Hz
GB 0
PC 1.40

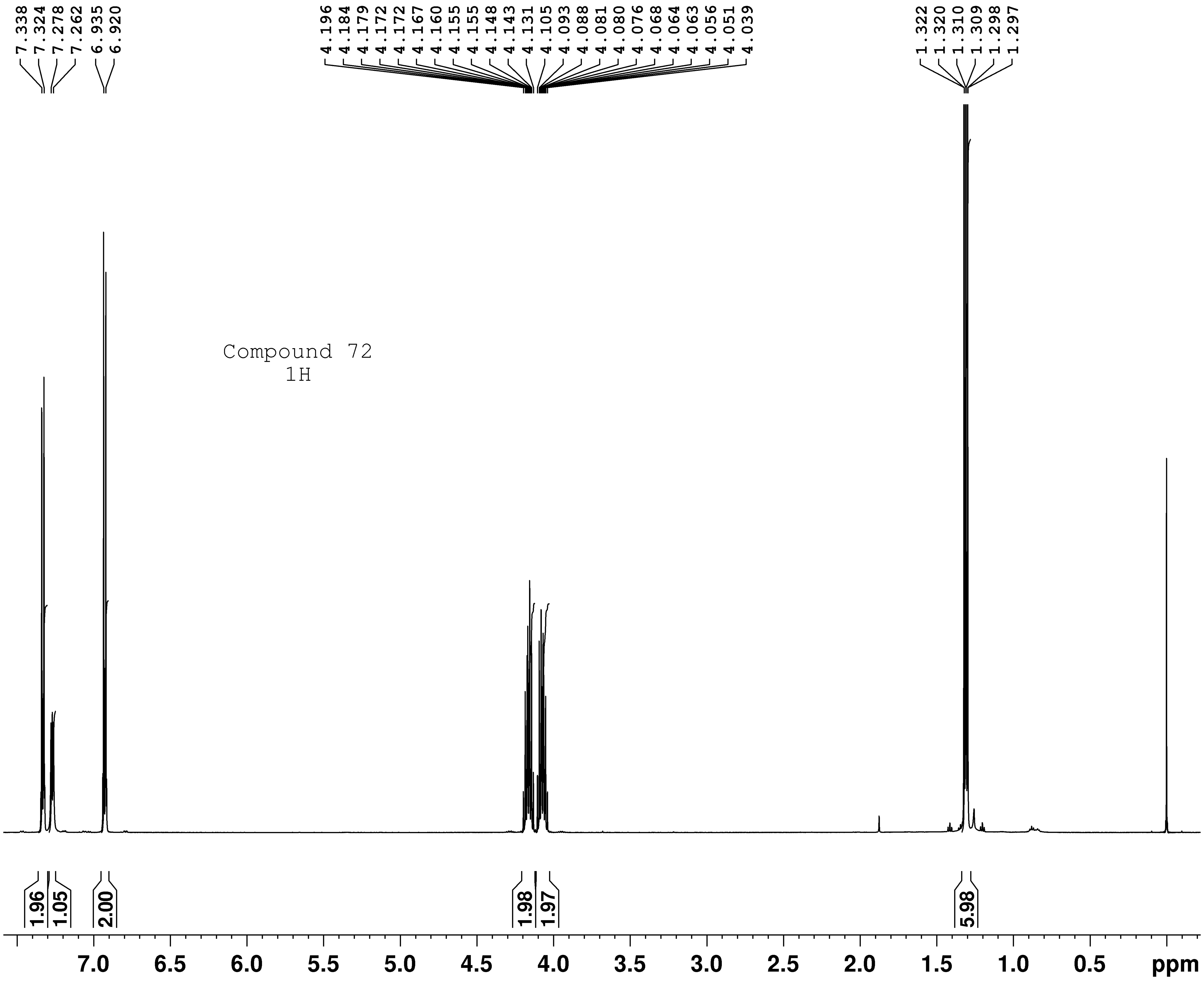

Current Data Parameters
NAME CE-108A
EXPNO 11
PROCNO 1

F2 - Acquisition Parameters
Date_ 20220221
Time 17.52 h
INSTRUM spect
PROBHD Z847801_0047 (
PULPROG zg30
TD 32768
SOLVENT CDCl3
NS 32
DS 0
SWH 9615.385 Hz
FIDRES 0.586877 Hz
AQ 1.7039360 sec
RG 101
DW 52.000 usec
DE 14.38 usec
TE 293.0 K
D1 1.00000000 sec
TD0 1
SFO1 599.9845606 MHz
NUC1 1H
P0 3.60 usec
P1 10.80 usec
PLW1 20.00000000 W

F2 - Processing parameters
SI 65536
SF 599.9800078 MHz
WDW EM
SSB 0
LB 0 Hz
GB 0
PC 1.00

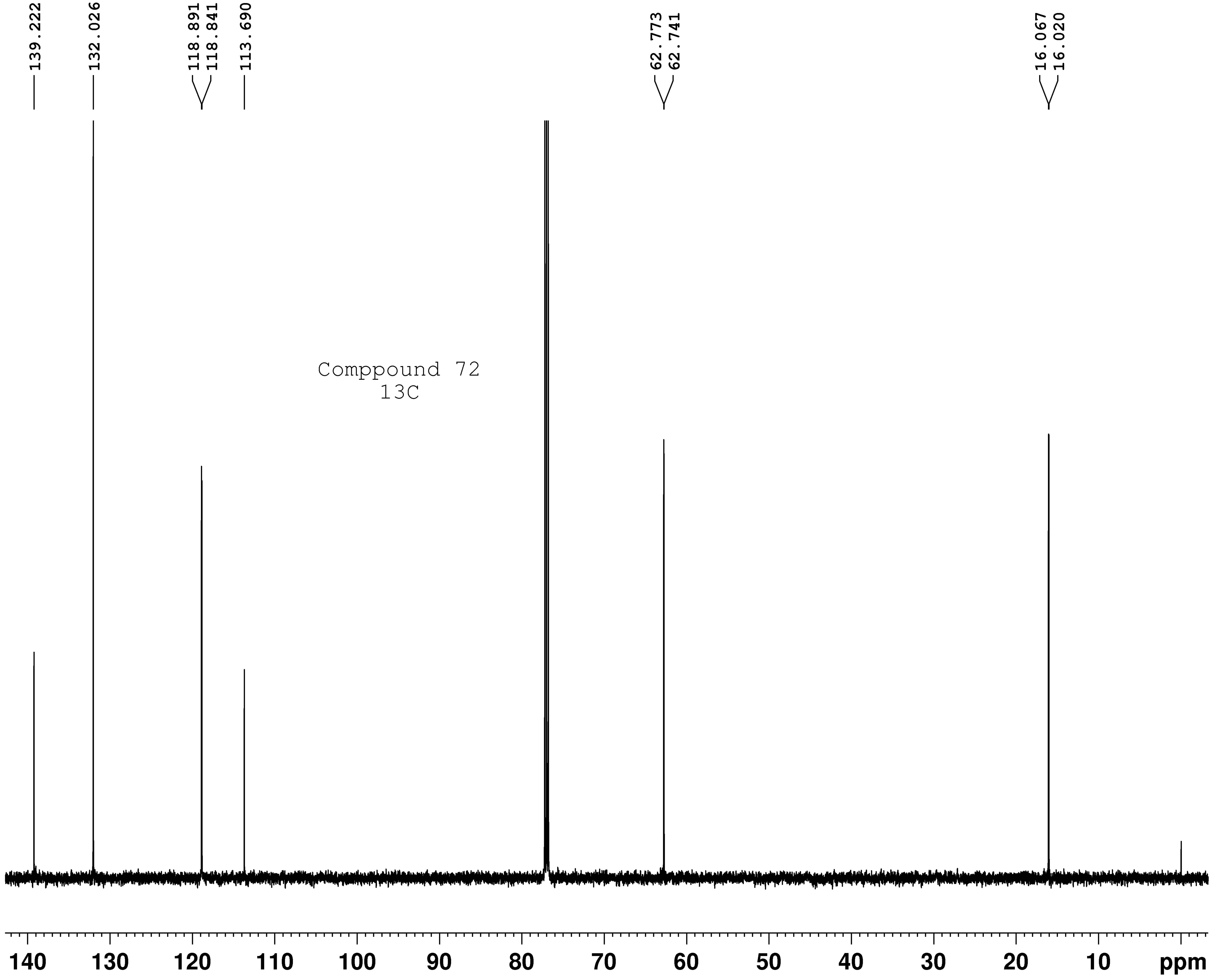


```
Current Data Parameters
NAME            CE-108A
EXPNO                12
PROCNO                1

F2 - Acquisition Parameters
Date_          20220221
Time              17.57 h
INSTRUM           spect
PROBHD   Z847801_0047 (
PULPROG          zgdc30
TD                32768
SOLVENT           CDCl3
NS                  128
DS                    0
SWH           36057.691 Hz
FIDRES         2.200787 Hz
AQ            0.4543829 sec
RG                 2050
DW               13.867 usec
DE                 6.50 usec
TE                293.0 K
D1           1.50000000 sec
D11          0.03000000 sec
TD0                   1
SFO1        150.8816889 MHz
NUC1                13C
P0                 3.27 usec
P1                 9.80 usec
PLW1        40.00000000 W
SFO2        599.9824003 MHz
NUC2                 1H
CPDPRG[2        waltz16
PCPD2             70.00 usec
PLW2        20.00000000 W
PLW12        0.53044999 W

F2 - Processing parameters
SI                65536
SF          150.8651001 MHz
WDW                  EM
SSB                   0
LB                 1.00 Hz
GB                    0
PC                 1.40
```

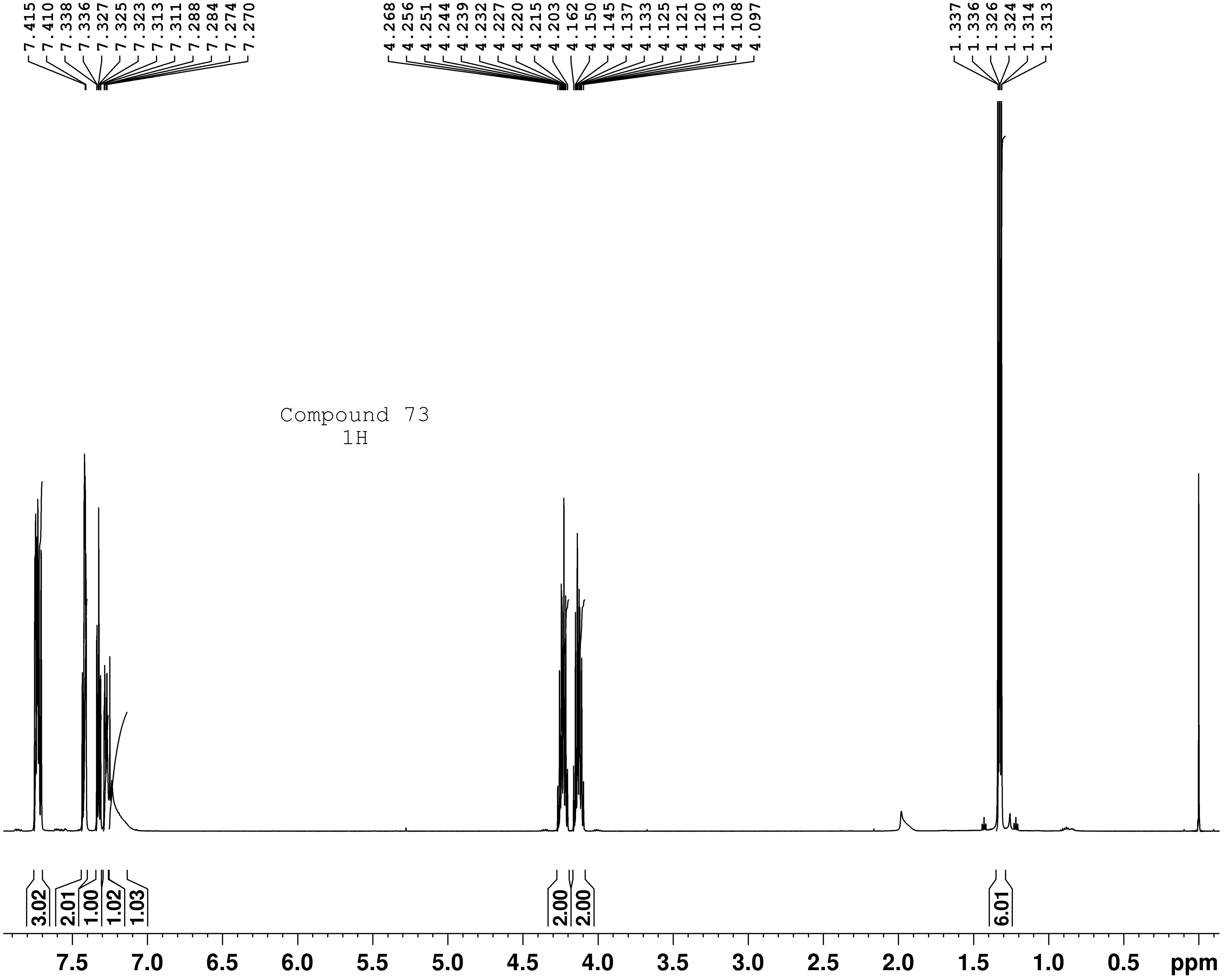

Current Data Parameters
NAME CE-107A
EXPNO 11
PROCNO 1

F2 - Acquisition Parameters
Date_ 20220221
Time 17.36 h
INSTRUM spect
PROBHD Z847801_0047 (
PULPROG zg30
TD 32768
SOLVENT CDCl3
NS 32
DS 0
SWH 9615.385 Hz
FIDRES 0.586877 Hz
AQ 1.7039360 sec
RG 90.5
DW 52.000 usec
DE 14.38 usec
TE 293.0 K
D1 1.00000000 sec
TD0 1
SFO1 599.9845606 MHz
NUC1 1H
P0 3.60 usec
P1 10.80 usec
PLW1 20.00000000 W

F2 - Processing parameters
SI 65536
SF 599.9800195 MHz
WDW EM
SSB 0
LB 0 Hz
GB 0
PC 1.00

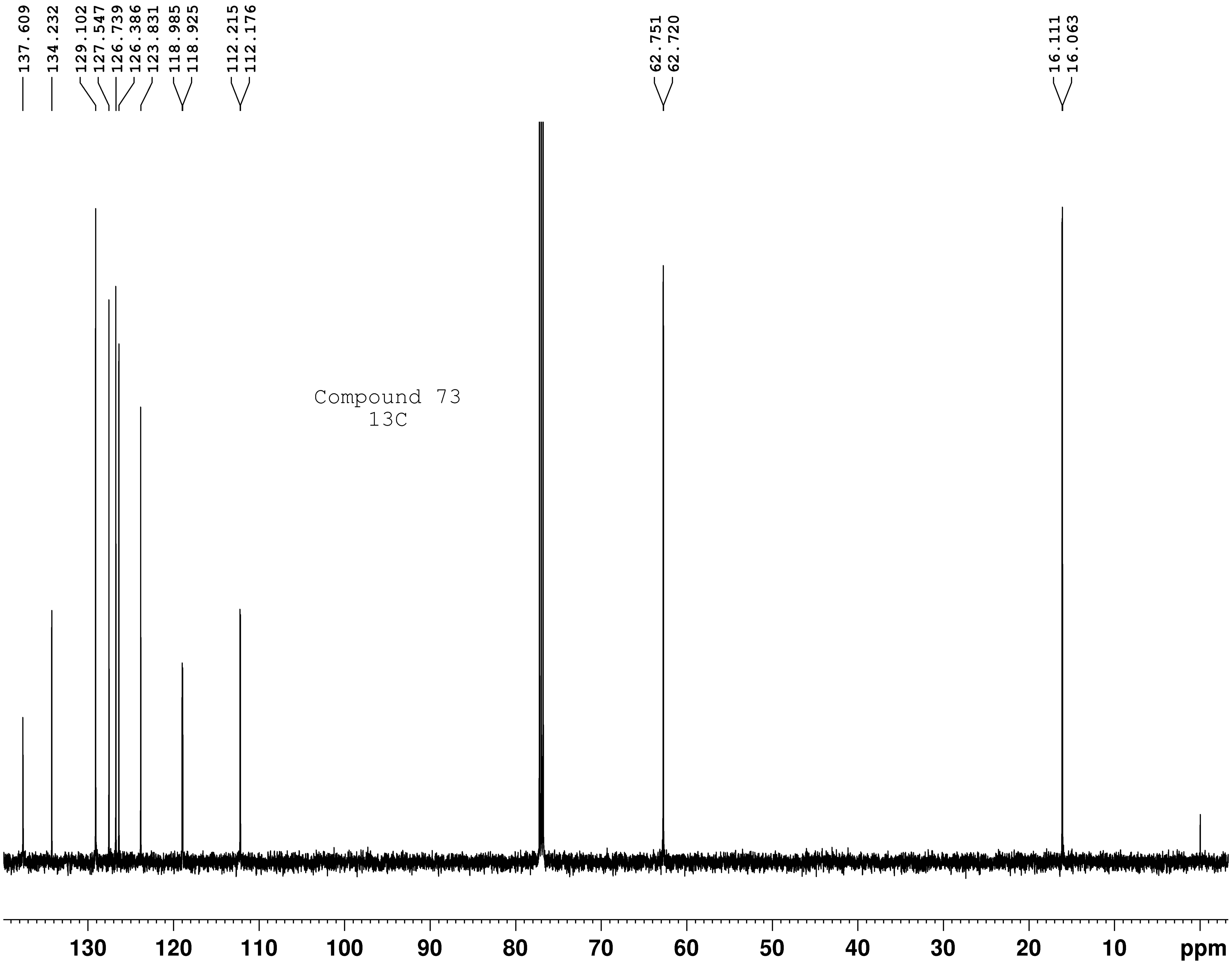

Current Data Parameters
NAME CE-107A
EXPNO 12
PROCNO 1

F2 - Acquisition Parameters
Date_ 20220221
Time 17.42 h
INSTRUM spect
PROBHD Z847801_0047 (
PULPROG zgdc30
TD 32768
SOLVENT CDCl3
NS 128
DS 0
SWH 36057.691 Hz
FIDRES 2.200787 Hz
AQ 0.4543829 sec
RG 2050
DW 13.867 usec
DE 6.50 usec
TE 293.0 K
D1 1.50000000 sec
D11 0.03000000 sec
TD0 1
SFO1 150.8816889 MHz
NUC1 13C
P0 3.27 usec
P1 9.80 usec
PLW1 40.00000000 W
SFO2 599.9824003 MHz
NUC2 1H
CPDPRG[2 waltz16
PCPD2 70.00 usec
PLW2 20.00000000 W
PLW12 0.53044999 W

F2 - Processing parameters
SI 65536
SF 150.8651040 MHz
WDW EM
SSB 0
LB 1.00 Hz
GB 0
PC 1.40

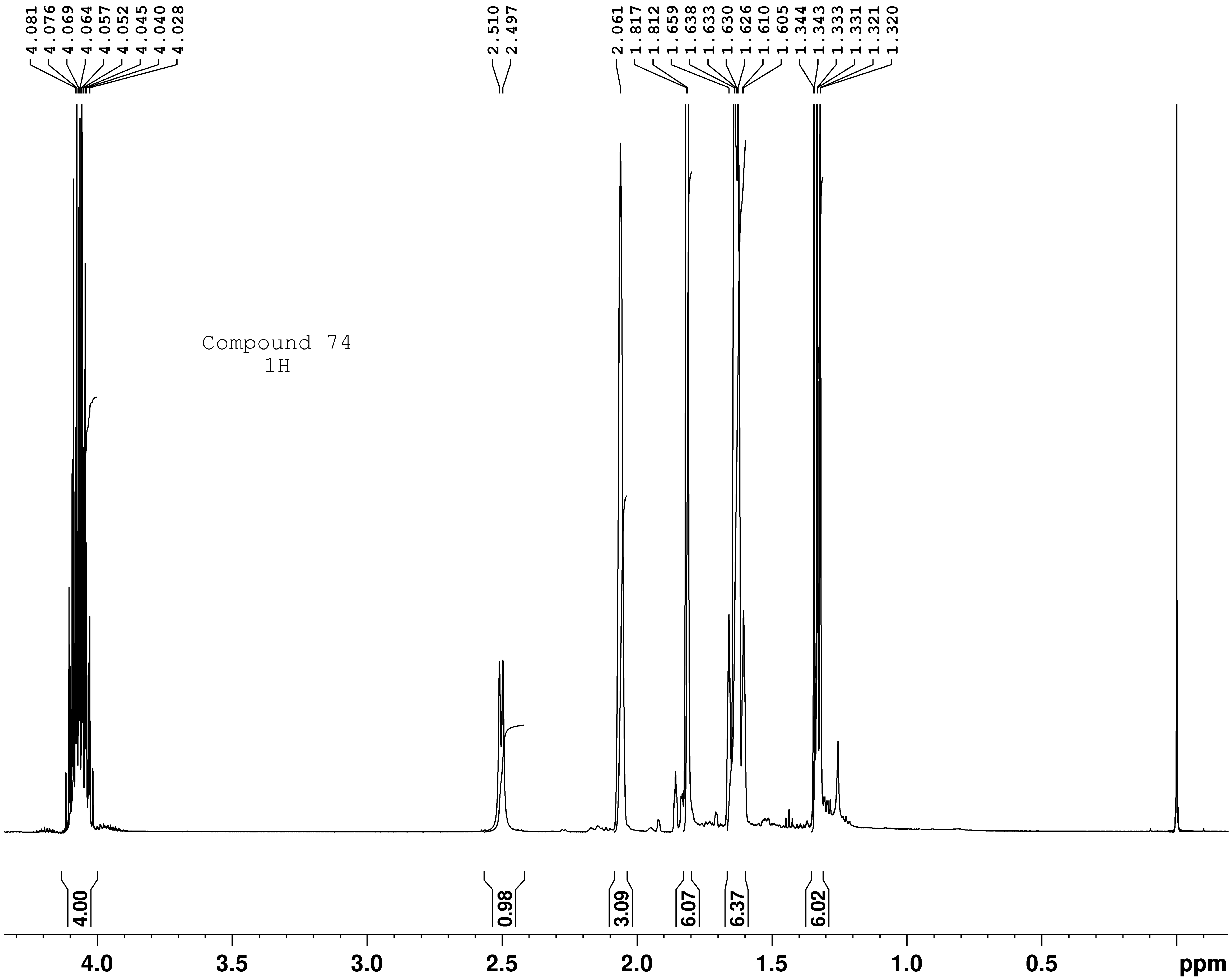

Current Data Parameters
NAME CE-115A
EXPNO 11
PROCNO 1

F2 - Acquisition Parameters
Date_ 20220221
Time 18.27 h
INSTRUM spect
PROBHD Z847801_0047 (
PULPROG zg30
TD 32768
SOLVENT CDCl3
NS 32
DS 0
SWH 9615.385 Hz
FIDRES 0.586877 Hz
AQ 1.7039360 sec
RG 64
DW 52.000 usec
DE 14.38 usec
TE 293.0 K
D1 1.00000000 sec
TD0 1
SFO1 599.9845606 MHz
NUC1 1H
P0 3.60 usec
P1 10.80 usec
PLW1 20.00000000 W

F2 - Processing parameters
SI 65536
SF 599.9799954 MHz
WDW EM
SSB 0
LB 0 Hz
GB 0
PC 1.00

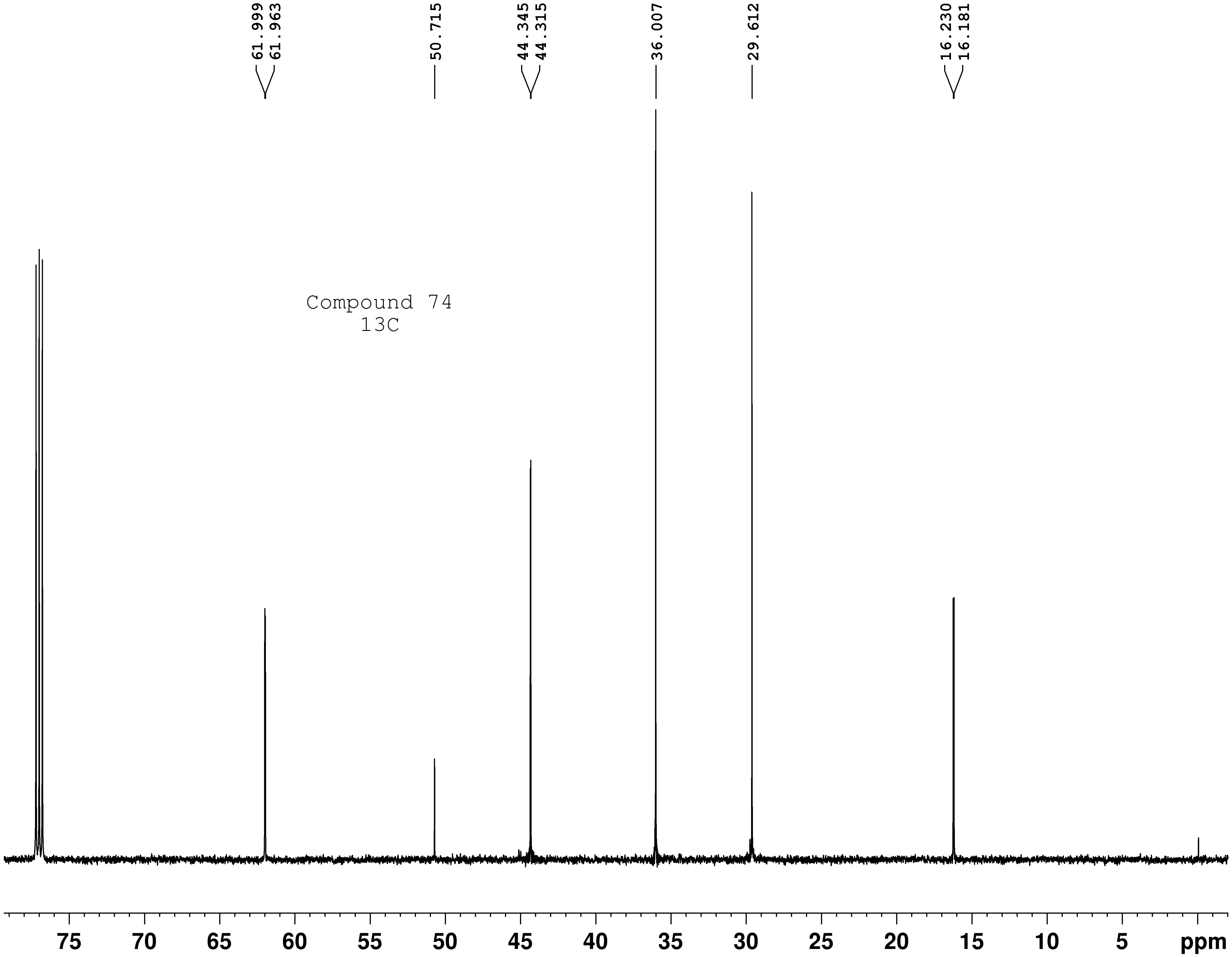


```
Current Data Parameters
NAME             CE-115A
EXPNO                 12
PROCNO                 1

F2 - Acquisition Parameters
Date_           20220221
Time               18.32 h
INSTRUM            spect
PROBHD   Z847801_0047 (
PULPROG          zgdc30
TD                 32768
SOLVENT            CDCl3
NS                   128
DS                     0
SWH            36057.691 Hz
FIDRES          2.200787 Hz
AQ             0.4543829 sec
RG                  2050
DW                13.867 usec
DE                  6.50 usec
TE                 293.0 K
D1            1.50000000 sec
D11           0.03000000 sec
TD0                    1
SFO1         150.8816889 MHz
NUC1                 13C
P0                  3.27 usec
P1                  9.80 usec
PLW1         40.00000000 W
SFO2         599.9824003 MHz
NUC2                  1H
CPDPRG[2        waltz16
PCPD2              70.00 usec
PLW2         20.00000000 W
PLW12         0.53044999 W

F2 - Processing parameters
SI                 65536
SF           150.8651013 MHz
WDW                   EM
SSB                    0
LB                  1.00 Hz
GB                     0
PC                  1.40
```

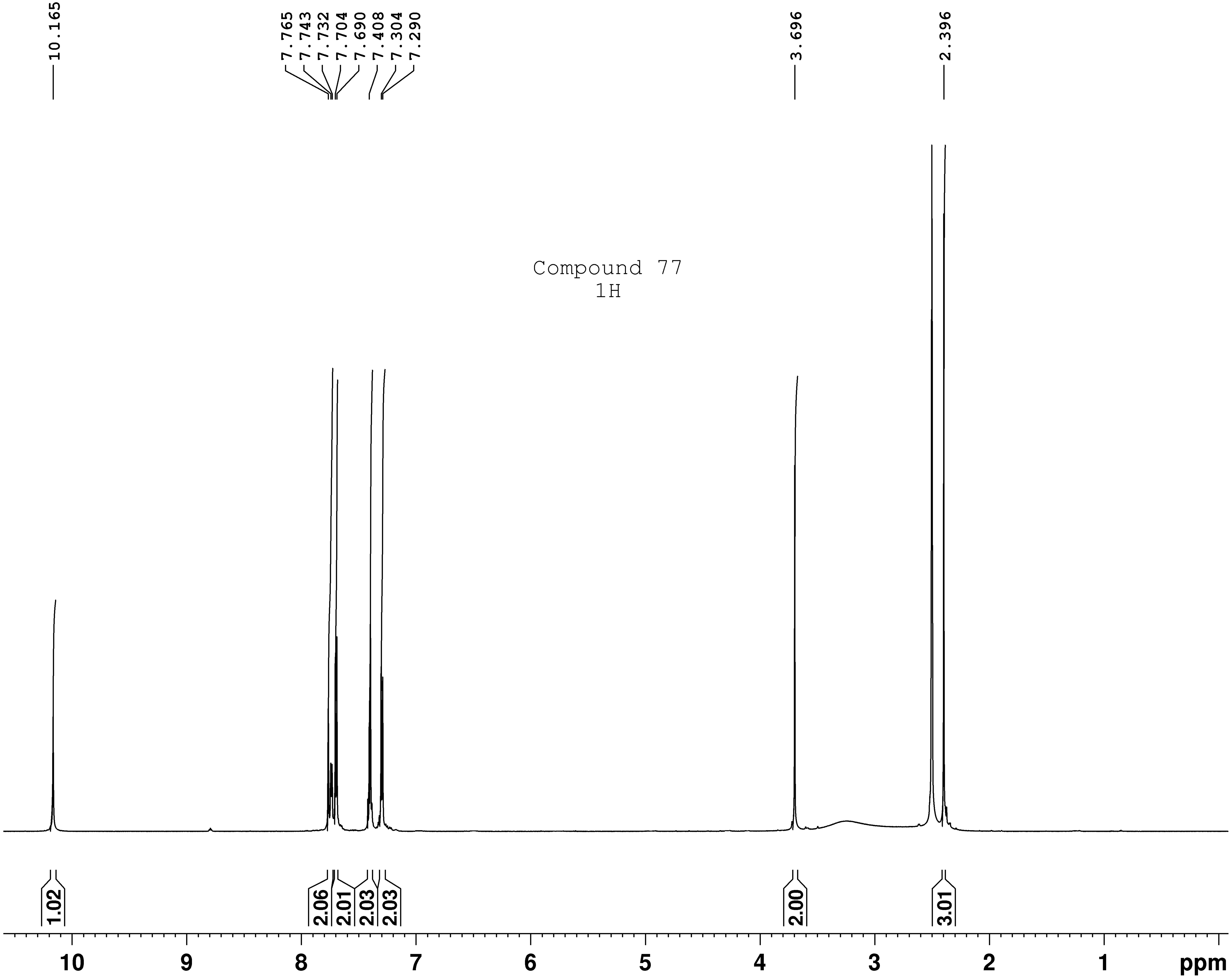

Current Data Parameters
NAME CD-195A
EXPNO 11
PROCNO 1

F2 - Acquisition Parameters
Date_ 20200723
Time 19.17 h
INSTRUM spect
PROBHD Z847801_0047 (
PULPROG zg30
TD 32768
SOLVENT DMSO
NS 32
DS 0
SWH 9615.385 Hz
FIDRES 0.586877 Hz
AQ 1.7039360 sec
RG 181
DW 52.000 usec
DE 14.37 usec
TE 293.0 K
D1 1.00000000 sec
TD0 1
SFO1 600.0145608 MHz
NUC1 1H
P0 3.62 usec
P1 10.85 usec
PLW1 20.00000000 W

F2 - Processing parameters
SI 65536
SF 600.0100048 MHz
WDW EM
SSB 0
LB 0 Hz
GB 0
PC 1.00

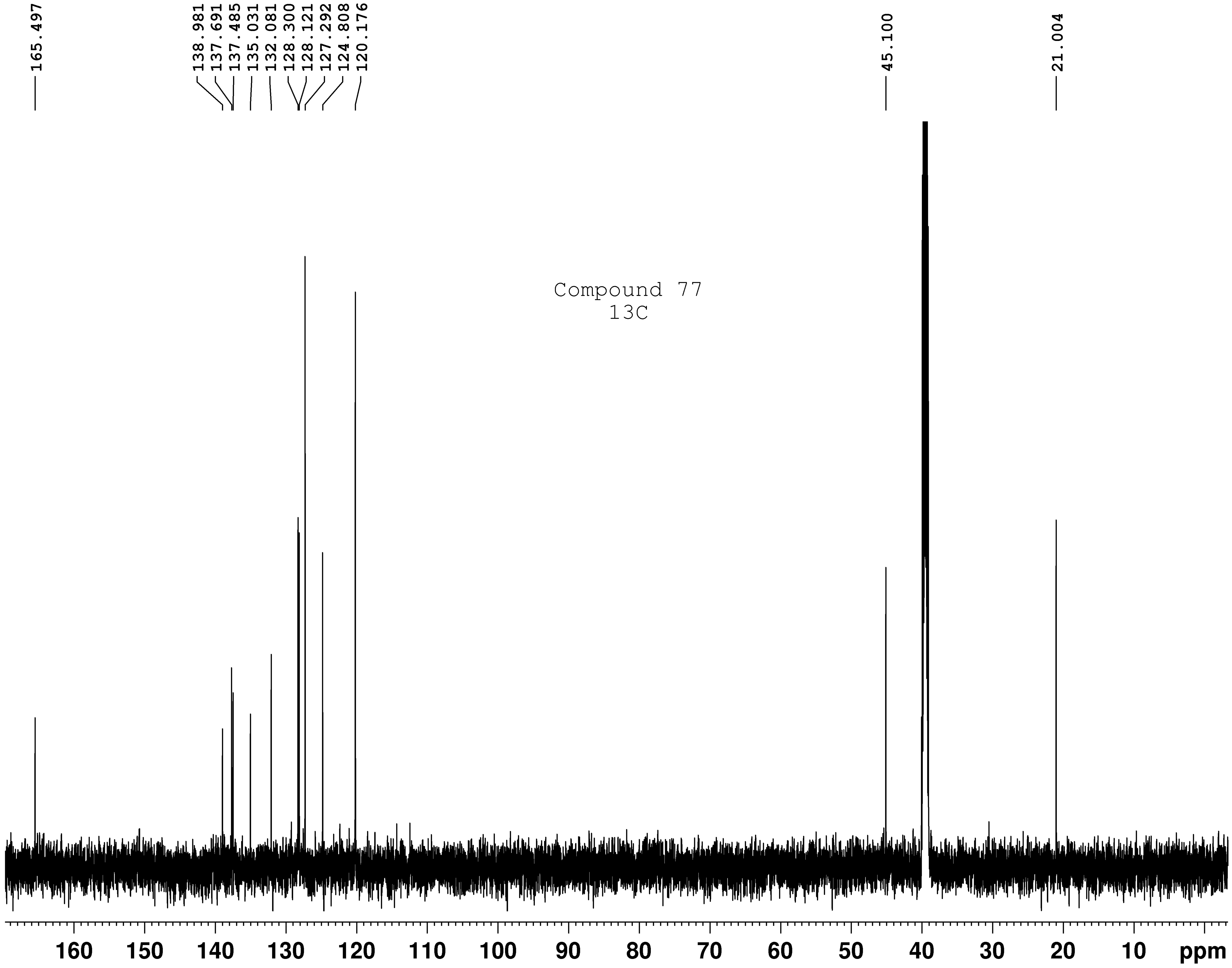


```
Current Data Parameters
NAME             CD-195A
EXPNO                 16
PROCNO                 1

F2 - Acquisition Parameters
Date_           20200723
Time               20.38 h
INSTRUM            spect
PROBHD   Z847801_0047 (
PULPROG          zgdc30
TD                 32768
SOLVENT             DMSO
NS                   128
DS                     0
SWH            36057.691 Hz
FIDRES          2.200787 Hz
AQ             0.4543829 sec
RG                  2050
DW                13.867 usec
DE                  6.50 usec
TE                 293.0 K
D1            1.50000000 sec
D11           0.03000000 sec
TD0                    1
SFO1         150.8892338 MHz
NUC1                 13C
P0                  3.27 usec
P1                  9.80 usec
PLW1         40.00000000 W
SFO2         600.0124004 MHz
NUC2                  1H
CPDPRG[2         waltz16
PCPD2              90.00 usec
PLW2         20.00000000 W
PLW12         0.33800000 W

F2 - Processing parameters
SI                 65536
SF           150.8727013 MHz
WDW                   EM
SSB                    0
LB                  1.00 Hz
GB                     0
PC                  1.40
```

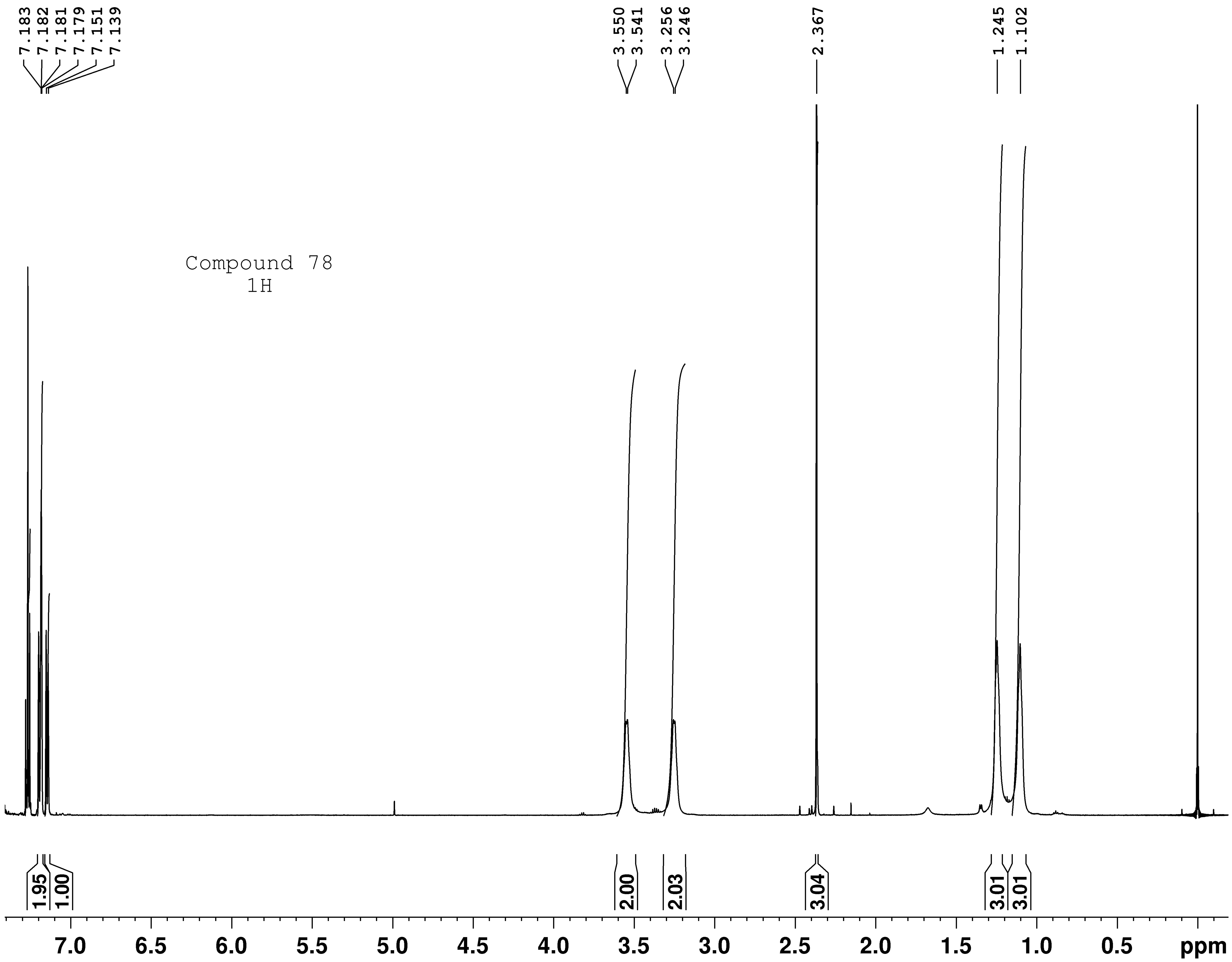

Current Data Parameters
NAME 80-CD-202A
EXPNO 11
PROCNO 1

F2 - Acquisition Parameters
Date_ 20220112
Time 17.40 h
INSTRUM spect
PROBHD Z847801_0047 (
PULPROG zg30
TD 32768
SOLVENT CDCl3
NS 32
DS 0
SWH 9615.385 Hz
FIDRES 0.586877 Hz
AQ 1.7039360 sec
RG 203
DW 52.000 usec
DE 14.38 usec
TE 293.0 K
D1 1.00000000 sec
TD0 1
SFO1 599.9845606 MHz
NUC1 1H
P0 3.60 usec
P1 10.80 usec
PLW1 20.00000000 W

F2 - Processing parameters
SI 65536
SF 599.9800111 MHz
WDW EM
SSB 0
LB 0 Hz
GB 0
PC 1.00

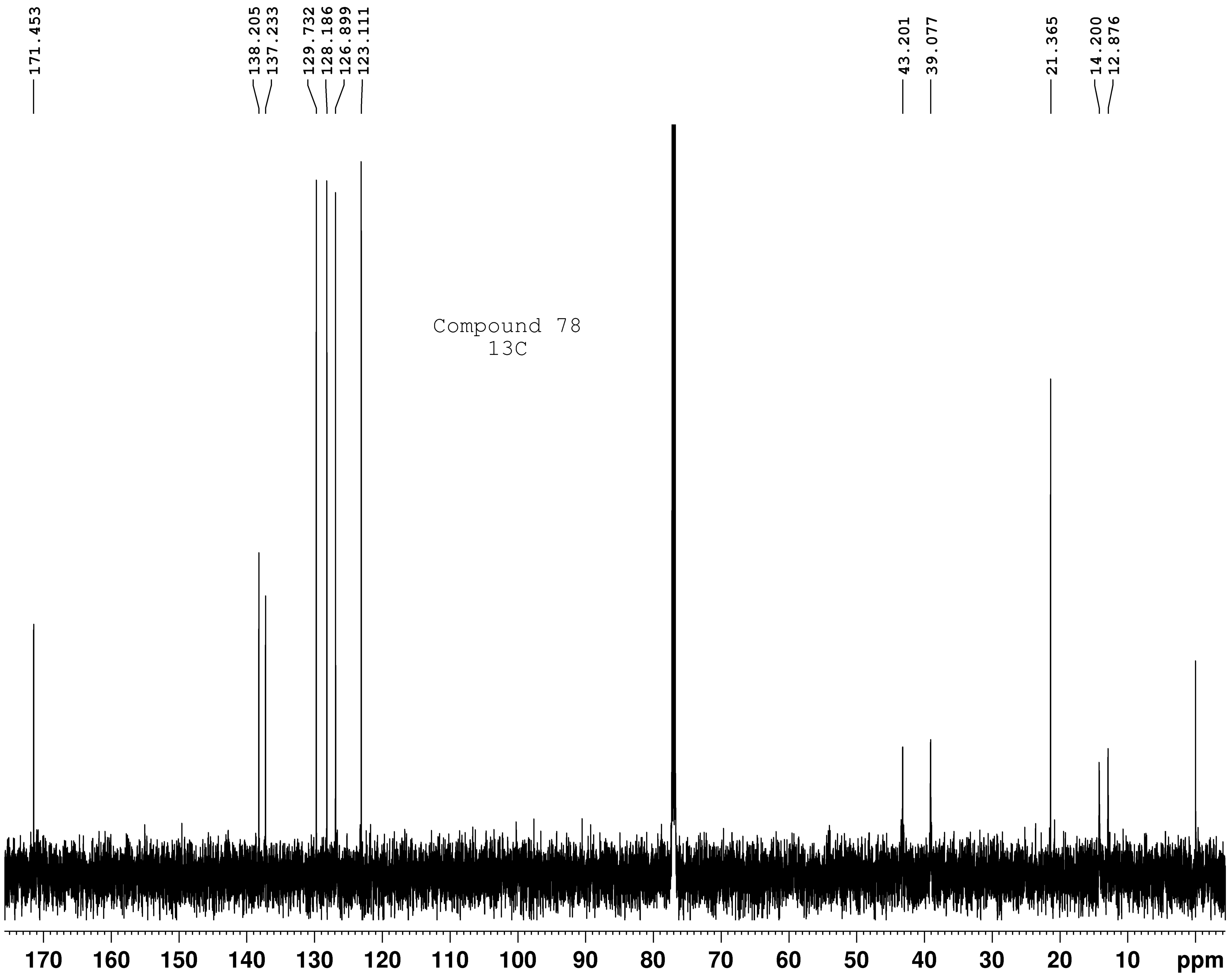


```
Current Data Parameters
NAME            CD-202A
EXPNO                12
PROCNO                1

F2 - Acquisition Parameters
Date_          20220112
Time              17.45 h
INSTRUM           spect
PROBHD   Z847801_0047 (
PULPROG          zgdc30
TD                32768
SOLVENT           CDCl3
NS                  128
DS                    0
SWH           36057.691 Hz
FIDRES         2.200787 Hz
AQ            0.4543829 sec
RG                 2050
DW               13.867 usec
DE                 6.50 usec
TE                293.1 K
D1           1.50000000 sec
D11          0.03000000 sec
TD0                   1
SFO1        150.8816889 MHz
NUC1                13C
P0                 3.27 usec
P1                 9.80 usec
PLW1        40.00000000 W
SFO2        599.9824003 MHz
NUC2                 1H
CPDPRG[2        waltz16
PCPD2             70.00 usec
PLW2        20.00000000 W
PLW12        0.53044999 W

F2 - Processing parameters
SI                65536
SF          150.8650977 MHz
WDW                  EM
SSB                   0
LB                 1.00 Hz
GB                    0
PC                 1.40
```

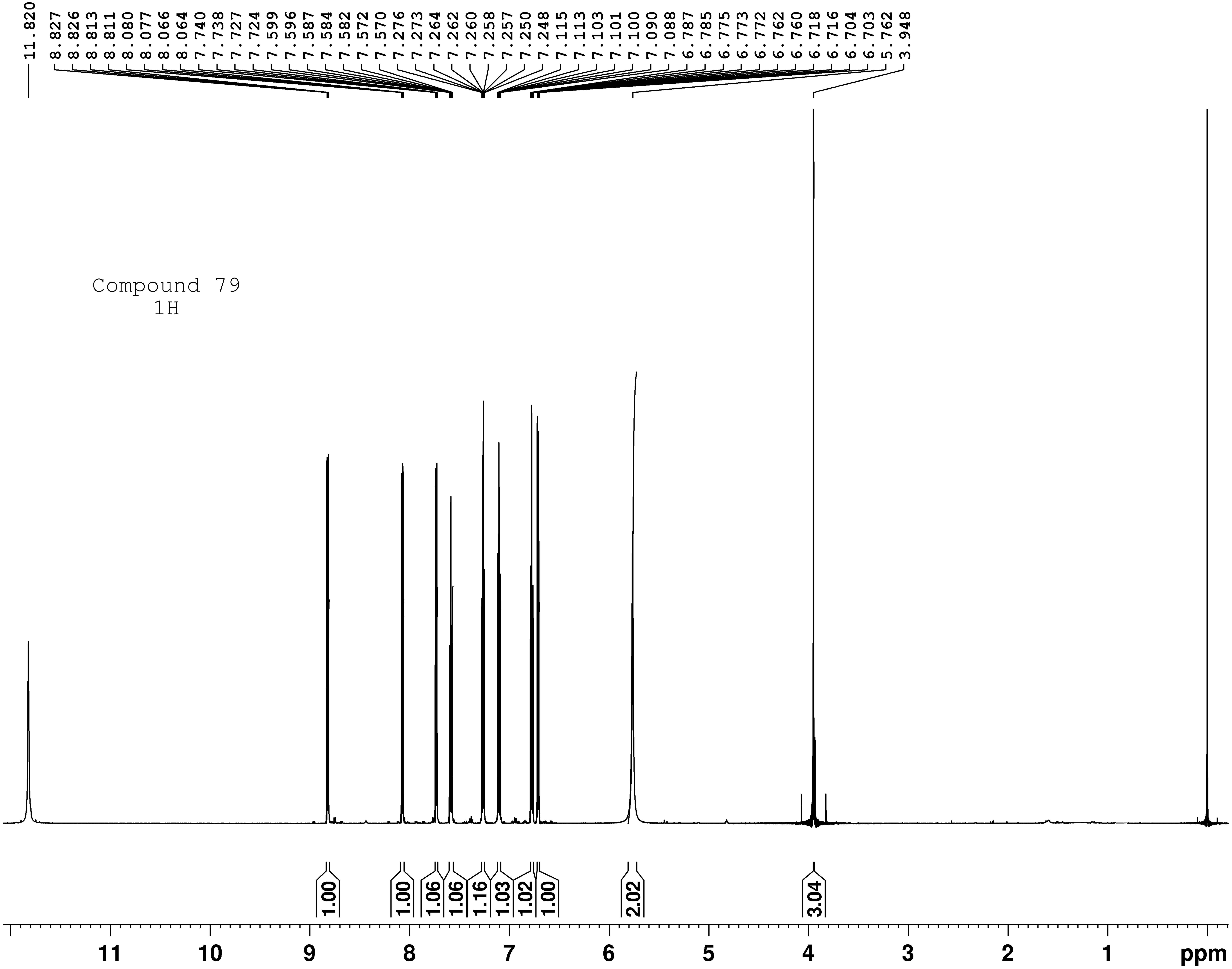


```
Current Data Parameters
NAME          81-CD-152A
EXPNO                 11
PROCNO                 1

F2 - Acquisition Parameters
Date_           20220118
Time               17.28 h
INSTRUM            spect
PROBHD   Z847801_0047 (
PULPROG             zg30
TD                 32768
SOLVENT            CDCl3
NS                    32
DS                     0
SWH            9615.385 Hz
FIDRES         0.586877 Hz
AQ            1.7039360 sec
RG                   161
DW               52.000 usec
DE                14.38 usec
TE                293.0 K
D1           1.00000000 sec
TD0                    1
SFO1        599.9845606 MHz
NUC1                  1H
P0                  3.60 usec
P1                 10.80 usec
PLW1        20.00000000 W

F2 - Processing parameters
SI                 65536
SF          599.9800158 MHz
WDW                   EM
SSB                    0
LB                     0 Hz
GB                     0
PC                  1.00
```

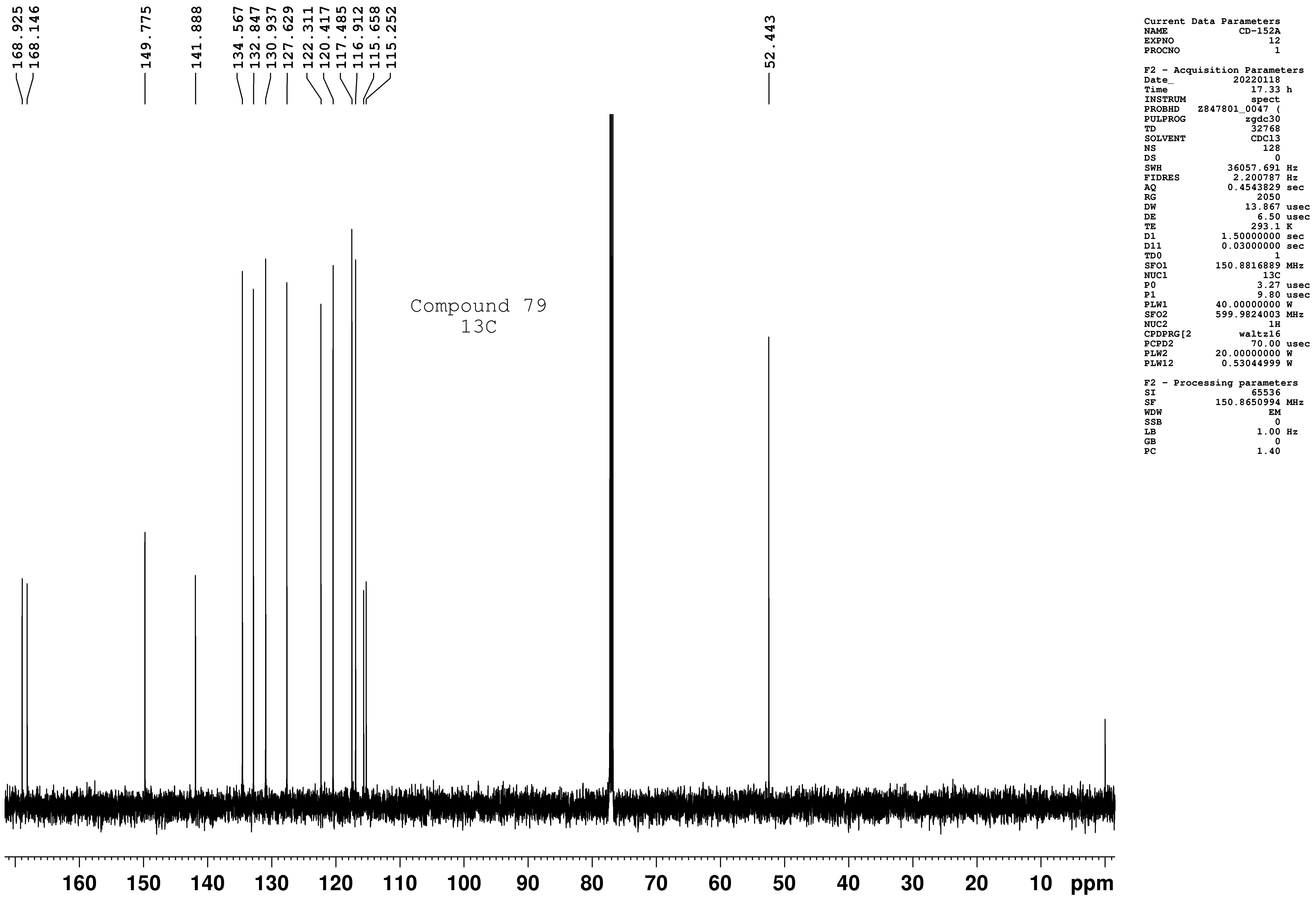
168.925
168.146
149.775
141.888
134.567
132.847
130.937
127.629
122.311
120.417
117.485
116.912
115.658
115.252
52.443
Compound 79
13C
160
150
140
130
120
110
100
90
80
70
60
50
40
30
20
10
ppm
Current Data Parameters
NAME CD-152A
EXPNO 12
PROCNO 1
F2 - Acquisition Parameters
Date_ 20220118
Time 17.33 h
INSTRUM spect
PROBHD Z847801_0047 (
PULPROG zgdc30
TD 32768
SOLVENT CDCl3
NS 128
DS 0
SWH 36057.691 Hz
FIDRES 2.200787 Hz
AQ 0.4543829 sec
RG 2050
DW 13.867 usec
DE 6.50 usec
TE 293.1 K
D1 1.50000000 sec
D11 0.03000000 sec
TD0 1
SFO1 150.8816889 MHz
NUC1 13C
P0 3.27 usec
P1 9.80 usec
PLW1 40.00000000 W
SFO2 599.9824003 MHz
NUC2 1H
CPDPRG[2 waltz16
PCPD2 70.00 usec
PLW2 20.00000000 W
PLW12 0.53044999 W
F2 - Processing parameters
SI 65536
SF 150.8650994 MHz
WDW EM
SSB 0
LB 1.00 Hz
GB 0
PC 1.40

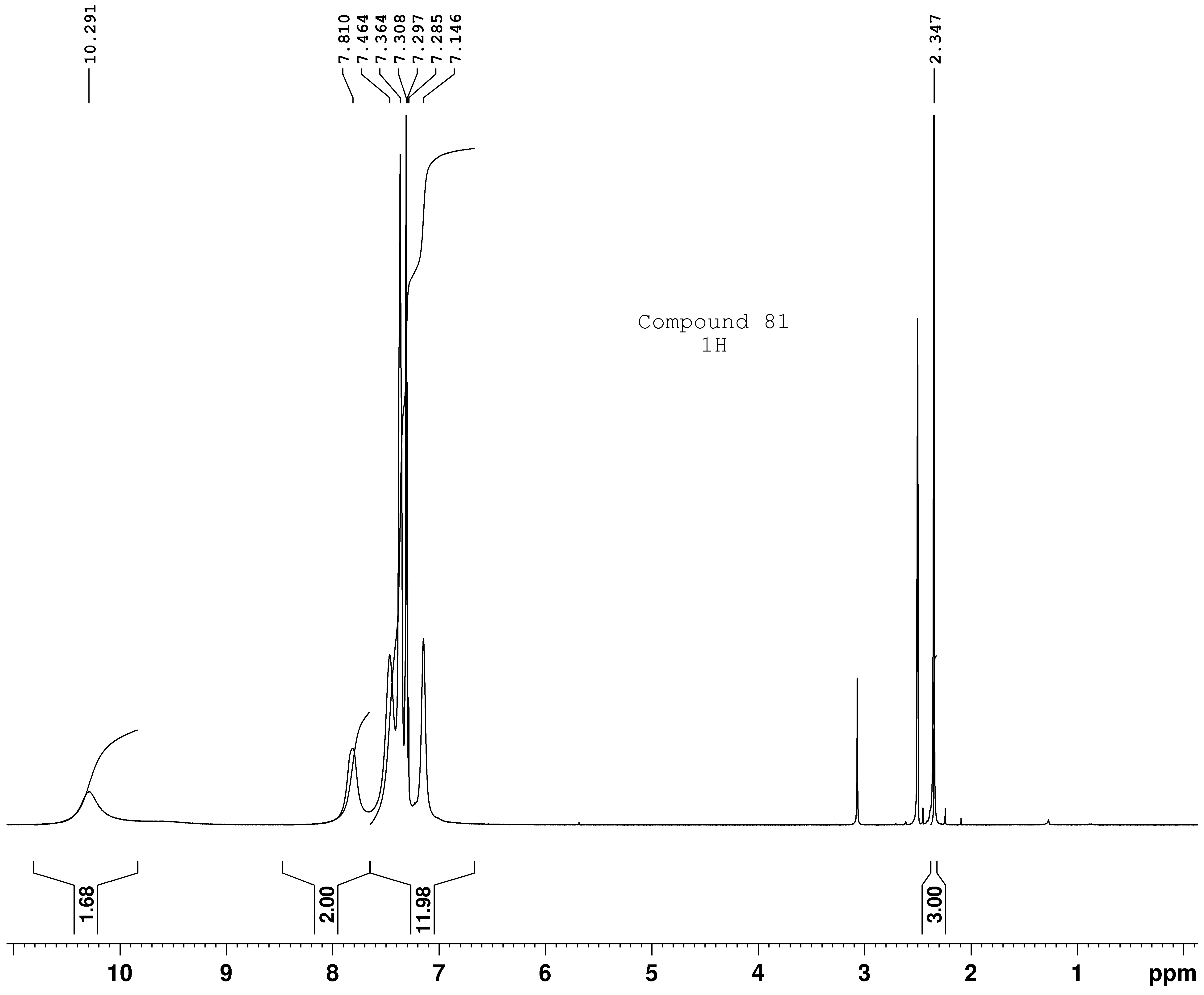

**Current Data Parameters**		
NAME	CD-68A	
EXPNO	11	
PROCNO	1	
**F2 - Acquisition Parameters**		
Date_	20220225	
Time	20.12	h
INSTRUM	spect	
PROBHD	Z847801_0047 (	
PULPROG	zg30	
TD	32768	
SOLVENT	DMSO	
NS	32	
DS	0	
SWH	9615.385	Hz
FIDRES	0.586877	Hz
AQ	1.7039360	sec
RG	90.5	
DW	52.000	usec
DE	14.38	usec
TE	353.2	K
D1	1.00000000	sec
TD0	1	
SFO1	599.9845606	MHz
NUC1	1H	
P0	3.60	usec
P1	10.80	usec
PLW1	20.00000000	W
**F2 - Processing parameters**		
SI	65536	
SF	599.9800043	MHz
WDW	EM	
SSB	0	
LB	0	Hz
GB	0	
PC	1.00	

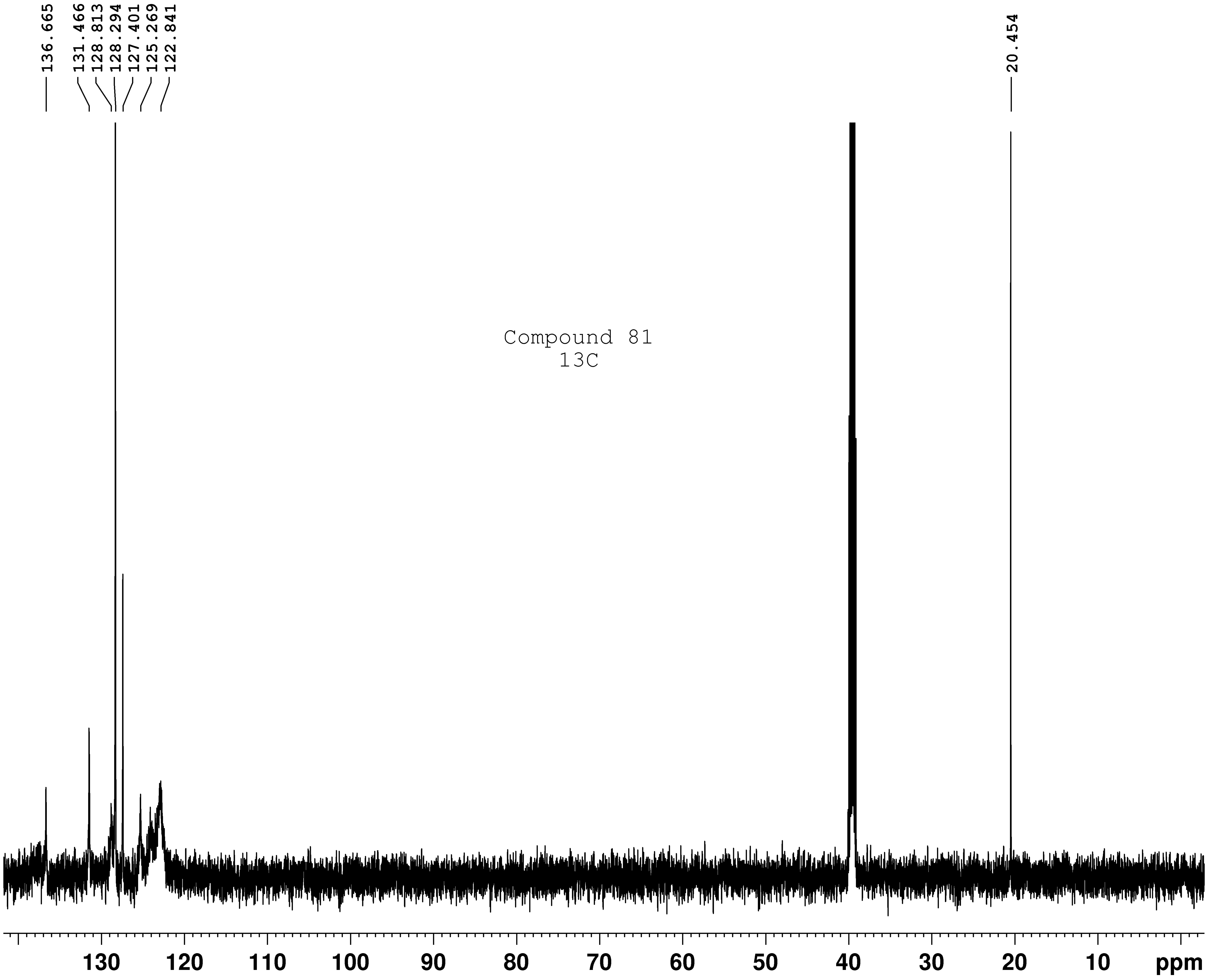

Current Data Parameters
NAME CD-68A
EXPNO 12
PROCNO 1

F2 - Acquisition Parameters
Date_ 20220225
Time 20.18 h
INSTRUM spect
PROBHD Z847801_0047 (
PULPROG zgdc30
TD 32768
SOLVENT DMSO
NS 128
DS 0
SWH 36057.691 Hz
FIDRES 2.200787 Hz
AQ 0.4543829 sec
RG 2050
DW 13.867 usec
DE 6.50 usec
TE 353.2 K
D1 1.50000000 sec
D11 0.03000000 sec
TD0 1
SFO1 150.8816889 MHz
NUC1 13C
P0 3.27 usec
P1 9.80 usec
PLW1 40.00000000 W
SFO2 599.9824003 MHz
NUC2 1H
CPDPRG[2 waltz16
PCPD2 70.00 usec
PLW2 20.00000000 W
PLW12 0.53044999 W

F2 - Processing parameters
SI 65536
SF 150.8652289 MHz
WDW EM
SSB 0
LB 1.00 Hz
GB 0
PC 1.40

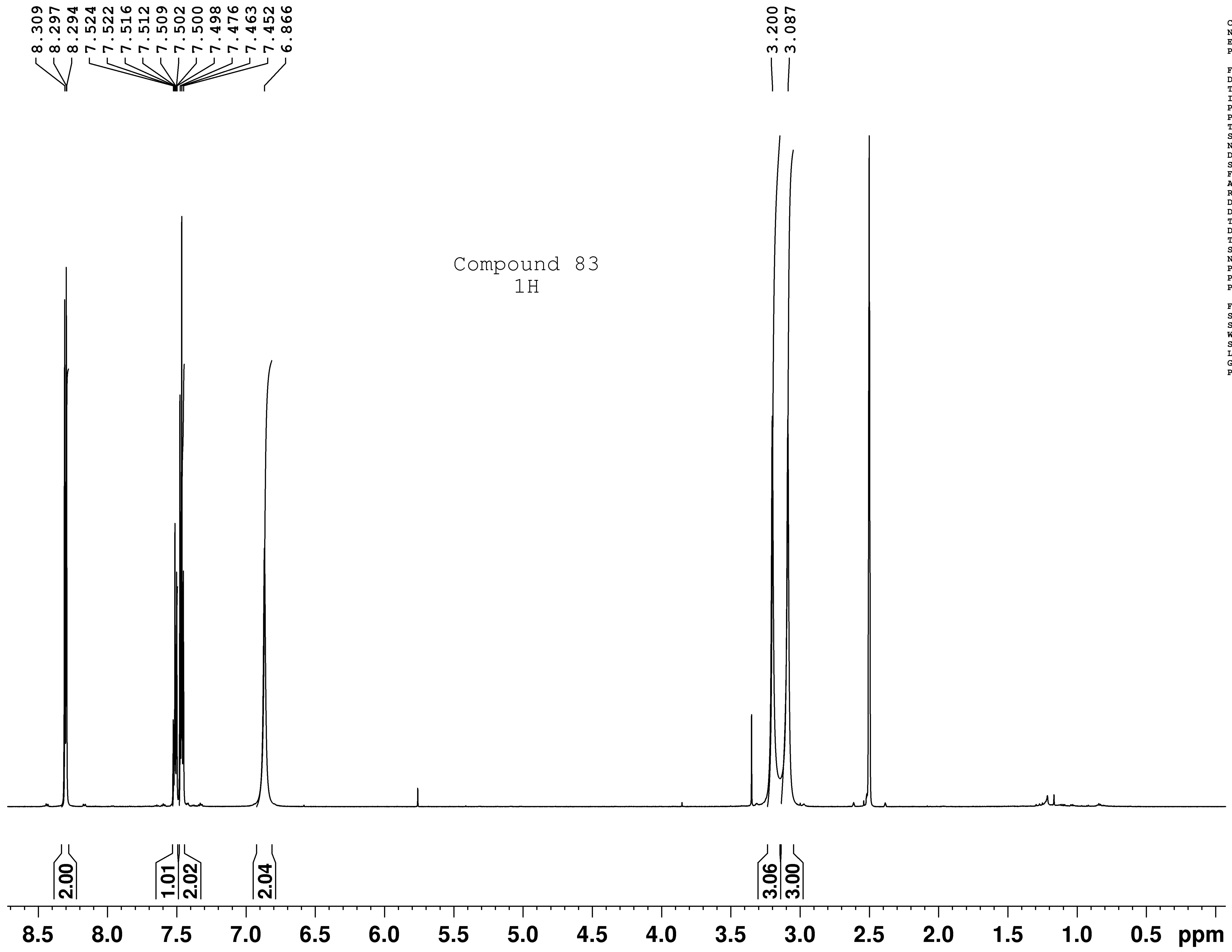

Current Data Parameters
NAME CE-60A
EXPNO 11
PROCNO 1

F2 - Acquisition Parameters
Date_ 20220224
Time 22.27 h
INSTRUM spect
PROBHD Z847801_0047 (
PULPROG zg30
TD 32768
SOLVENT DMSO
NS 16
DS 0
SWH 9615.385 Hz
FIDRES 0.586877 Hz
AQ 1.7039360 sec
RG 144
DW 52.000 usec
DE 14.38 usec
TE 293.0 K
D1 1.00000000 sec
TD0 1
SFO1 599.9845606 MHz
NUC1 1H
P0 3.60 usec
P1 10.80 usec
PLW1 20.00000000 W

F2 - Processing parameters
SI 65536
SF 599.9800038 MHz
WDW EM
SSB 0
LB 0 Hz
GB 0
PC 1.00

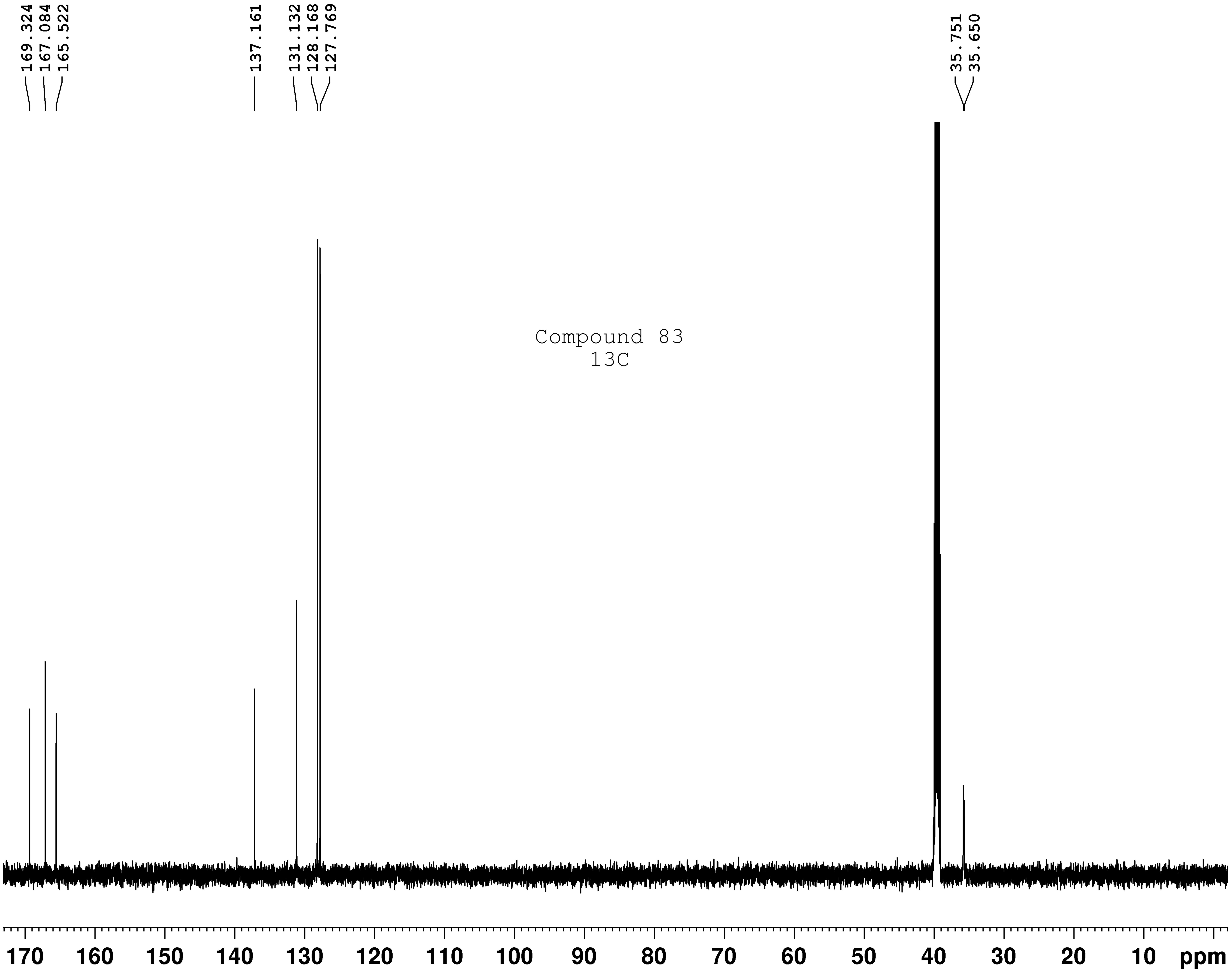

Current Data Parameters
NAME CE-60A
EXPNO 12
PROCNO 1

F2 - Acquisition Parameters
Date_ 20220224
Time 22.32 h
INSTRUM spect
PROBHD Z847801_0047 (
PULPROG zgdc30
TD 32768
SOLVENT DMSO
NS 128
DS 0
SWH 36057.691 Hz
FIDRES 2.200787 Hz
AQ 0.4543829 sec
RG 2050
DW 13.867 usec
DE 6.50 usec
TE 293.1 K
D1 1.50000000 sec
D11 0.03000000 sec
TD0 1
SFO1 150.8816889 MHz
NUC1 13C
P0 3.27 usec
P1 9.80 usec
PLW1 40.00000000 W
SFO2 599.9824003 MHz
NUC2 1H
CPDPRG[2 waltz16
PCPD2 70.00 usec
PLW2 20.00000000 W
PLW12 0.53044999 W

F2 - Processing parameters
SI 65536
SF 150.8651586 MHz
WDW EM
SSB 0
LB 1.00 Hz
GB 0
PC 1.40
